# Supplementary material for: Analysis of maternal and child health spillover effects in PEPFAR countries
Source: BMJ Open. 2023 Dec 21;13(12):e070221. doi: 10.1136/bmjopen-2022-070221 (PMC10748962; doi:10.1136/bmjopen-2022-070221)
Supplement: Supplementary data [file bmjopen-2022-070221supp001.pdf]

Analysis of Maternal and Child Health Spillover Effects in PEPFAR Countries Supplemental Materials

Gary Gaumer[1], William Crown[1], Jennifer Kates[2], Yiqun Luan[1], Dhwani Hariharan[1], Monica Jordan[1], Clare Hurley[1], AK Nandakumar[1]

[1]Institute for Global Health and Development, The Heller School for Social Policy and Management, Brandeis University, Waltham, MA, USA
[2]Kaiser Family Foundation, Global Health & HIV Policy, Washington, DC, USA

Supplement Number	Title
S1	Country list
S2	Data source and covariate inclusion rationale
S3	Full model of unlogged regression of seven outcomes from traditional DID
S4	Full model of logged regression of seven outcomes from traditional DID
S5	Full model of unlogged regression of seven outcomes from staggered DID
S6	Full model of logged regression of seven outcomes from staggered DID
S7	Parallel pre-trend assumption test for traditional DID
S8	Parallel pre-trend assumption test for staggered DID
S9	Placebo test for staggered DID
S10	Estimation results from logarithmically transformed equations 1 and 3

S1. Country list

All PEPFAR Funded LMICs	COPs	Non-COP PEPFAR countries	High intensity PEFAR funding per capita group	Middle intensity PEPFAR funding per capita group	Low intensity PEPFAR funding per capita group	Control group
Afghanistan	Angola	Afghanistan	Angola	Albania	Afghanistan	Algeria
Albania	Botswana	Albania	Botswana	Barbados	Armenia	American Samoa
Angola	Burundi	Armenia	Burundi	Belize	Bangladesh	Antigua and Barbuda
Armenia	Cambodia	Bangladesh	Cambodia	Benin	Bolivia	Argentina
Bangladesh	Cameroon	Barbados	Cameroon	Congo, Dem. Rep.	Brazil	Azerbaijan
Barbados	Congo, Dem. Rep.	Belize	Cote d'Ivoire	El Salvador	Burkina Faso	Belarus
Belize	Cote d'Ivoire	Benin	Djibouti	Eritrea	Central African Republic	Bhutan
Benin	Dominican Republic	Bolivia	Dominican Republic	Estonia	Chad	Bosnia and Herzegovina
Bolivia	Eswatini	Brazil	Eswatini	Gabon	China	Bulgaria
Botswana	Ethiopia	Burkina Faso	Ethiopia	Gambia, The	Comoros	Cabo Verde
Brazil	Ghana	Central African Republic	Ghana	Georgia	Congo, Rep.	Chile
Burkina Faso	Guyana	Chad	Guyana	Guatemala	Guinea-Bissau	Colombia
Burundi	Haiti	China	Haiti	Guinea	India	Costa Rica
Cambodia	India	Comoros	Honduras	Kyrgyz Republic	Indonesia	Croatia
Cameroon	Indonesia	Congo, Rep.	Jamaica	Lao PDR	Jordan	Cuba
Central African Republic	Kenya	Djibouti	Kenya	Madagascar	Kazakhstan	Czech Republic
Chad	Lesotho	El Salvador	Lesotho	Mali	Mauritania	Dominica
China	Malawi	Eritrea	Liberia	Moldova	Mexico	Ecuador
Comoros	Mozambique	Estonia	Malawi	Myanmar	Niger	Egypt, Arab Rep.
Congo, Dem. Rep.	Myanmar	Gabon	Mozambique	Nepal	North Macedonia	Equatorial Guinea
Congo, Rep.	Namibia	Gambia, The	Namibia	Nicaragua	Pakistan	Fiji
Cote d'Ivoire	Nigeria	Georgia	Nigeria	Papua New Guinea	Peru	Grenada
Djibouti	Papua New Guinea	Guatemala	Rwanda	Samoa	Philippines	Hungary
Dominican Republic	Rwanda	Guinea	Senegal	Sao Tome and Principe	Romania	Iran, Islamic Rep.
El Salvador	South Africa	Guinea-Bissau	South Africa	Sierra Leone	Russian Federation	Iraq
Eritrea	Tanzania	Honduras	Tanzania	Tajikistan	Seychelles	Kiribati
Estonia	Uganda	Jamaica	Uganda	Thailand	Suriname	Korea, Dem. People's Rep.
Eswatini	Ukraine	Jordan	Vietnam	Timor-Leste	Togo	Kosovo
Ethiopia	Vietnam	Kazakhstan	Zambia	Trinidad and Tobago	Turkmenistan	Latvia
Gabon	Zambia	Kyrgyz Republic	Zimbabwe	Ukraine	Uzbekistan	Lebanon
Gambia, The	Zimbabwe	Lao PDR				Libya
Georgia		Liberia				Lithuania
Ghana		Madagascar				Malaysia
Guatemala		Mali				Maldives
Guinea		Mauritania				Marshall Islands
Guinea-Bissau		Mexico				Mauritius
Guyana		Moldova				Mayotte
Haiti		Nepal				Micronesia, Fed. Sts.
Honduras		Nicaragua				Mongolia
India		Niger				Montenegro
Indonesia		North Macedonia				Morocco
Jamaica		Pakistan				Nauru
Jordan		Peru				Northern Mariana Islands
Kazakhstan		Philippines				Oman
Kenya		Romania				Palau
Kyrgyz Republic		Russian Federation				Panama
Lao PDR		Samoa				Paraguay
Lesotho		Sao Tome and Principe				Poland
Liberia		Senegal				Serbia
Madagascar		Seychelles				Serbia and Montenegro
Malawi		Sierra Leone				Slovak Republic
Mali		Suriname				Solomon Islands
Mauritania		Tajikistan				Somalia

Mexico		Thailand				Sri Lanka
Moldova		Timor-Leste				St. Kitts and Nevis
Mozambique		Togo				St. Lucia
Myanmar		Trinidad and Tobago				St. Vincent and the Grenadines
Namibia		Turkmenistan				Syrian Arab Republic
Nepal		Uzbekistan				Tonga
Nicaragua						Tunisia
Niger						Turkey
Nigeria						Tuvalu
North Macedonia						Uruguay
Pakistan						Vanuatu
Papua New Guinea						Venezuela, RB
Peru						West Bank and Gaza
Philippines						Yemen, Rep.
Romania						
Russian Federation						
Rwanda						
Samoa						
Sao Tome and Principe						
Senegal						
Seychelles						
Sierra Leone						
South Africa						
Suriname						
Tajikistan						
Tanzania						
Thailand						
Timor-Leste						
Togo						
Trinidad and Tobago						
Turkmenistan						
Uganda						
Ukraine						
Uzbekistan						
Vietnam						
Zambia						
Zimbabwe						

Notes: COP= country operational plans; LMICs = low- and middle-income countries; PEPFAR= President's Emergency Plan for AIDS Relief

S2. Data source and covariate inclusion rationale

Covariates	Data source	Rationale for inclusion
GDP per capita (constant USD)	WDI, https://datatopics.worldbank.org/world-development-indicators/	Income levels in country will contribute to accessibility of health facilities; fraction of population that are able to afford copayments for domestic health services
Total population	United Nations, Department of Economic and Social Affairs, Population Division (2019). World Population Prospects 2019, Online Edition. Rev, https://population.un.org/wpp/	The scale of the country may influence the size/scope/and financing capabilities of country governments
Life expectancy at birth (years)	WDI, https://datatopics.worldbank.org/world-development-indicators/	A general indicator of the health level of the population
Fertility rate, total (births per woman)	WDI, https://datatopics.worldbank.org/world-development-indicators/	Fertility rate will be an indicator of the demand for MCH services and an important dimension of how fast the population growing
Percent urban population (of total population)	WDI, https://datatopics.worldbank.org/world-development-indicators/	Disease spread differs, access to HIV and other health care services is also different by residential area
School enrollment, secondary (% gross)	WDI, https://datatopics.worldbank.org/world-development-indicators/	Level of education of the population matters in risk avoidance, and care seeking
HIV prevalence (% of population ages 15-49)	WDI, https://datatopics.worldbank.org/world-development-indicators/ (from UNAIDS); The Global Burden of Disease Collaborative Network, Global Burden of Disease Study 2019 (GBD 2019) Results. Seattle, United States: Institute for Health Metrics and Evaluation (IHME), 2020, http://ghdx.healthdata.org/gbd-results-tool.	The level of intensity of the epidemic in the population
non-PEPFAR donor spending on health per capita	OECD Creditor Reporting System database, https://stats.oecd.org/Index.aspx?DataSetCode=crs1	Other donors including the Global Fund, Gates, bilateral donors provide funding for HIV and other health purposes. This covariate would capture the intensity of these potentially confounding country programs
domestic health spending per capita	WDI, https://datatopics.worldbank.org/world-development-indicators/	Private spending on Health and Domestic Government spending on Health would indicate the intensity of domestic health activity in the country
Recipient of U.S. HIV funding prior to 2004 (dummy variable)	USAID, https://foreignassistance.gov/	Many countries began to receive US Aid for HIV programming prior to PEPFAR's passage in late 2003. This is a dummy variable (1=received early HIV aid) might indicate that a country has a 'running start" on setting up PEPFAR programming

WB country income classification	World Bank, https://datahelpdesk.worldbank.org/knowledgebase/articles/906519-world-bank-country-and-lending-groups	Aims to control for shared unobservable characteristics among recipients of the same income classification
Diphtheria prevalence in under 5 population (DPT immunization models only)	IHME, http://ghdx.healthdata.org/gbd-results-tool	The baseline incidence levels of one of these diseases, for example, would may indicate the level of demand for the vaccine by households or caregivers
Whooping cough prevalence in under 5 population (DPT immunization models only)	IHME, http://ghdx.healthdata.org/gbd-results-tool	
Tetanus prevalence in under 5 population (DPT immunization models only)	IHME, http://ghdx.healthdata.org/gbd-results-tool	
Hepatitis B prevalence in under 5 population (HepB3 immunization models only)	IHME, http://ghdx.healthdata.org/gbd-results-tool	
Measles prevalence (measles immunization models only)	IHME, http://ghdx.healthdata.org/gbd-results-tool	

Notes: COP= country operational plans; DPT=diphtheria, pertussis, tetanus; GDP=gross domestic product; HIV=human immunodeficiency virus; IHME=Institute for Health Metrics and Evaluation; LMICs = low- and middle-income countries; MCH=maternal child health; OECD=Organization for Economic Cooperation and Development; PEPFAR= President's Emergency Plan for AIDS Relief; WB=World Bank; WDI=World Development Indicators

S3. Full model of unlogged regression of seven outcomes from traditional DID

Table S3-1. Full model results for the indicator "Percent of children ages 12-23 months who received DPT vaccinations (3 doses)"

Variables	All PEPFAR countries		COP countries		Non-COP PEPFAR countries		High-intensity PEPFAR countries		Medium-intensity PEPFAR countries		Low-intensity PEPFAR countries	
	**Model 1**	**Model 2**	**Model 1**	**Model 2**	**Model 1**	**Model 2**	**Model 1**	**Model 2**	**Model 1**	**Model 2**	**Model 1**	**Model 2**
Time variable (=1 post-2004)	3.783***<br>(0.687)	3.308***<br>(0.422)	3.783***<br>(0.687)	3.301***<br>(0.416)	3.783***<br>(0.687)	3.293***<br>(0.409)	3.783***<br>(0.687)	3.287***<br>(0.413)	3.783***<br>(0.687)	3.293***<br>(0.408)	3.783***<br>(0.687)	3.260***<br>(0.405)
Intervention (=1 if PEPFAR)	-14.91***<br>(0.823)	-5.662***<br>(0.610)	-17.80***<br>(1.146)	-11.37***<br>(1.208)	-13.28***<br>(0.964)	-4.259***<br>(0.696)	-16.88***<br>(1.173)	-6.547***<br>(1.248)	-11.54***<br>(1.154)	-3.369***<br>(0.908)	-16.08***<br>(1.318)	-5.135***<br>(0.908)
Interaction term (PEPFAR impact estimate)	8.421***<br>(1.030)	8.810***<br>(0.647)	8.607***<br>(1.430)	8.693***<br>(0.920)	8.221***<br>(1.183)	8.889***<br>(0.725)	9.728***<br>(1.446)	9.380***<br>(0.856)	5.467***<br>(1.420)	6.635***<br>(1.009)	9.855***<br>(1.598)	10.07***<br>(0.903)
Country income level (=1 if middle income)		-5.021***<br>(0.660)		-6.596***<br>(0.857)		-2.787***<br>(0.792)		-5.729***<br>(0.888)		-1.991*<br>(0.897)		-2.623**<br>(0.995)
BL Population		-1.91e-09*<br>(0.000)		-7.54e-09***<br>(0.000)		-2.31e-09**<br>(0.000)		-1.21e-08<br>(0.000)		-5.88e-08***<br>(0.000)		-3.38e-09***<br>(0.000)
BL other donor health spending per capita		0.00376<br>(0.008)		-0.0194*<br>(0.008)		-0.00417<br>(0.008)		-0.0151<br>(0.008)		-0.0169*<br>(0.008)		-0.0166<br>(0.009)
BL domestic health spending per capita		-0.00460***<br>(0.001)		-0.00274<br>(0.001)		-0.00610***<br>(0.001)		-0.00586***<br>(0.001)		-0.00759***<br>(0.001)		-0.00415***<br>(0.001)
BL GDP per capita		0.000123*<br>(0.000)		-0.00000876<br>(0.000)		0.000142**<br>(0.000)		0.0000377<br>(0.000)		0.000178***<br>(0.000)		0.0000729<br>(0.000)
BL HIV prevalence (% of population ages 15-49)		0.827***<br>(0.071)		0.685***<br>(0.088)		-0.799*<br>(0.312)		0.481***<br>(0.077)		-1.664**<br>(0.555)		-1.519***<br>(0.412)
BL diphtheria prevalence (% of population, under 5)		-3912.0***<br>(535.878)		-5160.0***<br>(547.070)		-4392.1***<br>(924.111)		-3999.0***<br>(643.387)		-1502.8<br>(1678.940)		-5415.7***<br>(961.081)
BL tetanus prevalence (% of population, under 5)		-307.1**<br>(111.856)		414.2**<br>(136.699)		-482.3***<br>(132.748)		-65.90<br>(159.354)		-46.31<br>(152.256)		-867.9***<br>(138.327)
BL pertussis (whooping cough) prevalence (% of population, under 5)		-2571.3***<br>(113.999)		-2560.4***<br>(144.504)		-2067.7***<br>(125.198)		-2787.8***<br>(136.462)		-2246.2***<br>(150.496)		-1905.3***<br>(121.393)
BL life expectancy at birth		0.628***<br>(0.067)		0.322***<br>(0.084)		0.440***<br>(0.072)		0.305***<br>(0.071)		0.428***<br>(0.083)		0.218**<br>(0.083)
BL Urban population (%)		-0.0285*<br>(0.012)		-0.0393**<br>(0.013)		-0.00421<br>(0.012)		-0.0364**<br>(0.012)		0.0374**<br>(0.013)		-0.0421***<br>(0.012)
BL School enrollment, secondary (% gross)		0.0778***<br>(0.014)		0.0745***<br>(0.017)		0.0740***<br>(0.015)		0.0447**<br>(0.016)		0.0628***<br>(0.016)		0.0349*<br>(0.016)
		-0.667*		-1.475***		-1.563***		-2.493***		-1.054**		-3.114***

BL Fertility rate (births per woman)		(0.285)		(0.385)		(0.291)		(0.355)		(0.368)		(0.308)
Recipient of US HIV aid before 2004 (=1 if yes)		-0.577<br>(0.488)		0.226<br>(0.741)		-1.001<br>(0.599)		-0.799<br>(0.891)		-2.251**<br>(0.818)		-1.366<br>(0.746)
Constant	86.03***<br>(0.516)	55.99***<br>(5.247)	86.03***<br>(0.516)	82.86***<br>(6.490)	86.03***<br>(0.516)	67.69***<br>(5.670)	86.03***<br>(0.516)	90.30***<br>(5.548)	86.03***<br>(0.516)	67.01***<br>(6.235)	86.03***<br>(0.516)	92.32***<br>(6.207)
Adjusted R-squared	0.144	0.656	0.181	0.669	0.121	0.661	0.163	0.696	0.096	0.564	0.136	0.717
Observations	4282	3926	2628	2328	3386	3087	2592	2263	2565	2322	2589	2319

Notes: Standard errors in parentheses *** p<0.001, ** p<0.01, * p<0.05 BL=baseline; COP=country operational plans; PEPFAR= President's Emergency Plan for AIDS Relief

Table S3-2. Full model results for the indicator "Percentage of children age 12-23 months who received hepatitis B vaccines, 3 doses) "

Variables	All PEPFAR countries		COP countries		Non-COP PEPFAR countries		High-intensity PEPFAR countries		Medium-intensity PEPFAR countries		Low-intensity PEPFAR countries	
	Model 1	Model 2	Model 1	Model 2	Model 1	Model 2	Model 1	Model 2	Model 1	Model 2	Model 1	Model 2
Time variable (=1 post-2004)	11.54***	11.60***	11.54***	11.55***	11.54***	11.60***	11.54***	11.37***	11.54***	11.55***	11.54***	11.29***
	(1.288)	(1.316)	(1.289)	(1.321)	(1.289)	(1.299)	(1.289)	(1.305)	(1.289)	(1.286)	(1.289)	(1.279)
Intervention (=1 if PEPFAR)	-8.247***	-5.161*	-18.39***	-20.57***	-2.948	-1.352	-9.384**	-4.230	-9.150**	-6.953*	-6.256	-2.329
	(2.114)	(2.171)	(2.933)	(3.351)	(2.449)	(2.452)	(3.124)	(3.406)	(3.135)	(3.278)	(3.243)	(3.163)
Interaction term (PEPFAR impact estimate)	1.374	6.667**	8.027**	13.64***	-2.097	3.638	2.661	8.922**	2.163	5.446	-0.648	7.410*
	(2.203)	(2.145)	(3.079)	(2.967)	(2.542)	(2.490)	(3.228)	(3.105)	(3.253)	(3.210)	(3.396)	(3.176)
Country income level (=1 if middle income)		-4.026***		-6.567***		-4.236**		-5.682***		-5.353**		-4.018*
		(1.189)		(1.752)		(1.400)		(1.677)		(1.692)		(2.040)
BL Population		-1.21e-08**		-3.39e-08***		-2.53e-09		-0.000000104***		-8.85e-08***		-1.60e-08***
		(0.000)		(0.000)		(0.000)		(0.000)		(0.000)		(0.000)
BL other donor health spending per capita		0.0161		0.0100		0.0162		0.00816		0.00729		0.0413**
		(0.013)		(0.014)		(0.013)		(0.014)		(0.014)		(0.014)
BL domestic health spending per capita		-0.00691*		-0.00178		-0.00983***		-0.00499		-0.00964***		-0.00944**
		(0.003)		(0.003)		(0.003)		(0.003)		(0.003)		(0.003)
BL GDP per capita		0.000364***		0.000312*		0.000397***		0.000329**		0.000379***		0.000490***
		(0.000)		(0.000)		(0.000)		(0.000)		(0.000)		(0.000)
BL HIV prevalence (% of population ages 15-49)		0.726***		0.795***		-0.0293		0.193		-0.223		-0.994
		(0.116)		(0.147)		(0.666)		(0.128)		(1.234)		(0.916)
BL hepatitis B prevalence (% of population, under 5)		-73.76***		-88.09***		-59.27***		-85.64***		-79.29***		-1.979
		(6.893)		(11.420)		(9.726)		(9.377)		(11.973)		(14.980)
BL life expectancy at birth		0.544***		0.385*		0.297*		0.244		0.452**		0.626***
		(0.111)		(0.164)		(0.120)		(0.145)		(0.160)		(0.152)
BL Urban population (%)		-0.0208		-0.111***		0.00257		-0.0877***		0.0198		-0.0551*
		(0.021)		(0.024)		(0.023)		(0.024)		(0.027)		(0.025)
BL School enrollment, secondary (% gross)		0.0694**		0.0793*		0.0801**		0.0632		0.111***		0.130***
		(0.025)		(0.035)		(0.026)		(0.033)		(0.031)		(0.030)
BL Fertility rate (births per woman)		-0.266		-0.843		-2.202***		-2.715***		-0.834		-4.052***
		(0.482)		(0.641)		(0.505)		(0.641)		(0.623)		(0.629)
Recipient of US HIV aid before 2004 (=1 if yes)		-1.066		6.564***		-1.702		2.547		1.188		-3.207
		(0.846)		(1.451)		(1.054)		(1.457)		(1.524)		(1.733)
Constant	78.67***	41.62***	78.67***	59.37***	78.67***	62.93***	78.67***	76.13***	78.67***	47.78***	78.67***	40.84***

	(1.219)	(8.855)	(1.220)	(12.966)	(1.219)	(9.572)	(1.220)	(11.518)	(1.220)	(12.488)	(1.220)	(11.480)
Adjusted R-squared	0.093	0.288	0.158	0.351	0.071	0.278	0.104	0.343	0.104	0.264	0.085	0.325
Observations	2799	2579	1789	1598	2270	2065	1744	1541	1809	1633	1766	1573

Notes: Standard errors in parentheses *** p<0.001, ** p<0.01, * p<0.05 BL=baseline; COP=country operational plans; PEPFAR= President's Emergency Plan for AIDS Relief

Table S3-3. Full model results for the indicator "Percent of children ages 12-23 months who received the measles vaccination"

Variables	All PEPFAR countries		COP countries		Non-COP PEPFAR countries		High-intensity PEPFAR countries		Medium-intensity PEPFAR countries		Low-intensity PEPFAR countries	
	Model 1	Model 2	Model 1	Model 2	Model 1	Model 2	Model 1	Model 2	Model 1	Model 2	Model 1	Model 2
Time variable (=1 post-2004)	4.348*** (0.679)	3.968*** (0.461)	4.348*** (0.679)	3.964*** (0.468)	4.348*** (0.679)	3.954*** (0.450)	4.348*** (0.679)	3.958*** (0.474)	4.348*** (0.679)	3.962*** (0.453)	4.348*** (0.679)	3.928*** (0.450)
Intervention (=1 if PEPFAR)	-14.16*** (0.794)	-6.963*** (0.672)	-16.87*** (1.061)	-11.20*** (1.267)	-12.64*** (0.928)	-5.398*** (0.740)	-15.92*** (1.057)	-10.19*** (1.301)	-11.49*** (1.120)	-5.582*** (1.022)	-14.91*** (1.265)	-5.543*** (0.874)
Interaction term (PEPFAR impact estimate)	6.644*** (0.995)	6.946*** (0.694)	7.192*** (1.321)	7.462*** (0.986)	6.260*** (1.153)	6.560*** (0.768)	7.034*** (1.330)	6.973*** (0.954)	4.287** (1.399)	5.061*** (1.053)	8.439*** (1.540)	8.529*** (0.905)
Country income level (=1 if middle income)		-5.948*** (0.657)		-7.716*** (0.862)		-4.429*** (0.827)		-7.699*** (0.916)		-6.306*** (1.022)		-4.098*** (0.982)
BL Population		-4.14e-09*** (0.000)		-9.31e-09*** (0.000)		-1.69e-09 (0.000)		-7.29e-08*** (0.000)		-1.66e-08 (0.000)		-6.02e-09*** (0.000)
BL other donor health spending per capita		0.0585*** (0.009)		0.0499*** (0.010)		0.0402*** (0.009)		0.0358*** (0.010)		0.0364*** (0.009)		0.0469*** (0.009)
BL domestic health spending per capita		-0.00638*** (0.001)		-0.00652*** (0.001)		-0.00475*** (0.001)		-0.00494*** (0.001)		-0.00383** (0.001)		-0.00556*** (0.001)
BL GDP per capita		0.000253*** (0.000)		0.000292*** (0.000)		0.000185*** (0.000)		0.000224*** (0.000)		0.000163** (0.000)		0.000280*** (0.000)
BL HIV prevalence (% of population ages 15-49)		1.208*** (0.067)		1.162*** (0.076)		-0.231 (0.310)		1.047*** (0.077)		0.137 (0.554)		0.0681 (0.385)
BL measles prevalence (% of population, under 5)		-17.05*** (1.365)		-21.21*** (1.669)		-5.349*** (1.579)		-20.82*** (1.694)		-0.382 (1.815)		-10.86*** (1.805)
BL life expectancy at birth		0.826*** (0.063)		0.714*** (0.075)		0.624*** (0.072)		0.694*** (0.074)		0.706*** (0.087)		0.650*** (0.081)
BL Urban population (%)		-0.0128 (0.013)		-0.0555*** (0.013)		0.0322* (0.013)		-0.0425*** (0.013)		0.0319* (0.013)		-0.0279* (0.013)
BL School enrollment, secondary (% gross)		0.0834*** (0.014)		0.0567*** (0.017)		0.128*** (0.014)		0.0774*** (0.016)		0.120*** (0.017)		0.126*** (0.015)
BL Fertility rate (births per woman)		-2.887*** (0.281)		-3.192*** (0.348)		-3.468*** (0.299)		-2.524*** (0.351)		-3.561*** (0.385)		-3.976*** (0.289)
Recipient of US HIV aid before 2004 (=1 if yes)		2.089*** (0.528)		3.191*** (0.839)		2.937*** (0.598)		4.077*** (0.907)		2.064* (0.902)		2.265** (0.770)
Constant	85.59*** (0.529)	36.74*** (5.004)	85.59*** (0.529)	51.92*** (5.769)	85.59*** (0.529)	44.10*** (5.710)	85.59*** (0.529)	50.15*** (5.719)	85.59*** (0.529)	40.33*** (6.769)	85.59*** (0.529)	46.69*** (6.147)
Adjusted R-squared	0.147	0.578	0.192	0.572	0.123	0.602	0.175	0.571	0.106	0.504	0.135	0.675

Observations	4275	3926	2621	2328	3379	3087	2585	2263	2558	2322	2582	2319

Notes: Standard errors in parentheses *** p<0.001, ** p<0.01, * p<0.05 BL=baseline; COP=country operational plans; PEPFAR= President's Emergency Plan for AIDS Relief

Table S3-4. Full model results for the indicator "Percentage of births by women of child-bearing age who are immunized against tetanus"

Variables	All PEPFAR countries		COP countries		Non-COP PEPFAR countries		High-intensity PEPFAR countries		Medium-intensity PEPFAR countries		Low-intensity PEPFAR countries	
	Model 1	Model 2	Model 1	Model 2	Model 1	Model 2	Model 1	Model 2	Model 1	Model 2	Model 1	Model 2
Time variable (=1 post-2004)	14.23*** (1.201)	14.93*** (1.144)	14.23*** (1.202)	15.09*** (1.128)	14.23*** (1.201)	14.83*** (1.120)	14.23*** (1.202)	14.92*** (1.139)	14.23*** (1.202)	14.51*** (1.134)	14.23*** (1.202)	15.06*** (1.098)
Intervention (=1 if PEPFAR)	-6.327*** (1.211)	0.630 (1.316)	-5.814*** (1.405)	-3.080 (1.840)	-6.706*** (1.341)	1.822 (1.435)	-6.555*** (1.399)	-3.278 (2.029)	-4.992** (1.621)	0.458 (1.777)	-7.175*** (1.576)	3.863** (1.448)
Interaction term (PEPFAR impact estimate)	6.069*** (1.399)	5.240*** (1.330)	4.775** (1.603)	3.736* (1.526)	6.995*** (1.543)	6.393*** (1.431)	6.154*** (1.600)	5.271*** (1.548)	5.845** (1.838)	5.510** (1.763)	6.059*** (1.807)	5.229*** (1.490)
Country income level (=1 if middle income)		-0.0482 (0.981)		2.648* (1.229)		0.0849 (1.450)		1.399 (1.367)		-0.717 (1.938)		-0.317 (1.683)
BL Population		7.33e-09*** (0.000)		4.18e-09* (0.000)		3.24e-09 (0.000)		-9.99e-08*** (0.000)		-3.03e-08 (0.000)		6.74e-10 (0.000)
BL other donor health spending per capita		0.481*** (0.046)		0.184** (0.056)		0.478*** (0.051)		0.138* (0.061)		0.408*** (0.064)		0.332*** (0.049)
BL domestic health spending per capita		0.00673* (0.003)		-0.00432 (0.005)		0.00927** (0.003)		0.00477 (0.005)		0.0163*** (0.005)		0.0108*** (0.003)
BL GDP per capita		0.000399*** (0.000)		0.000653*** (0.000)		0.000427*** (0.000)		0.000430*** (0.000)		0.0000233 (0.000)		0.000584*** (0.000)
BL HIV prevalence (% of population ages 15-49)		0.0139 (0.104)		0.351** (0.131)		-1.602** (0.515)		0.116 (0.135)		0.0926 (0.855)		-1.498* (0.604)
BL life expectancy at birth		0.0449 (0.099)		0.325* (0.146)		-0.481*** (0.137)		0.236 (0.138)		-0.543*** (0.163)		-0.434* (0.183)
BL Urban population (%)		-0.162*** (0.023)		-0.267*** (0.030)		-0.163*** (0.028)		-0.258*** (0.028)		-0.116*** (0.032)		-0.315*** (0.031)
BL School enrollment, secondary (% gross)		0.00306 (0.024)		-0.0430 (0.035)		0.0214 (0.029)		-0.0244 (0.039)		0.198*** (0.038)		-0.0307 (0.033)
BL Fertility rate (births per woman)		-3.190*** (0.475)		-2.920*** (0.649)		-5.454*** (0.619)		-2.315** (0.824)		-3.763*** (0.678)		-8.121*** (0.619)
Recipient of US HIV aid before 2004 (=1 if yes)		0.828 (0.663)		6.109*** (1.090)		-0.649 (0.832)		6.284*** (1.092)		1.447 (1.288)		-1.469 (1.127)
Constant	68.91*** (1.027)	73.74*** (7.814)	68.91*** (1.027)	61.94*** (10.981)	68.91*** (1.027)	115.9*** (10.941)	68.91*** (1.027)	67.17*** (11.383)	68.91*** (1.027)	103.5*** (12.419)	68.91*** (1.027)	131.1*** (13.269)
Adjusted R-squared	0.264	0.381	0.233	0.361	0.257	0.409	0.252	0.378	0.220	0.331	0.226	0.504
Observations	2830	2657	1624	1480	1964	1848	1614	1441	1336	1249	1396	1309

Notes: Standard errors in parentheses *** p<0.001, ** p<0.01, * p<0.05 BL=baseline; COP=country operational plans; PEPFAR= President's Emergency Plan for AIDS Relief

Table S3-5. Full model results for the indicator "Number of women who die from pregnancy-related causes while pregnant or within 42 days of pregnancy termination per 100,000 live births"

Variables	All PEPFAR countries		COP countries		Non-COP PEPFAR countries		High-intensity PEPFAR countries		Medium-intensity PEPFAR countries		Low-intensity PEPFAR countries	
	Model 1	Model 2	Model 1	Model 2	Model 1	Model 2	Model 1	Model 2	Model 1	Model 2	Model 1	Model 2
Time variable (=1 post-2004)	-23.62* (12.025)	-22.22*** (6.590)	-23.62* (12.031)	-22.22*** (4.599)	-23.62* (12.027)	-22.22** (6.926)	-23.62* (12.031)	-22.22*** (4.952)	-23.62* (12.031)	-22.22** (6.818)	-23.62* (12.031)	-22.22*** (5.261)
Intervention (=1 if PEPFAR)	344.3*** (24.509)	119.3*** (13.902)	434.5*** (28.938)	173.1*** (22.518)	296.8*** (31.804)	96.95*** (16.785)	457.8*** (29.865)	312.0*** (21.591)	287.8*** (44.605)	96.56*** (25.704)	287.2*** (40.462)	127.4*** (20.937)
Interaction term (PEPFAR impact estimate)	-96.46*** (26.427)	-100.7*** (14.549)	-120.9*** (31.291)	-130.9*** (18.636)	-83.62* (34.231)	-85.40*** (18.448)	-125.8*** (32.313)	-135.7*** (17.343)	-98.65* (47.410)	-100.0*** (27.738)	-64.96 (44.089)	-68.94** (22.129)
Country income level (=1 if middle income)		-9.210 (12.949)		-54.34*** (12.326)		8.032 (14.711)		-52.43*** (12.773)		11.30 (17.366)		-15.88 (14.605)
BL Population		1.61e-08 (0.000)		-5.30e-09 (0.000)		2.39e-08* (0.000)		0.00000154*** (0.000)		-6.83e-08 (0.000)		-2.78e-08** (0.000)
BL other donor health spending per capita		-1.030*** (0.131)		-0.590*** (0.086)		-0.976*** (0.132)		-0.329*** (0.061)		-0.850*** (0.124)		-0.646*** (0.105)
BL domestic health spending per capita		0.235*** (0.019)		0.126*** (0.017)		0.287*** (0.022)		0.0875*** (0.015)		0.269*** (0.027)		0.195*** (0.021)
BL GDP per capita		-0.00652*** (0.001)		-0.00247*** (0.000)		-0.00829*** (0.001)		-0.00151*** (0.000)		-0.00682*** (0.001)		-0.00604*** (0.001)
BL HIV prevalence (% of population ages 15-49)		-18.21*** (1.719)		-8.354*** (1.404)		3.454 (6.607)		-10.38*** (1.112)		-9.382 (8.656)		25.44*** (7.458)
BL life expectancy at birth		-27.23*** (1.694)		-16.37*** (1.316)		-26.98*** (2.223)		-15.91*** (1.039)		-28.87*** (2.979)		-14.35*** (1.447)
BL Urban population (%)		-0.0420 (0.183)		-0.195 (0.155)		-0.403* (0.191)		-0.468** (0.153)		-0.157 (0.208)		-0.454** (0.162)
BL School enrollment, secondary (% gross)		-1.340*** (0.203)		-0.846*** (0.192)		-1.851*** (0.203)		-1.176*** (0.180)		-1.333*** (0.204)		-2.084*** (0.218)
BL Fertility rate (births per woman)		24.85*** (4.525)		26.97*** (3.681)		25.77*** (4.823)		12.82** (4.175)		22.32*** (4.459)		37.64*** (4.616)
Recipient of US HIV aid before 2004 (=1 if yes)		-66.19*** (8.720)		-27.82* (11.967)		-49.84*** (9.233)		-155.8*** (11.494)		-35.08* (16.111)		-46.21*** (9.503)
Constant	101.8*** (11.036)	2057.0*** (120.547)	101.8*** (11.042)	1287.4*** (88.946)	101.8*** (11.038)	2069.6*** (156.571)	101.8*** (11.042)	1322.0*** (75.173)	101.8*** (11.042)	2149.3*** (207.386)	101.8*** (11.042)	1195.5*** (98.220)
Adjusted R-squared	0.196	0.772	0.449	0.815	0.154	0.780	0.469	0.861	0.161	0.726	0.181	0.789
Observations	2628	2412	1566	1386	2070	1890	1548	1350	1548	1404	1548	1386

Notes: Standard errors in parentheses *** p<0.001, ** p<0.01, * p<0.05 BL=baseline; COP=country operational plans; PEPFAR= President's Emergency Plan for AIDS Relief

Table S3-6. Full model results for the indicator "Probability of a child dying between birth and 5 years of age, per 1,000 live births"

Variables	All PEPFAR countries		COP countries		Non-COP PEPFAR countries		High-intensity PEPFAR countries		Medium-intensity PEPFAR countries		Low-intensity PEPFAR countries	
	Model 1	Model 2	Model 1	Model 2	Model 1	Model 2	Model 1	Model 2	Model 1	Model 2	Model 1	Model 2
Time variable (=1 post-2004)	-14.45***<br>(1.374)	-14.62***<br>(0.795)	-14.45***<br>(1.374)	-14.62***<br>(0.767)	-14.45***<br>(1.374)	-14.62***<br>(0.794)	-14.45***<br>(1.374)	-14.62***<br>(0.772)	-14.45***<br>(1.374)	-14.62***<br>(0.767)	-14.45***<br>(1.374)	-14.62***<br>(0.775)
Intervention (=1 if PEPFAR)	62.90***<br>(2.123)	14.73***<br>(1.291)	82.70***<br>(2.868)	17.64***<br>(2.437)	52.49***<br>(2.549)	11.21***<br>(1.364)	86.70***<br>(3.043)	33.69***<br>(3.286)	49.38***<br>(3.253)	7.373***<br>(1.576)	52.62***<br>(3.323)	12.81***<br>(1.893)
Interaction term (PEPFAR impact estimate)	-26.90***<br>(2.517)	-27.38***<br>(1.285)	-34.29***<br>(3.362)	-35.67***<br>(1.984)	-23.02***<br>(3.021)	-23.17***<br>(1.418)	-37.76***<br>(3.522)	-40.24***<br>(2.209)	-21.98***<br>(3.856)	-21.81***<br>(1.661)	-20.97***<br>(3.981)	-21.18***<br>(1.797)
Country income level (=1 if middle income)		-1.820<br>(1.324)		2.627<br>(2.046)		-4.866**<br>(1.526)		3.619<br>(2.094)		-5.253**<br>(1.638)		-5.711*<br>(2.229)
BL Population		9.74e-09***<br>(0.000)		1.27e-08***<br>(0.000)		1.05e-08***<br>(0.000)		6.95e-08***<br>(0.000)		6.61e-08***<br>(0.000)		6.26e-09***<br>(0.000)
BL other donor health spending per capita		-0.266***<br>(0.019)		-0.210***<br>(0.018)		-0.261***<br>(0.020)		-0.203***<br>(0.019)		-0.239***<br>(0.020)		-0.207***<br>(0.020)
BL domestic health spending per capita		0.0148***<br>(0.002)		0.00285<br>(0.003)		0.0203***<br>(0.002)		-0.00188<br>(0.003)		0.0190***<br>(0.003)		0.0119***<br>(0.003)
BL GDP per capita		-0.000482***<br>(0.000)		0.0000306<br>(0.000)		-0.000612***<br>(0.000)		0.000102<br>(0.000)		-0.000481***<br>(0.000)		-0.000364***<br>(0.000)
BL HIV prevalence (% of population ages 15-49)		-2.428***<br>(0.148)		-1.969***<br>(0.166)		-0.694<br>(0.612)		-2.338***<br>(0.183)		1.199<br>(0.861)		1.000<br>(0.808)
BL life expectancy at birth		-3.943***<br>(0.118)		-3.659***<br>(0.153)		-3.952***<br>(0.146)		-3.748***<br>(0.146)		-4.121***<br>(0.177)		-3.139***<br>(0.164)
BL Urban population (%)		-0.00453<br>(0.023)		0.0526*<br>(0.023)		-0.0839***<br>(0.022)		0.0754**<br>(0.026)		-0.0708**<br>(0.022)		-0.0734***<br>(0.022)
BL School enrollment, secondary (% gross)		-0.206***<br>(0.024)		-0.274***<br>(0.031)		-0.194***<br>(0.025)		-0.259***<br>(0.033)		-0.125***<br>(0.027)		-0.239***<br>(0.027)
BL Fertility rate (births per woman)		7.443***<br>(0.525)		6.159***<br>(0.630)		7.046***<br>(0.569)		5.158***<br>(0.704)		6.509***<br>(0.545)		7.496***<br>(0.683)
Recipient of US HIV aid before 2004 (=1 if yes)		-1.934<br>(1.010)		3.893*<br>(1.634)		-0.604<br>(1.060)		-6.205**<br>(2.401)		1.181<br>(1.355)		0.592<br>(1.491)
Constant	37.56***<br>(1.129)	317.9***<br>(8.954)	37.56***<br>(1.129)	298.1***<br>(12.020)	37.56***<br>(1.129)	323.9***<br>(10.724)	37.56***<br>(1.129)	304.9***<br>(11.757)	37.56***<br>(1.129)	329.0***<br>(12.990)	37.56***<br>(1.129)	269.2***<br>(11.589)
Adjusted R-squared	0.298	0.838	0.486	0.842	0.243	0.843	0.494	0.837	0.240	0.840	0.254	0.839
Observations	4408	4002	2697	2349	3509	3161	2668	2291	2668	2378	2668	2349

Notes: Standard errors in parentheses *** p<0.001, ** p<0.01, * p<0.05 BL=baseline; COP=country operational plans; PEPFAR= President's Emergency Plan for AIDS Relief

Table S3-7. Full model results for the indicator "Prevalence of anemia among women of reproductive age (% of women ages 15-49)"

Variables	All PEPFAR countries		COP countries		Non-COP PEPFAR countries		High-intensity PEPFAR countries		Medium-intensity PEPFAR countries		Low-intensity PEPFAR countries	
	**Model 1**	**Model 2**	**Model 1**	**Model 2**	**Model 1**	**Model 2**	**Model 1**	**Model 2**	**Model 1**	**Model 2**	**Model 1**	**Model 2**
Time variable (=1 post-2004)	-5.415*** (0.478)	-5.587*** (0.413)	-5.415*** (0.479)	-5.587*** (0.390)	-5.415*** (0.478)	-5.587*** (0.395)	-5.415*** (0.479)	-5.587*** (0.395)	-5.415*** (0.479)	-5.587*** (0.380)	-5.415*** (0.479)	-5.587*** (0.390)
Intervention (=1 if PEPFAR)	8.469*** (0.501)	-0.967* (0.480)	10.39*** (0.588)	-2.582** (0.889)	7.461*** (0.591)	-2.707*** (0.488)	10.16*** (0.610)	-1.116 (1.017)	7.960*** (0.773)	-2.932*** (0.578)	7.287*** (0.710)	-2.899*** (0.692)
Interaction term (PEPFAR impact estimate)	-1.256 (0.688)	-1.023 (0.536)	-1.908* (0.839)	-1.581* (0.739)	-0.914 (0.813)	-0.740 (0.551)	-2.389** (0.875)	-2.184** (0.800)	-0.897 (1.063)	-0.724 (0.657)	-0.482 (0.989)	-0.252 (0.632)
Country income level (=1 if middle income)		-5.738*** (0.562)		-0.478 (0.757)		-7.546*** (0.705)		-1.090 (0.819)		-4.577*** (0.766)		-9.547*** (1.025)
BL Population		3.62e-09** (0.000)		1.21e-08*** (0.000)		-2.66e-10 (0.000)		-1.16e-08 (0.000)		3.43e-08*** (0.000)		2.27e-09* (0.000)
BL other donor health spending per capita		-0.0717*** (0.003)		-0.0633*** (0.004)		-0.0685*** (0.004)		-0.0628*** (0.004)		-0.0797*** (0.004)		-0.0605*** (0.005)
BL domestic health spending per capita		-0.00834*** (0.001)		-0.00623*** (0.001)		-0.00420*** (0.001)		-0.00714*** (0.001)		0.000529 (0.001)		-0.00470*** (0.001)
BL GDP per capita		0.000395*** (0.000)		0.000380*** (0.000)		0.000315*** (0.000)		0.000416*** (0.000)		0.000224*** (0.000)		0.000315*** (0.000)
BL HIV prevalence (% of population ages 15-49)		-0.674*** (0.043)		-0.571*** (0.052)		0.782*** (0.197)		-0.690*** (0.063)		0.766 (0.393)		0.628** (0.220)
BL life expectancy at birth		-0.584*** (0.036)		-0.474*** (0.049)		-0.452*** (0.041)		-0.541*** (0.053)		-0.597*** (0.048)		-0.247*** (0.054)
BL Urban population (%)		0.0352** (0.012)		-0.0543*** (0.013)		-0.0240* (0.011)		-0.0362** (0.014)		-0.0739*** (0.012)		-0.0698*** (0.013)
BL School enrollment, secondary (% gross)		-0.0529*** (0.011)		-0.128*** (0.014)		-0.0689*** (0.011)		-0.130*** (0.015)		-0.114*** (0.012)		-0.0621*** (0.013)
BL Fertility rate (births per woman)		1.243*** (0.195)		0.329 (0.308)		1.543*** (0.210)		-0.0342 (0.367)		1.816*** (0.229)		1.459*** (0.257)
Recipient of US HIV aid before 2004 (=1 if yes)		-0.777* (0.335)		2.547*** (0.534)		0.361 (0.387)		2.561*** (0.638)		-1.045 (0.534)		1.752** (0.584)
Constant	32.99*** (0.359)	79.22*** (3.029)	32.99*** (0.359)	78.72*** (4.425)	32.99*** (0.359)	73.80*** (3.318)	32.99*** (0.359)	84.49*** (4.858)	32.99*** (0.359)	85.47*** (3.923)	32.99*** (0.359)	63.42*** (4.450)
Adjusted R-squared	0.162	0.590	0.241	0.526	0.137	0.659	0.226	0.505	0.146	0.654	0.144	0.636
Observations	3996	3672	2403	2133	3159	2889	2376	2079	2376	2160	2376	2133

Notes: Standard errors in parentheses ** p<0.001, ** p<0.01, * p<0.05 BL=baseline; COP=country operational plans; PEPFAR= President's Emergency Plan for AIDS Relief

S4. Full model of logged regression of seven outcomes from traditional DID

Table S4-1. Full model results for the indicator "Percent of children ages 12-23 months who received DPT vaccinations (3 doses)"

Variables	All PEPFAR countries		COP countries		Non-COP PEPFAR countries		High-intensity PEPFAR countries		Medium-intensity PEPFAR countries		Low-intensity PEPFAR countries	
	Model 1	Model 2	Model 1	Model 2	Model 1	Model 2	Model 1	Model 2	Model 1	Model 2	Model 1	Model 2
Time variable (=1 post-2004)	0.0457*** (0.011)	0.0388*** (0.010)	0.0457*** (0.011)	0.0382*** (0.010)	0.0457*** (0.011)	0.0387*** (0.008)	0.0457*** (0.011)	0.0382*** (0.009)	0.0457*** (0.011)	0.0385*** (0.008)	0.0457*** (0.011)	0.0383*** (0.008)
Intervention (=1 if PEPFAR)	-0.237*** (0.014)	-0.120*** (0.012)	-0.277*** (0.021)	-0.119*** (0.024)	-0.214*** (0.017)	-0.106*** (0.013)	-0.260*** (0.021)	-0.0709** (0.025)	-0.173*** (0.020)	-0.0529*** (0.015)	-0.273*** (0.025)	-0.153*** (0.018)
Interaction term (PEPFAR impact estimate)	0.157*** (0.018)	0.171*** (0.014)	0.160*** (0.026)	0.186*** (0.020)	0.154*** (0.020)	0.165*** (0.015)	0.172*** (0.025)	0.159*** (0.020)	0.102*** (0.023)	0.143*** (0.018)	0.193*** (0.028)	0.214*** (0.020)
Country income level (=1 if middle income)		-0.0674*** (0.015)		-0.0864*** (0.020)		-0.0727*** (0.014)		-0.0877*** (0.022)		-0.0884*** (0.016)		-0.100*** (0.018)
Ln(BL Population)		-0.00746*** (0.002)		-0.00232 (0.003)		-0.0165*** (0.002)		0.000465 (0.003)		-0.00577* (0.002)		-0.0157*** (0.002)
Ln(BL other donor health spending per capita)		0.0172*** (0.004)		0.00806 (0.004)		0.00300 (0.003)		0.00551 (0.004)		0.00967** (0.004)		0.00221 (0.003)
Ln(BL domestic health spending per capita)		-0.0194 (0.012)		0.00207 (0.014)		-0.0537*** (0.013)		-0.0283* (0.013)		-0.000728 (0.013)		-0.0624*** (0.015)
Ln(BL GDP per capita)		-0.0143 (0.016)		-0.0582** (0.022)		-0.0243 (0.017)		-0.0500* (0.020)		-0.0306 (0.017)		-0.0226 (0.021)
Ln(BL HIV prevalence (% of population ages 15-49))		-0.000723 (0.001)		-0.00307 (0.002)		-0.00518*** (0.001)		-0.00490 (0.003)		-0.00458** (0.001)		-0.00301 (0.002)
Ln(BL diphtheria prevalence (% of population, under 5))		-0.0302*** (0.002)		-0.0331*** (0.003)		-0.0104*** (0.002)		-0.0298*** (0.003)		-0.00679** (0.002)		-0.00376 (0.002)
Ln(BL tetanus prevalence (% of population, under 5))		0.0153*** (0.002)		0.0110*** (0.002)		0.0132*** (0.002)		0.00464 (0.002)		0.0138*** (0.003)		0.0105*** (0.002)
Ln(BL pertussis (whooping cough) prevalence (% of population, under 5))		-0.143*** (0.007)		-0.132*** (0.010)		-0.0992*** (0.006)		-0.137*** (0.010)		-0.106*** (0.007)		-0.0779*** (0.006)
Ln(BL life expectancy at birth)		-0.00507 (0.045)		-0.200*** (0.050)		0.770*** (0.088)		-0.110* (0.050)		0.764*** (0.097)		0.958*** (0.110)
Ln(BL Urban population (%))		0.00682 (0.012)		-0.00816 (0.015)		0.130*** (0.015)		0.00458 (0.015)		0.0841*** (0.012)		0.109*** (0.017)
Ln(BL School enrollment, secondary (%		0.128*** (0.017)		0.0650** (0.023)		0.140*** (0.019)		0.0787*** (0.022)		0.114*** (0.023)		0.159*** (0.021)

gross))												
Ln(BL Fertility rate (births per woman))		-0.173***<br>(0.016)		-0.255***<br>(0.022)		-0.112***<br>(0.015)		-0.229***<br>(0.022)		-0.0900***<br>(0.017)		-0.151***<br>(0.017)
Recipient of US HIV aid before 2004 (=1 if yes)		0.0298**<br>(0.011)		-0.0474**<br>(0.014)		0.0464***<br>(0.010)		-0.0452*<br>(0.018)		-0.0208<br>(0.013)		0.0339**<br>(0.012)
Constant	4.433***<br>(0.008)	3.430***<br>(0.201)	4.433***<br>(0.008)	4.865***<br>(0.232)	4.433***<br>(0.008)	0.429<br>(0.336)	4.433***<br>(0.008)	4.357***<br>(0.247)	4.433***<br>(0.008)	0.344<br>(0.421)	4.433***<br>(0.008)	-0.102<br>(0.416)
Adjusted R-squared	0.120	0.511	0.142	0.502	0.106	0.569	0.130	0.492	0.074	0.492	0.123	0.618
Observations	4282	3540	2628	2024	3386	2728	2592	1986	2565	1992	2589	1986

Notes: Standard errors in parentheses *** p<0.001, ** p<0.01, * p<0.05 BL=baseline; COP=country operational plans; PEPFAR= President's Emergency Plan for AIDS Relief

Table S4-2. Full model results for the indicator "Percentage of children age 12-23 months who received hepatitis B vaccines, 3 doses) "

Variables	All PEPFAR countries		COP countries		Non-COP PEPFAR countries		High-intensity PEPFAR countries		Medium-intensity PEPFAR countries		Low-intensity PEPFAR countries	
	Model 1	Model 2	Model 1	Model 2	Model 1	Model 2	Model 1	Model 2	Model 1	Model 2	Model 1	Model 2
Time variable (=1 post-2004)	0.240*** (0.034)	0.212*** (0.032)	0.240*** (0.034)	0.201*** (0.032)	0.240*** (0.034)	0.212*** (0.032)	0.240*** (0.034)	0.200*** (0.032)	0.240*** (0.034)	0.212*** (0.032)	0.240*** (0.034)	0.199*** (0.031)
Intervention (=1 if PEPFAR)	-0.184** (0.060)	-0.190** (0.060)	-0.343*** (0.090)	-0.348*** (0.087)	-0.101 (0.070)	-0.158* (0.072)	-0.149 (0.094)	-0.0337 (0.084)	-0.253** (0.095)	-0.230* (0.093)	-0.123 (0.082)	-0.115 (0.088)
Interaction term (PEPFAR impact estimate)	0.0894 (0.061)	0.185** (0.060)	0.194* (0.091)	0.262** (0.086)	0.0350 (0.071)	0.172* (0.072)	0.0669 (0.095)	0.148 (0.090)	0.162 (0.096)	0.220* (0.092)	0.0121 (0.084)	0.194* (0.088)
Country income level (=1 if middle income)		0.0346 (0.029)		0.0849* (0.041)		-0.0451 (0.031)		0.0127 (0.036)		-0.0288 (0.038)		0.0358 (0.052)
Ln(BL Population)		-0.0174* (0.008)		-0.0265** (0.010)		-0.0103 (0.008)		-0.00208 (0.009)		-0.0105 (0.009)		-0.0194* (0.009)
Ln(BL other donor health spending per capita)		0.0168 (0.009)		-0.00133 (0.011)		0.0171 (0.010)		0.0137 (0.012)		0.0109 (0.010)		0.0109 (0.011)
Ln(BL domestic health spending per capita)		-0.0654** (0.023)		-0.0549 (0.030)		-0.102*** (0.029)		-0.0955** (0.029)		-0.140*** (0.032)		-0.0888* (0.036)
Ln(BL GDP per capita)		0.0164 (0.033)		-0.0127 (0.054)		0.0474 (0.035)		0.0470 (0.051)		0.0532 (0.041)		0.0240 (0.046)
Ln(BL HIV prevalence (% of population ages 15-49))		0.0130*** (0.004)		0.0252*** (0.006)		0.00269 (0.003)		0.00918 (0.005)		0.00156 (0.004)		0.0111* (0.005)
Ln(BL hepatitis B prevalence (% of population, under 5))		-0.0742*** (0.009)		-0.0683*** (0.013)		-0.0561*** (0.010)		-0.0618*** (0.012)		-0.0641*** (0.013)		-0.0455*** (0.013)
Ln(BL life expectancy at birth)		0.109 (0.111)		0.0343 (0.158)		0.601*** (0.168)		0.247 (0.134)		0.410 (0.217)		0.947*** (0.209)
Ln(BL Urban population (%))		0.0303 (0.025)		-0.0338 (0.028)		0.0879* (0.036)		-0.0553* (0.028)		0.125*** (0.035)		0.0304 (0.041)
Ln(BL School enrollment, secondary (% gross))		0.0153 (0.029)		0.0250 (0.041)		0.0291 (0.033)		0.0337 (0.038)		0.109* (0.047)		0.0543 (0.041)
Ln(BL Fertility rate (births per woman))		-0.136*** (0.036)		-0.184*** (0.052)		-0.118** (0.038)		-0.231*** (0.054)		-0.121* (0.052)		-0.144*** (0.041)
Recipient of US HIV aid before 2004 (=1 if yes)		0.00105 (0.022)		0.0784* (0.038)		-0.0232 (0.026)		-0.0458 (0.036)		0.0247 (0.037)		-0.110* (0.048)
Constant	4.247*** (0.034)	3.956*** (0.542)	4.247*** (0.034)	4.901*** (0.783)	4.247*** (0.034)	1.496* (0.729)	4.247*** (0.034)	3.459*** (0.736)	4.247*** (0.034)	1.948 (1.011)	4.247*** (0.034)	0.452 (0.855)

Adjusted R-squared	0.081	0.184	0.106	0.217	0.067	0.179	0.076	0.211	0.090	0.194	0.067	0.215
Observations	2799	2304	1789	1379	2270	1809	1744	1341	1809	1399	1766	1332

Notes: Standard errors in parentheses *** p<0.001, ** p<0.01, * p<0.05 BL=baseline; COP=country operational plans; PEPFAR= President's Emergency Plan for AIDS Relief

Table S4-3. Full model results for the indicator "Percent of children ages 12-23 months who received the measles vaccination"

Variables	All PEPFAR countries		COP countries		Non-COP PEPFAR countries		High-intensity PEPFAR countries		Medium-intensity PEPFAR countries		Low-intensity PEPFAR countries	
	Model 1	Model 2	Model 1	Model 2	Model 1	Model 2	Model 1	Model 2	Model 1	Model 2	Model 1	Model 2
Time variable (=1 post-2004)	0.0577***<br>(0.010)	0.0546***<br>(0.008)	0.0577***<br>(0.010)	0.0545***<br>(0.009)	0.0577***<br>(0.010)	0.0551***<br>(0.008)	0.0577***<br>(0.010)	0.0546***<br>(0.008)	0.0577***<br>(0.010)	0.0550***<br>(0.008)	0.0577***<br>(0.010)	0.0552***<br>(0.008)
Intervention (=1 if PEPFAR)	-0.213***<br>(0.013)	-0.136***<br>(0.013)	-0.248***<br>(0.019)	-0.178***<br>(0.024)	-0.193***<br>(0.015)	-0.105***<br>(0.013)	-0.228***<br>(0.018)	-0.115***<br>(0.023)	-0.167***<br>(0.019)	-0.0911***<br>(0.017)	-0.241***<br>(0.022)	-0.116***<br>(0.016)
Interaction term (PEPFAR impact estimate)	0.118***<br>(0.016)	0.124***<br>(0.013)	0.129***<br>(0.022)	0.143***<br>(0.020)	0.110***<br>(0.018)	0.115***<br>(0.014)	0.118***<br>(0.022)	0.105***<br>(0.019)	0.0769***<br>(0.022)	0.100***<br>(0.018)	0.155***<br>(0.026)	0.168***<br>(0.018)
Country income level (=1 if middle income)		-0.0269*<br>(0.014)		-0.0130<br>(0.017)		-0.0541***<br>(0.014)		-0.00223<br>(0.018)		-0.0879***<br>(0.017)		-0.0434**<br>(0.017)
Ln(BL Population)		-0.00658**<br>(0.002)		-0.0130***<br>(0.003)		-0.00953***<br>(0.002)		-0.00973**<br>(0.003)		-0.00599*<br>(0.003)		-0.0134***<br>(0.002)
Ln(BL other donor health spending per capita)		0.0161***<br>(0.003)		0.00291<br>(0.004)		0.00384<br>(0.003)		0.00268<br>(0.004)		0.00734*<br>(0.003)		0.00190<br>(0.003)
Ln(BL domestic health spending per capita)		0.0176<br>(0.012)		0.0314*<br>(0.014)		-0.0165<br>(0.013)		0.00623<br>(0.014)		0.0140<br>(0.014)		-0.0243<br>(0.014)
Ln(BL GDP per capita)		-0.0122<br>(0.013)		-0.0256<br>(0.019)		-0.0198<br>(0.014)		-0.0247<br>(0.016)		-0.00894<br>(0.014)		-0.00176<br>(0.016)
Ln(BL HIV prevalence (% of population ages 15-49))		0.0113***<br>(0.001)		0.0146***<br>(0.002)		0.00308**<br>(0.001)		0.0126***<br>(0.002)		0.00435***<br>(0.001)		0.00502***<br>(0.001)
Ln(BL measles prevalence (% of population, under 5))		-0.00127<br>(0.001)		-0.0000882<br>(0.001)		0.00105<br>(0.001)		-0.00144<br>(0.001)		0.00140<br>(0.001)		0.00437***<br>(0.001)
Ln(BL life expectancy at birth)		0.222***<br>(0.047)		0.0349<br>(0.056)		0.825***<br>(0.079)		0.117*<br>(0.050)		0.756***<br>(0.092)		0.963***<br>(0.091)
Ln(BL Urban population (%))		-0.0360**<br>(0.012)		-0.0702***<br>(0.013)		0.0732***<br>(0.013)		-0.0522***<br>(0.011)		0.0364**<br>(0.013)		0.0420**<br>(0.014)
Ln(BL School enrollment, secondary (% gross))		0.117***<br>(0.014)		0.0400*<br>(0.020)		0.136***<br>(0.015)		0.0641***<br>(0.018)		0.124***<br>(0.017)		0.132***<br>(0.017)
Ln(BL Fertility rate (births per woman))		-0.222***<br>(0.017)		-0.287***<br>(0.023)		-0.115***<br>(0.016)		-0.246***<br>(0.023)		-0.107***<br>(0.021)		-0.138***<br>(0.017)
Recipient of US HIV aid before 2004 (=1 if yes)		0.0595***<br>(0.011)		0.0299*<br>(0.015)		0.0604***<br>(0.010)		0.0219<br>(0.015)		0.0258<br>(0.015)		0.0444***<br>(0.011)
Constant	4.427***<br>(0.008)	3.498***<br>(0.201)	4.427***<br>(0.008)	4.964***<br>(0.244)	4.427***<br>(0.008)	0.639*<br>(0.313)	4.427***<br>(0.008)	4.469***<br>(0.223)	4.427***<br>(0.008)	0.830*<br>(0.413)	4.427***<br>(0.008)	0.172<br>(0.356)

Adjusted R-squared	0.129	0.441	0.160	0.394	0.111	0.548	0.145	0.374	0.090	0.435	0.129	0.615
Observations	4275	3540	2621	2024	3379	2728	2585	1986	2558	1992	2582	1986

Notes: Standard errors in parentheses *** p<0.001, ** p<0.01, * p<0.05 BL=baseline; COP=country operational plans; PEPFAR= President's Emergency Plan for AIDS Relief

Table S4-4. Full model results for the indicator "Percentage of births by women of child-bearing age who are immunized against tetanus"

Variables	All PEPFAR countries		COP countries		Non-COP PEPFAR countries		High-intensity PEPFAR countries		Medium-intensity PEPFAR countries		Low-intensity PEPFAR countries	
	Model 1	Model 2	Model 1	Model 2	Model 1	Model 2	Model 1	Model 2	Model 1	Model 2	Model 1	Model 2
Time variable (=1 post-2004)	0.233*** (0.022)	0.243*** (0.023)	0.233*** (0.022)	0.247*** (0.022)	0.233*** (0.022)	0.243*** (0.023)	0.233*** (0.022)	0.245*** (0.023)	0.233*** (0.022)	0.237*** (0.023)	0.233*** (0.022)	0.254*** (0.022)
Intervention (=1 if PEPFAR)	-0.109*** (0.025)	-0.0370 (0.027)	-0.0992*** (0.029)	-0.112** (0.038)	-0.115*** (0.027)	-0.0228 (0.029)	-0.109*** (0.029)	-0.0966* (0.042)	-0.0835** (0.032)	-0.0452 (0.034)	-0.130*** (0.032)	0.0334 (0.031)
Interaction term (PEPFAR impact estimate)	0.109*** (0.026)	0.0978*** (0.026)	0.0918** (0.031)	0.0747* (0.031)	0.122*** (0.029)	0.111*** (0.029)	0.109*** (0.031)	0.0954** (0.032)	0.0979** (0.034)	0.0958** (0.034)	0.119*** (0.035)	0.0973** (0.031)
Country income level (=1 if middle income)		-0.00739 (0.021)		0.00782 (0.026)		-0.0485 (0.030)		0.0394 (0.027)		-0.00567 (0.036)		-0.0985** (0.033)
Ln(BL Population)		-0.0240*** (0.005)		-0.0268*** (0.006)		-0.0245*** (0.006)		-0.0302*** (0.007)		-0.0358*** (0.007)		-0.0181** (0.006)
Ln(BL other donor health spending per capita)		0.00245 (0.006)		-0.00663 (0.008)		0.00546 (0.007)		-0.00149 (0.008)		-0.00557 (0.008)		0.0162* (0.007)
Ln(BL domestic health spending per capita)		-0.0446** (0.016)		-0.0858*** (0.022)		-0.0293 (0.021)		-0.0217 (0.029)		-0.0175 (0.025)		-0.0821*** (0.024)
Ln(BL GDP per capita)		0.0601*** (0.017)		0.134*** (0.025)		0.0598*** (0.018)		0.0645* (0.029)		0.00203 (0.022)		0.196*** (0.023)
Ln(BL HIV prevalence (% of population ages 15-49))		-0.00119 (0.002)		-0.00516 (0.003)		0.000978 (0.003)		0.00249 (0.004)		-0.000466 (0.003)		-0.000968 (0.003)
Ln(BL life expectancy at birth)		-0.0369 (0.073)		-0.154 (0.111)		-0.00327 (0.117)		0.0562 (0.095)		-0.379** (0.130)		0.134 (0.154)
Ln(BL Urban population (%))		-0.0261 (0.017)		-0.115*** (0.022)		-0.00585 (0.025)		-0.107*** (0.020)		0.0509* (0.025)		-0.162*** (0.026)
Ln(BL School enrollment, secondary (% gross))		-0.0258 (0.018)		-0.0871*** (0.026)		-0.00317 (0.026)		-0.0797** (0.027)		-0.00822 (0.042)		-0.0253 (0.032)
Ln(BL Fertility rate (births per woman))		-0.264*** (0.032)		-0.313*** (0.048)		-0.291*** (0.036)		-0.204*** (0.053)		-0.342*** (0.044)		-0.398*** (0.042)
Recipient of US HIV aid before 2004 (=1 if yes)		0.0615*** (0.016)		0.182*** (0.028)		0.0185 (0.017)		0.132*** (0.028)		0.0427 (0.024)		0.0566** (0.022)
Constant	4.174*** (0.021)	4.900*** (0.361)	4.174*** (0.021)	5.621*** (0.545)	4.174*** (0.021)	4.585*** (0.488)	4.174*** (0.021)	4.865*** (0.502)	4.174*** (0.021)	6.625*** (0.574)	4.174*** (0.021)	3.812*** (0.604)
Adjusted R-squared	0.217	0.289	0.187	0.267	0.216	0.317	0.201	0.265	0.184	0.265	0.191	0.389
Observations	2830	2657	1624	1480	1964	1848	1614	1441	1336	1249	1396	1309

Notes: Standard errors in parentheses *** p<0.001, ** p<0.01, * p<0.05 BL=baseline; COP=country operational plans; PEPFAR= President's Emergency Plan for AIDS Relief

Table S4-5. Full model results for the indicator "Number of women who die from pregnancy-related causes while pregnant or within 42 days of pregnancy termination per 100,000 live births"

Variables	All PEPFAR countries		COP countries		Non-COP PEPFAR countries		High-intensity PEPFAR countries		Medium-intensity PEPFAR countries		Low-intensity PEPFAR countries	
	Model 1	Model 2	Model 1	Model 2	Model 1	Model 2	Model 1	Model 2	Model 1	Model 2	Model 1	Model 2
Time variable (=1 post-2004)	-0.289***<br>(0.083)	-0.272***<br>(0.049)	-0.289***<br>(0.083)	-0.272***<br>(0.050)	-0.289***<br>(0.083)	-0.272***<br>(0.047)	-0.289***<br>(0.083)	-0.272***<br>(0.049)	-0.289***<br>(0.083)	-0.272***<br>(0.046)	-0.289***<br>(0.083)	-0.272***<br>(0.046)
Intervention (=1 if PEPFAR)	1.497***<br>(0.098)	0.179**<br>(0.055)	2.037***<br>(0.104)	0.480***<br>(0.083)	1.213***<br>(0.114)	0.0921<br>(0.057)	2.080***<br>(0.104)	0.332***<br>(0.077)	1.195***<br>(0.141)	0.00660<br>(0.062)	1.216***<br>(0.142)	0.146<br>(0.075)
Interaction term (PEPFAR impact estimate)	-0.0337<br>(0.112)	-0.0358<br>(0.058)	-0.0171<br>(0.118)	-0.0504<br>(0.064)	-0.0424<br>(0.130)	-0.0282<br>(0.062)	-0.00368<br>(0.118)	-0.0275<br>(0.062)	-0.0530<br>(0.160)	-0.0612<br>(0.068)	-0.0444<br>(0.163)	-0.0177<br>(0.078)
Country income level (=1 if middle income)		-0.188***<br>(0.040)		-0.174**<br>(0.053)		-0.0875<br>(0.046)		-0.295***<br>(0.048)		-0.0132<br>(0.050)		-0.141*<br>(0.064)
Ln(BL Population)		-0.00714<br>(0.010)		-0.0187<br>(0.012)		-0.0438***<br>(0.010)		-0.0513***<br>(0.012)		-0.0937***<br>(0.010)		-0.0401**<br>(0.013)
Ln(BL other donor health spending per capita)		-0.0201<br>(0.014)		-0.0162<br>(0.017)		-0.0392**<br>(0.015)		-0.0275<br>(0.018)		-0.0891***<br>(0.014)		-0.0351*<br>(0.018)
Ln(BL domestic health spending per capita)		-0.202***<br>(0.029)		-0.148***<br>(0.041)		-0.244***<br>(0.035)		-0.0176<br>(0.046)		-0.307***<br>(0.035)		-0.318***<br>(0.046)
Ln(BL GDP per capita)		0.101***<br>(0.030)		0.0177<br>(0.046)		0.0579<br>(0.032)		-0.0985*<br>(0.045)		0.0429<br>(0.036)		0.0300<br>(0.039)
Ln(BL HIV prevalence (% of population ages 15-49))		0.0384***<br>(0.005)		0.0153*<br>(0.006)		0.0467***<br>(0.004)		0.0331***<br>(0.006)		0.0366***<br>(0.005)		0.0337***<br>(0.006)
Ln(BL life expectancy at birth)		-2.348***<br>(0.105)		-1.986***<br>(0.156)		-2.147***<br>(0.199)		-1.969***<br>(0.126)		-2.663***<br>(0.216)		-0.828**<br>(0.263)
Ln(BL Urban population (%))		0.0605*<br>(0.029)		0.0170<br>(0.035)		0.127**<br>(0.041)		0.0255<br>(0.035)		0.133**<br>(0.042)		0.129*<br>(0.052)
Ln(BL School enrollment, secondary (% gross))		-0.238***<br>(0.044)		-0.141**<br>(0.052)		-0.509***<br>(0.054)		-0.141**<br>(0.051)		-0.981***<br>(0.066)		-0.462***<br>(0.057)
Ln(BL Fertility rate (births per woman))		1.030***<br>(0.055)		0.856***<br>(0.094)		0.948***<br>(0.059)		1.007***<br>(0.093)		0.494***<br>(0.068)		1.100***<br>(0.080)
Recipient of US HIV aid before 2004 (=1 if yes)		-0.0728*<br>(0.032)		-0.00921<br>(0.044)		-0.0185<br>(0.038)		-0.0615<br>(0.043)		0.0313<br>(0.041)		0.123<br>(0.064)
Constant	4.004***<br>(0.073)	14.78***<br>(0.528)	4.004***<br>(0.073)	13.68***<br>(0.845)	4.004***<br>(0.073)	16.03***<br>(0.817)	4.004***<br>(0.073)	14.37***<br>(0.725)	4.004***<br>(0.073)	21.82***<br>(0.969)	4.004***<br>(0.073)	10.69***<br>(1.021)
Adjusted R-squared	0.263	0.822	0.478	0.821	0.187	0.816	0.492	0.836	0.183	0.809	0.179	0.785
Observations	2628	2178	1566	1206	2070	1674	1548	1188	1548	1206	1548	1188

BMJ Publishing Group Limited (BMJ) disclaims all liability and responsibility arising from any reliance placed on this supplemental material which has been supplied by the author(s)

Notes: Standard errors in parentheses *** $p<0.001$, ** $p<0.01$, * $p<0.05$ BL=baseline; COP=country operational plans; PEPFAR= President's Emergency Plan for AIDS Relief

Table S4-6. Full model results for the indicator "Probability of a child dying between birth and 5 years of age, per 1,000 live births"

Variables	All PEPFAR countries		COP countries		Non-COP PEPFAR countries		High-intensity PEPFAR countries		Medium-intensity PEPFAR countries		Low-intensity PEPFAR countries	
	Model 1	Model 2	Model 1	Model 2	Model 1	Model 2	Model 1	Model 2	Model 1	Model 2	Model 1	Model 2
Time variable (=1 post-2004)	-0.526*** (0.035)	-0.479*** (0.025)	-0.526*** (0.035)	-0.479*** (0.025)	-0.526*** (0.035)	-0.479*** (0.024)	-0.526*** (0.035)	-0.479*** (0.024)	-0.526*** (0.035)	-0.479*** (0.023)	-0.526*** (0.035)	-0.479*** (0.023)
Intervention (=1 if PEPFAR)	1.013*** (0.033)	0.162*** (0.025)	1.309*** (0.037)	0.188*** (0.045)	0.857*** (0.037)	0.0812** (0.025)	1.337*** (0.037)	0.0916* (0.045)	0.821*** (0.047)	0.0452 (0.027)	0.881*** (0.045)	-0.00393 (0.031)
Interaction term (PEPFAR impact estimate)	-0.0390 (0.047)	-0.0860** (0.027)	0.00754 (0.051)	-0.0516 (0.032)	-0.0634 (0.054)	-0.104*** (0.028)	-0.00887 (0.052)	-0.0759* (0.032)	-0.0656 (0.068)	-0.0963** (0.030)	-0.0425 (0.066)	-0.0854** (0.032)
Country income level (=1 if middle income)		-0.206*** (0.021)		-0.0972** (0.033)		-0.240*** (0.026)		-0.0970** (0.033)		-0.139*** (0.031)		-0.326*** (0.037)
Ln(BL Population)		0.0165*** (0.004)		0.0165** (0.006)		0.0161** (0.005)		0.000547 (0.007)		-0.00462 (0.006)		0.0136* (0.006)
Ln(BL other donor health spending per capita)		-0.0176** (0.007)		0.000581 (0.008)		-0.0215** (0.007)		-0.0148 (0.008)		-0.0362*** (0.007)		-0.0123 (0.008)
Ln(BL domestic health spending per capita)		-0.116*** (0.015)		-0.139*** (0.026)		-0.0719*** (0.018)		-0.0884** (0.029)		-0.117*** (0.023)		-0.0820*** (0.023)
Ln(BL GDP per capita)		-0.0146 (0.020)		0.0262 (0.037)		-0.0479* (0.021)		-0.0577 (0.036)		-0.0125 (0.024)		-0.0515 (0.029)
Ln(BL HIV prevalence (% of population ages 15-49))		-0.0205*** (0.003)		-0.0314*** (0.004)		-0.0174*** (0.003)		-0.0228*** (0.004)		-0.0229*** (0.003)		-0.0187*** (0.004)
Ln(BL life expectancy at birth)		-2.372*** (0.072)		-2.435*** (0.112)		-3.076*** (0.117)		-2.526*** (0.102)		-3.689*** (0.140)		-2.983*** (0.187)
Ln(BL Urban population (%))		0.199*** (0.016)		0.204*** (0.022)		0.150*** (0.022)		0.239*** (0.023)		0.149*** (0.022)		0.192*** (0.028)
Ln(BL School enrollment, secondary (% gross))		-0.139*** (0.020)		-0.212*** (0.032)		-0.128*** (0.023)		-0.187*** (0.032)		-0.309*** (0.034)		-0.142*** (0.029)
Ln(BL Fertility rate (births per woman))		0.483*** (0.027)		0.367*** (0.050)		0.423*** (0.029)		0.450*** (0.050)		0.198*** (0.035)		0.451*** (0.042)
Recipient of US HIV aid before 2004 (=1 if yes)		0.0436** (0.014)		0.100*** (0.025)		0.126*** (0.016)		0.135*** (0.029)		0.167*** (0.021)		0.273*** (0.031)
Constant	3.347*** (0.024)	13.62*** (0.344)	3.347*** (0.024)	13.91*** (0.561)	3.347*** (0.024)	16.90*** (0.477)	3.347*** (0.024)	14.70*** (0.534)	3.347*** (0.024)	20.64*** (0.585)	3.347*** (0.024)	16.56*** (0.744)
Adjusted R-squared	0.343	0.847	0.487	0.814	0.281	0.841	0.492	0.817	0.256	0.816	0.283	0.808
Observations	4408	3596	2697	2030	3509	2784	2668	2001	2668	2030	2668	2001

Notes: Standard errors in parentheses *** p<0.001, ** p<0.01, * p<0.05 BL=baseline; COP=country operational plans; PEPFAR= President's Emergency Plan for AIDS Relief

Table S4-7. Full model results for the indicator "Prevalence of anemia among women of reproductive age (% of women ages 15-49)"

Variables	All PEPFAR countries		COP countries		Non-COP PEPFAR countries		High-intensity PEPFAR countries		Medium-intensity PEPFAR countries		Low-intensity PEPFAR countries	
	Model 1	Model 2	Model 1	Model 2	Model 1	Model 2	Model 1	Model 2	Model 1	Model 2	Model 1	Model 2
Time variable (=1 post-2004)	-0.176*** (0.015)	-0.206*** (0.017)	-0.176*** (0.015)	-0.206*** (0.017)	-0.176*** (0.015)	-0.206*** (0.016)	-0.176*** (0.015)	-0.206*** (0.017)	-0.176*** (0.015)	-0.206*** (0.015)	-0.176*** (0.015)	-0.206*** (0.016)
Intervention (=1 if PEPFAR)	0.227*** (0.014)	0.0302 (0.017)	0.294*** (0.016)	0.106*** (0.029)	0.192*** (0.016)	-0.0199 (0.017)	0.286*** (0.016)	0.0870** (0.031)	0.201*** (0.021)	-0.0353 (0.019)	0.193*** (0.020)	-0.0345 (0.022)
Interaction term (PEPFAR impact estimate)	-0.0116 (0.021)	0.0140 (0.020)	-0.0224 (0.024)	0.00718 (0.024)	-0.00592 (0.024)	0.0176 (0.020)	-0.0409 (0.025)	-0.0106 (0.025)	-0.000701 (0.030)	0.0219 (0.023)	0.00682 (0.029)	0.0305 (0.022)
Country income level (=1 if middle income)		-0.258*** (0.020)		-0.202*** (0.026)		-0.234*** (0.025)		-0.241*** (0.027)		-0.124*** (0.028)		-0.265*** (0.033)
BL Population		-0.000913 (0.003)		-0.00223 (0.004)		0.00358 (0.003)		-0.00821 (0.004)		-0.0144*** (0.004)		0.0100** (0.004)
BL other donor health spending per capita		-0.0318*** (0.004)		-0.0244*** (0.005)		-0.0336*** (0.004)		-0.0216*** (0.005)		-0.0662*** (0.005)		-0.0287*** (0.005)
BL domestic health spending per capita		-0.0960*** (0.011)		-0.0535*** (0.016)		-0.0606*** (0.014)		-0.0166 (0.017)		-0.0231 (0.017)		-0.0766*** (0.015)
BL GDP per capita		0.143*** (0.012)		0.181*** (0.016)		0.129*** (0.012)		0.171*** (0.016)		0.0557*** (0.015)		0.156*** (0.011)
BL HIV prevalence (% of population ages 15-49)		-0.0198*** (0.002)		-0.0357*** (0.003)		-0.0142*** (0.002)		-0.0335*** (0.003)		-0.0229*** (0.002)		-0.0170*** (0.002)
BL life expectancy at birth		-0.584*** (0.048)		-0.237*** (0.068)		-1.234*** (0.077)		-0.283*** (0.063)		-1.371*** (0.104)		-0.958*** (0.088)
BL Urban population (%)		0.0726*** (0.014)		-0.0212 (0.019)		-0.0464** (0.017)		-0.0150 (0.020)		-0.0873*** (0.020)		-0.111*** (0.021)
BL School enrollment, secondary (% gross)		-0.0676*** (0.014)		-0.148*** (0.024)		-0.0668*** (0.017)		-0.173*** (0.024)		-0.302*** (0.025)		-0.0469* (0.021)
BL Fertility rate (births per woman)		0.243*** (0.020)		0.222*** (0.033)		0.168*** (0.021)		0.230*** (0.034)		0.0626* (0.026)		0.206*** (0.028)
Recipient of US HIV aid before 2004 (=1 if yes)		-0.0162 (0.012)		0.137*** (0.018)		-0.00280 (0.014)		0.120*** (0.021)		0.0369* (0.019)		0.0342 (0.022)
Constant	3.449*** (0.011)	5.184*** (0.233)	3.449*** (0.011)	3.748*** (0.345)	3.449*** (0.011)	8.333*** (0.327)	3.449*** (0.011)	4.024*** (0.340)	3.449*** (0.011)	10.77*** (0.445)	3.449*** (0.011)	7.049*** (0.359)
Adjusted R-squared	0.159	0.524	0.238	0.449	0.129	0.590	0.221	0.442	0.134	0.580	0.135	0.560
Observations	3996	3321	2403	1863	3159	2565	2376	1836	2376	1863	2376	1836

Notes: Standard errors in parentheses *** p<0.001, ** p<0.01, * p<0.05 BL=baseline; COP=country operational plans; PEPFAR= President's Emergency Plan for AIDS Relief

S5. Full model of unlogged regression of seven outcomes from staggered DID

Table S5-1. Full model results for the indicator "Percent of children ages 12-23 months who received DPT vaccinations (3 doses)"

Variables	All PEPFAR countries		COP countries		Non-COP PEPFAR countries		High-intensity PEPFAR countries		Medium-intensity PEPFAR countries		Low-intensity PEPFAR countries	
	Model 1	Model 2	Model 1	Model 2	Model 1	Model 2	Model 1	Model 2	Model 1	Model 2	Model 1	Model 2
Intervention (=1 if PEPFAR)	-14.85*** (0.802)	-4.304*** (0.559)	-17.09*** (1.199)	-10.68*** (1.206)	-14.45*** (0.922)	-3.359*** (0.638)	-16.66*** (1.240)	-5.981*** (1.266)	-10.83*** (1.103)	-3.004*** (0.858)	-18.07*** (1.253)	-4.129*** (0.852)
Treatment (=1 for the years PEPFAR starts and after)	8.206*** (0.992)	7.328*** (0.593)	6.538*** (1.461)	7.459*** (0.911)	10.80*** (1.104)	8.334*** (0.681)	8.373*** (1.498)	8.326*** (0.899)	4.149** (1.364)	6.836*** (0.964)	14.49*** (1.473)	8.979*** (0.875)
Country income level (=1 if middle income)		-4.911*** (0.649)		-6.629*** (0.850)		-2.615*** (0.765)		-5.702*** (0.883)		-1.832* (0.871)		-2.634** (0.980)
BL Population		-1.75e-09 (0.000)		-7.40e-09*** (0.000)		-2.10e-09* (0.000)		-1.05e-08 (0.000)		-5.58e-08*** (0.000)		-3.31e-09*** (0.000)
BL other donor health spending per capita		0.00318 (0.008)		-0.0196* (0.008)		-0.00465 (0.008)		-0.0154 (0.008)		-0.0168* (0.008)		-0.0180* (0.009)
BL domestic health spending per capita		-0.00474*** (0.001)		-0.00302* (0.001)		-0.00608*** (0.001)		-0.00608*** (0.001)		-0.00777*** (0.001)		-0.00396** (0.001)
BL GDP per capita		0.000120 (0.000)		-0.00000602 (0.000)		0.000131* (0.000)		0.0000389 (0.000)		0.000180*** (0.000)		0.0000538 (0.000)
BL HIV prevalence (% of population ages 15-49)		0.823*** (0.070)		0.680*** (0.088)		-0.675* (0.316)		0.478*** (0.077)		-1.646** (0.567)		-1.378*** (0.415)
BL diphtheria prevalence (% of population, under 5)		-3820.2*** (537.869)		-5143.0*** (573.583)		-4092.6*** (895.923)		-4071.4*** (666.350)		-1930.0 (1677.185)		-4972.3*** (944.550)
BL tetanus prevalence (% of population, under 5)		-341.2** (109.133)		389.8** (135.183)		-526.3*** (129.114)		-72.51 (158.000)		-44.21 (147.707)		-921.9*** (136.270)
BL pertussis (whooping cough) prevalence (% of population, under 5)		-2568.6*** (112.485)		-2581.9*** (144.914)		-2080.6*** (122.128)		-2806.0*** (136.893)		-2212.6*** (149.232)		-1976.4*** (120.957)
BL life expectancy at birth		0.639*** (0.066)		0.324*** (0.085)		0.455*** (0.070)		0.306*** (0.071)		0.448*** (0.082)		0.216** (0.081)
BL Urban population (%)		-0.0291* (0.012)		-0.0380** (0.013)		-0.00745 (0.012)		-0.0349** (0.012)		0.0345** (0.013)		-0.0406*** (0.012)
BL School enrollment, secondary (% gross)		0.0804*** (0.014)		0.0725*** (0.017)		0.0768*** (0.015)		0.0435** (0.016)		0.0685*** (0.016)		0.0316* (0.016)
BL Fertility rate (births per woman)		-0.552 (0.282)		-1.485*** (0.388)		-1.434*** (0.287)		-2.477*** (0.357)		-0.920* (0.362)		-3.016*** (0.307)

Recipient of US HIV aid before 2004 (=1 if yes)		-1.621*** (0.487)		-0.356 (0.737)		-2.125*** (0.593)		-1.480 (0.871)		-2.957*** (0.808)		-2.217** (0.726)
Constant	84.78*** (1.761)	53.74*** (5.391)	86.15*** (1.800)	83.46*** (6.642)	85.39*** (1.971)	65.45*** (5.862)	86.68*** (1.800)	90.42*** (5.691)	84.99*** (2.062)	64.31*** (6.481)	86.34*** (2.002)	93.42*** (6.346)
Adjusted R-squared	0.152	0.663	0.173	0.668	0.140	0.670	0.156	0.695	0.094	0.572	0.160	0.719
Observations	4282	3926	2628	2328	3386	3087	2592	2263	2565	2322	2589	2319

Notes: Standard errors in parentheses *** p<0.001, ** p<0.01, * p<0.05 BL=baseline; COP=country operational plans; PEPFAR= President's Emergency Plan for AIDS Relief

Table S5-2. Full model results for the indicator "Percentage of children age 12-23 months who received hepatitis B vaccines, 3 doses) "

Variables	All PEPFAR countries		COP countries		Non-COP PEPFAR countries		High-intensity PEPFAR countries		Medium-intensity PEPFAR countries		Low-intensity PEPFAR countries	
	Model 1	Model 2	Model 1	Model 2	Model 1	Model 2	Model 1	Model 2	Model 1	Model 2	Model 1	Model 2
Intervention (=1 if PEPFAR)	-12.21***	-7.517***	-26.51***	-26.10***	-7.571***	-3.643	-17.68***	-12.76**	-10.08***	-7.392**	-12.64***	-4.294
	(1.898)	(1.755)	(3.197)	(3.375)	(2.115)	(1.943)	(4.480)	(4.488)	(2.490)	(2.572)	(3.120)	(2.746)
Treatment (=1 for the years PEPFAR starts and after)	5.436**	10.52***	16.33***	21.33***	3.000	7.050***	10.78*	17.15***	3.096	6.967**	6.397	9.912***
	(1.963)	(1.827)	(3.311)	(3.220)	(2.205)	(2.076)	(4.545)	(4.303)	(2.624)	(2.600)	(3.277)	(2.839)
Country income level (=1 if middle income)		-3.539**		-6.157***		-3.752**		-5.545***		-4.939**		-3.550
		(1.140)		(1.676)		(1.337)		(1.633)		(1.605)		(1.916)
BL Population		-1.17e-08**		-3.30e-08***		-2.41e-09		-0.000000104***		-8.32e-08***		-1.54e-08***
		(0.000)		(0.000)		(0.000)		(0.000)		(0.000)		(0.000)
BL other donor health spending per capita		0.0312**		0.0230		0.0277*		0.0194		0.0191		0.0509***
		(0.011)		(0.012)		(0.012)		(0.012)		(0.012)		(0.013)
BL domestic health spending per capita		-0.00869**		-0.00398		-0.0113***		-0.00652*		-0.0112***		-0.0104***
		(0.003)		(0.003)		(0.003)		(0.003)		(0.003)		(0.003)
BL GDP per capita		0.000426***		0.000390**		0.000446***		0.000389**		0.000441***		0.000530***
		(0.000)		(0.000)		(0.000)		(0.000)		(0.000)		(0.000)
BL HIV prevalence (% of population ages 15-49)		0.713***		0.751***		-0.0858		0.235		-0.233		-1.404
		(0.109)		(0.140)		(0.658)		(0.124)		(1.231)		(0.890)
BL hepatitis B prevalence (% of population, under 5)		-75.75***		-83.95***		-62.05***		-84.02***		-77.59***		-3.689
		(6.747)		(10.895)		(9.734)		(9.175)		(11.448)		(15.177)
BL life expectancy at birth		0.566***		0.370*		0.307**		0.227		0.453**		0.573***
		(0.107)		(0.161)		(0.115)		(0.143)		(0.151)		(0.143)
BL Urban population (%)		-0.0318		-0.116***		-0.00460		-0.0875***		0.0106		-0.0554*
		(0.021)		(0.023)		(0.023)		(0.022)		(0.026)		(0.024)
BL School enrollment, secondary (% gross)		0.0864***		0.0847*		0.0998***		0.0779*		0.129***		0.144***
		(0.024)		(0.035)		(0.026)		(0.033)		(0.030)		(0.030)
BL Fertility rate (births per woman)		0.0261		-1.001		-1.844***		-2.578***		-0.554		-3.787***
		(0.477)		(0.640)		(0.501)		(0.655)		(0.624)		(0.634)
Recipient of US HIV aid before 2004 (=1 if yes)		-2.917***		3.818**		-2.991**		1.791		-0.307		-4.525*
		(0.851)		(1.361)		(1.100)		(1.462)		(1.535)		(1.775)
Constant	58.54***	19.80	60.92***	45.55**	63.80***	46.74**	63.80***	63.54***	58.18***	27.72	63.80***	29.63
	(12.180)	(13.776)	(11.556)	(16.530)	(13.426)	(15.388)	(13.454)	(16.797)	(12.354)	(15.964)	(13.452)	(16.476)
Adjusted R-squared	0.138	0.341	0.218	0.410	0.114	0.324	0.150	0.390	0.159	0.317	0.136	0.377
Observations	2799	2579	1789	1598	2270	2065	1744	1541	1809	1633	1766	1573

Notes: Standard errors in parentheses *** p<0.001, ** p<0.01, * p<0.05 BL=baseline; COP=country operational plans; PEPFAR= President's Emergency Plan for AIDS Relief

Table S5-3. Full model results for the indicator "Percent of children ages 12-23 months who received the measles vaccination"

Variables	All PEPFAR countries		COP countries		Non-COP PEPFAR countries		High-intensity PEPFAR countries		Medium-intensity PEPFAR countries		Low-intensity PEPFAR countries	
	Model 1	Model 2	Model 1	Model 2	Model 1	Model 2	Model 1	Model 2	Model 1	Model 2	Model 1	Model 2
Intervention (=1 if PEPFAR)	-14.30*** (0.773)	-5.796*** (0.636)	-16.04*** (1.118)	-10.16*** (1.255)	-13.97*** (0.885)	-4.759*** (0.708)	-15.53*** (1.119)	-9.263*** (1.283)	-10.96*** (1.079)	-5.385*** (1.000)	-17.04*** (1.194)	-4.471*** (0.841)
Treatment (=1 for the years PEPFAR starts and after)	6.816*** (0.952)	5.515*** (0.677)	5.091*** (1.344)	5.546*** (0.997)	9.077*** (1.089)	6.115*** (0.753)	5.692*** (1.355)	5.158*** (0.970)	3.280* (1.365)	5.357*** (1.036)	13.28*** (1.417)	7.083*** (0.885)
Country income level (=1 if middle income)		-5.795*** (0.635)		-7.742*** (0.835)		-4.200*** (0.799)		-7.702*** (0.893)		-6.163*** (0.989)		-3.973*** (0.974)
BL Population		-4.03e-09*** (0.000)		-9.32e-09*** (0.000)		-1.48e-09 (0.000)		-7.25e-08*** (0.000)		-1.38e-08 (0.000)		-6.01e-09*** (0.000)
BL other donor health spending per capita		0.0581*** (0.009)		0.0501*** (0.010)		0.0399*** (0.009)		0.0360*** (0.009)		0.0373*** (0.009)		0.0456*** (0.009)
BL domestic health spending per capita		-0.00649*** (0.001)		-0.00679*** (0.001)		-0.00476*** (0.001)		-0.00518*** (0.001)		-0.00418** (0.001)		-0.00523*** (0.001)
BL GDP per capita		0.000250*** (0.000)		0.000295*** (0.000)		0.000178*** (0.000)		0.000226*** (0.000)		0.000167** (0.000)		0.000264*** (0.000)
BL HIV prevalence (% of population ages 15-49)		1.212*** (0.067)		1.168*** (0.075)		-0.117 (0.318)		1.056*** (0.076)		0.194 (0.565)		0.179 (0.395)
BL measles prevalence (% of population, under 5)		-17.01*** (1.341)		-21.20*** (1.657)		-5.621*** (1.554)		-20.76*** (1.681)		-0.661 (1.794)		-11.01*** (1.774)
BL life expectancy at birth		0.839*** (0.062)		0.724*** (0.075)		0.638*** (0.071)		0.705*** (0.072)		0.732*** (0.086)		0.646*** (0.079)
BL Urban population (%)		-0.0128 (0.013)		-0.0545*** (0.012)		0.0294* (0.013)		-0.0415*** (0.012)		0.0301* (0.013)		-0.0286* (0.012)
BL School enrollment, secondary (% gross)		0.0855*** (0.013)		0.0562*** (0.017)		0.131*** (0.014)		0.0772*** (0.016)		0.122*** (0.017)		0.126*** (0.015)
BL Fertility rate (births per woman)		-2.767*** (0.275)		-3.176*** (0.349)		-3.321*** (0.292)		-2.496*** (0.352)		-3.439*** (0.375)		-3.855*** (0.288)
Recipient of US HIV aid before 2004 (=1 if yes)		1.307* (0.524)		2.753** (0.842)		2.090*** (0.587)		3.695*** (0.907)		1.434 (0.880)		1.588* (0.765)
Constant	82.28*** (1.616)	32.25*** (5.134)	82.36*** (1.775)	48.39*** (5.926)	82.88*** (1.811)	39.84*** (5.903)	83.19*** (1.797)	46.44*** (5.851)	81.93*** (1.890)	35.17*** (6.957)	82.77*** (1.952)	44.05*** (6.303)
Adjusted R-squared	0.160	0.588	0.190	0.574	0.143	0.615	0.174	0.574	0.112	0.519	0.162	0.680
Observations	4275	3926	2621	2328	3379	3087	2585	2263	2558	2322	2582	2319

Notes: Standard errors in parentheses *** p<0.001, ** p<0.01, * p<0.05 BL=baseline; COP=country operational plans; PEPFAR= President's Emergency Plan for AIDS Relief

Table S5-4. Full model results for the indicator "Percentage of births by women of child-bearing age who are immunized against tetanus"

Variables	All PEPFAR countries		COP countries		Non-COP PEPFAR countries		High-intensity PEPFAR countries		Medium-intensity PEPFAR countries		Low-intensity PEPFAR countries	
	Model 1	Model 2	Model 1	Model 2	Model 1	Model 2	Model 1	Model 2	Model 1	Model 2	Model 1	Model 2
Intervention (=1 if PEPFAR)	-5.962*** (1.037)	1.698 (1.093)	-6.498*** (1.422)	-3.279 (1.790)	-6.051*** (1.151)	3.261** (1.233)	-7.470*** (1.394)	-3.572 (1.887)	-4.661** (1.574)	0.572 (1.674)	-6.488*** (1.349)	6.202*** (1.266)
Treatment (=1 for the years PEPFAR starts and after)	5.218*** (1.049)	3.619*** (1.014)	5.444*** (1.518)	4.053** (1.447)	5.942*** (1.228)	4.103*** (1.170)	7.005*** (1.483)	5.475*** (1.446)	4.966** (1.720)	5.391** (1.638)	5.401*** (1.474)	1.022 (1.242)
Country income level (=1 if middle income)		0.0616 (0.888)		2.662* (1.126)		0.277 (1.337)		1.422 (1.267)		-0.621 (1.786)		-0.364 (1.583)
BL Population		7.32e-09*** (0.000)		4.12e-09* (0.000)		3.11e-09 (0.000)		-9.92e-08*** (0.000)		-2.91e-08 (0.000)		5.24e-10 (0.000)
BL other donor health spending per capita		0.476*** (0.045)		0.180** (0.055)		0.472*** (0.050)		0.134* (0.059)		0.400*** (0.062)		0.334*** (0.048)
BL domestic health spending per capita		0.00687* (0.003)		-0.00487 (0.005)		0.00968** (0.003)		0.00411 (0.005)		0.0161*** (0.005)		0.0108*** (0.003)
BL GDP per capita		0.000394*** (0.000)		0.000666*** (0.000)		0.000415*** (0.000)		0.000443*** (0.000)		0.0000186 (0.000)		0.000589*** (0.000)
BL HIV prevalence (% of population ages 15-49)		-0.000776 (0.099)		0.354** (0.121)		-1.559** (0.513)		0.115 (0.121)		0.206 (0.841)		-1.416* (0.574)
BL life expectancy at birth		0.0388 (0.096)		0.335* (0.135)		-0.489*** (0.133)		0.237 (0.125)		-0.539*** (0.160)		-0.416* (0.165)
BL Urban population (%)		-0.162*** (0.022)		-0.270*** (0.029)		-0.166*** (0.027)		-0.260*** (0.027)		-0.120*** (0.032)		-0.320*** (0.031)
BL School enrollment, secondary (% gross)		0.000962 (0.022)		-0.0445 (0.032)		0.0192 (0.027)		-0.0273 (0.036)		0.198*** (0.036)		-0.0312 (0.031)
BL Fertility rate (births per woman)		-3.193*** (0.460)		-2.913*** (0.613)		-5.457*** (0.595)		-2.330** (0.769)		-3.805*** (0.651)		-8.096*** (0.590)
Recipient of US HIV aid before 2004 (=1 if yes)		0.303 (0.656)		5.835*** (1.088)		-1.239 (0.804)		5.876*** (1.041)		0.975 (1.275)		-1.459 (1.087)
Constant	58.82*** (2.478)	63.39*** (8.043)	60.35*** (3.064)	52.43*** (10.716)	58.95*** (3.007)	105.5*** (11.324)	59.55*** (3.087)	56.95*** (11.082)	60.39*** (3.513)	94.79*** (13.137)	60.16*** (3.493)	119.8*** (12.960)
Adjusted R-squared	0.325	0.444	0.290	0.422	0.307	0.464	0.318	0.452	0.261	0.380	0.263	0.546
Observations	2830	2657	1624	1480	1964	1848	1614	1441	1336	1249	1396	1309

Notes: Standard errors in parentheses *** p<0.001, ** p<0.01, * p<0.05 BL=baseline; COP=country operational plans; PEPFAR= President's Emergency Plan for AIDS Relief

Table S5-5. Full model results for the indicator "Number of women who die from pregnancy-related causes while pregnant or within 42 days of pregnancy termination per 100,000 live births"

Variables	All PEPFAR countries		COP countries		Non-COP PEPFAR countries		High-intensity PEPFAR countries		Medium-intensity PEPFAR countries		Low-intensity PEPFAR countries	
	Model 1	Model 2	Model 1	Model 2	Model 1	Model 2	Model 1	Model 2	Model 1	Model 2	Model 1	Model 2
Intervention (=1 if PEPFAR)	355.4*** (26.828)	80.00*** (12.827)	412.6*** (38.080)	133.1*** (26.575)	359.5*** (30.661)	69.38*** (13.947)	486.8*** (38.028)	298.9*** (24.624)	195.9*** (41.087)	53.63* (22.027)	462.5*** (38.059)	141.0*** (19.905)
Treatment (=1 for the years PEPFAR starts and after)	-109.3*** (28.820)	-60.11*** (14.914)	-82.58* (39.845)	-80.42*** (24.385)	-171.6*** (33.208)	-58.73*** (16.511)	-145.1*** (39.857)	-118.6*** (23.209)	19.84 (44.065)	-53.09* (26.557)	-310.2*** (40.523)	-95.90*** (21.931)
Country income level (=1 if middle income)		-11.16 (12.778)		-53.87*** (12.214)		5.238 (14.585)		-52.15*** (12.726)		9.295 (17.031)		-18.82 (14.331)
BL Population		1.56e-08 (0.000)		-4.26e-09 (0.000)		2.25e-08* (0.000)		0.00000154*** (0.000)		-8.65e-08 (0.000)		-2.62e-08** (0.000)
BL other donor health spending per capita		-1.019*** (0.131)		-0.585*** (0.084)		-0.960*** (0.131)		-0.322*** (0.058)		-0.841*** (0.123)		-0.626*** (0.103)
BL domestic health spending per capita		0.234*** (0.019)		0.128*** (0.017)		0.284*** (0.022)		0.0901*** (0.015)		0.271*** (0.028)		0.183*** (0.021)
BL GDP per capita		-0.00644*** (0.001)		-0.00247*** (0.000)		-0.00812*** (0.001)		-0.00150*** (0.000)		-0.00682*** (0.001)		-0.00560*** (0.001)
BL HIV prevalence (% of population ages 15-49)		-18.06*** (1.697)		-8.348*** (1.413)		2.198 (6.563)		-10.47*** (1.106)		-9.512 (8.684)		23.58** (7.206)
BL life expectancy at birth		-27.28*** (1.685)		-16.44*** (1.328)		-27.02*** (2.227)		-16.07*** (1.048)		-29.07*** (3.032)		-14.02*** (1.414)
BL Urban population (%)		-0.0358 (0.183)		-0.202 (0.159)		-0.355 (0.193)		-0.489** (0.155)		-0.127 (0.211)		-0.439** (0.161)
BL School enrollment, secondary (% gross)		-1.400*** (0.200)		-0.832*** (0.192)		-1.919*** (0.201)		-1.160*** (0.176)		-1.400*** (0.205)		-2.094*** (0.216)
BL Fertility rate (births per woman)		23.31*** (4.534)		27.28*** (3.611)		24.11*** (4.863)		12.70** (4.108)		20.92*** (4.679)		36.01*** (4.603)
Recipient of US HIV aid before 2004 (=1 if yes)		-52.71*** (8.684)		-17.72 (11.645)		-37.63*** (9.542)		-141.1*** (10.915)		-25.98 (15.371)		-31.98** (9.902)
Constant	109.0*** (30.611)	2103.9*** (123.188)	121.7*** (24.827)	1314.3*** (90.961)	81.44* (33.768)	2105.6*** (161.891)	103.1*** (24.677)	1343.5*** (76.467)	146.4*** (34.214)	2198.5*** (217.129)	48.75 (32.077)	1181.9*** (98.242)
Adjusted R-squared	0.203	0.776	0.445	0.814	0.171	0.782	0.471	0.862	0.151	0.723	0.249	0.795
Observations	2628	2412	1566	1386	2070	1890	1548	1350	1548	1404	1548	1386

Notes: Standard errors in parentheses *** p<0.001, ** p<0.01, * p<0.05 BL=baseline; COP=country operational plans; PEPFAR= President's Emergency Plan for AIDS Relief

Table S5-6. Full model results for the indicator "Probability of a child dying between birth and 5 years of age, per 1,000 live births"

Variables	All PEPFAR countries		COP countries		Non-COP PEPFAR countries		High-intensity PEPFAR countries		Medium-intensity PEPFAR countries		Low-intensity PEPFAR countries	
	**Model 1**	**Model 2**	**Model 1**	**Model 2**	**Model 1**	**Model 2**	**Model 1**	**Model 2**	**Model 1**	**Model 2**	**Model 1**	**Model 2**
Intervention (=1 if PEPFAR)	60.72*** (2.098)	9.613*** (1.098)	82.72*** (3.063)	15.97*** (2.302)	53.72*** (2.443)	7.436*** (1.148)	88.03*** (3.236)	32.41*** (3.127)	43.97*** (3.147)	4.532*** (1.371)	59.82*** (3.158)	11.02*** (1.654)
Treatment (=1 for the years PEPFAR starts and after)	-22.41*** (2.529)	-20.65*** (1.098)	-30.81*** (3.485)	-32.80*** (1.957)	-26.47*** (2.909)	-18.44*** (1.171)	-36.15*** (3.633)	-37.75*** (2.159)	-11.73** (3.798)	-19.11*** (1.532)	-37.12*** (3.634)	-19.56*** (1.488)
Country income level (=1 if middle income)		-2.241 (1.242)		2.733 (1.938)		-5.415*** (1.429)		3.657 (1.992)		-5.732*** (1.531)		-6.059** (2.110)
BL Population		9.64e-09*** (0.000)		1.30e-08*** (0.000)		1.02e-08*** (0.000)		6.95e-08*** (0.000)		6.18e-08*** (0.000)		6.47e-09*** (0.000)
BL other donor health spending per capita		-0.263*** (0.019)		-0.210*** (0.019)		-0.258*** (0.020)		-0.202*** (0.019)		-0.239*** (0.020)		-0.203*** (0.019)
BL domestic health spending per capita		0.0147*** (0.002)		0.00330 (0.002)		0.0196*** (0.002)		-0.00144 (0.003)		0.0194*** (0.002)		0.0105*** (0.002)
BL GDP per capita		-0.000464*** (0.000)		0.0000298 (0.000)		-0.000578*** (0.000)		0.000104 (0.000)		-0.000480*** (0.000)		-0.000310*** (0.000)
BL HIV prevalence (% of population ages 15-49)		-2.397*** (0.144)		-1.964*** (0.161)		-0.936 (0.586)		-2.352*** (0.178)		1.191 (0.819)		0.737 (0.775)
BL life expectancy at birth		-3.953*** (0.112)		-3.675*** (0.148)		-3.958*** (0.141)		-3.775*** (0.142)		-4.159*** (0.172)		-3.107*** (0.160)
BL Urban population (%)		-0.00291 (0.023)		0.0506* (0.022)		-0.0748*** (0.022)		0.0715** (0.025)		-0.0648** (0.021)		-0.0700** (0.021)
BL School enrollment, secondary (% gross)		-0.219*** (0.023)		-0.270*** (0.030)		-0.207*** (0.023)		-0.255*** (0.032)		-0.139*** (0.025)		-0.241*** (0.026)
BL Fertility rate (births per woman)		7.117*** (0.495)		6.249*** (0.591)		6.726*** (0.536)		5.146*** (0.670)		6.211*** (0.506)		7.277*** (0.649)
Recipient of US HIV aid before 2004 (=1 if yes)		0.939 (0.955)		6.445*** (1.525)		1.772 (0.974)		-3.306 (2.242)		3.206** (1.242)		2.400 (1.351)
Constant	50.14*** (4.776)	334.4*** (8.895)	45.67*** (4.779)	306.7*** (12.055)	49.05*** (5.219)	340.5*** (10.837)	45.22*** (4.967)	314.4*** (11.918)	50.38*** (5.249)	345.9*** (12.926)	45.60*** (5.259)	279.5*** (11.904)
Adjusted R-squared	0.313	0.855	0.494	0.856	0.264	0.859	0.504	0.850	0.242	0.856	0.293	0.854
Observations	4408	4002	2697	2349	3509	3161	2668	2291	2668	2378	2668	2349

Notes: Standard errors in parentheses *** p<0.001, ** p<0.01, * p<0.05 BL=baseline; COP=country operational plans; PEPFAR= President's Emergency Plan for AIDS Relief

Table S5-7. Full model results for the indicator "Prevalence of anemia among women of reproductive age (% of women ages 15-49)"

Variables	All PEPFAR countries		COP countries		Non-COP PEPFAR countries		High-intensity PEPFAR countries		Medium-intensity PEPFAR countries		Low-intensity PEPFAR countries	
	**Model 1**	**Model 2**	**Model 1**	**Model 2**	**Model 1**	**Model 2**	**Model 1**	**Model 2**	**Model 1**	**Model 2**	**Model 1**	**Model 2**
Intervention (=1 if PEPFAR)	8.473*** (0.467)	-1.293** (0.409)	10.09*** (0.592)	-2.824*** (0.828)	7.934*** (0.541)	-3.064*** (0.415)	9.923*** (0.615)	-1.341 (0.959)	6.922*** (0.716)	-3.062*** (0.494)	8.935*** (0.661)	-3.030*** (0.630)
Treatment (=1 for the years PEPFAR starts and after)	-1.246* (0.630)	-0.422 (0.457)	-1.131 (0.798)	-1.091 (0.707)	-1.990* (0.772)	0.00201 (0.483)	-1.672* (0.836)	-1.715* (0.770)	1.286 (1.031)	-0.547 (0.609)	-4.197*** (0.930)	0.0226 (0.577)
Country income level (=1 if middle income)		-5.748*** (0.537)		-0.474 (0.724)		-7.546*** (0.675)		-1.088 (0.791)		-4.592*** (0.726)		-9.547*** (0.993)
BL Population		3.62e-09*** (0.000)		1.21e-08*** (0.000)		-2.66e-10 (0.000)		-1.16e-08 (0.000)		3.42e-08*** (0.000)		2.27e-09* (0.000)
BL other donor health spending per capita		-0.0717*** (0.003)		-0.0633*** (0.004)		-0.0685*** (0.003)		-0.0628*** (0.004)		-0.0797*** (0.004)		-0.0605*** (0.004)
BL domestic health spending per capita		-0.00835*** (0.001)		-0.00621*** (0.001)		-0.00420*** (0.001)		-0.00712*** (0.001)		0.000541 (0.001)		-0.00470*** (0.001)
BL GDP per capita		0.000396*** (0.000)		0.000380*** (0.000)		0.000315*** (0.000)		0.000416*** (0.000)		0.000224*** (0.000)		0.000315*** (0.000)
BL HIV prevalence (% of population ages 15-49)		-0.673*** (0.042)		-0.571*** (0.050)		0.783*** (0.193)		-0.691*** (0.061)		0.765* (0.386)		0.628** (0.215)
BL life expectancy at birth		-0.584*** (0.034)		-0.475*** (0.047)		-0.452*** (0.038)		-0.543*** (0.051)		-0.598*** (0.046)		-0.248*** (0.052)
BL Urban population (%)		0.0352** (0.011)		-0.0544*** (0.012)		-0.0240* (0.011)		-0.0364** (0.013)		-0.0737*** (0.012)		-0.0698*** (0.013)
BL School enrollment, secondary (% gross)		-0.0532*** (0.010)		-0.128*** (0.013)		-0.0689*** (0.010)		-0.129*** (0.014)		-0.114*** (0.012)		-0.0621*** (0.013)
BL Fertility rate (births per woman)		1.236*** (0.189)		0.333 (0.301)		1.544*** (0.203)		-0.0349 (0.361)		1.806*** (0.221)		1.459*** (0.249)
Recipient of US HIV aid before 2004 (=1 if yes)		-0.714* (0.331)		2.638*** (0.504)		0.360 (0.374)		2.702*** (0.605)		-0.982 (0.511)		1.749** (0.548)
Constant	38.50*** (0.910)	84.97*** (2.990)	38.71*** (1.047)	84.42*** (4.341)	38.47*** (1.049)	79.70*** (3.259)	38.71*** (1.054)	90.28*** (4.815)	39.21*** (1.184)	91.53*** (3.877)	38.19*** (1.151)	69.22*** (4.362)
Adjusted R-squared	0.191	0.620	0.274	0.561	0.166	0.689	0.259	0.540	0.175	0.687	0.184	0.669
Observations	3996	3672	2403	2133	3159	2889	2376	2079	2376	2160	2376	2133

Notes: Standard errors in parentheses *** p<0.001, ** p<0.01, * p<0.05 BL=baseline; COP=country operational plans; PEPFAR= President's Emergency Plan for AIDS Relief

S6. Full model of logged regression of seven outcomes from staggered DID

Table S6-1. Full model results for the indicator "Percent of children ages 12-23 months who received DPT vaccinations (3 doses)"

Variables	All PEPFAR countries		COP countries		Non-COP PEPFAR countries		High-intensity PEPFAR countries		Medium-intensity PEPFAR countries		Low-intensity PEPFAR countries	
	Model 1	Model 2	Model 1	Model 2	Model 1	Model 2	Model 1	Model 2	Model 1	Model 2	Model 1	Model 2
Intervention (=1 if PEPFAR)	-0.233*** (0.014)	-0.0906*** (0.011)	-0.264*** (0.022)	-0.102*** (0.024)	-0.230*** (0.016)	-0.0833*** (0.012)	-0.255*** (0.022)	-0.0574* (0.026)	-0.161*** (0.019)	-0.0417** (0.014)	-0.301*** (0.023)	-0.125*** (0.016)
Treatment (=1 for the years PEPFAR starts and after)	0.147*** (0.017)	0.135*** (0.013)	0.122*** (0.026)	0.155*** (0.021)	0.190*** (0.019)	0.144*** (0.013)	0.145*** (0.026)	0.134*** (0.021)	0.0806*** (0.022)	0.141*** (0.018)	0.261*** (0.026)	0.179*** (0.019)
Country income level (=1 if middle income)		-0.0644*** (0.015)		-0.0866*** (0.020)		-0.0661*** (0.014)		-0.0873*** (0.022)		-0.0852*** (0.016)		-0.0979*** (0.018)
Ln(BL Population)		-0.00818*** (0.002)		-0.00212 (0.003)		-0.0170*** (0.002)		0.000520 (0.003)		-0.00564* (0.002)		-0.0164*** (0.002)
Ln(BL other donor health spending per capita)		0.0165*** (0.004)		0.00808 (0.005)		0.00247 (0.003)		0.00549 (0.004)		0.00999** (0.004)		0.00129 (0.004)
Ln(BL domestic health spending per capita)		-0.0181 (0.012)		0.00146 (0.014)		-0.0513*** (0.013)		-0.0280* (0.014)		0.00146 (0.013)		-0.0578*** (0.015)
Ln(BL GDP per capita)		-0.0167 (0.016)		-0.0587** (0.022)		-0.0266 (0.017)		-0.0516** (0.020)		-0.0318 (0.017)		-0.0284 (0.022)
Ln(BL HIV prevalence (% of population ages 15-49))		-0.000417 (0.001)		-0.00310 (0.002)		-0.00461*** (0.001)		-0.00488 (0.003)		-0.00409** (0.002)		-0.00257 (0.002)
Ln(BL diphtheria prevalence (% of population, under 5))		-0.0294*** (0.002)		-0.0331*** (0.003)		-0.00966*** (0.002)		-0.0298*** (0.003)		-0.00606** (0.002)		-0.00371 (0.002)
Ln(BL tetanus prevalence (% of population, under 5))		0.0149*** (0.002)		0.0112*** (0.002)		0.0126*** (0.002)		0.00505* (0.002)		0.0138*** (0.003)		0.0104*** (0.002)
Ln(BL pertussis (whooping cough) prevalence (% of population, under 5))		-0.144*** (0.008)		-0.134*** (0.010)		-0.100*** (0.006)		-0.139*** (0.010)		-0.105*** (0.007)		-0.0825*** (0.006)
Ln(BL life expectancy at birth)		0.0155 (0.045)		-0.196*** (0.049)		0.773*** (0.087)		-0.107* (0.050)		0.798*** (0.097)		0.922*** (0.109)
Ln(BL Urban population (%))		0.00706 (0.012)		-0.00672 (0.015)		0.126*** (0.015)		0.00650 (0.014)		0.0804*** (0.012)		0.111*** (0.017)
Ln(BL School enrollment, secondary (% gross))		0.128*** (0.017)		0.0640** (0.023)		0.140*** (0.019)		0.0783*** (0.022)		0.122*** (0.023)		0.155*** (0.021)
		-0.166***		-0.256***		-0.104***		-0.229***		-0.0793***		-0.148***

Ln(BL Fertility rate (births per woman))		(0.016)		(0.023)		(0.015)		(0.022)		(0.017)		(0.017)
Recipient of US HIV aid before 2004 (=1 if yes)		0.0113<br>(0.011)		-0.0608***<br>(0.015)		0.0271**<br>(0.010)		-0.0567**<br>(0.018)		-0.0353**<br>(0.013)		0.0175<br>(0.012)
Constant	4.407***<br>(0.033)	3.338***<br>(0.203)	4.438***<br>(0.032)	4.861***<br>(0.233)	4.411***<br>(0.038)	0.403<br>(0.337)	4.445***<br>(0.032)	4.353***<br>(0.249)	4.403***<br>(0.040)	0.157<br>(0.427)	4.435***<br>(0.038)	0.0783<br>(0.417)
Adjusted R-squared	0.127	0.515	0.132	0.496	0.122	0.574	0.122	0.486	0.070	0.497	0.145	0.614
Observations	4282	3540	2628	2024	3386	2728	2592	1986	2565	1992	2589	1986

Notes: Standard errors in parentheses *** p<0.001, ** p<0.01, * p<0.05 BL=baseline; COP=country operational plans; PEPFAR= President's Emergency Plan for AIDS Relief

Table S6-2. Full model results for the indicator "Percentage of children age 12-23 months who received hepatitis B vaccines, 3 doses) "

Variables	All PEPFAR countries		COP countries		Non-COP PEPFAR countries		High-intensity PEPFAR countries		Medium-intensity PEPFAR countries		Low-intensity PEPFAR countries	
	Model 1	Model 2	Model 1	Model 2	Model 1	Model 2	Model 1	Model 2	Model 1	Model 2	Model 1	Model 2
Intervention (=1 if PEPFAR)	-0.271***<br>(0.051)	-0.232***<br>(0.050)	-0.549***<br>(0.107)	-0.478***<br>(0.095)	-0.185**<br>(0.056)	-0.175**<br>(0.057)	-0.397**<br>(0.148)	-0.239<br>(0.134)	-0.236***<br>(0.070)	-0.197**<br>(0.068)	-0.271***<br>(0.077)	-0.163*<br>(0.078)
Treatment (=1 for the years PEPFAR starts and after)	0.173***<br>(0.051)	0.252***<br>(0.052)	0.404***<br>(0.108)	0.446***<br>(0.105)	0.119*<br>(0.056)	0.200***<br>(0.059)	0.313*<br>(0.148)	0.353*<br>(0.147)	0.134<br>(0.072)	0.191**<br>(0.069)	0.172*<br>(0.079)	0.255**<br>(0.080)
Country income level (=1 if middle income)		0.0404<br>(0.027)		0.0882*<br>(0.039)		-0.0382<br>(0.030)		0.0202<br>(0.036)		-0.0187<br>(0.037)		0.0383<br>(0.050)
Ln(BL Population)		-0.0186**<br>(0.007)		-0.0275**<br>(0.009)		-0.0124<br>(0.007)		-0.00579<br>(0.009)		-0.0130<br>(0.008)		-0.0212*<br>(0.009)
Ln(BL other donor health spending per capita)		0.0205*<br>(0.009)		0.000936<br>(0.010)		0.0194*<br>(0.009)		0.0131<br>(0.011)		0.0120<br>(0.009)		0.0123<br>(0.010)
Ln(BL domestic health spending per capita)		-0.0709**<br>(0.022)		-0.0679*<br>(0.028)		-0.102***<br>(0.029)		-0.0991***<br>(0.028)		-0.142***<br>(0.031)		-0.0882*<br>(0.035)
Ln(BL GDP per capita)		0.0297<br>(0.031)		0.00630<br>(0.052)		0.0547<br>(0.033)		0.0533<br>(0.048)		0.0596<br>(0.038)		0.0291<br>(0.044)
Ln(BL HIV prevalence (% of population ages 15-49))		0.0123***<br>(0.003)		0.0244***<br>(0.006)		0.00260<br>(0.003)		0.00930<br>(0.005)		0.00279<br>(0.003)		0.0103*<br>(0.005)
Ln(BL hepatitis B prevalence (% of population, under 5))		-0.0682***<br>(0.008)		-0.0597***<br>(0.013)		-0.0529***<br>(0.010)		-0.0580***<br>(0.012)		-0.0590***<br>(0.012)		-0.0420**<br>(0.013)
Ln(BL life expectancy at birth)		0.161<br>(0.104)		0.0731<br>(0.141)		0.586***<br>(0.157)		0.188<br>(0.112)		0.381<br>(0.204)		0.917***<br>(0.195)
Ln(BL Urban population (%))		0.0144<br>(0.024)		-0.0394<br>(0.027)		0.0746*<br>(0.035)		-0.0548*<br>(0.027)		0.111**<br>(0.034)		0.0263<br>(0.041)
Ln(BL School enrollment, secondary (% gross))		0.0375<br>(0.028)		0.0433<br>(0.040)		0.0554<br>(0.032)		0.0500<br>(0.037)		0.142**<br>(0.045)		0.0801*<br>(0.040)
Ln(BL Fertility rate (births per woman))		-0.112**<br>(0.035)		-0.168**<br>(0.051)		-0.0944*<br>(0.037)		-0.216***<br>(0.053)		-0.0911<br>(0.050)		-0.120**<br>(0.043)
Recipient of US HIV aid before 2004 (=1 if yes)		-0.0354<br>(0.023)		0.0234<br>(0.037)		-0.0478<br>(0.027)		-0.0548<br>(0.036)		-0.000819<br>(0.035)		-0.127**<br>(0.047)
Constant	3.814***<br>(0.327)	3.186***<br>(0.613)	3.860***<br>(0.318)	4.216***<br>(0.799)	3.923***<br>(0.373)	1.139<br>(0.787)	3.923***<br>(0.374)	3.353***<br>(0.756)	3.808***<br>(0.330)	1.553<br>(0.998)	3.923***<br>(0.374)	0.128<br>(0.890)
Adjusted R-squared	0.149	0.236	0.192	0.277	0.135	0.214	0.154	0.245	0.167	0.246	0.140	0.251
Observations	2799	2304	1789	1379	2270	1809	1744	1341	1809	1399	1766	1332

Notes: Standard errors in parentheses *** p<0.001, ** p<0.01, * p<0.05 BL=baseline; COP=country operational plans; PEPFAR= President's Emergency Plan for AIDS Relief

Table S6-3. Full model results for the indicator "Percent of children ages 12-23 months who received the measles vaccination"

Variables	All PEPFAR countries		COP countries		Non-COP PEPFAR countries		High-intensity PEPFAR countries		Medium-intensity PEPFAR countries		Low-intensity PEPFAR countries	
	Model 1	Model 2	Model 1	Model 2	Model 1	Model 2	Model 1	Model 2	Model 1	Model 2	Model 1	Model 2
Intervention (=1 if PEPFAR)	-0.214***	-0.117***	-0.237***	-0.161***	-0.212***	-0.0908***	-0.223***	-0.101***	-0.159***	-0.0873***	-0.270***	-0.0914***
	(0.013)	(0.012)	(0.020)	(0.024)	(0.015)	(0.012)	(0.019)	(0.023)	(0.018)	(0.017)	(0.021)	(0.014)
Treatment (=1 for the years PEPFAR starts and after)	0.119***	0.104***	0.0970***	0.111***	0.151***	0.104***	0.0972***	0.0778***	0.0629**	0.109***	0.224***	0.135***
	(0.015)	(0.013)	(0.023)	(0.020)	(0.017)	(0.013)	(0.022)	(0.020)	(0.021)	(0.019)	(0.023)	(0.017)
Country income level (=1 if middle income)		-0.0239		-0.0130		-0.0490***		-0.00184		-0.0850***		-0.0407*
		(0.013)		(0.017)		(0.013)		(0.018)		(0.017)		(0.017)
Ln(BL Population)		-0.00723**		-0.0131***		-0.00989***		-0.00986***		-0.00599*		-0.0141***
		(0.002)		(0.003)		(0.002)		(0.003)		(0.003)		(0.002)
Ln(BL other donor health spending per capita)		0.0156***		0.00277		0.00354		0.00256		0.00750*		0.00116
		(0.003)		(0.004)		(0.003)		(0.004)		(0.003)		(0.003)
Ln(BL domestic health spending per capita)		0.0188		0.0311*		-0.0145		0.00652		0.0153		-0.0205
		(0.012)		(0.014)		(0.013)		(0.014)		(0.014)		(0.014)
Ln(BL GDP per capita)		-0.0144		-0.0260		-0.0217		-0.0254		-0.00971		-0.00611
		(0.013)		(0.019)		(0.014)		(0.017)		(0.014)		(0.017)
Ln(BL HIV prevalence (% of population ages 15-49))		0.0115***		0.0147***		0.00345**		0.0127***		0.00464***		0.00548***
		(0.001)		(0.002)		(0.001)		(0.002)		(0.001)		(0.001)
Ln(BL measles prevalence (% of population, under 5))		-0.00107		0.0000214		0.00118		-0.00134		0.00156		0.00442***
		(0.001)		(0.001)		(0.001)		(0.001)		(0.001)		(0.001)
Ln(BL life expectancy at birth)		0.239***		0.0412		0.828***		0.122*		0.783***		0.940***
		(0.047)		(0.056)		(0.078)		(0.050)		(0.092)		(0.091)
Ln(BL Urban population (%))		-0.0360**		-0.0698***		0.0702***		-0.0518***		0.0340**		0.0418**
		(0.012)		(0.013)		(0.013)		(0.011)		(0.013)		(0.014)
Ln(BL School enrollment, secondary (% gross))		0.117***		0.0396*		0.136***		0.0638***		0.129***		0.131***
		(0.014)		(0.020)		(0.015)		(0.018)		(0.017)		(0.017)
Ln(BL Fertility rate (births per woman))		-0.217***		-0.287***		-0.109***		-0.246***		-0.0981***		-0.136***
		(0.016)		(0.024)		(0.016)		(0.023)		(0.020)		(0.017)
Recipient of US HIV aid before 2004 (=1 if yes)		0.0455***		0.0209		0.0463***		0.0161		0.0145		0.0317**
		(0.010)		(0.015)		(0.010)		(0.015)		(0.014)		(0.011)
Constant	4.384***	3.409***	4.390***	4.919***	4.392***	0.601	4.400***	4.421***	4.382***	0.661	4.394***	0.267
	(0.028)	(0.201)	(0.028)	(0.243)	(0.031)	(0.314)	(0.029)	(0.221)	(0.030)	(0.412)	(0.034)	(0.362)
Adjusted R-squared	0.140	0.452	0.156	0.392	0.129	0.558	0.143	0.375	0.093	0.450	0.153	0.615
Observations	4275	3540	2621	2024	3379	2728	2585	1986	2558	1992	2582	1986

Notes: Standard errors in parentheses *** p<0.001, ** p<0.01, * p<0.05 BL=baseline; COP=country operational plans; PEPFAR= President's Emergency Plan for AIDS Relief

Table S6-4. Full model results for the indicator "Percentage of births by women of child-bearing age who are immunized against tetanus"

Variables	All PEPFAR countries		COP countries		Non-COP PEPFAR countries		High-intensity PEPFAR countries		Medium-intensity PEPFAR countries		Low-intensity PEPFAR countries	
	Model 1	Model 2	Model 1	Model 2	Model 1	Model 2	Model 1	Model 2	Model 1	Model 2	Model 1	Model 2
Intervention (=1 if PEPFAR)	-0.102*** (0.021)	-0.0190 (0.022)	-0.114*** (0.030)	-0.117** (0.037)	-0.104*** (0.023)	0.000771 (0.024)	-0.126*** (0.029)	-0.103* (0.040)	-0.0804** (0.031)	-0.0426 (0.031)	-0.115*** (0.027)	0.0756** (0.027)
Treatment (=1 for the years PEPFAR starts and after)	0.0947*** (0.019)	0.0697*** (0.019)	0.107*** (0.030)	0.0813** (0.030)	0.103*** (0.022)	0.0729*** (0.022)	0.125*** (0.029)	0.0993** (0.031)	0.0874** (0.032)	0.0947** (0.031)	0.104*** (0.027)	0.0190 (0.024)
Country income level (=1 if middle income)		-0.00576 (0.019)		0.00662 (0.024)		-0.0453 (0.027)		0.0386 (0.025)		-0.00149 (0.033)		-0.0993** (0.031)
Ln(BL Population)		-0.0234*** (0.005)		-0.0267*** (0.006)		-0.0235*** (0.006)		-0.0297*** (0.007)		-0.0351*** (0.007)		-0.0174** (0.006)
Ln(BL other donor health spending per capita)		0.00299 (0.006)		-0.00627 (0.007)		0.00661 (0.007)		-0.000983 (0.008)		-0.00484 (0.008)		0.0176* (0.007)
Ln(BL domestic health spending per capita)		-0.0432** (0.015)		-0.0882*** (0.022)		-0.0264 (0.020)		-0.0229 (0.027)		-0.0154 (0.024)		-0.0821*** (0.022)
Ln(BL GDP per capita)		0.0579*** (0.017)		0.137*** (0.024)		0.0581*** (0.017)		0.0651* (0.028)		-0.000499 (0.021)		0.199*** (0.022)
Ln(BL HIV prevalence (% of population ages 15-49))		-0.000730 (0.002)		-0.00498 (0.003)		0.00161 (0.003)		0.00265 (0.004)		0.000212 (0.003)		-0.000435 (0.003)
Ln(BL life expectancy at birth)		-0.0252 (0.070)		-0.144 (0.106)		-0.0140 (0.116)		0.0601 (0.090)		-0.379** (0.129)		0.132 (0.150)
Ln(BL Urban population (%))		-0.0248 (0.016)		-0.116*** (0.021)		-0.00817 (0.024)		-0.107*** (0.019)		0.0486* (0.025)		-0.166*** (0.026)
Ln(BL School enrollment, secondary (% gross))		-0.0284 (0.017)		-0.0884*** (0.024)		-0.00440 (0.025)		-0.0815** (0.026)		-0.00713 (0.040)		-0.0258 (0.030)
Ln(BL Fertility rate (births per woman))		-0.264*** (0.031)		-0.313*** (0.044)		-0.293*** (0.036)		-0.203*** (0.049)		-0.343*** (0.042)		-0.400*** (0.041)
Recipient of US HIV aid before 2004 (=1 if yes)		0.0499** (0.016)		0.177*** (0.027)		0.00608 (0.017)		0.124*** (0.026)		0.0331 (0.024)		0.0544* (0.022)
Constant	3.946*** (0.062)	4.614*** (0.358)	3.985*** (0.082)	5.371*** (0.530)	3.937*** (0.077)	4.368*** (0.506)	3.965*** (0.083)	4.615*** (0.475)	3.979*** (0.092)	6.420*** (0.591)	3.955*** (0.093)	3.558*** (0.622)
Adjusted R-squared	0.284	0.359	0.250	0.334	0.270	0.376	0.273	0.345	0.226	0.313	0.232	0.434
Observations	2830	2657	1624	1480	1964	1848	1614	1441	1336	1249	1396	1309

Notes: Standard errors in parentheses *** p<0.001, ** p<0.01, * p<0.05 BL=baseline; COP=country operational plans; PEPFAR= President's Emergency Plan for AIDS Relief

Table S6-5. Full model results for the indicator "Number of women who die from pregnancy-related causes while pregnant or within 42 days of pregnancy termination per 100,000 live births"

Variables	All PEPFAR countries		COP countries		Non-COP PEPFAR countries		High-intensity PEPFAR countries		Medium-intensity PEPFAR countries		Low-intensity PEPFAR countries	
	Model 1	Model 2	Model 1	Model 2	Model 1	Model 2	Model 1	Model 2	Model 1	Model 2	Model 1	Model 2
Intervention (=1 if PEPFAR)	1.503*** (0.089)	0.113** (0.040)	1.984*** (0.126)	0.424*** (0.079)	1.439*** (0.099)	0.0248 (0.042)	2.163*** (0.121)	0.309*** (0.071)	0.862*** (0.123)	-0.0240 (0.048)	1.822*** (0.118)	0.115 (0.062)
Treatment (=1 for the years PEPFAR starts and after)	-0.0406 (0.094)	0.0594 (0.040)	0.0448 (0.130)	0.0214 (0.061)	-0.347** (0.110)	0.0696 (0.046)	-0.0987 (0.126)	0.00106 (0.057)	0.383** (0.139)	-0.0257 (0.057)	-0.881*** (0.134)	0.0251 (0.064)
Country income level (=1 if middle income)		-0.186*** (0.039)		-0.174*** (0.051)		-0.0830 (0.045)		-0.295*** (0.046)		-0.0140 (0.048)		-0.140* (0.062)
Ln(BL Population)		-0.00770 (0.010)		-0.0186 (0.012)		-0.0440*** (0.010)		-0.0513*** (0.012)		-0.0938*** (0.010)		-0.0403** (0.013)
Ln(BL other donor health spending per capita)		-0.0204 (0.014)		-0.0162 (0.017)		-0.0393** (0.015)		-0.0275 (0.018)		-0.0893*** (0.014)		-0.0351* (0.018)
Ln(BL domestic health spending per capita)		-0.202*** (0.028)		-0.149*** (0.041)		-0.242*** (0.034)		-0.0176 (0.045)		-0.307*** (0.034)		-0.316*** (0.046)
Ln(BL GDP per capita)		0.0988*** (0.029)		0.0176 (0.046)		0.0560 (0.031)		-0.0985* (0.044)		0.0430 (0.035)		0.0284 (0.039)
Ln(BL HIV prevalence (% of population ages 15-49))		0.0385*** (0.004)		0.0153* (0.006)		0.0470*** (0.004)		0.0331*** (0.006)		0.0366*** (0.005)		0.0338*** (0.005)
Ln(BL life expectancy at birth)		-2.337*** (0.101)		-1.985*** (0.151)		-2.156*** (0.192)		-1.969*** (0.121)		-2.667*** (0.208)		-0.839** (0.257)
Ln(BL Urban population (%))		0.0611* (0.028)		0.0171 (0.034)		0.124** (0.040)		0.0255 (0.033)		0.134** (0.041)		0.129* (0.051)
Ln(BL School enrollment, secondary (% gross))		-0.237*** (0.043)		-0.141** (0.050)		-0.507*** (0.053)		-0.141** (0.049)		-0.983*** (0.066)		-0.463*** (0.056)
Ln(BL Fertility rate (births per woman))		1.035*** (0.054)		0.855*** (0.093)		0.952*** (0.058)		1.007*** (0.093)		0.491*** (0.068)		1.100*** (0.079)
Recipient of US HIV aid before 2004 (=1 if yes)		-0.0854** (0.033)		-0.0120 (0.044)		-0.0328 (0.038)		-0.0616 (0.043)		0.0356 (0.041)		0.120 (0.063)
Constant	4.049*** (0.110)	14.84*** (0.514)	4.073*** (0.123)	13.74*** (0.833)	3.940*** (0.121)	16.16*** (0.793)	4.024*** (0.122)	14.42*** (0.712)	4.176*** (0.131)	21.91*** (0.951)	3.846*** (0.133)	10.80*** (1.010)
Adjusted R-squared	0.265	0.829	0.478	0.827	0.192	0.823	0.492	0.842	0.185	0.815	0.201	0.790
Observations	2628	2178	1566	1206	2070	1674	1548	1188	1548	1206	1548	1188

Notes: Standard errors in parentheses *** p<0.001, ** p<0.01, * p<0.05 BL=baseline; COP=country operational plans; PEPFAR= President's Emergency Plan for AIDS Relief

f

Table S6-6. Full model results for the indicator "Probability of a child dying between birth and 5 years of age, per 1,000 live births"

Variables	All PEPFAR countries		COP countries		Non-COP PEPFAR countries		High-intensity PEPFAR countries		Medium-intensity PEPFAR countries		Low-intensity PEPFAR countries	
	Model 1	Model 2	Model 1	Model 2	Model 1	Model 2	Model 1	Model 2	Model 1	Model 2	Model 1	Model 2
Intervention (=1 if PEPFAR)	1.001*** (0.032)	0.141*** (0.020)	1.294*** (0.038)	0.182*** (0.041)	0.892*** (0.036)	0.0607** (0.020)	1.337*** (0.038)	0.0846* (0.040)	0.734*** (0.046)	0.0357 (0.022)	1.008*** (0.044)	-0.0136 (0.026)
Treatment (=1 for the years PEPFAR starts and after)	-0.0147 (0.043)	-0.0521** (0.019)	0.0326 (0.049)	-0.0394 (0.027)	-0.135** (0.050)	-0.0758*** (0.021)	-0.00670 (0.049)	-0.0620* (0.028)	0.103 (0.065)	-0.0907*** (0.024)	-0.306*** (0.061)	-0.0750** (0.025)
Country income level (=1 if middle income)		-0.207*** (0.018)		-0.0972** (0.031)		-0.243*** (0.023)		-0.0972** (0.030)		-0.141*** (0.027)		-0.328*** (0.034)
Ln(BL Population)		0.0167*** (0.004)		0.0165** (0.006)		0.0163** (0.005)		0.000543 (0.006)		-0.00483 (0.006)		0.0140* (0.006)
Ln(BL other donor health spending per capita)		-0.0174** (0.006)		0.000586 (0.008)		-0.0213** (0.007)		-0.0148 (0.008)		-0.0365*** (0.007)		-0.0119 (0.008)
Ln(BL domestic health spending per capita)		-0.117*** (0.013)		-0.139*** (0.025)		-0.0729*** (0.017)		-0.0886** (0.027)		-0.117*** (0.021)		-0.0843*** (0.022)
Ln(BL GDP per capita)		-0.0138 (0.019)		0.0263 (0.036)		-0.0468* (0.020)		-0.0571 (0.035)		-0.0124 (0.022)		-0.0490 (0.028)
Ln(BL HIV prevalence (% of population ages 15-49))		-0.0205*** (0.002)		-0.0314*** (0.004)		-0.0176*** (0.003)		-0.0229*** (0.004)		-0.0231*** (0.003)		-0.0190*** (0.003)
Ln(BL life expectancy at birth)		-2.378*** (0.068)		-2.436*** (0.106)		-3.072*** (0.109)		-2.527*** (0.096)		-3.698*** (0.131)		-2.968*** (0.181)
Ln(BL Urban population (%))		0.198*** (0.015)		0.204*** (0.020)		0.152*** (0.021)		0.238*** (0.021)		0.152*** (0.020)		0.193*** (0.026)
Ln(BL School enrollment, secondary (% gross))		-0.139*** (0.018)		-0.212*** (0.030)		-0.129*** (0.022)		-0.187*** (0.029)		-0.314*** (0.032)		-0.141*** (0.027)
Ln(BL Fertility rate (births per woman))		0.480*** (0.025)		0.367*** (0.047)		0.420*** (0.027)		0.450*** (0.048)		0.192*** (0.032)		0.451*** (0.039)
Recipient of US HIV aid before 2004 (=1 if yes)		0.0506*** (0.013)		0.103*** (0.023)		0.136*** (0.014)		0.139*** (0.026)		0.177*** (0.018)		0.280*** (0.027)
Constant	3.534*** (0.060)	13.83*** (0.329)	3.522*** (0.069)	14.08*** (0.539)	3.541*** (0.068)	17.10*** (0.449)	3.518*** (0.070)	14.87*** (0.515)	3.591*** (0.077)	20.93*** (0.546)	3.518*** (0.074)	16.70*** (0.723)
Adjusted R-squared	0.364	0.874	0.504	0.837	0.303	0.868	0.509	0.839	0.276	0.844	0.309	0.834
Observations	4408	3596	2697	2030	3509	2784	2668	2001	2668	2030	2668	2001

Notes: Standard errors in parentheses *** p<0.001, ** p<0.01, * p<0.05 BL=baseline; COP=country operational plans; PEPFAR= President's Emergency Plan for AIDS Relief

Table S6-7. Full model results for the indicator "Prevalence of anemia among women of reproductive age (% of women ages 15-49)"

Variables	All PEPFAR countries		COP countries		Non-COP PEPFAR countries		High-intensity PEPFAR countries		Medium-intensity PEPFAR countries		Low-intensity PEPFAR countries	
	**Model 1**	**Model 2**	**Model 1**	**Model 2**	**Model 1**	**Model 2**	**Model 1**	**Model 2**	**Model 1**	**Model 2**	**Model 1**	**Model 2**
Intervention (=1 if PEPFAR)	0.231*** (0.013)	0.0319* (0.014)	0.283*** (0.016)	0.102*** (0.028)	0.211*** (0.015)	-0.0194 (0.014)	0.278*** (0.016)	0.0839** (0.030)	0.177*** (0.019)	-0.0276 (0.016)	0.243*** (0.018)	-0.0258 (0.020)
Treatment (=1 for the years PEPFAR starts and after)	-0.0189 (0.018)	0.0130 (0.015)	-0.000761 (0.022)	0.0138 (0.022)	-0.0484* (0.022)	0.0200 (0.016)	-0.0211 (0.023)	-0.00408 (0.023)	0.0506 (0.029)	0.00721 (0.021)	-0.103*** (0.027)	0.0145 (0.019)
Country income level (=1 if middle income)		-0.258*** (0.019)		-0.202*** (0.026)		-0.233*** (0.024)		-0.242*** (0.026)		-0.124*** (0.027)		-0.264*** (0.032)
BL Population		-0.000988 (0.003)		-0.00222 (0.004)		0.00352 (0.003)		-0.00821* (0.004)		-0.0144*** (0.004)		0.00994** (0.004)
BL other donor health spending per capita		-0.0318*** (0.004)		-0.0244*** (0.005)		-0.0337*** (0.004)		-0.0216*** (0.005)		-0.0662*** (0.005)		-0.0288*** (0.005)
BL domestic health spending per capita		-0.0960*** (0.011)		-0.0535*** (0.015)		-0.0603*** (0.014)		-0.0166 (0.016)		-0.0231 (0.016)		-0.0761*** (0.015)
BL GDP per capita		0.143*** (0.012)		0.181*** (0.015)		0.129*** (0.011)		0.171*** (0.015)		0.0557*** (0.015)		0.156*** (0.011)
BL HIV prevalence (% of population ages 15-49)		-0.0198*** (0.002)		-0.0357*** (0.002)		-0.0141*** (0.002)		-0.0335*** (0.003)		-0.0229*** (0.002)		-0.0169*** (0.002)
BL life expectancy at birth		-0.583*** (0.047)		-0.237*** (0.065)		-1.235*** (0.075)		-0.283*** (0.061)		-1.370*** (0.102)		-0.961*** (0.084)
BL Urban population (%)		0.0726*** (0.013)		-0.0211 (0.018)		-0.0470** (0.017)		-0.0151 (0.020)		-0.0875*** (0.020)		-0.111*** (0.020)
BL School enrollment, secondary (% gross)		-0.0675*** (0.014)		-0.148*** (0.024)		-0.0664*** (0.016)		-0.173*** (0.023)		-0.302*** (0.024)		-0.0471* (0.021)
BL Fertility rate (births per woman)		0.244*** (0.019)		0.222*** (0.033)		0.169*** (0.021)		0.230*** (0.033)		0.0631* (0.025)		0.206*** (0.028)
Recipient of US HIV aid before 2004 (=1 if yes)		-0.0180 (0.012)		0.136*** (0.018)		-0.00555 (0.013)		0.121*** (0.021)		0.0361* (0.018)		0.0327 (0.021)
Constant	3.602*** (0.022)	5.334*** (0.226)	3.612*** (0.026)	3.911*** (0.336)	3.605*** (0.026)	8.508*** (0.319)	3.613*** (0.026)	4.190*** (0.331)	3.629*** (0.029)	10.94*** (0.436)	3.601*** (0.029)	7.234*** (0.349)
Adjusted R-squared	0.188	0.554	0.270	0.482	0.160	0.621	0.253	0.475	0.166	0.613	0.172	0.592
Observations	3996	3321	2403	1863	3159	2565	2376	1836	2376	1863	2376	1836

Notes: Standard errors in parentheses *** p<0.001, ** p<0.01, * p<0.05 BL=baseline; COP=country operational plans; PEPFAR= President's Emergency Plan for AIDS Relief

S7. Parallel pre-trend assumption test for traditional DID

S7-1. DPT immunization rate

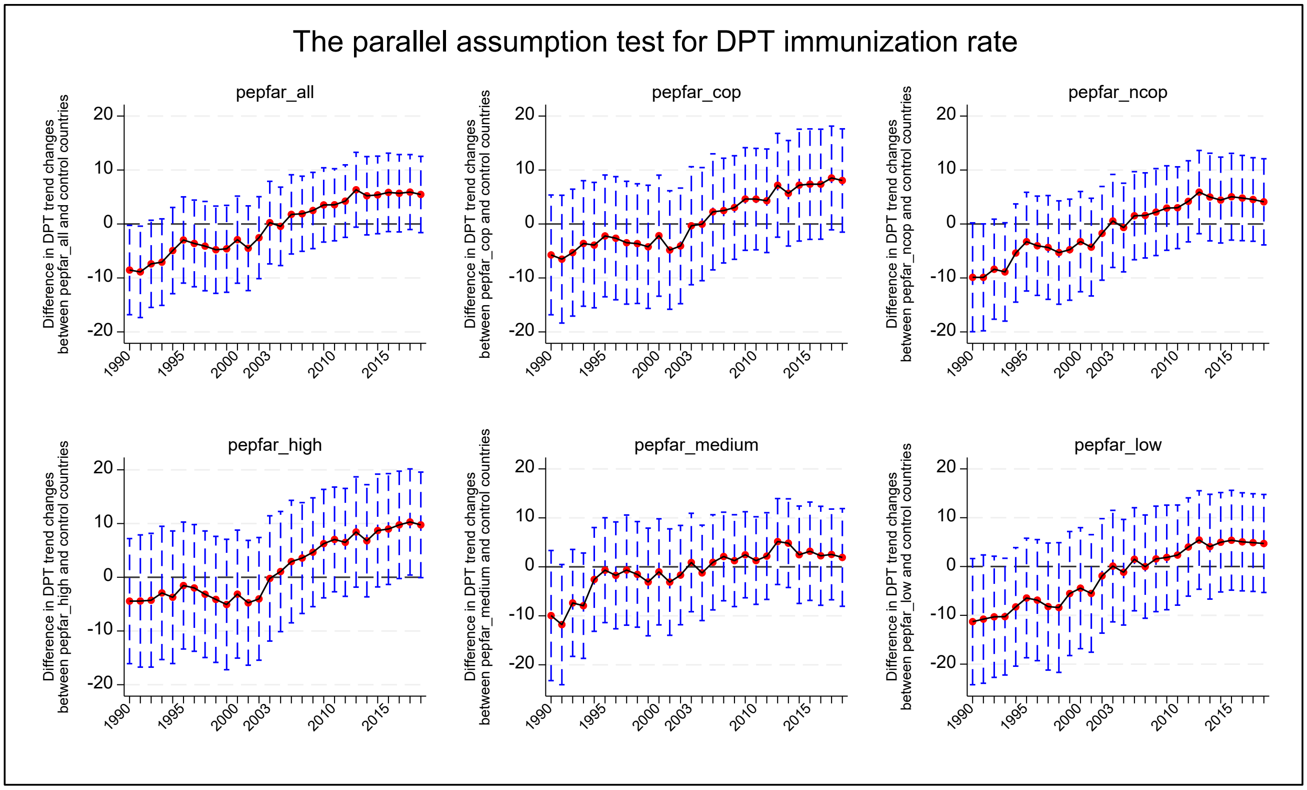

Notes: pepfar_all = all PEPFAR country cohort; pepfar_cop = COP PEPFAR country cohort; pepfar_ncop = non-COP PEPFAR country cohort; pepfar_high = high-intensity PEPFAR country cohort; pepfar_medium = medium-intensity PEPFAR country cohort; pepfar_low = low-intensity PEPFAR country cohort. COP= country operational plans; DPT=diphtheria, pertussis, tetanus; PEPFAR= President's Emergency Plan for AIDS Relief

S7-2. Hepatitis B immunization rate

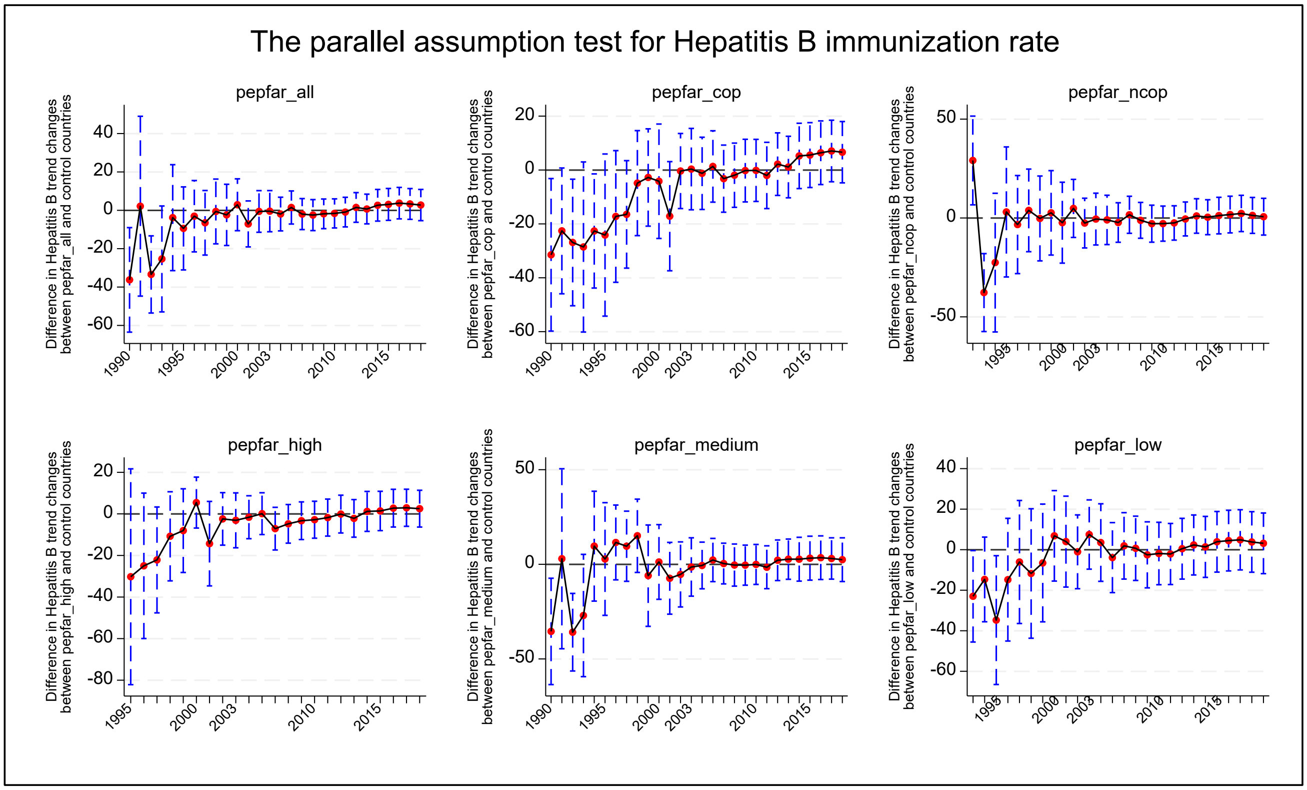

Notes: pepfar_all = all PEPFAR country cohort; pepfar_cop = COP PEPFAR country cohort; pepfar_ncop = non-COP PEPFAR country cohort; pepfar_high = high-intensity PEPFAR country cohort; pepfar_medium = medium-intensity PEPFAR country cohort; pepfar_low = low-intensity PEPFAR country cohort. COP= country operational plans; PEPFAR= President's Emergency Plan for AIDS Relief

S7-3. Measles immunization rate

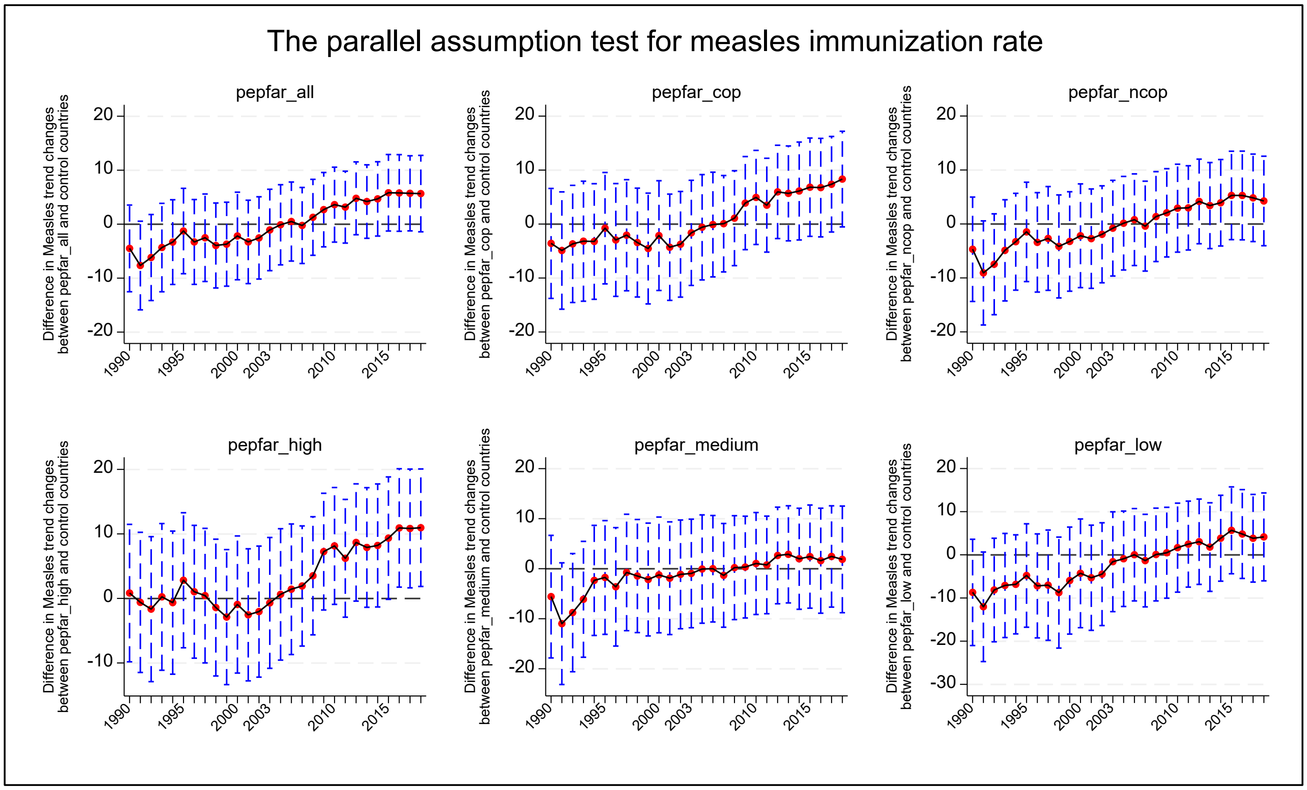

Notes: pepfar_all = all PEPFAR country cohort; pepfar_cop = COP PEPFAR country cohort; pepfar_ncop = non-COP PEPFAR country cohort; pepfar_high = high-intensity PEPFAR country cohort; pepfar_medium = medium-intensity PEPFAR country cohort; pepfar_low = low-intensity PEPFAR country cohort. COP= country operational plans; PEPFAR= President’s Emergency Plan for AIDS Relief

S7-4. Prevalence of newborns protected against tetanus

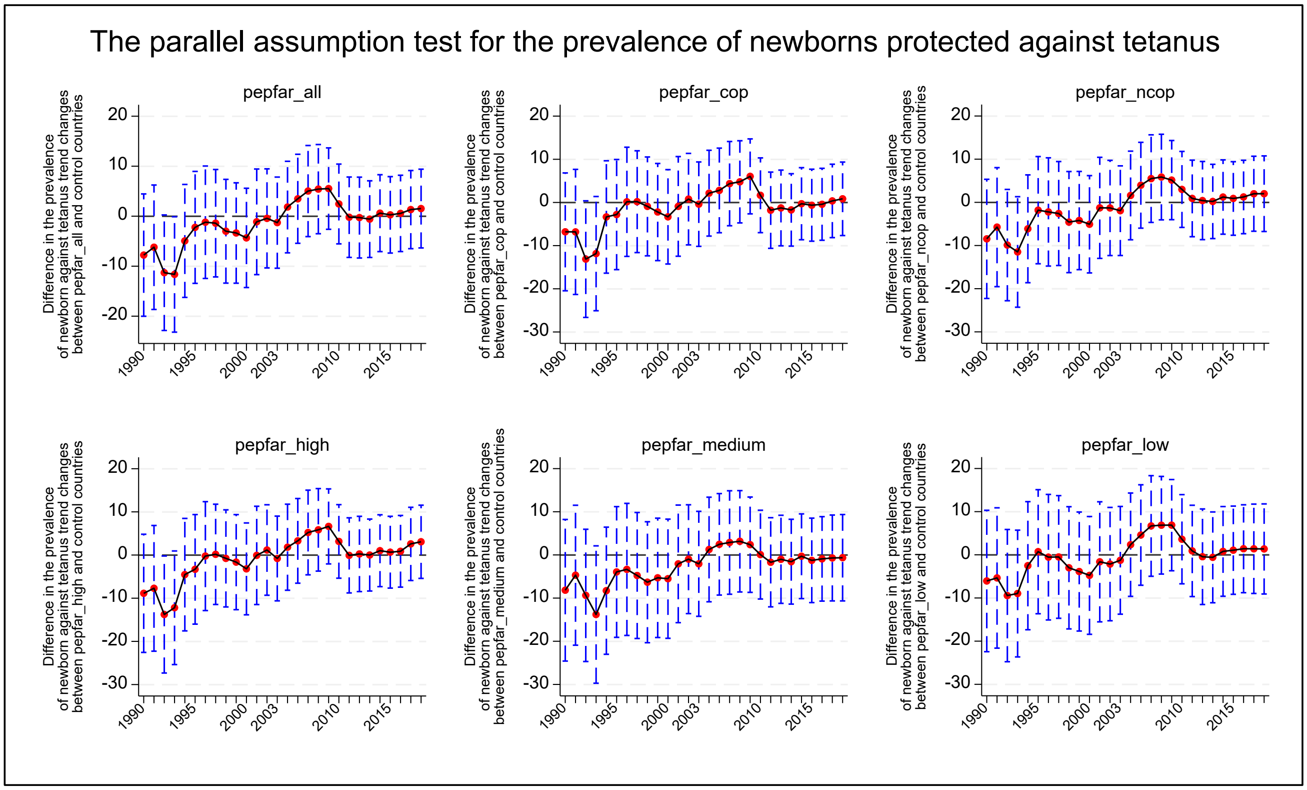

Notes: pepfar_all = all PEPFAR country cohort; pepfar_cop = COP PEPFAR country cohort; pepfar_ncop = non-COP PEPFAR country cohort; pepfar_high = high-intensity PEPFAR country cohort; pepfar_medium = medium-intensity PEPFAR country cohort; pepfar_low = low-intensity PEPFAR country cohort. COP= country operational plans; PEPFAR= President's Emergency Plan for AIDS Relief

S7-5. Maternal mortality ratio

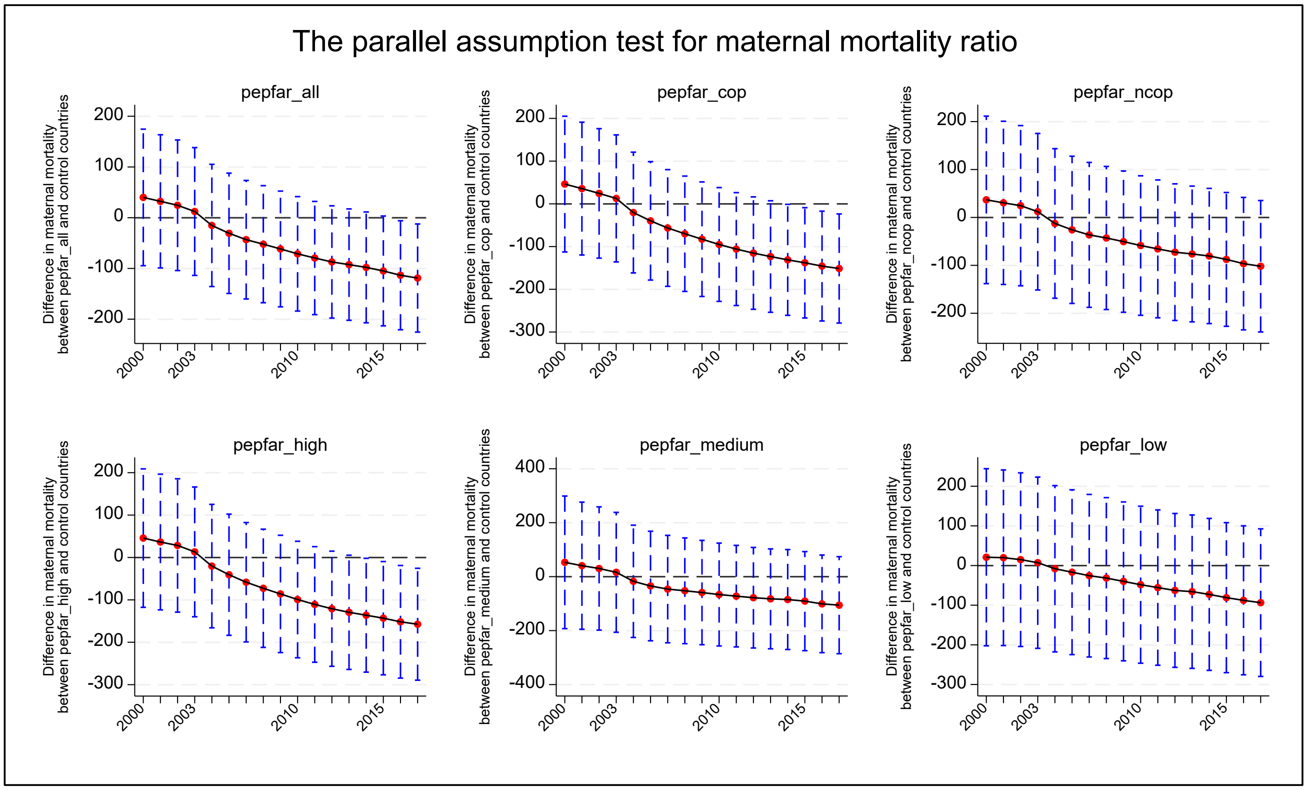

Notes: pepfar_all = all PEPFAR country cohort; pepfar_cop = COP PEPFAR country cohort; pepfar_ncop = non-COP PEPFAR country cohort; pepfar_high = high-intensity PEPFAR country cohort; pepfar_medium = medium-intensity PEPFAR country cohort; pepfar_low = low-intensity PEPFAR country cohort. COP= country operational plans; PEPFAR= President's Emergency Plan for AIDS Relief

S7-6. Child under-five mortality rate

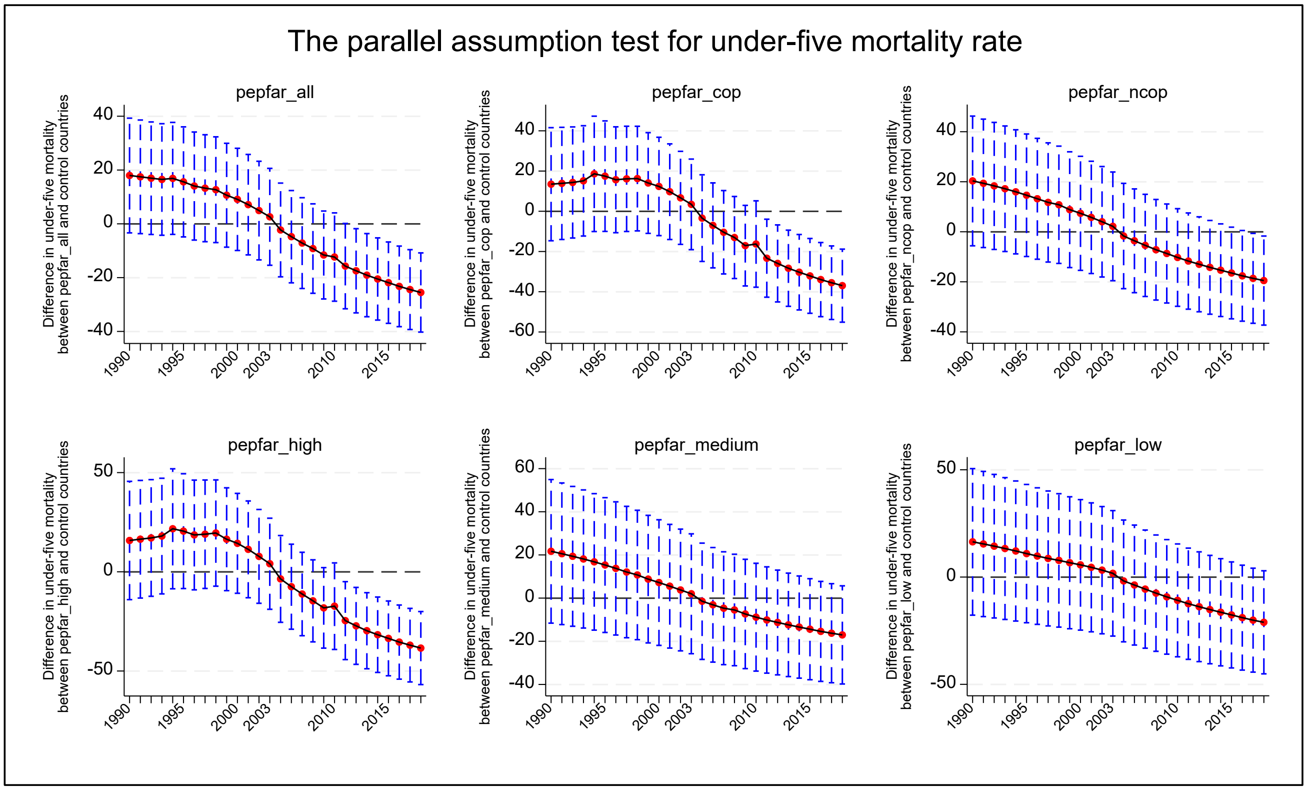

Notes: pepfar_all = all PEPFAR country cohort; pepfar_cop = COP PEPFAR country cohort; pepfar_ncop = non-COP PEPFAR country cohort; pepfar_high = high-intensity PEPFAR country cohort; pepfar_medium = medium-intensity PEPFAR country cohort; pepfar_low = low-intensity PEPFAR country cohort. COP= country operational plans; PEPFAR= President's Emergency Plan for AIDS Relief

S7-7. Prevalence of anemia among women of reproductive age

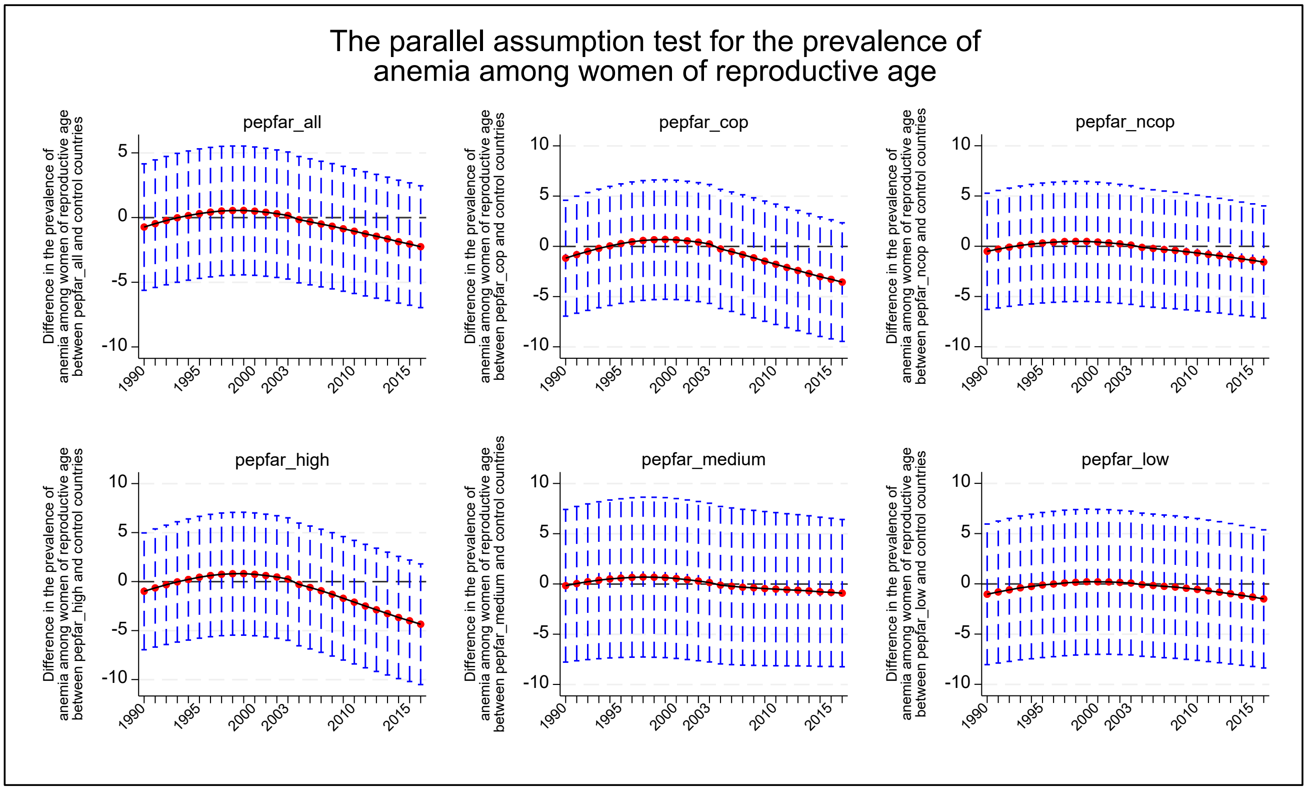

Notes: pepfar_all = all PEPFAR country cohort; pepfar_cop = COP PEPFAR country cohort; pepfar_ncop = non-COP PEPFAR country cohort; pepfar_high = high-intensity PEPFAR country cohort; pepfar_medium = medium-intensity PEPFAR country cohort; pepfar_low = low-intensity PEPFAR country cohort. COP= country operational plans; PEPFAR= President's Emergency Plan for AIDS Relief

S8. Parallel pre-trend assumption test for staggered DID

Figures in supplement S4 illustrate the dynamic impacts of PEPFAR as estimated by D_{it}^{T} from equation (4).

There are two key findings for the four immunization rates (see S4-1 to S4-4). First, improvements in hepatitis B immunization rate did not precede the PEPFAR program for all six country cohorts, as shown as D_{it}^{T} that are insignificantly different from zero (eg. without trends for all the years before PEPFAR started). A similar finding is also true for the COP and high-intensity country cohorts in DPT, measles, and tetanus immunization rates, but not for the other country cohorts of these three immunization rates. This verifies the internal validity of the staggered DID design for the six cohorts in hepatitis B immunization rate and for the COP and high-intensity country cohorts in DPT, measles, and tetanus immunization rates.

Second, for hepatitis B immunization rate, PEPFAR materialized its impact quickly in the COP country cohort, represented by the immediate jump of the 95% confidence interval that is right after the program started. Furthermore, PEPFAR gradually increased its magnitude of impact in the all PEPFAR country, COP country, and high-intensity country cohorts but leveled off or even diminished in the other three country cohorts. A similar impact pattern was observed for DPT and measles immunization rates, but not for the tetanus immunization rate.

Several patterns were observed for maternal and child mortality rate, as well as the prevalence of amenia among women of reproductive age. Although all the country cohorts have statistically insignificant D_{it}^{T}s prior to PEPFAR in terms of maternal mortality rate, D_{it}^{T}s exhibited some degree of declining trend before recipient countries joined the PEPFAR program (see S4-5). The existence of this preceding trend suggested limited internal validity for the staggered DID method in estimating the PEPFAR spillover effects on maternal mortality ratios. In terms of child mortality rate, a pattern of flat D_{it}^{T}s that are prior to PEPFAR started is evident for the COP and high-intensity country segments but not for other segments, demonstrating the good internal validity for the COP and high-intensity country cohorts but not for the other cohorts. Additionally, the impact of PEPFAR on child mortality rate becomes evident rapidly in COP and high-intensity countries, marked as an immediate decline for the 95% confidence intervals right after the cohorts started the PEPFAR program. No evident trend is observed before or after the start of the PEPFAR program for the prevalence of anemia among women of reproductive age, consistent with the inconclusive coefficients estimated by staggered DID.

S8-1. DPT immunization rate

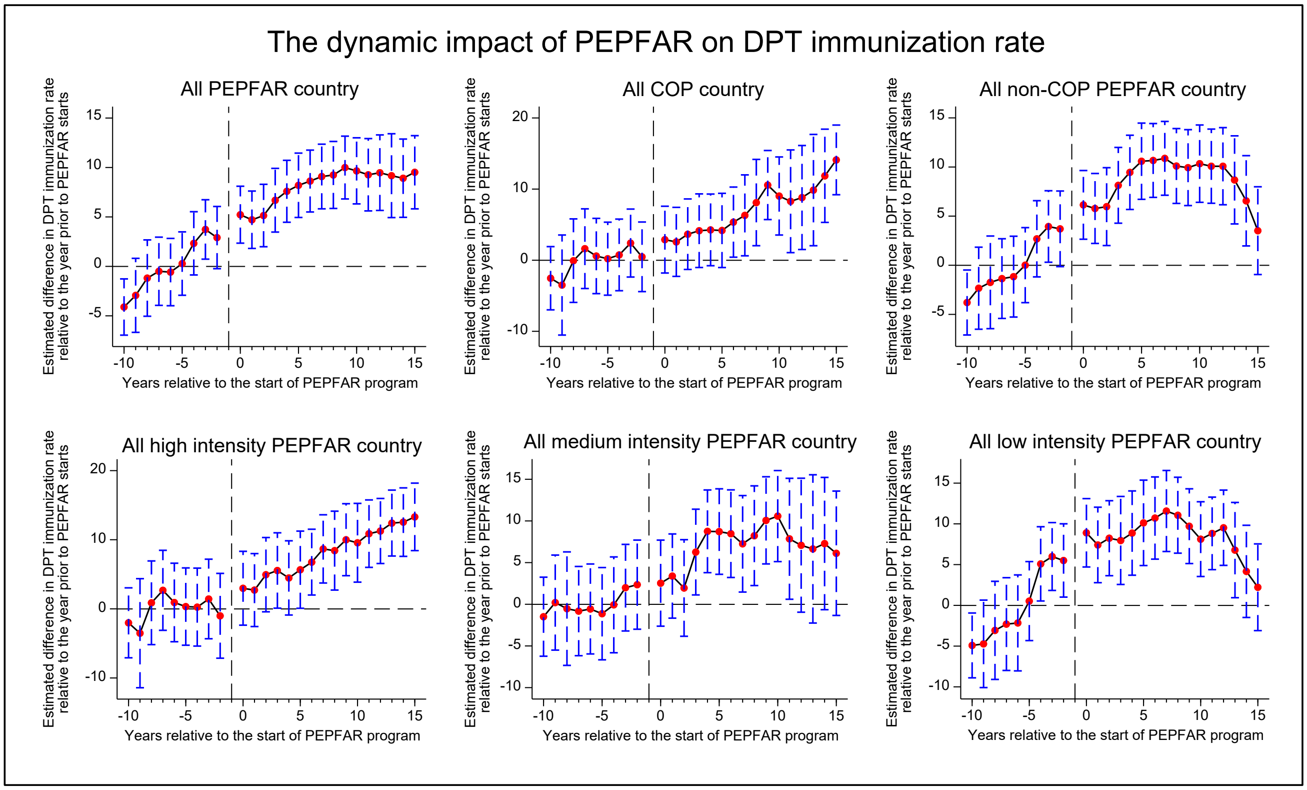

Notes: pepfar_all = all PEPFAR country cohort; pepfar_cop = COP PEPFAR country cohort; pepfar_ncop = non-COP PEPFAR country cohort; pepfar_high = high-intensity PEPFAR country cohort; pepfar_medium = medium-intensity PEPFAR country cohort; pepfar_low = low-intensity PEPFAR country cohort. COP= country operational plans; DPT=diphtheria, pertussis, tetanus; PEPFAR= President's Emergency Plan for AIDS Relief

S8-2. Hepatitis B immunization rate

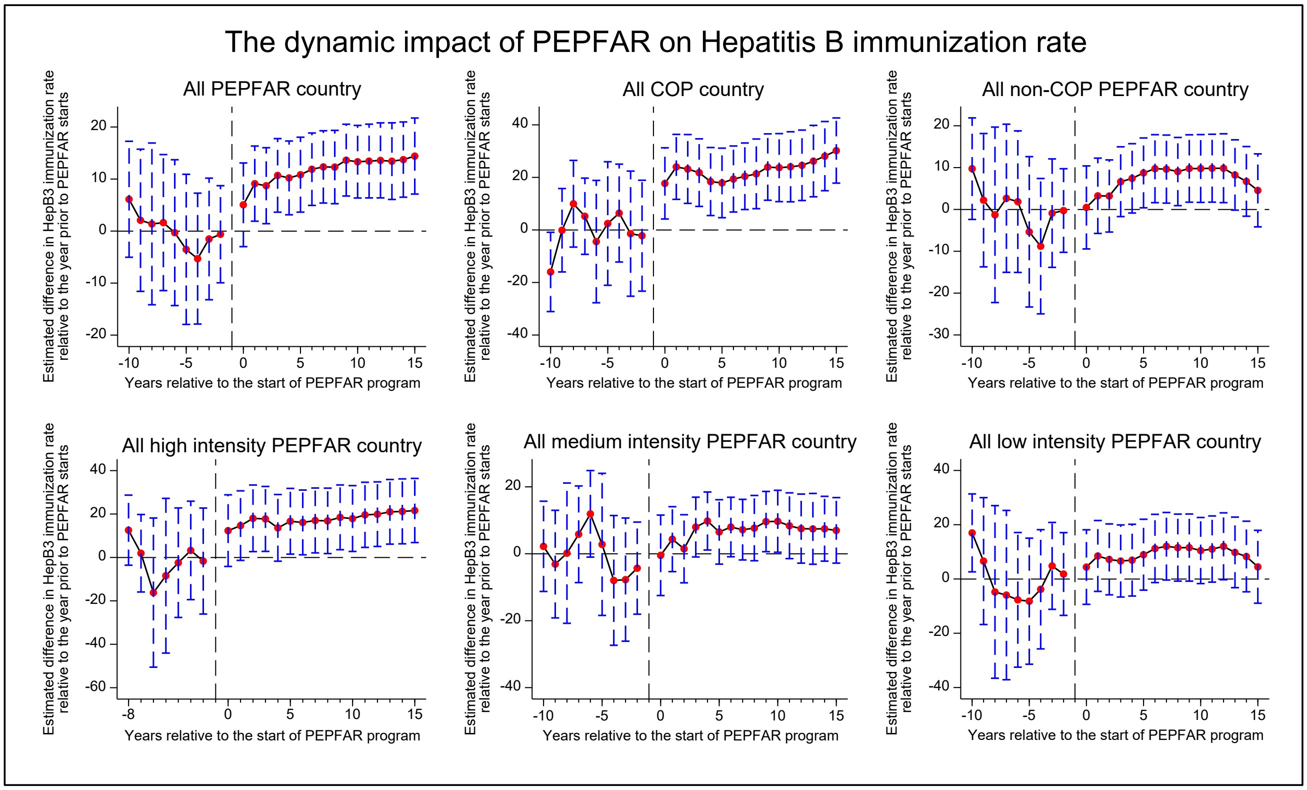

Notes: pepfar_all = all PEPFAR country cohort; pepfar_cop = COP PEPFAR country cohort; pepfar_ncop = non-COP PEPFAR country cohort; pepfar_high = high-intensity PEPFAR country cohort; pepfar_medium = medium-intensity PEPFAR country cohort; pepfar_low = low-intensity PEPFAR country cohort. COP= country operational plans; PEPFAR= President's Emergency Plan for AIDS Relief

S8-3. Measles immunization rate

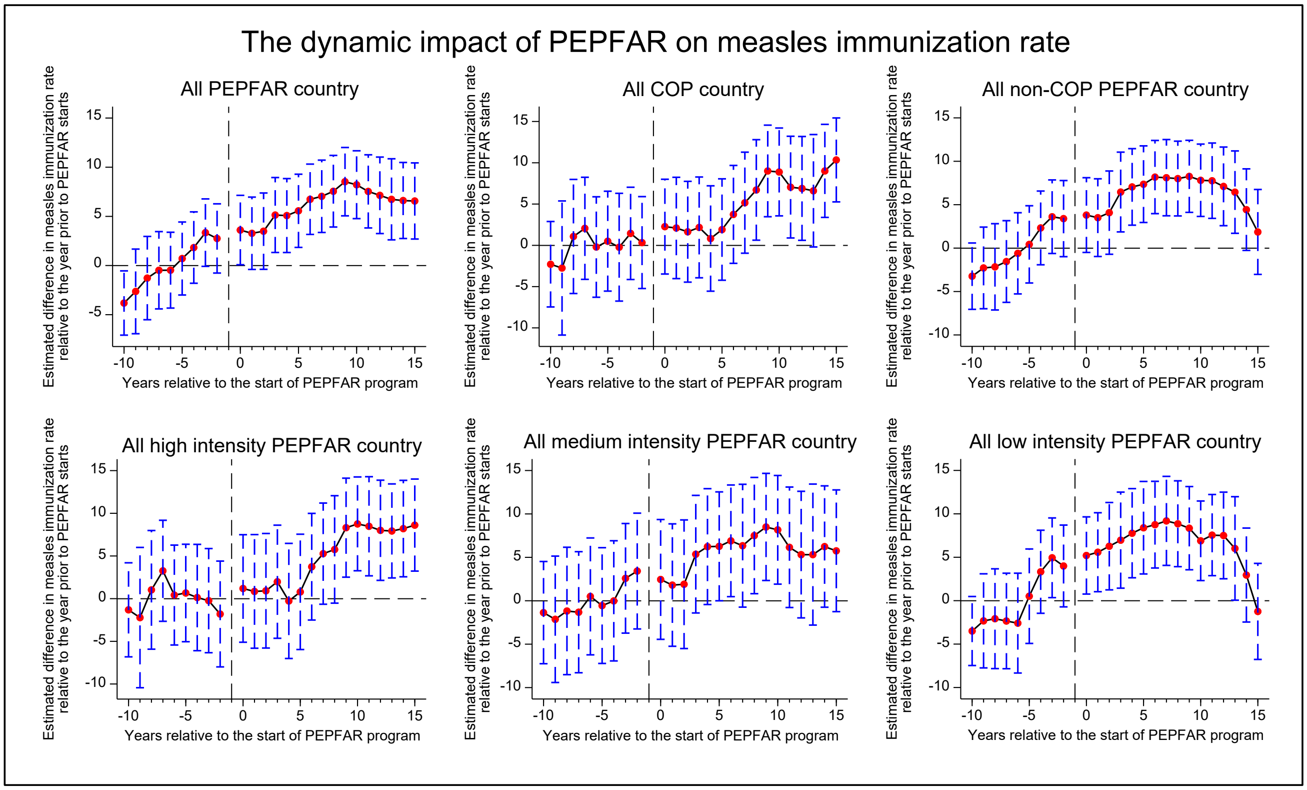

Notes: pepfar_all = all PEPFAR country cohort; pepfar_cop = COP PEPFAR country cohort; pepfar_ncop = non-COP PEPFAR country cohort; pepfar_high = high-intensity PEPFAR country cohort; pepfar_medium = medium-intensity PEPFAR country cohort; pepfar_low = low-intensity PEPFAR country cohort. COP= country operational plans; PEPFAR= President's Emergency Plan for AIDS Relief

S8-4. Prevalence of newborns against tetanus

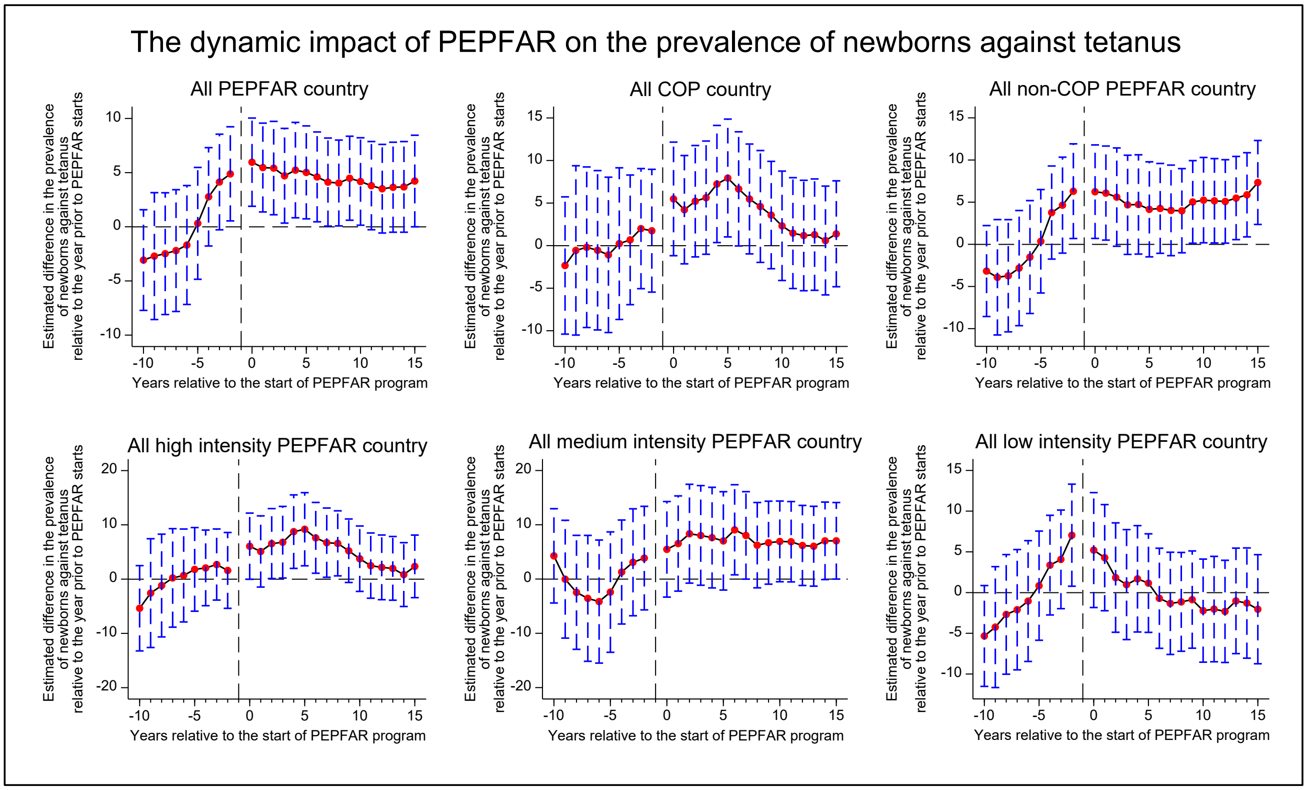

Notes: pepfar_all = all PEPFAR country cohort; pepfar_cop = COP PEPFAR country cohort; pepfar_ncop = non-COP PEPFAR country cohort; pepfar_high = high-intensity PEPFAR country cohort; pepfar_medium = medium-intensity PEPFAR country cohort; pepfar_low = low-intensity PEPFAR country cohort.   COP= country operational plans; PEPFAR= President's Emergency Plan for AIDS Relief

S8-5. Maternal mortality ratio

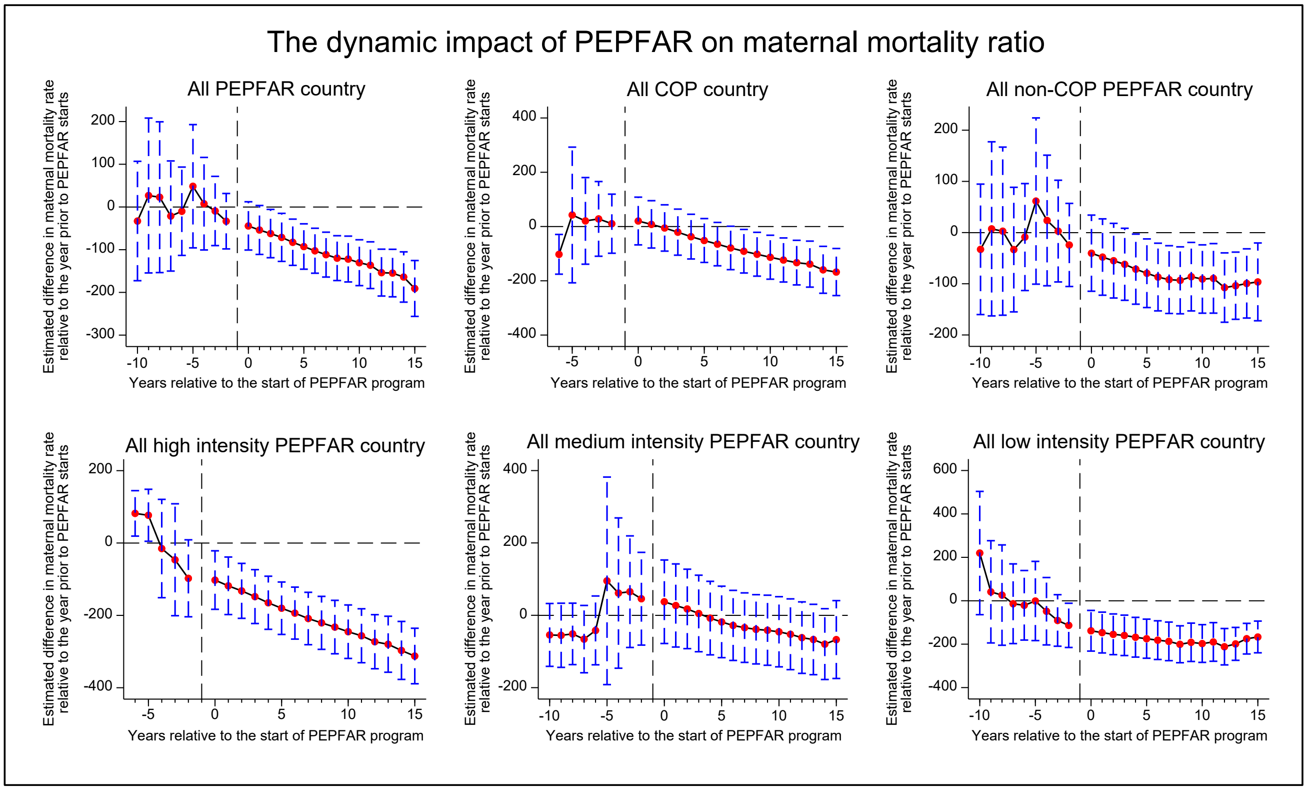

Notes: pepfar_all = all PEPFAR country cohort; pepfar_cop = COP PEPFAR country cohort; pepfar_ncop = non-COP PEPFAR country cohort; pepfar_high = high-intensity PEPFAR country cohort; pepfar_medium = medium-intensity PEPFAR country cohort; pepfar_low = low-intensity PEPFAR country cohort.   COP= country operational plans; PEPFAR= President's Emergency Plan for AIDS Relief

S8-6. Child (under-five) mortality rate

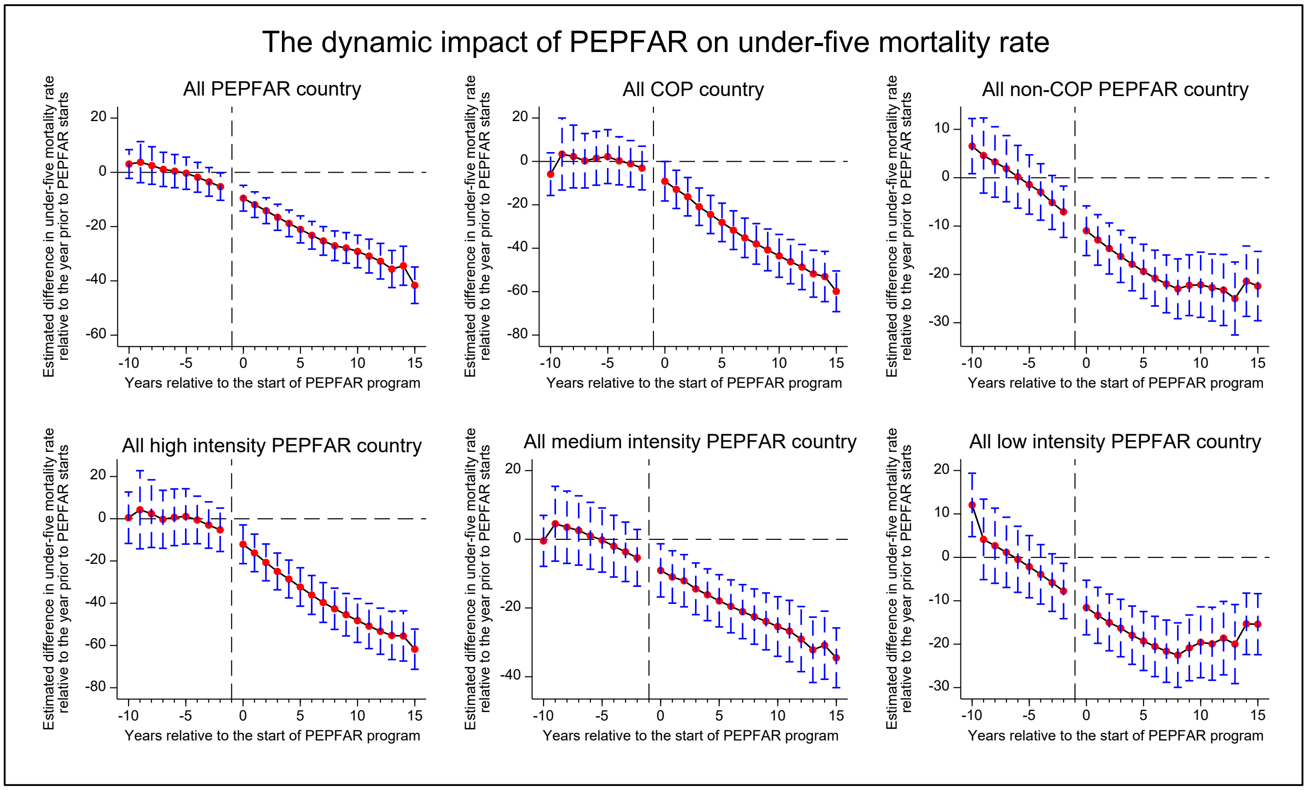

Notes: pepfar_all = all PEPFAR country cohort; pepfar_cop = COP PEPFAR country cohort; pepfar_ncop = non-COP PEPFAR country cohort; pepfar_high = high-intensity PEPFAR country cohort; pepfar_medium = medium-intensity PEPFAR country cohort; pepfar_low = low-intensity PEPFAR country cohort. COP= country operational plans; PEPFAR= President's Emergency Plan for AIDS Relief

S8-7. Prevalence of anemia among women of reproductive age

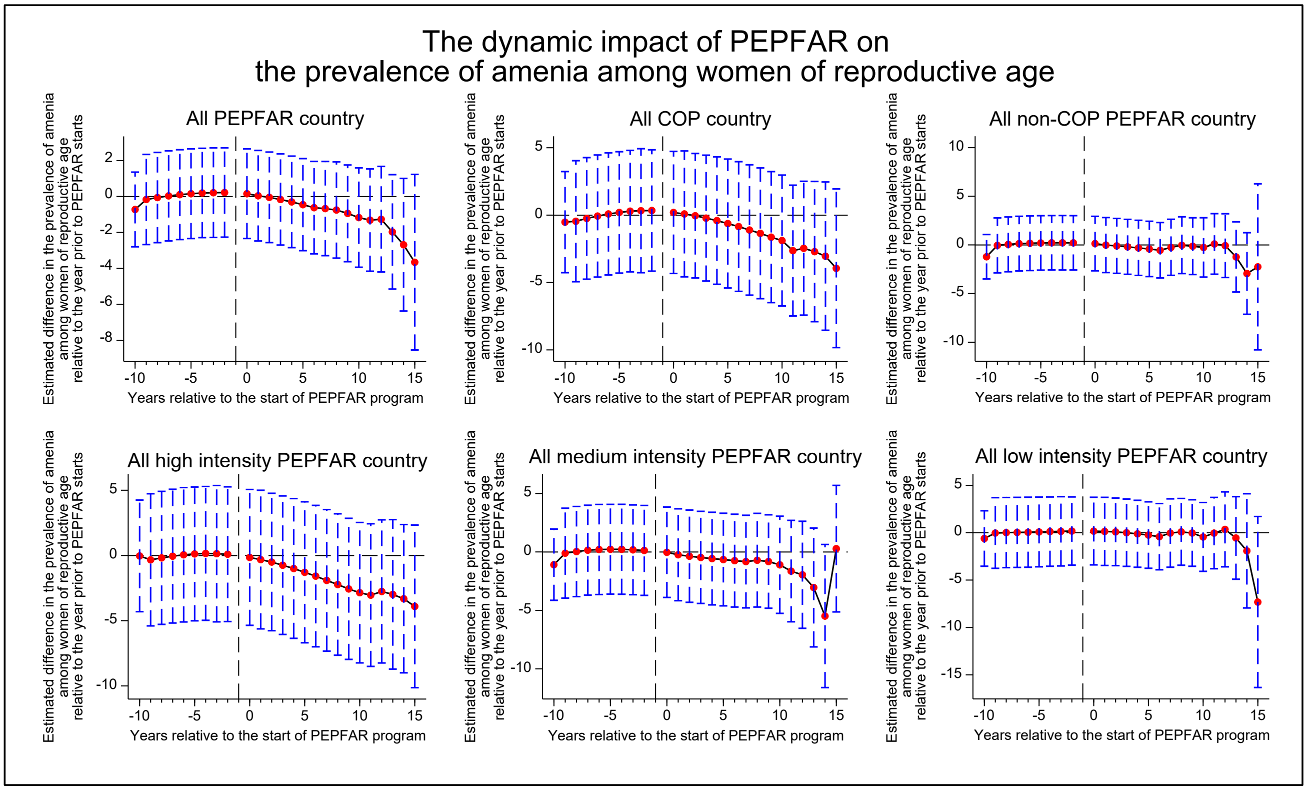

Notes: pepfar_all = all PEPFAR country cohort; pepfar_cop = COP PEPFAR country cohort; pepfar_ncop = non-COP PEPFAR country cohort; pepfar_high = high-intensity PEPFAR country cohort; pepfar_medium = medium-intensity PEPFAR country cohort; pepfar_low = low-intensity PEPFAR country cohort. COP= country operational plans; PEPFAR= President's Emergency Plan for AIDS Relief

S9. Placebo test for staggered DID

S9-1. DPT immunization rate

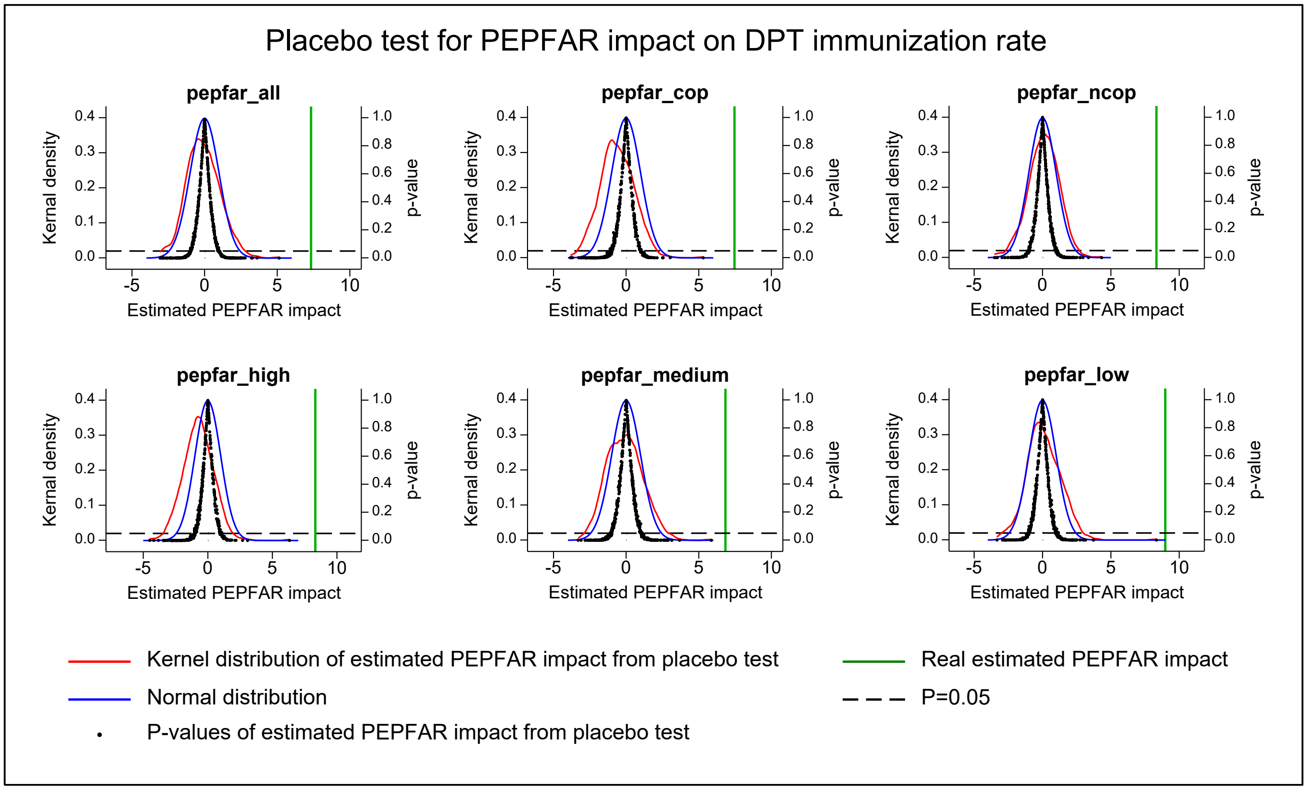

Notes: pepfar_all = all PEPFAR country cohort; pepfar_cop = COP PEPFAR country cohort; pepfar_ncop = non-COP PEPFAR country cohort; pepfar_high = high-intensity PEPFAR country cohort; pepfar_medium = medium-intensity PEPFAR country cohort; pepfar_low = low-intensity PEPFAR country cohort. COP= country operational plans; DID=difference-in-differences; PEPFAR= President's Emergency Plan for AIDS Relief

S9-2. Hepatitis B immunization rate

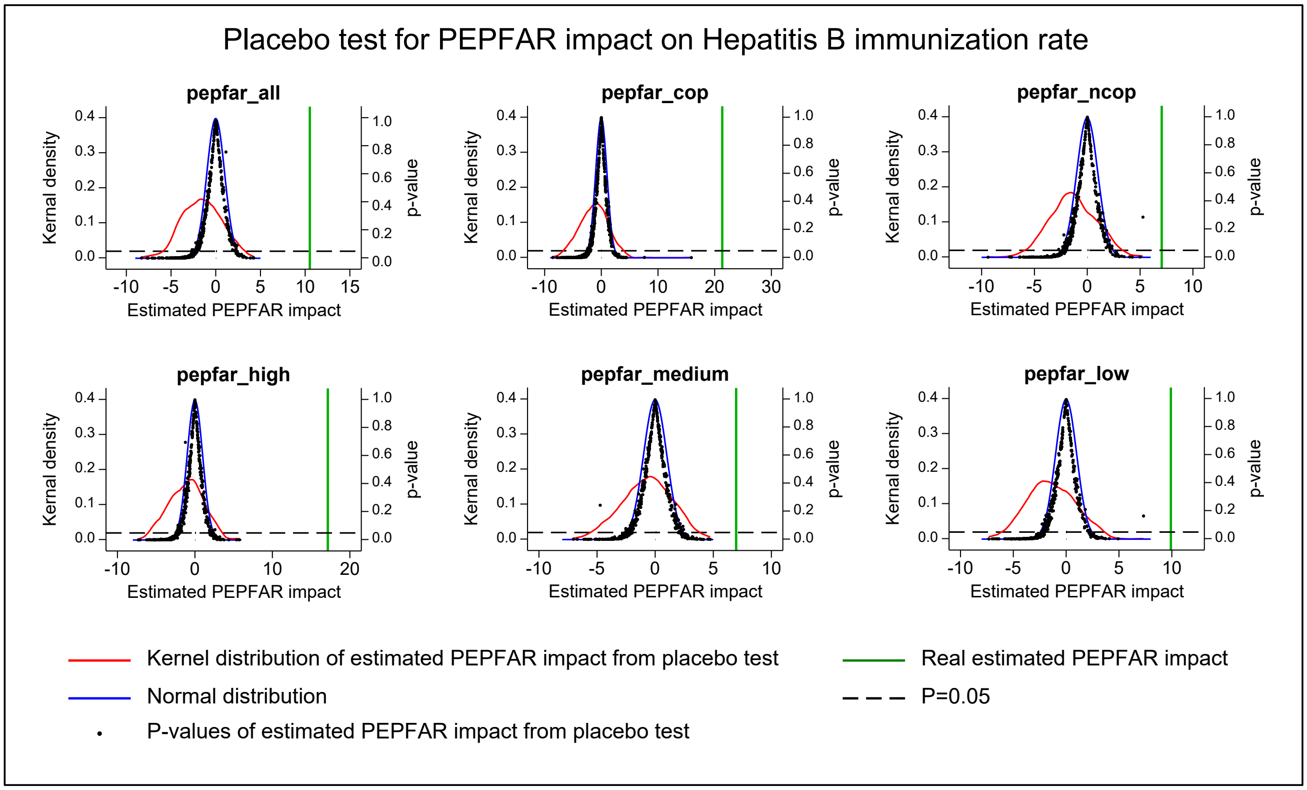

Notes: pepfar_all = all PEPFAR country cohort; pepfar_cop = COP PEPFAR country cohort; pepfar_ncop = non-COP PEPFAR country cohort; pepfar_high = high-intensity PEPFAR country cohort; pepfar_medium = medium-intensity PEPFAR country cohort; pepfar_low = low-intensity PEPFAR country cohort. COP= country operational plans; PEPFAR= President's Emergency Plan for AIDS Relief

S9-3. Measles immunization rate

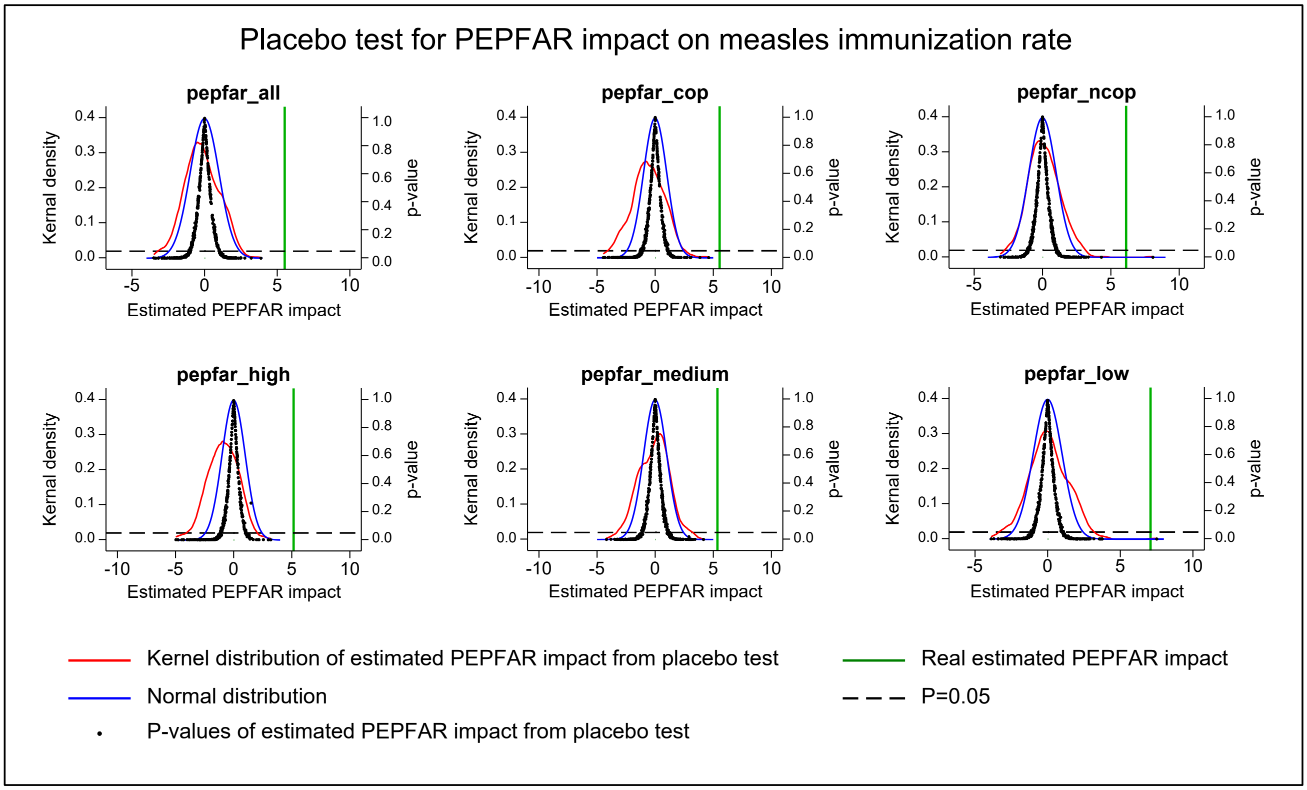

Notes: pepfar_all = all PEPFAR country cohort; pepfar_cop = COP PEPFAR country cohort; pepfar_ncop = non-COP PEPFAR country cohort; pepfar_high = high-intensity PEPFAR country cohort; pepfar_medium = medium-intensity PEPFAR country cohort; pepfar_low = low-intensity PEPFAR country cohort. COP= country operational plans; PEPFAR= President's Emergency Plan for AIDS Relief

S9-4. Prevalence of newborns against tetanus

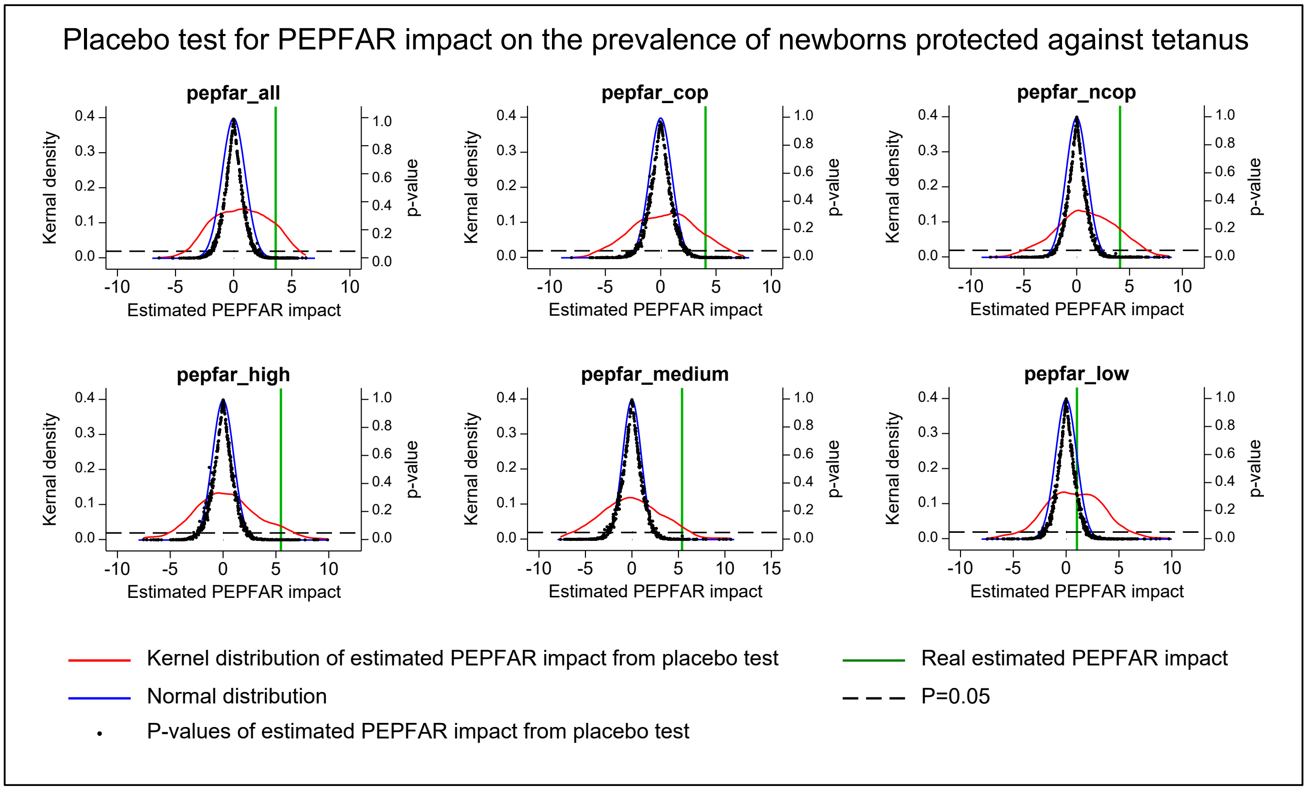

Notes: pepfar_all = all PEPFAR country cohort; pepfar_cop = COP PEPFAR country cohort; pepfar_ncop = non-COP PEPFAR country cohort; pepfar_high = high-intensity PEPFAR country cohort; pepfar_medium = medium-intensity PEPFAR country cohort; pepfar_low = low-intensity PEPFAR country cohort. COP= country operational plans; PEPFAR= President's Emergency Plan for AIDS Relief

S9-5. Maternal mortality ratio

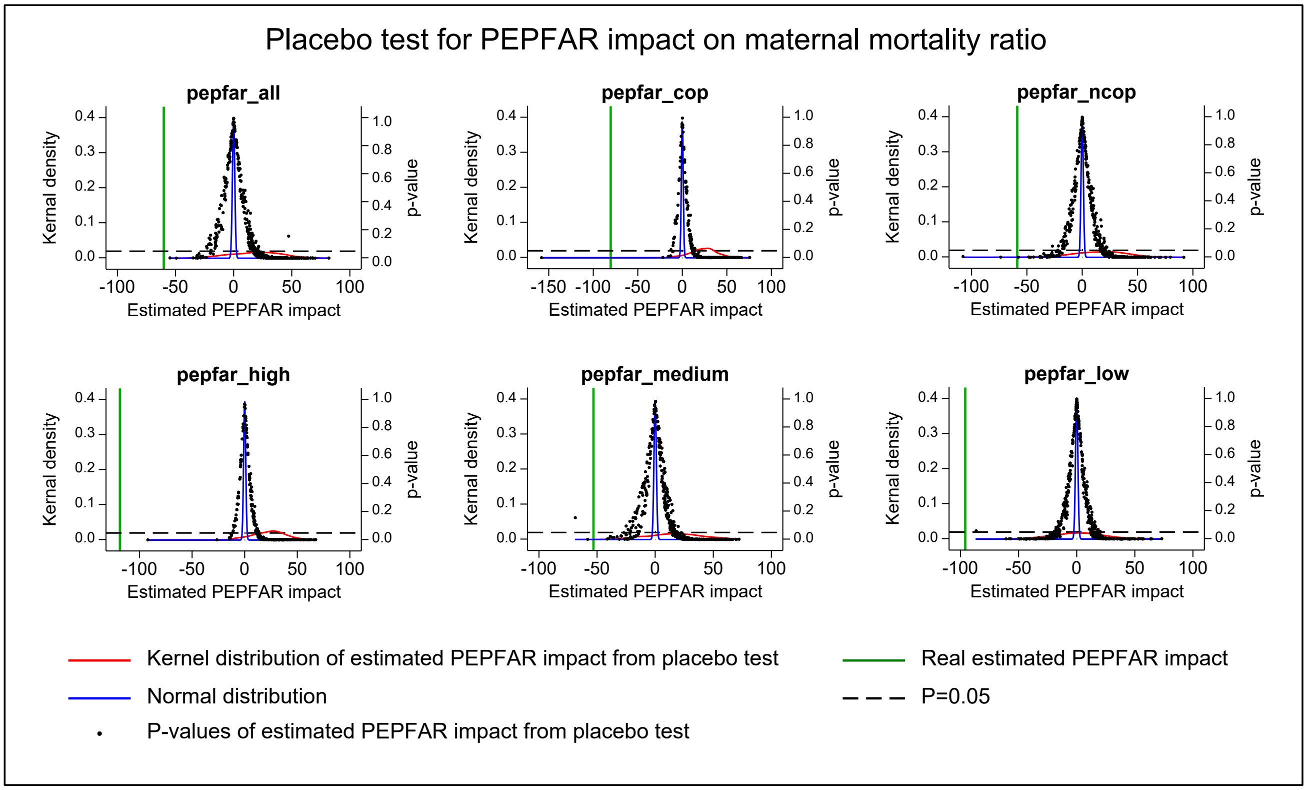

Notes: pepfar_all = all PEPFAR country cohort; pepfar_cop = COP PEPFAR country cohort; pepfar_ncop = non-COP PEPFAR country cohort; pepfar_high = high-intensity PEPFAR country cohort; pepfar_medium = medium-intensity PEPFAR country cohort; pepfar_low = low-intensity PEPFAR country cohort. COP= country operational plans; PEPFAR= President's Emergency Plan for AIDS Relief

S9-6. Under-five mortality rate

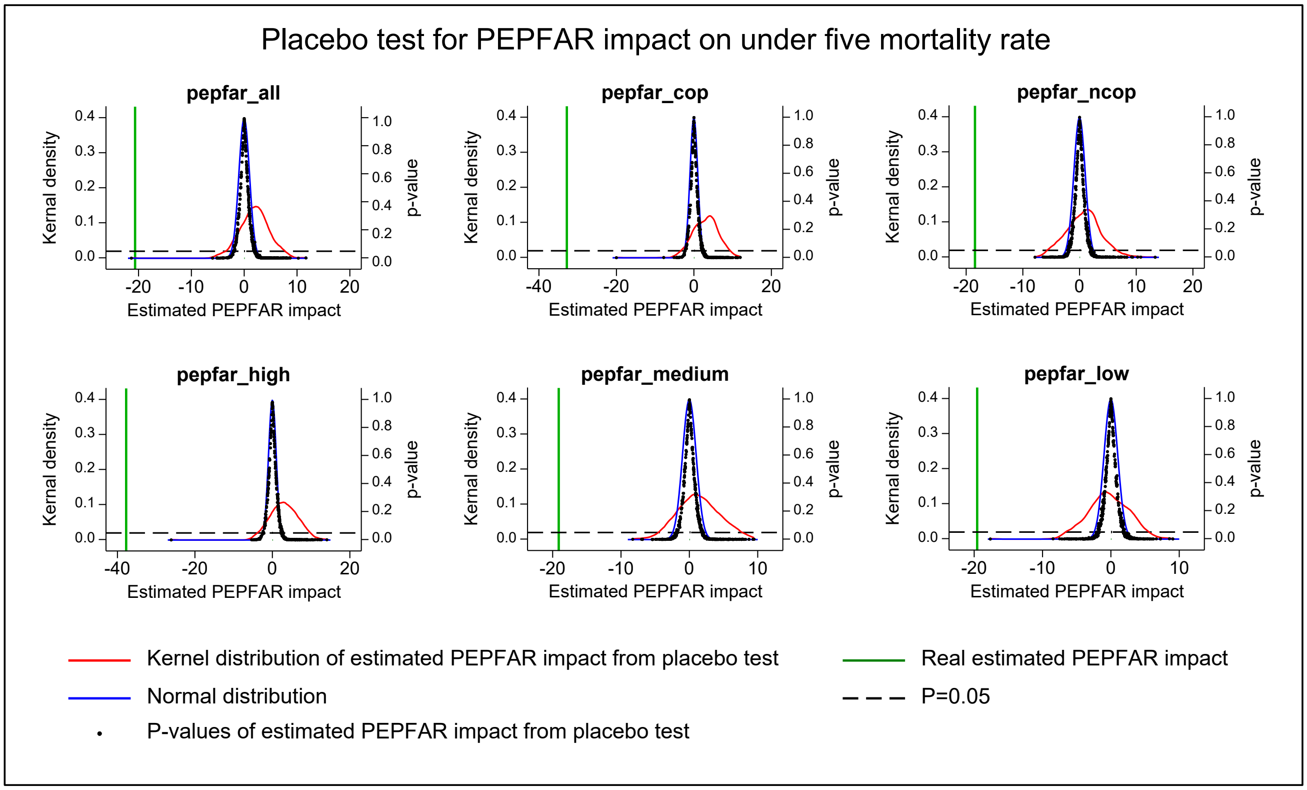

Notes: pepfar_all = all PEPFAR country cohort; pepfar_cop = COP PEPFAR country cohort; pepfar_ncop = non-COP PEPFAR country cohort; pepfar_high = high-intensity PEPFAR country cohort; pepfar_medium = medium-intensity PEPFAR country cohort; pepfar_low = low-intensity PEPFAR country cohort. COP= country operational plans; PEPFAR= President's Emergency Plan for AIDS Relief

S9-7. Prevalence of anemia among women of reproductive age

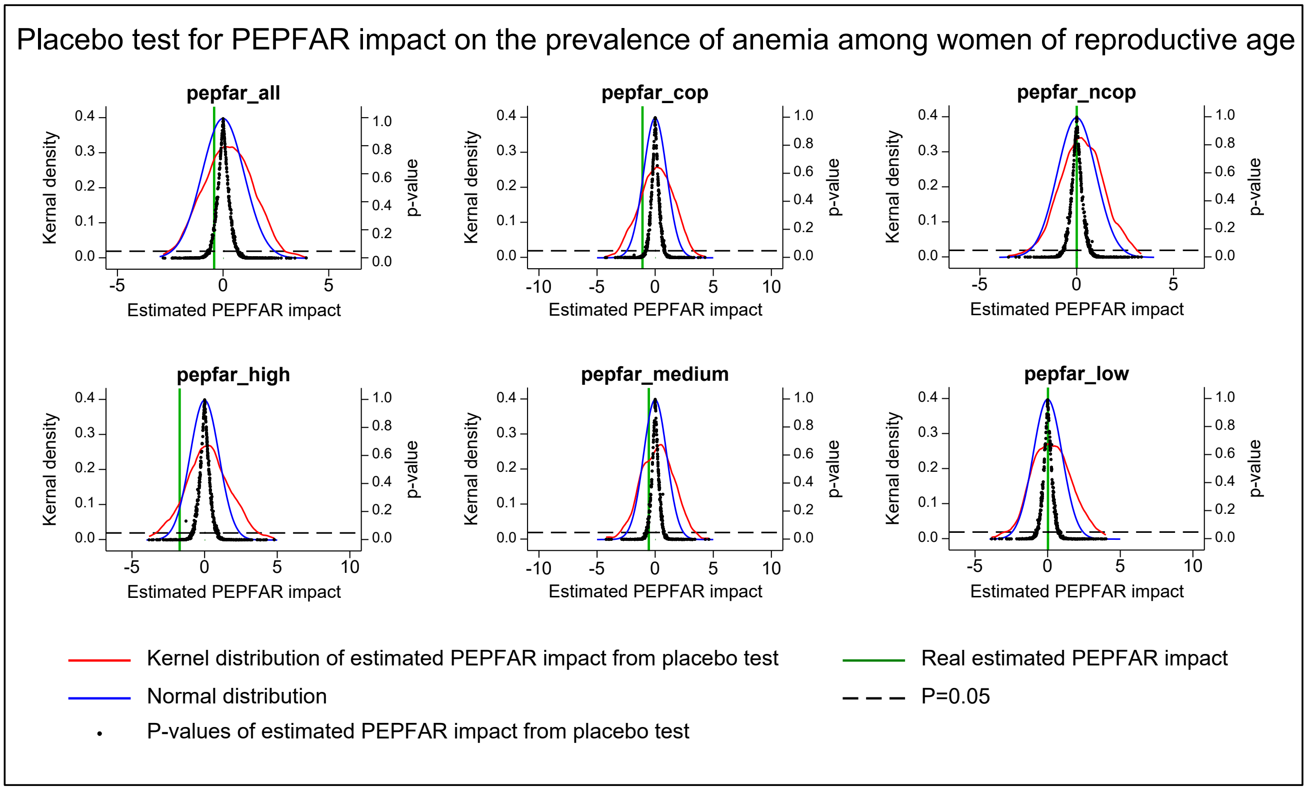

Notes: pepfar_all = all PEPFAR country cohort; pepfar_cop = COP PEPFAR country cohort; pepfar_ncop = non-COP PEPFAR country cohort; pepfar_high = high-intensity PEPFAR country cohort; pepfar_medium = medium-intensity PEPFAR country cohort; pepfar_low = low-intensity PEPFAR country cohort. COP= country operational plans; PEPFAR= President's Emergency Plan for AIDS Relief

S10. Estimation results from logarithmically transformed equations 1 and 3

S10-1. PEPFAR impacts on four immunization rates: traditional and staggered DID estimates

Outcomes and estimation methods	All PEPFAR countries	COP countries	Non-COP countries	High intensity PEPFAR countries	Medium intensity PEPFAR countries	Low intensity PEPFAR countries
**DPT immunization rate**						
Model 1. Unadjusted model (traditional)	0.157***	0.160***	0.154***	0.172***	0.102***	0.193***
Model 1. Unadjusted model (staggered)	0.147***	0.122***	0.190***	0.145***	0.0806***	0.261***
Model 2. Adjusted model (traditional)	0.171***	0.186***	0.165***	0.159***	0.143***	0.214***
Model 2. Adjusted model (staggered)	0.135***	0.155***	0.144***	0.134***	0.141***	0.179***
**Hepatitis B immunization rate**						
Model 1. Unadjusted model (traditional)	0.0894	0.194*	0.0350	0.0669	0.162	0.0121
Model 1. Unadjusted model (staggered)	0.173***	0.404***	0.119*	0.313*	0.134	0.172*
Model 2. Adjusted model (traditional)	0.185**	0.262**	0.172*	0.148	0.220*	0.194*
Model 2. Adjusted model (staggered)	0.252***	0.446***	0.200***	0.353*	0.191**	0.255**
**Measles immunization rate**						
Model 1. Unadjusted model (traditional)	0.118***	0.129***	0.110***	0.118***	0.0769***	0.155***
Model 1. Unadjusted model (staggered)	0.119***	0.0970***	0.151***	0.0972***	0.0629**	0.224***
Model 2. Adjusted model (traditional)	0.124***	0.143***	0.115***	0.105***	0.100***	0.168***
Model 2. Adjusted model (staggered)	0.104***	0.111***	0.104***	0.0778***	0.109***	0.135***
**Prevalence of newborns protected against tetanus**						
Model 1. Unadjusted model (traditional)	0.109***	0.0918**	0.122***	0.109***	0.0979**	0.119***
Model 1. Unadjusted model (staggered)	0.0947***	0.107***	0.103***	0.125***	0.0874**	0.104***
Model 2. Adjusted model (traditional)	0.0978***	0.0747*	0.111***	0.0954**	0.0958**	0.0973**
Model 2. Adjusted model (staggered)	0.0697***	0.0813**	0.0729***	0.0993**	0.0947**	0.0190

***p < 0.001 **p < 0.01 * p< 0.05. Notes: COP=country operational plans; DPT=diphtheria, pertussis, tetanus; PEPFAR= President's Emergency Plan for AIDS Relief

S10-2. PEPFAR impacts on maternal mortality ratio, child (under-five) mortality rate, and the prevalence of anemia among women of reproductive age: traditional and staggered DID estimates

Outcomes and methods	All PEPFAR countries	COP countries	Non-COP countries	High intensity PEPFAR countries	Medium intensity PEPFAR countries	Low intensity PEPFAR countries
**Maternal mortality ratio**						
Model 1. Unadjusted model (traditional)	-0.0337	-0.0171	-0.0424	-0.00368	-0.0530	-0.0444
Model 1. Unadjusted model (staggered)	-0.0406	0.0448	-0.347**	-0.0987	0.383**	-0.881***
Model 2. Adjusted model (traditional)	-0.0358	-0.0504	-0.0282	-0.0275	-0.0612	-0.0177
Model 2. Adjusted model (staggered)	0.0594	0.0214	0.0696	0.00106	-0.0257	0.0251
**Child (under-five) mortality rate**						
Model 1. Unadjusted model (traditional)	-0.0390	0.00754	-0.0634	-0.00887	-0.0656	-0.0425
Model 1. Unadjusted model (staggered)	-0.0147	0.0326	-0.135**	-0.00670	0.103	-0.306***
Model 2. Adjusted model (traditional)	-0.0860**	-0.0516	-0.104***	-0.0759*	-0.0963**	-0.0854**
Model 2. Adjusted model (staggered)	-0.0521**	-0.0394	-0.0758***	-0.0620*	-0.0907***	-0.0750**
**Prevalence of anemia among women of reproductive age**						
Model 1. Unadjusted model (traditional)	-0.0116	-0.0224	-0.00592	-0.0409	-0.000701	0.00682
Model 1. Unadjusted model (staggered)	-0.0189	-0.000761	-0.0484*	-0.0211	0.0506	-0.103***
Model 2. Adjusted model (traditional)	0.0140	0.00718	0.0176	-0.0106	0.0219	0.0305
Model 2. Adjusted model (staggered)	0.0130	0.0138	0.0200	-0.00408	0.00721	0.0145

***p < 0.001 **p < 0.01 * p< 0.05. Notes: COP=country operational plans; PEPFAR= President's Emergency Plan for AIDS Relief

** p<0.001, ** p<0.01, * p<0.05 BL=baseline; COP=country operational plans; PEPFAR= President's Emergency Plan for AIDS Relief
