# Supplementary material for: Analysis of maternal and child health spillover effects in PEPFAR countries
Source: BMJ Open. 2023 Dec 21;13(12):e070221. doi: 10.1136/bmjopen-2022-070221 (PMC10748962; doi:10.1136/bmjopen-2022-070221)
Supplement: Author's manuscript [file bmjopen-2022-070221.draft_revisions.pdf]

BMJ Open is committed to open peer review. As part of this commitment we make the peer review history of every article we publish publicly available.

When an article is published we post the peer reviewers' comments and the authors' responses online. We also post the versions of the paper that were used during peer review. These are the versions that the peer review comments apply to.

The versions of the paper that follow are the versions that were submitted during the peer review process. They are not the versions of record or the final published versions. They should not be cited or distributed as the published version of this manuscript.

BMJ Open is an open access journal and the full, final, typeset and author-corrected version of record of the manuscript is available on our site with no access controls, subscription charges or pay-per-view fees (http://bmjopen.bmj.com).

If you have any questions on BMJ Open's open peer review process please email
info.bmjopen@bmj.com

BMJ Open

Analysis of Maternal and Child Health Spillover Effects in PEPFAR Countries: An Economic Evaluation

Journal:	*BMJ Open*
Manuscript ID	bmjopen-2022-070221
Article Type:	Original research
Date Submitted by the Author:	08-May-2023
Complete List of Authors:	Gaumer, Gary; Brandeis University Heller School for Social Policy and Management, Institute for Global Health and Development<br>Hariharan, Dhwani; Brandeis University Heller School for Social Policy and Management, Institute for Global Health and Development<br>Crown, William H; Brandeis University Heller School for Social Policy and Management, Institute for Global Health and Development<br>Kates, Jennifer; The Kaiser Family Foundation, Global Heallh and HIV Policy<br>Jordan, Monica; Brandeis University Heller School for Social Policy and Management, Institute for Global Health and Development<br>Hurley, Clare; Brandeis University Heller School for Social Policy and Management, Institute for Global Health and Development<br>Nandakumar, Allyala; Brandeis University Heller School for Social Policy and Management, Institute for Global Health and Development
Keywords:	Health policy < HEALTH SERVICES ADMINISTRATION & MANAGEMENT, Health economics < HEALTH SERVICES ADMINISTRATION & MANAGEMENT, HIV & AIDS < INFECTIOUS DISEASES, QUALITATIVE RESEARCH

SCHOLARONE™
Manuscripts

I, the Submitting Author has the right to grant and does grant on behalf of all authors of the Work (as defined in the below author licence), an exclusive licence and/or a non-exclusive licence for contributions from authors who are: i) UK Crown employees; ii) where BMJ has agreed a CC-BY licence shall apply, and/or iii) in accordance with the terms applicable for US Federal Government officers or employees acting as part of their official duties; on a worldwide, perpetual, irrevocable, royalty-free basis to BMJ Publishing Group Ltd ("BMJ") its licensees and where the relevant Journal is co-owned by BMJ to the co-owners of the Journal, to publish the Work in this journal and any other BMJ products and to exploit all rights, as set out in our licence.

The Submitting Author accepts and understands that any supply made under these terms is made by BMJ to the Submitting Author unless you are acting as an employee on behalf of your employer or a postgraduate student of an affiliated institution which is paying any applicable article publishing charge ("APC") for Open Access articles. Where the Submitting Author wishes to make the Work available on an Open Access basis (and intends to pay the relevant APC), the terms of reuse of such Open Access shall be governed by a Creative Commons licence – details of these licences and which Creative Commons licence will apply to this Work are set out in our licence referred to above.

Other than as permitted in any relevant BMJ Author's Self Archiving Policies, I confirm this Work has not been accepted for publication elsewhere, is not being considered for publication elsewhere and does not duplicate material already published. I confirm all authors consent to publication of this Work and authorise the granting of this licence.

For peer review only

Analysis of Maternal and Child Health Spillover Effects in PEPFAR Countries: An Economic Evaluation

Gary Gaumer[1]* Dhwani Hariharan[1], William Crown[1], Jennifer Kates[2], Monica Jordan[1], Clare Hurley[1], AK Nandakumar[1]

May 2, 2023

[1]Institute for Global Health and Development, The Heller School for Social Policy and Management, Brandeis University, Waltham, MA, USA

[2] Kaiser Family Foundation, Global Health & HIV Policy, Washington, DC, USA

*Corresponding Author: Gary Gaumer, PhD, Institute for Global Health and Development, Heller School for Social Policy and Management, Brandeis University, 415 South Street/MS035, Waltham, MA 02453, USA. E-mail: garygaumer@gmail.com

Word count for Introduction to References: 3,791

For peer review only

ABSTRACT

Objectives: This study examines whether the US President's Emergency Plan for AIDS Relief (PEPFAR) funding may have had effects beyond HIV, specifically on several measures of maternal and child health.

Design: Panel data and a difference-in-differences estimator was used to estimate the impact of the PEPFAR program from 2004-2018 using a control group of 67 low- and middle-income countries (LMICs).

Setting: All Low and Middle income Countries 1990-2018.

Participants: 90 LMICs receiving PEPFAR aid, and segments of those countries including those required to submit annual country operating plans (COP), other countries (non-COP), and segments of countries with high, medium and low intensity of PEPFAR aid per capita.

Interventions: PEPFAR aid to combat the HIV epidemic.

Primary outcome measures: Maternal mortality and child mortality rates, vaccination rates to protect children for DPT, measles, HepB3, and tetanus, and prevalence of anemia in women of childbearing age.

Results: Across PEPFAR recipient countries, very large, favorable PEPFAR health spillover effects are found for childhood immunization rates, and for child and maternal mortality rates. These health effects were large and significant in all segments of PEPFAR recipient countries studied. We also find significant and favorable program effects on prevalence of anemia in women of childbearing age in PEPFAR recipient countries receiving the most intensive support from the PEPFAR program. Other recipient countries did not demonstrate significant effects on anemia.

Conclusions: This study demonstrates that important health indicators, beyond HIV, have been consistently and favorably influenced by PEPFAR presence: child and maternal mortality and childhood immunization rates. We also found no evidence of "crowding out" or negative spillovers in these areas in resource-poor countries. These findings add to the evidence base that PEPFAR has had favorable health effects beyond HIV.

Article Summary:

This study examines whether PEPFAR may have had health effects beyond HIV. Specifically, we look at the relationship between PEPFAR funding and several measures of maternal and child health. Results show that across PEPFAR recipient countries, favorable PEPFAR health spillover effects are found for childhood immunization rates, child and maternal mortality rates, and prevalence of anemia in women of childbearing age. These findings add to the evidence base that PEPFAR has had favorable health effects beyond HIV.

For peer review only - http://bmjopen.bmj.com/site/about/guidelines.xhtml

Strengths and Limitations:

- This study used long baseline and treatment periods using a difference-in-difference research design, which is a useful tool when individual randomization is not feasible.
- Though we controlled for numerous other country differences, we may have failed to control for other unobserved variables and possible biasing our estimates of the magnitude of PEPFAR impact on various outcomes.
- Construction of our LMIC comparison group, including 18 countries that received very low amounts of aid from PEPFAR, may make our estimated program impacts somewhat conservative.

Keywords: HIV, PEPFAR, low- and middle-income countries, spillover, impacts, panel dataset, maternal and child health

For peer review only

For peer review only - http://bmjopen.bmj.com/site/about/guidelines.xhtml

INTRODUCTION AND OBJECTIVES

The US President's Emergency Plan for AIDS Relief (PEPFAR) was authorized in 2003 to address the impact of HIV globally, and represents the largest commitment by any nation to address a single disease.[1] Between 2004 and 2018, PEPFAR disbursed approximately $70 billion in bilateral aid to provide testing, prevention, treatment, and other services in more than 100 low- and middle-income countries (LMICs), and reports that it has supported nearly 20 million persons with life-saving antiretroviral treatment (ARV). PEPFAR also reports that its efforts have enabled 5.5 million babies to be born HIV-free, through the reduction of HIV incidence and prevalence among women and men of child-bearing age.[2]

While PEPFAR is credited with savings millions of lives and changing the trajectory of the HIV epidemic, there have been concerns about negative spillover (unintended and indirect) effects on other health sectors, given the vertical nature of the PEPFAR program and its significant size.[3] Some have argued that PEPFAR could theoretically "crowd out" the availability of scarce human and other resources from other health programs in these resource-poor countries.[4-11] If so, these negative spillover effects could potentially lead to worse, non-HIV related outcomes.[7 9] One study, using the OECD Credit Reporting System (CRS) data, found financial displacement effects within countries, showing that more HIV/AIDS funding was associated with a reduction in malaria program funding.[7] Another study found HIV funding was associated with reduced provision of childhood immunizations.[12]

Other researchers have argued and demonstrated that PEPFAR could indirectly result in positive health spillovers effects in other areas of health, particularly maternal and child health.[6] One confirming study of favorable impacts on maternal care in 257 facilities in eight African countries, found evidence that pregnant women who were not infected with HIV were more likely to deliver in hospitals in areas that had more HIV-funded patient services or more HIV infrastructure support.[5] Another study examined 46 countries in sub-Saharan Africa (SSA) from 1995-2010, and found three of four vaccination indicators were positively associated with PEPFAR.[13] This study found, however, that PEPFAR was not significantly associated with improvements in infant mortality and under-five child mortality.[12]

While PEPFAR does not directly fund non-HIV specific maternal and child health services, its potential impact in this area is plausible, since it supports HIV interventions and programs that are intended to address maternal and child mortality, such as the prevention of mother to-child transmission (PMTCT) program and the program for Saving Mothers, Giving Life.[14-16] These programs provide resources to prevent vertical transmission to newborns, and also provide care, treatment, and support to women living with HIV and their families. These programs should translate into reductions in infant and under-five deaths. Two studies of under-5 mortality, however, do not show significant reductions associated with PEPFAR.[13 17] One study shows favorable impacts on child mortality by the President's Malaria Initiative in 19 countries in sub-Saharan Africa from 2006 to 2014, but the PEPFAR covariate was found to be insignificant in influencing child mortality.[18] In a recent Kenya study of PMTCT impacts, the authors found infant mortality declines associated with PEPFAR, but the study did not examine child mortality.[19]

For peer review only - http://bmjopen.bmj.com/site/about/guidelines.xhtml

Although the intended outcomes of these programs were to save many maternal lives via antiretroviral therapy (ART), and to save many children's lives by preventing HIV from spreading to them from their infected mothers, the programs may have also improved non-HIV care of women and their children through counseling and linkage efforts, and relationship-building with providers. Indeed, PEPFAR has increasingly emphasized reaching women where they receive prenatal care and seek immunizations and other services for their children, and on integrating services.[20 21]

A second PEPFAR programmatic area of focus that might produce potential spillover effects is through PEPFAR's investments in health system strengthening (HSS), including investments in training the health care workforce, estimated to be over $1 billion annually.[22] These investments, in turn, may have had favorable health impacts through health care worker training, improved management, infrastructure, equipment, and supply chains, and may have even improved the operational culture of these places.[23] In addition to the need to geographically expand the availability of HIV treatment, prevention, and testing services, the HSS interventions may have also led to adding and improving health services in previously underserved areas, and by so doing, exposing many households to medical care services for the first time. Emerging evidence from activity-based costing and management studies across multiple countries indicates that people who are fully funded to do HIV work spend 30-40% of their time providing broader primary health care services at the facility level.[24 25]

METHODS

Using a 29-year panel dataset (1990-2018) of 157 LMICs, we assessed the spillover impacts of PEPFAR in 90 recipient countries on several measures of maternal and child health (MCH). Indicators were selected from the World Bank's World Development Indicators (WDI)[26] list and represent global health target areas[3] (Table 1).

<Insert Table 1 about here>

We estimated PEPFAR impacts using the difference-in-differences (DID) method (see **Supplement S1).** The comparison group included 67 LMICs that either did not receive any PEPFAR support or received minimal PEPFAR support (<$1M in total or <$0.05 per capita cumulatively between 2004 and 2018).

Three model specifications were examined. The basic unadjusted DID model (Model 1) uses three dummy variables to estimate the DID impacts: a dummy variable that captures the overall differences in the mean value of the dependent variable between the baseline period (pre-2004) and the follow-up period, which measures the time trend; a dummy variable for PEPFAR countries and comparison group countries that measures differences at baseline; and an interaction dummy between the first two dummy variables, which is interpreted as the impact of the PEPFAR program. Model 2 adds a set of baseline covariates to model 1, including HIV prevalence rate (% of population with HIV among those ages 15-49), country income level,

For peer review only - http://bmjopen.bmj.com/site/about/guidelines.xhtml

population, GDP per capita (purchasing power parity (PPP), constant international dollars), percent population urban, secondary school enrollment (% gross), life expectancy at birth, fertility rate (births per woman), and if the country received HIV aid from the U.S. prior to 2004. Model 3 adds two additional baseline covariates - other donor health spending per capita and domestic total health spending per capita. For the three childhood vaccination variables, models 2 and 3 also included baseline prevalence of the target infection in the under 5 population (diphtheria, whooping cough and tetanus (DPT), measles, and hepatitis B (HepB)).

Data sources for all covariates included the World Bank's World Development Indicators (WDI) database,[26] the U.S. government's Foreignassistance.gov database,[27] the Organization for Economic Co-operation and Development (OECD) Creditor Reporting System database,[28] and the Institute of Health Metrics and Evaluation's GBD Results tool.[29] We also used data from the Office of the Global AIDS Coordinator and Health Diplomacy (OGAC), the Congressionally authorized office which manages and oversees PEPFAR. Ethics approval was not required for this study. Neither patients nor the public were involved in this research study.

In addition to examining outcomes for all 90 PEPFAR recipient countries taken together, we also estimated PEPFAR impacts on separate cohorts of recipient countries. These separate cohorts were created based on (1) the intensity of PEPFAR funding (cumulative PEPFAR disbursements per capita from 2004 to 2018 - high, medium, and low), and (2) OGAC programmatic planning differences (whether the country had to develop an annual Country Operational Plan (COP) with OGAC or not) (see Supplement S2 for list of countries in these segments).

RESULTS

Trends in the outcomes from 1990-2018 are presented in Figures 1 and 2 across PEPFAR recipient, PEPFAR COP, and comparison countries. Descriptive data indicate that PEPFAR recipient countries, particularly the COP countries, have lower (poorer) initial values for most indicators. In all cases the pre-period trend pattern (before 2004) is generally the same in PEPFAR countries and comparisons, though in some cases there is evidence that the roughly parallel baseline trend is altered in PEPFAR countries after 2004.

The four immunization indicators in Figure 1 all show the same general pattern: PEPFAR segments have lower initial immunization rates than comparisons and all show increasing immunization rates over time. In all cases, however, the uptake appears to be faster in PEPFAR countries than in comparison countries. The faster PEPFAR uptake for DPT and measles is more pronounced than for Hepatitis B, and the situation is even less pronounced for tetanus than Hepatitis B.

In Figure 2A, the MMR for comparison countries is rather flat and not improving, while rates in PEPFAR recipient countries improve and catch up to the comparison group. The under-5 child mortality rate (Figure 2B) shows a very clear initial pattern of higher baseline child mortality in PEPFAR recipient countries and then faster declines after 2004. In COP and high-spending recipient countries, there were higher baseline levels of under-5 mortality and even faster

For peer review only - http://bmjopen.bmj.com/site/about/guidelines.xhtml

declines after 2004. Trends show poorer baseline levels of anemia among women aged 15-49 in PEPFAR countries (Figure 2C), and particularly in COP and highly supported country segments, with only slight evidence of convergence between PEPFAR and comparison countries.

Multivariate regression analyses examined the estimated impact of PEPFAR on the seven indicators across the various PEPFAR recipient cohorts (All, COP, non-COP, and by intensity of PEPFAR spending). These results are presented in Tables 2 and 3 for each of the three model specifications. The complete modeling results for each outcome are presented in Supplements S3-S9.

<Insert Table 2 about here>

The data on childhood immunizations suggest that PEPFAR presence has been associated with improvements in three childhood vaccination rates relative to what would have been expected in the absence of the program, as seen in 67 of 72 models presented in Table 2. Similarly, there has been improvement in tetanus coverage for newborns, a vaccine administered to pregnant women. In no case is there evidence of a deterioration in immunization rates due to PEPFAR presence. Across models, we estimate the effects of PEPFAR on immunization rates to be about 5-10% higher than would have otherwise been expected to occur, and in the country segments where aggressive COP management is used and where PEPFAR spends the most money, the percentage improvements associated with PEPFAR presence are typically larger.

Maternal mortality ratios (Table 3) are substantially and consistently lower in the presence of PEPFAR. These positive spillover effects for all recipient countries taken together (Table 3, column 1) are estimated to be declines in the MMR of approximately 24%. The pattern generally shows larger coefficients on MMR in COP and high-spending segments. These PEPFAR segments have a much higher baseline MMR.

<Insert Table 3 about here>

Child mortality rates are similarly reduced in the presence of PEPFAR. Consistent reductions in all models are shown in the range of over 35% in the high impact segments and somewhat lower in other recipient countries.

PEPFAR effects in reducing prevalence of anemia in women of childbearing age were evident in some segments but not others. These spillover benefits are only statistically significant in the models for COP and high-spending segments, in the range of 4-6% reductions in those segments. The other segments show no significance in any of the models.

DISCUSSION

Our assessment of PEPFAR's impact on several non-HIV/AIDS health measures in the area of maternal and child health finds favorable, large, spillover effects. Though immunizations are

For peer review only - http://bmjopen.bmj.com/site/about/guidelines.xhtml

not provided directly by PEPFAR, utilization rates for several childhood immunizations demonstrate positive spillover health benefits of PEPFAR presence in recipient countries. Childhood immunizations were 5-10% higher by 2018 than would have been expected in the absence of PEPFAR. The immunization spillover effects have been demonstrated before in less comprehensive studies.[13]

MMR and under-5 child mortality rates also show large, consistent patterns of positive health spillover effects of the PEPFAR program. Child mortality impacts are quite large, with reductions of over 30% across country segments. MMR declined by about 24%. Limited evidence is shown for favorable impacts on the prevalence of anemia in women of childbearing age in COP and highly-funded countries. Child and maternal mortality and prevalence of anemia among women indicators have not been previously studied using multi-country panel data.

Our findings are particularly strong for child and maternal mortality. Some of these reductions are likely due to reductions in HIV-related mortality, but they appear to extend beyond this. For example, HIV accounted for 5.1% of the estimated 4 million child deaths in sub-Saharan Africa in 2003, compared to 1.6% of the estimated 2.8 million child deaths in 2018, a drop that would not fully drive the overall decline in child mortality we estimate.[30] Similarly, HIV, which is not considered to be a direct cause of maternal mortality, was estimated to have accounted for 6.4% of maternal deaths in sub-Saharan Africa between 2003 and 2009.[3]

No negative spillover effects were found for any of the seven outcomes assessed. While our evidence of positive spillover effects on maternal and child health is notable and important, the mechanisms for such effects are not readily apparent from our work. As noted earlier, one possible means is through PEPFAR's efforts to address HIV in pregnant women and children, since it brings women (and their children) into contact with formal health care services and providers.[20] A second, related mechanism may be the widespread scale-up of PEPFAR infrastructure that has created health-delivery system capacities to serve many isolated and previously underserved communities; this scale up often has been more rapid than the demand for HIV services, enabling and encouraging provision of other needed medical services in these locations.[24] More research is needed to document the source mechanisms of these and possibly other spillover benefits.

Strengths and Limitations:

Despite the strengths of the difference-in-difference research design, there are limitations to this analysis. Even though we attempted to control for numerous other factors that may be associated with both PEPFAR presence and our outcomes of interest, we may have failed to control for other unobserved variables thereby biasing our estimates of the magnitude of PEPFAR impact on the various outcomes. It should also be noted that our estimates reflect average effects across all PEPFAR and non-PEPFAR countries in the sample. It is likely that PEPFAR impacts are much larger than average in some countries and smaller in others.

On the other hand, the construction of our LMIC comparison group may make our estimated impacts somewhat conservative. Included within this group are 18 countries that did receive some aid from PEPFAR, though very low amounts. To the extent that this minimal aid

For peer review only - http://bmjopen.bmj.com/site/about/guidelines.xhtml

(cumulative aid is less than $1M or less than $0.50 per capita over the period) was effective in saving lives or having some positive health impacts, the estimates of impact presented in this study will be too small.

CONCLUSIONS

In addition to averting HIV deaths and limiting the spread of HIV, we find that PEPFAR has had other health benefits beyond HIV for LMIC populations, without any evidence of adverse spillover effects that some feared. While some early and focused studies reported instances of crowding out and evidence of negative spillover effects, this study, which includes a more comprehensive, multi-country data set, with observations obtained over a longer time period, did not identify such effects.

This study demonstrates evidence of large, positive spillovers in maternal and child mortality and consistent improvements in childhood immunization rates. These effects likely stem from significant resource investments in health system infrastructure, staff and equipment, and improved practice patterns, among other factors.[13, 31] The specific mechanisms for such effects warrant further research.

For peer review only - http://bmjopen.bmj.com/site/about/guidelines.xhtml

Acknowledgments: The authors thank Yara Halasa-Rappel, Brandeis University, Waltham, MA, and Adam Wexler, Kaiser Family Foundation (KFF), Washington DC, for data retrieval and dataset preparation.

Contributors: GG, WC, JK and AKN led the study conception and design; GG, DH, WC, JK and MJ conducted the analysis and interpretation of results; GG, DH, WC, JK, MJ, and CLH contributed to manuscript preparation; AKN was involved in funding acquisition and supervision. All authors reviewed the results and approved the final version of the manuscript. GG is the guarantor of this work.

Funding sources: This paper was produced with funding from Palladium International, LLC under subcontract number 217730-Brandeis-01; and Prime Contract number 2021-002516 from the Global Fund to Fight AIDS, Tuberculosis, and Malaria. Its contents are solely the responsibility of Brandeis University and do not necessarily represent the official views of Palladium or The Global Fund.

Conflict of Interest: None declared.

Patient and public involvement: Patients or the public were not involved in the design, conduct, reporting, or dissemination plans of this research study.

Patient consent: Not applicable.

Ethics approval: Ethics approval was not required for this study.

Provenance and peer review: Not commissioned; externally peer reviewed.

Data availability: Our data came from four publicly available datasets: World Bank's World Development Indicators; U.S. government's foreignassistance.gov database; OECD Creditor Reporting System database; and the Institute of Health Metrics and Evaluation GBD Result's Tool.

Supplemental Material: This content has been supplied by the authors. It has not been vetted by BMJ Publishing Group Limited (BMJ) and may not have been peer-reviewed. Any opinions or recommendations discussed are solely those of the authors and are not endorsed by BMJ. BMJ disclaims all liability and responsibility arising from any reliance placed on the content. Where the content includes any translated material, BMJ does not warrant the accuracy and reliability of the translations (including but not limited to local regulations, clinical guidelines, terminology, drug names and drug dosages), and is not responsible for any error and/or omissions arising from translation and adaptation or otherwise.

Open access: This is an open access article distributed in accordance with the Creative Commons Attribution 4.0 Unported (CC BY 4.0) license, which permits others to copy, redistribute, remix, transform and build upon this work for any purpose, provided the original

For peer review only - http://bmjopen.bmj.com/site/about/guidelines.xhtml

work is properly cited, a link to the license is given, and indication of whether changes were made. See: https://creativecommons.org/licenses/by/4.0/.

ORCID IDs:
Gary Gaumer: https://orcid.org/0000-0003-3304-1258
Dhwani Hariharan: https://orcid.org/0000-0001-7329-7376
William Crown: https://orcid.org/0000-0002-8822-2737
Allyala Nandakumar: https://orcid.org/0000-0002-0976-9313

Author email addresses:
Gary Gaumer: garygaumer@gmail.com
Dhwani Hariharan: dhwanihariharan@brandeis.edu
William Crown: wcrown@brandeis.edu
Jennifer Kates: JenniferK@kkf.org
Monica Jordan: mjordan@brandeis.edu
Clare Hurley: churley@brandeis.edu
AK Nandakumar: aknkumar@brandeis.edu

For peer review only

REFERENCES

1. PEPFAR. The United States President's Emergency Plan for AIDS Relief 2021 Annual Report to Congress 2021 Available: https://www.state.gov/wp-content/uploads/2021/02/PEPFAR2021AnnualReporttoCongress.pdf. [Accessed 21 Jun 2022].
2. PEPFAR. PEPFAR's Latest Global Results 2022 Available: https://www.state.gov/wp-content/uploads/2022/07/PEPFAR-Latest-Global-Results_JULY-2022_FINAL.pdf. [Accessed 26 Aug 2022].
3. Kates J, Gaumer G, Crown W, *et al*. Assessing PEPFAR's Impact: Analysis of Maternal and Child Health Spillover Effects in PEPFAR Countries. 2022. Available: https://www.kff.org/global-health-policy/issue-brief/assessing-pepfars-impact-analysis-of-maternal-and-child-health-spillover-effects-in-pepfar-countries/. [Accessed 26 Aug 2022].
4. Garrett L. The Challenge of Global Health Foreign Affairs 2007;Jan/Feb:14-38. Available: https://www.foreignaffairs.com/articles/world/2007-01-01/challenge-global-health. [Accessed 17 Mar 2023].
5. Kruk ME, Jakubowski A, Rabkin M, *et al*. PEPFAR programs linked to more deliveries in health facilities by African women who are not infected with HIV. *Health Aff (Millwood)* 2012;31(7):1478-88. doi: 10.1377/hlthaff.2012.0197
6. Leeper SC, Reddi A. United States global health policy: HIV/AIDS, maternal and child health, and The President's Emergency Plan for AIDS Relief (PEPFAR). *AIDS* 2010;24(14):2145-9.
7. Lordan G, Tang KK, Carmignani F. Has HIV/AIDS displaced other health funding priorities? Evidence from a new dataset of development aid for health. *Soc Sci Med* 2011;73(3):351-5.
8. McCoy D, Chopra M, Loewenson R, *et al*. Expanding access to anti-retroviral therapy in sub-Saharan Africa, avoiding pitfalls and dangers, capitalizing on the opportunities. *Am J Pub Health* 2005;95(1):18–22.
9. Shiffman J. Has donor prioritization of HIV/AIDS displaced aid for other health issues? *Health Policy Plan* 2008;23(2):95–100.
10. Walensky RP, Kuritzkes DR. The impact of the President's Emergency Plan for AIDS Relief (PEPFAR) beyond HIV and why it remains essential. *Clin Infect Dis* 2010;50(2):272–75.
11. Walsh A, Ndubani P, Simbaya J, *et al.* Task Sharing in Zambia: HIV service scaleup compounds the human resource crisis. *BMC Health Serv Res* 2010;10(1):272.
12. Grépin KA. HIV donor funding has both boosted and curbed the delivery of different non-HIV health services in sub-Saharan Africa. *Health Aff (Millwood)* 2012;31(7):1406-14.
13. Cohen RL, Li Y, Giese R, *et al*. An evaluation of the President's Emergency Plan for AIDS Relief effect on health systems strengthening in sub-Saharan Africa. *J Acquir Immune Defic Syndr* 2013;62(4):471-9.
14. Conlon CM, Serbanescu F, Marum L, *et al.* Saving Mothers, Giving Life: It Takes a System to Save a Mother (Republication). *Glob Health Sci Pract* 2019;7(1):20-40.
15. Kruk ME, Rabkin M, Grépin KA, *et al.* 'Big Push' To Reduce Maternal Mortality In Uganda And Zambia Enhanced Health Systems But Lacked A Sustainability Plan. *Health Aff (Millwood)* 2014;66(6):1058-66.

For peer review only - http://bmjopen.bmj.com/site/about/guidelines.xhtml

16. Quam L, Achrekar A, Clay R. Saving mothers, giving life: A systems approach to reducing maternal and perinatal deaths in Uganda and Zambia. *Glob Health Sci Pract* 2019;7(Supplement 1):S1-S5.
17. Saya U, Wagner Z. The Impact of PEPFAR Funding on Child Morbidity and Mortality: Evidence from Sub-Saharan Africa (presentation) RAND Corporation, 2021 ImmunizationEconomics.org; July 12, 2021. Available: https://healtheconomics.confex.com/healtheconomics/2021/meetingapp.cgi/Paper/5219. [Accessed 7 Mar 2023].
18. Jakubowski A, Stearns SC, Kruk ME, *et al.* The US President's Malaria Initiative and under-5 child mortality in sub-Saharan Africa: A difference-in-differences analysis. *PLoS Med* 2017;14(6): e1002319.
19. Barnhart DA, Tsikhutsu I, Kirui D, *et al*. Association of the US President's Emergency Plan for AIDS Relief's Funding with Prevention of Mother-to-Child Transmission of HIV in Kenya. *JAMA Netw Open* 2019;2(9):e1911318.
20. PEPFAR 2022 Country Operational Plan and Regional (COP/ROP) for all PEPFAR Supported Guidance Countries. 2022 Available: https://www.state.gov/wp-content/uploads/2022/01/COP22-Guidance-Final_508-Compliant.pdf.[Accessed 26 Aug 2022].
21. PEPFAR Solutions Platform. CFM: Improving mother-baby pair retention in integrated maternal and child health and HIV services in Eswatini 2018 [18 Oct 2022]. Available: https://www.pepfarsolutions.org/solutions/2018/12/19/cfm-improving-mother-baby-pair-retention-in-interated-maternal-and-child-health-and-hiv-services-in-eswatini?rq=eswatini.
22. PEPFAR. Leveraging American Rescue Plan Act Funding to Support HIV and COVID-19 Responses. 2022 Available: https://www.state.gov/wp-content/uploads/2022/01/PEPFAR-ARPA-Funding-Fact-Sheet_2022.pdf. [Accessed 26 Aug 2022].
23. Institute of Medicine. Evaluation of PEPFAR. Washington, DC: The National Academies Press 2013. Available: https://www.nap.edu/catalog/18256/evaluation-of-pepfar. [Accessed 20 Dec 2021].
24. Lee B, Pan H, Ruhago G, *et al.* Applying Activity-Based Costing and Management to HIV Services in Tanzania: Improving Resource Allocation and Efficiency. Table 6. Washington, DC: Palladium, Health Policy Plus, 2022.
25. USAID, Ministry of Health Republic of Uganda, Uganda AIDS Commission. Uganda Health Systems Strengthening (UHSS) Activity. Applying Activity-Based Costing and Management (ABC/M) to HIV Services in Uganda: Improving Resource Allocation and Efficiency. 2022. Available: http://www.healthpolicyplus.com/ns/pubs/18589-19023_UgandaABCMReport.pdf. [Accessed 23 Sep 2022].
26. World Bank. World Development Indicators. 2021. Available: https://datatopics.worldbank.org/world-development-indicators/. [Accessed 14 Feb 2022].
27. USAID, US Department of State. Foreign Assistance.gov. Available: https://foreignassistance.gov/. [Accessed 18 October 2022].

For peer review only - http://bmjopen.bmj.com/site/about/guidelines.xhtml

28. Organizaiton for Economic Cooperation and Development (OECD). Creditor Reporting System (CRS). Available: https://stats.oecd.org/Index.aspx?DataSetCode=crs1. [Accessed 14 Feb 2022].
29. Institute for Health Metrics and Evaluation (IHME). GBD Results Tool. 2022. Available: http://ghdx.healthdata.org/gbd-results-tool. [Accessed 14 Feb 2022].
30. Salihu HM. Global reduction in HIV-related maternal mortality: ART as a key strategy. I*nt J MCH AIDS* 2015;4(2):8–10.
31. Duber HC, Coates TJ, Szekeras G, *et al*. Is there an association between PEPFAR funding and improvement in national health indicators in Africa? A retrospective study. *J Int AIDS Soc* 2010;13:21.

For peer review only

For peer review only - http://bmjopen.bmj.com/site/about/guidelines.xhtml

Tables

Table 1. Indicators analyzed in this study

Variable	Definition
DPT immunizations	Percent of children ages 12-23 months who received DPT vaccinations (3 doses)
HepB3 immunizations	Percent of children ages 12-23 months who received hepatitis B vaccines (3 doses)
Measles immunization	Percent of children ages 12-23 months who received the measles vaccination
Newborns protected against tetanus	Percentage of births by women of child-bearing age who are immunized against tetanus
Maternal mortality ratio	Number of women who die from pregnancy-related causes while pregnant or within 42 days of pregnancy termination per 100,000 live births
Child mortality rate	Probability of a child dying between birth and 5 years of age, per 1,000 live births
Prevalence of anemia among women of reproductive age	Prevalence of anemia among women of reproductive age (% of women ages 15-49)

Notes: DPT = diphtheria, whooping cough and tetanus; HepB3 = immunization against acute hepatitis B

For peer review only

Table 2. DID estimates of PEPFAR's impact on child immunizations in PEPFAR recipient countries, 2004-2018

	ALL PEPFAR	COP PEPFAR	Non-COP PEPFAR	High-Program Spending Intensity	Medium-Program Spending Intensity	Low-Program Spending Intensity
**DPT immunizations (% of children ages 12-23 months)**						
Mean (2004)	78.0	74.2	79.9	74.8	80.1	79.0
Model 1. DID model with no covariates, 1990-2018	8.421***	8.607***	8.221***	9.728***	5.467***	9.855***
Model 2. DID model with BL covariates only, 1990-2018[a]	8.893***	8.754***	8.985***	9.434***	7.090***	10.20***
Model 3. DID model with BL covariates and BL spending covariates (other donor spending and domestic health spending), 1990-2018[b]	8.810***	8.693***	8.889***	9.350***	6.999***	10.11***
% change using 2004 mean for the column[c]	10.8-11.4%	11.6-11.8%	10.3-11.2%	12.5-13.0%	6.8-8.9%	12.5-12.9%
**HepB3 immunizations (% of children ages 12-23 months)**						
Mean (2004)	79.1	74.4	81.7	80.7	78.3	78.5
Model 1. DID model with no covariates, 1990-2018	1.3740	8.027***	-2.0970	2.6610	2.1630	-0.6480
Model 2. DID model with BL covariates only, 1990-2018[a]	6.840***	13.73***	3.717*	8.179***	6.034**	5.612*
Model 3. DID model with BL covariates and BL spending covariates (other donor spending and domestic health spending), 1990-2018[b]	6.667***	13.64***	3.638*	7.875*	5.901**	5.385*
% change using 2004 mean for the column[c]	8.4-8.6%	10.8-18.5%	4.1-4.5%	9.8-10.1%	7.5-7.7%	6.9-7.1%
**Measles immunizations (% of children ages 12-23 months)**						

Mean (2004)	77.5	74.5	79.1	72.9	79.9	79.7
Model 1. DID model with no covariates, 1990-2018	6.644***	7.192***	6.260***	7.034***	4.287**	8.439***
Model 2. DID model with BL covariates only, 1990-2018[a]	7.281***	7.795***	6.862***	7.283***	5.645***	8.931***
Model 3. DID model with BL covariates and BL spending covariates (other donor spending and domestic health spending), 1990-2018[b]	6.946***	7.462***	6.560***	6.947***	5.339***	8.571***
% change using 2004 mean for the column[c]	8.6-9.4%	9.7-10.5%	7.9-8.7%	9.5-10.0%	5.4-7.1%	10.5-11.2%
**Newborns protected against tetanus (%)**						
Mean (2004)	75.7	75.6	75.8	75.1	78.3	74.2
Model 1. DID model with no covariates, 1990-2018	6.069***	4.775**	6.995***	6.154***	5.845***	6.059***
Model 2. DID model with BL covariates only, 1990-2018[a]	5.523***	3.779*	6.772***	5.427***	5.619***	5.522***
Model 3. DID model with BL covariates and BL spending covariates (other donor spending and domestic health spending), 1990-2018[b]	5.240***	3.736*	6.393***	5.338***	4.921**	5.398***
% change using 2004 mean for the column[c]	6.9-8.0%	4.9-6.3%	8.4-9.9%	7.1-8.2%	6.3-7.4%	7.3-8.2%

BL = baseline (2004); COP = country operational plans; DID = difference-in-difference; DPT = diphtheria, pertussis (whooping cough) and tetanus; HepB3 = immunization against acute hepatitis B; PEPFAR = President's Emergency Plan for AIDS Relief.
a Non-financial covariates include HIV prevalence rate (% of population ages 15-49), GDP per capita (constant USD), percent population urban, secondary school enrollment (% gross), life expectancy at birth, fertility rate (births per woman), and if the country received HIV aid from the U.S. prior to 2004. Disease prevalence rates are also baseline covariates in the DPT, Measles and HepB3 models.
b Financial covariates include other donor spending and domestic health spending.
c Approximate percentage reductions of the estimates shown. Range is low to high for significant coefficients.
***p < 0.001 **p < 0.01 * p< 0.05.

For peer review only - http://bmjopen.bmj.com/site/about/guidelines.xhtml

Table 3. DID estimates of PEPFAR's impact on maternal and child mortality and prevalence of anemia among women of reproductive age in PEPFAR recipient countries, 2004-2018

	ALL PEPFAR	COP PEPFAR	Non-COP PEPFAR	High-Program Spending Intensity	Medium-Program Spending Intensity	Low-Program Spending Intensity
**Maternal mortality ratio (modeled estimate, per 100,000 live births)**						
Mean (2004)	409.8	497.5	363.7	519.6	345.5	364.4
Model 1. DID model with no covariates, 2000-2017	-96.46***	-120.9***	-83.62**	-125.8***	-98.65***	-64.96
Model 2. DID model with BL covariates only, 2000-2017[a]	-101.1***	-131.3***	-85.77***	-136.0***	-100.4***	-69.31***
Model 3. DID model with BL covariates and BL spending covariates (other donor spending and domestic health spending), 2000-2017[b]	-100.7***	-130.9***	-85.40***	-135.7***	-100.0***	-68.94***
% change using 2004 mean for the column[c]	23.5-24.7%	23.4-26.4%	23.0-23.6%	24.2-26.1%	28.5-29.0%	18.9-19.0%
**Child mortality rate, under-5 (per 1,000 live births)**						
Mean (2004)	78.9	97.9	68.9	99.5	65.4	71.8
Model 1. DID model with no covariates, 1990-2018	-26.90***	-34.29***	-23.02***	-37.76***	-21.98***	-20.97***
Model 2. DID model with BL covariates only, 1990-2018[a]	-27.50***	-35.78***	-23.28***	-40.36***	-21.92***	-21.29***
Model 3. DID model with BL covariates and BL spending covariates (other donor spending and domestic health spending), 1990-2018[b]	-27.38***	-35.67***	-23.17***	-40.24***	-21.81***	-21.18***
% change using 2004 mean for the column[c]	34.1-35.0%	35.0-36.5%	33.4-33.8%	37.9-40.5	33.3-33.6%	29.2-29.6%

**Prevalence of anemia among women of reproductive age (% of women ages 15-49)**						
Mean (2004)	37.0	39.0	36.0	38.6	36.3	36.2
Model 1. DID model with no covariates, 1990-2018	-1.256	-1.908*	-0.914	-2.389*	-0.897	-0.482
Model 2. DID model with BL covariates only, 1990-2018[a]	-1.095	-1.653*	-0.812	-2.256**	-0.796	-0.324
Model 3. DID model with BL covariates and BL spending covariates (other donor spending and domestic health spending), 1990-2018[b]	-1.023	-1.581*	-0.740	-2.184**	-0.724	-0.252
% change using 2004 mean for the column[c]	ns	4.1-4.9%	ns	5.7-6.2%	ns	ns

BL = baseline (2004); COP = country Operational plans; DID = difference-in-difference; ns = not significant; PEPFAR = President's Emergency Plan for AIDS Relief.

a Non-financial covariates include HIV prevalence rate (% of population ages 15-49), GDP per capita (constant USD), percent population urban, secondary school enrollment (% gross), life expectancy at birth, fertility rate (births per woman), and if the country received HIV aid from the U.S. prior to 2004.

b Financial covariates include other donor spending and domestic health spending.

c Approximate percentage reductions of the estimates shown. Range is low to high for significant coefficients.

***$p < 0.001$ **$p < 0.01$ * $p< 0.05$.

For peer review only - http://bmjopen.bmj.com/site/about/guidelines.xhtml

For peer review only

For peer review only - http://bmjopen.bmj.com/site/about/guidelines.xhtml

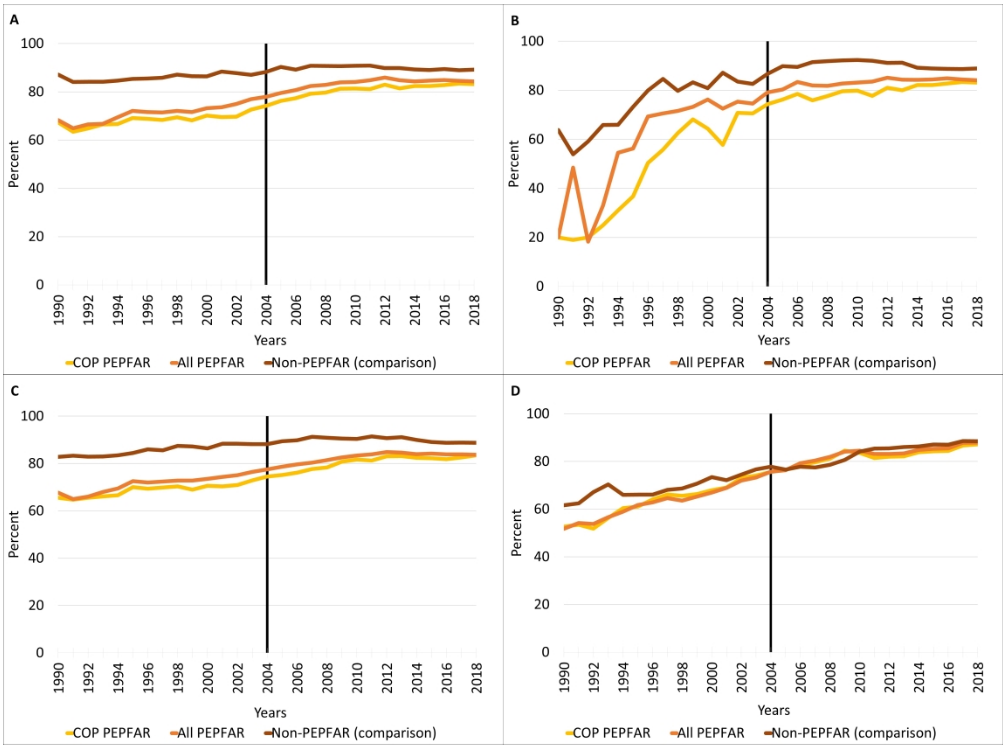

Figure 1. Trends in childhood immunizations in PEPFAR recipient countries, 1990-2018
(A) DPT immunizations, (B) HepB3 immunizations, (C) Measles immunizations, and (D) Newborns protected against tetanus

1371x1016mm (96 x 96 DPI)

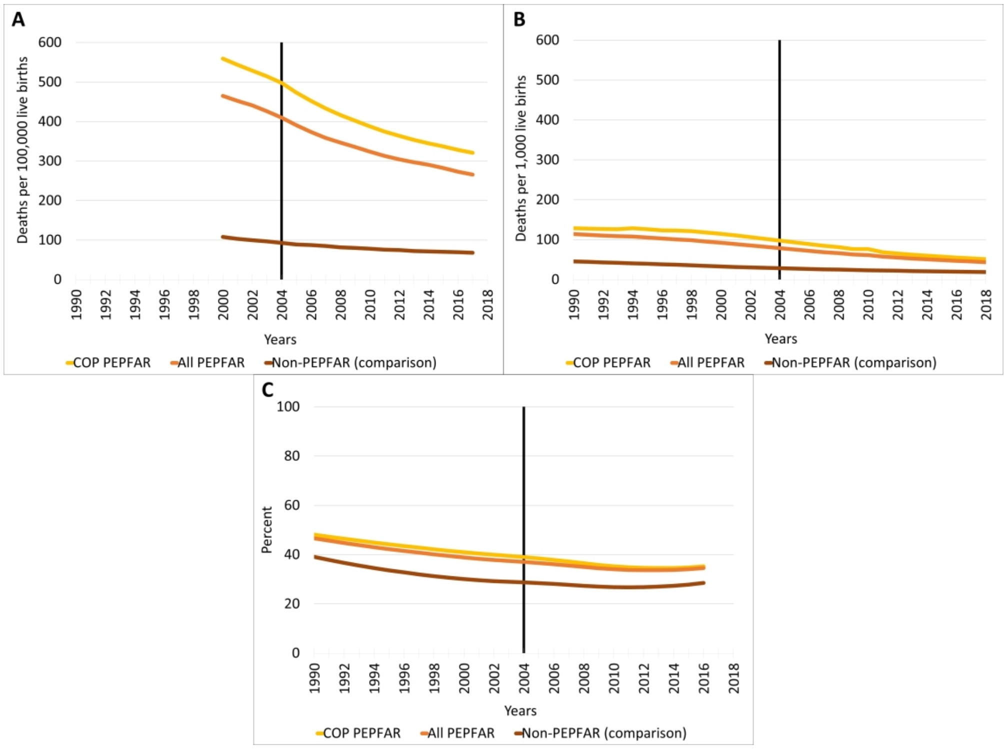

Figure 2. Trends in (A) Maternal mortality ratio, (B) Child mortality rate (under-5), and (C) Prevalence of anemia among women of reproductive age, in PEPFAR recipient countries, 1990-2018

1371x1016mm (96 x 96 DPI)

Health Spillover Impacts of PEPFAR
Supplemental Tables

Gary Gaumer[1], Dhwani Hariharan[1], William Crown[1], Jennifer Kates[2], Monica Jordan[1], Clare Hurley[1], AK Nandakumar[1]

[1]Institute for Global Health and Development, The Heller School for Social Policy and Management, Brandeis University, Waltham, MA, USA
[2] KFF, Global Health & HIV Policy, Washington, DC, USA

Supplement Number	Title
S1	DID method
S2	Country list
S3	Full model results for the indicator "Percent of children ages 12-23 months who received DPT vaccinations (3 doses)"
S4	Full model results for the indicator "Percentage of children age 12-23 months who received hepatitis B vaccines, 3 doses) "
S5	Full model results for the indicator "Percent of children ages 12-23 months who received the measles vaccination"
S6	Full model results for the indicator "Percentage of births by women of child-bearing age who are immunized against tetanus"
S7	Full model results for the indicator "Number of women who die from pregnancy-related causes while pregnant or within 42 days of pregnancy termination per 100,000 live births"
S8	Full model results for the indicator "Probability of a child dying between birth and 5 years of age, per 1,000 live births"
S9	Full model results for the indicator "Prevalence of anemia among women of reproductive age (% of women ages 15-49)"

S1. DID method

The Difference in Difference method provides a direct estimate of the impacts of PEPFAR on outcomes, based on the counterfactual provided by a control group of countries. DID can be thought of as an extension of Quasi Experimental Design to account for unobserved variables potentially correlated with both an intervention and the outcomes that are assumed to remain fixed over time. This DID method has been widely used in the program evaluation literature to estimate treatment effects as a non-parametric alternative to parametric sample selection models (Wooldridge, 2002). The method can be used when pre and post data are available for countries that received PEPFAR funding and for those that did not (eg. the control group). Characteristics of the control group countries were also measured in the baseline period. The first group of PEPFAR countries began receiving funding in late 2003, and the earliest health effects could first be measured in 2004 , the beginning of our followup period. We also measure the outcomes for countries in the control group in the same post-2004 period. If we assume that countries may also have unobserved characteristics, λ_i, that are correlated with outcomes and that these characteristics remain fixed over time (e.g., unobserved health endowment), DID provides a method to control for these fixed, unobserved characteristics.

To see this, we defined the outcome equations for periods 1 and 2, as shown in equations 1a and 1b, respectively:

(1a) $Y_{i1}=B_0 + B_1X_{i1}+B_2\lambda_i+\epsilon_{i1}$

(1b) $Y_{i2}=B_0 + B_1X_{i2}+B_2\lambda_i+B_3T_i+\epsilon_{i2}$

Calculating the change in outcomes and explanatory variables between time 1 and time 2, and re-estimating the outcome equation, is equivalent to subtracting equation (1a) from (1b):

(2) $(Y_{i2}- Y_{i1})= (B_0- B_0)+ B_1(X_{i2}- X_{i1})+ B_2 (\lambda_i- \lambda_i) +B_3T_i+(\epsilon_{i2}- \epsilon_{i1})$

Which simplifies to:

(3) $(Y_{i2}- Y_{i1})= B_1(X_{i2}- X_{i1})+B_3T_i+(\epsilon_{i2}- \epsilon_{i1})$

In other words, the DID approach subtracts out unobserved fixed effects of countries that may be correlated with both treatment selection and outcomes.

Operationally, the DID model is easy to implement using three dummy variables in a panel data set; (1) a time dummy captures the overall differences in the mean value of the dependent variable between the baseline period (pre 2004) and the follow-up period. (2) A dummy variable for PEPFAR countries and 0 for control group countries. (3) an interaction dummy between the first two dummy variables, the coefficient on which estimates the impact of PEPFAR.

S2. Country list

All PEPFAR Funded LMICs*	COPs	Other PEPFAR	High intensity PEFAR funding per capita group	Middle intensity PEPFAR funding per capita group	Low intensity PEPFAR funding per capita group	Control group (all LMICs)
Afghanistan	Angola	Afghanistan	Angola	Albania	Afghanistan	Algeria
Albania	Botswana	Albania	Botswana	Barbados	Armenia	American Samoa
Angola	Burundi	Armenia	Burundi	Belize	Bangladesh	Antigua and Barbuda
Armenia	Cambodia	Bangladesh	Cambodia	Benin	Bolivia	Argentina
Bangladesh	Cameroon	Barbados	Cameroon	Congo, Dem. Rep.	Brazil	Azerbaijan
Barbados	Congo, Dem. Rep.	Belize	Cote d'Ivoire	El Salvador	Burkina Faso	Belarus
Belize	Cote d'Ivoire	Benin	Djibouti	Eritrea	Central African Republic	Bhutan
Benin	Dominican Republic	Bolivia	Dominican Republic	Estonia	Chad	Bosnia and Herzegovina
Bolivia	Eswatini	Brazil	Eswatini	Gabon	China	Bulgaria
Botswana	Ethiopia	Burkina Faso	Ethiopia	Gambia, The	Comoros	Cabo Verde
Brazil	Ghana	Central African Republic	Ghana	Georgia	Congo, Rep.	Chile
Burkina Faso	Guyana	Chad	Guyana	Guatemala	Guinea-Bissau	Colombia
Burundi	Haiti	China	Haiti	Guinea	India	Costa Rica
Cambodia	India	Comoros	Honduras	Kyrgyz Republic	Indonesia	Croatia
Cameroon	Indonesia	Congo, Rep.	Jamaica	Lao PDR	Jordan	Cuba
Central African Republic	Kenya	Djibouti	Kenya	Madagascar	Kazakhstan	Czech Republic
Chad	Lesotho	El Salvador	Lesotho	Mali	Mauritania	Dominica
China	Malawi	Eritrea	Liberia	Moldova	Mexico	Ecuador
Comoros	Mozambique	Estonia	Malawi	Myanmar	Niger	Egypt, Arab Rep.
Congo, Dem. Rep.	Myanmar	Gabon	Mozambique	Nepal	North Macedonia	Equatorial Guinea
Congo, Rep.	Namibia	Gambia, The	Namibia	Nicaragua	Pakistan	Fiji
Cote d'Ivoire	Nigeria	Georgia	Nigeria	Papua New Guinea	Peru	Grenada
Djibouti	Papua New Guinea	Guatemala	Rwanda	Samoa	Philippines	Hungary
Dominican Republic	Rwanda	Guinea	Senegal	Sao Tome and Principe	Romania	Iran, Islamic Rep.
El Salvador	South Africa	Guinea-Bissau	South Africa	Sierra Leone	Russian Federation	Iraq
Eritrea	Tanzania	Honduras	Tanzania	Tajikistan	Seychelles	Kiribati
Estonia	Uganda	Jamaica	Uganda	Thailand	Suriname	Korea, Dem. People's Rep.
Eswatini	Ukraine	Jordan	Vietnam	Timor-Leste	Togo	Kosovo
Ethiopia	Vietnam	Kazakhstan	Zambia	Trinidad and Tobago	Turkmenistan	Latvia
Gabon	Zambia	Kyrgyz Republic	Zimbabwe	Ukraine	Uzbekistan	Lebanon
Gambia, The	Zimbabwe	Lao PDR				Libya
Georgia		Liberia				Lithuania
Ghana		Madagascar				Malaysia
Guatemala		Mali				Maldives
Guinea		Mauritania				Marshall Islands
Guinea-Bissau		Mexico				Mauritius
Guyana		Moldova				Mayotte
Haiti		Nepal				Micronesia, Fed. Sts.
Honduras		Nicaragua				Mongolia
India		Niger				Montenegro
Indonesia		North Macedonia				Morocco
Jamaica		Pakistan				Nauru
Jordan		Peru				Northern Mariana Islands
Kazakhstan		Philippines				Oman
Kenya		Romania				Palau
Kyrgyz Republic		Russian Federation				Panama
Lao PDR		Samoa				Paraguay
Lesotho		Sao Tome and Principe				Poland
Liberia		Senegal				Serbia
Madagascar		Seychelles				Serbia and Montenegro

S2. Country list

All PEPFAR Funded LMICs*	COPs	Other PEPFAR	High intensity PEFAR funding per capita group	Middle intensity PEPFAR funding per capita group	Low intensity PEPFAR funding per capita group	Control group (all LMICs)
Malawi		Sierra Leone				Slovak Republic
Mali		Suriname				Solomon Islands
Mauritania		Tajikistan				Somalia
Mexico		Thailand				Sri Lanka
Moldova		Timor-Leste				St. Kitts and Nevis
Mozambique		Togo				St. Lucia
Myanmar		Trinidad and Tobago				St. Vincent and the Grenadines
Namibia		Turkmenistan				Syrian Arab Republic
Nepal		Uzbekistan				Tonga
Nicaragua						Tunisia
Niger						Turkey
Nigeria						Tuvalu
North Macedonia						Uruguay
Pakistan						Vanuatu
Papua New Guinea						Venezuela, RB
Peru						West Bank and Gaza
Philippines						Yemen, Rep.
Romania						
Russian Federation						
Rwanda						
Samoa						
Sao Tome and Principe						
Senegal						
Seychelles						
Sierra Leone						
South Africa						
Suriname						
Tajikistan						
Tanzania						
Thailand						
Timor-Leste						
Togo						
Trinidad and Tobago						
Turkmenistan						
Uganda						
Ukraine						
Uzbekistan						
Vietnam						
Zambia						
Zimbabwe						

S3. Full model results for the indicator "Percent of children ages 12-23 months who received DPT vaccinations (3 doses)"

VARIABLES	All PEPFAR			COP			Other			H/M/L		
	Model 1	Model 2	Model 3	Model 1	Model 2	Model 3	Model 1	Model 2	Model 3	Model 1	Model 2	Model 3
Time variable (=1 post-2004)	3.783***	3.231***	3.308***	3.783***	3.242***	3.301***	3.783***	3.207***	3.293***	3.783***	3.225***	3.306***
	(0.828)	(0.553)	(0.555)	(0.762)	(0.497)	(0.499)	(0.805)	(0.523)	(0.524)	(0.826)	(0.551)	(0.553)
Intervention (=1 if PEPFAR)	-14.91***	-5.752***	-5.662***	-17.80***	-10.98***	-11.37***	-13.28***	-4.460***	-4.259***			
	(0.780)	(0.628)	(0.633)	(0.942)	(1.068)	(1.083)	(0.840)	(0.646)	(0.648)			
Intervention (intensity of PEPFAR funding per cap)												
Low										-16.08***	-6.599***	-6.502***
										(1.041)	(0.745)	(0.751)
Medium										-11.54***	-4.480***	-4.385***
										(1.062)	(0.769)	(0.772)
High										-16.88***	-3.094**	-2.836**
										(1.038)	(0.958)	(0.963)
Interaction term (PEPFAR impact estimate)	8.421***	8.893***	8.810***	8.607***	8.754***	8.693***	8.221***	8.985***	8.889***			
	(1.073)	(0.703)	(0.705)	(1.304)	(0.829)	(0.830)	(1.153)	(0.729)	(0.729)			
Interaction term (PEPFAR impact estimate)												
Low vs control										9.855***	10.20***	10.11***
										(1.435)	(0.923)	(0.925)
Medium vs control										5.467***	7.090***	6.999***
										(1.451)	(0.924)	(0.925)
High vs control										9.728***	9.434***	9.350***
										(1.434)	(0.944)	(0.946)
Country income level (=1 if middle income)		-4.907***	-5.021***		-6.910***	-6.596***		-2.990***	-2.787***		-4.709***	-4.813***
		(0.652)	(0.671)		(0.783)	(0.809)		(0.740)	(0.779)		(0.654)	(0.675)
BL Population		-1.68E-09	-1.91E-09		-7.77e-09***	-7.54e-09***		-1.63E-09	-2.31E-09		-7.15E-10	-9.38E-10
		(0.000)	(0.000)		(0.000)	(0.000)		(0.000)	(0.000)		(0.000)	(0.000)
Other donor spending (baseline)			0.00376			-0.0194*			-0.00417			0.0034
			(0.008)			(0.008)			(0.008)			(0.008)
Domestic spending (baseline)			-0.00460**			-0.00274			-0.00610***			-0.00511***
			(0.001)			(0.002)			(0.001)			(0.001)
BL GDP per capita, PPP (constant 2011 international $)		0.00000174	0.000123*		-0.0000498	-0.00000876		-0.0000054	0.000142**		0.00000532	0.000139**
		(0.000)	(0.000)		(0.000)	(0.000)		(0.000)	(0.000)		(0.000)	(0.000)
BL HIV prevalence (% of population ages 15-49)		0.784***	0.827***		0.726***	0.685***		-0.712**	-0.799**		0.658***	0.697***
		(0.064)	(0.067)		(0.074)	(0.078)		(0.254)	(0.263)		(0.070)	(0.072)

S3. Full model results for the indicator "Percent of children ages 12-23 months who received DPT vaccinations (3 doses)"

VARIABLES	All PEPFAR			COP			Other			H/M/L		
	Model 1	Model 2	Model 3	Model 1	Model 2	Model 3	Model 1	Model 2	Model 3	Model 1	Model 2	Model 3
BL diphtheria prevalence (% of population, under 5)		-427,897***	-391,197***		-515,342***	-515,995***		-490,406***	-439,209***		-451,760***	-413,221***
		(43187.000)	(45038.000)		(51643.000)	(54421.000)		(60584.000)	(63114.000)		(43455.000)	(45170.000)
BL tetanus prevalence (% of population, under 5)		-29,174***	-30,712***		44,495**	41,416**		-46,050***	-48,227***		-24,640***	-26,142***
		(8828.000)	(8845.000)		(14177.000)	(14296.000)		(9627.000)	(9631.000)		(8853.000)	(8864.000)
BL pertussis (whooping cough) prevalence (% of population, under 5)		-2,517***	-2,571***		-2,488***	-2,560***		-1,986***	-2,068***		-2,494***	-2,553***
		(96.300)	(98.300)		(107.800)	(111.500)		(110.100)	(111.700)		(96.300)	(98.220)
BL life expectancy at birth		0.596***	0.628***		0.380***	0.322***		0.425***	0.440***		0.582***	0.616***
		(0.054)	(0.056)		(0.071)	(0.077)		(0.059)	(0.063)		(0.054)	(0.056)
BL Urban population (%)		-0.0416***	-0.0285*		-0.0476***	-0.0393**		-0.0236	-0.00421		-0.0355**	-0.0208
		(0.012)	(0.013)		(0.013)	(0.014)		(0.013)	(0.013)		(0.012)	(0.013)
BL School enrollment, secondary (% gross)		0.0785***	0.0778***		0.0683***	0.0745***		0.0760***	0.0740***		0.0864***	0.0863***
		(0.014)	(0.014)		(0.016)	(0.017)		(0.014)	(0.015)		(0.014)	(0.014)
BL Fertility rate (births per woman)		-0.585*	-0.667**		-1.449***	-1.475***		-1.408***	-1.563***		-0.573*	-0.661*
		(0.255)	(0.258)		(0.325)	(0.331)		(0.280)	(0.285)		(0.255)	(0.258)
Recepient of US HIV aid before 2004 (=1 if yes)		-0.384	-0.577		0.417	0.226		-0.519	-1.001		-1.233*	-1.497**
		(0.446)	(0.453)		(0.740)	(0.743)		(0.518)	(0.530)		(0.485)	(0.493)
Constant	86.03***	57.54***	55.99***	86.03***	78.46***	82.86***	86.03***	67.71***	67.69***	86.03***	57.23***	55.58***
	(0.601)	(4.232)	(4.340)	(0.553)	(5.797)	(6.062)	(0.585)	(4.674)	(4.877)	(0.600)	(4.218)	(4.325)
Observations	4,282	3,939	3,926	2,628	2,341	2,328	3,386	3,100	3,087	4,282	3,939	3,926
R-squared	0.144	0.656	0.657	0.182	0.67	0.672	0.122	0.661	0.663	0.149	0.659	0.66

Standard errors in parentheses

*** p<0.001, ** p<0.01, * p<0.05

For peer review only - http://bmjopen.bmj.com/site/about/guidelines.xhtml

S4. Full model results for the indicator "Percentage of children age 12-23 months who received hepatitis B vaccines, 3 doses) "

	All PEPFAR			COP			Other			H/M/L		
	Model 1	Model 2	Model 3	Model 1	Model 2	Model 3	Model 1	Model 2	Model 3	Model 1	Model 2	Model 3
Time variable (=1 post-2004)	11.54***	11.24***	11.60***	11.54***	11.37***	11.55***	11.54***	11.23***	11.60***	11.54***	11.23***	11.61***
	(1.104)	(1.059)	(1.067)	(1.071)	(1.020)	(1.030)	(1.071)	(1.021)	(1.026)	(1.104)	(1.053)	(1.062)
Intervention (=1 if PEPFAR)	-8.247***	-5.353***	-5.161***	-18.39***	-20.87***	-20.57***	-2.948	-1.588	-1.352			
	(1.432)	(1.437)	(1.451)	(2.026)	(2.422)	(2.457)	(1.589)	(1.557)	(1.560)			
Intervention (intensity of PEPFAR funding per cap)												
Low										-6.256**	-2.208	-1.943
										(2.117)	(2.025)	(2.035)
Medium										-9.150***	-6.575***	-6.395***
										(1.928)	(1.815)	(1.824)
High										-9.384***	-2.021	-1.306
										(2.394)	(2.648)	(2.661)
Interaction term (PEPFAR impact estimate)	1.374	6.840***	6.667***	8.027***	13.73***	13.64***	-2.097	3.717*	3.638*			
	(1.638)	(1.540)	(1.545)	(2.273)	(2.094)	(2.102)	(1.802)	(1.673)	(1.674)			
Interaction term (PEPFAR impact estimate)												
Low vs control										-0.648	5.612*	5.385*
										(2.376)	(2.194)	(2.197)
Medium vs control										2.163	6.034**	5.901**
										(2.201)	(2.008)	(2.011)
High vs control										2.661	8.179***	7.875**
										(2.626)	(2.433)	(2.436)
Country income level (=1 if middle income)		-3.703**	-4.026***		-6.196***	-6.567***		-3.989**	-4.236**		-4.057***	-4.414***
		(1.149)	(1.199)		(1.484)	(1.548)		(1.277)	(1.365)		(1.150)	(1.200)
BL Population		-1.17e-08***	-1.21e-08***		-3.35e-08***	-3.39e-08***		-1.57E-09	-2.53E-09		-1.31e-08***	-1.35e-08***
		(0.000)	(0.000)		(0.000)	(0.000)		(0.000)	(0.000)		(0.000)	(0.000)
Other donor spending (baseline)			0.0161			0.01			0.0162			0.0182
			(0.013)			(0.013)			(0.013)			(0.013)
Domestic spending (baseline)			-0.00691*			-0.00178			-0.00983***			-0.00791**
			(0.003)			(0.003)			(0.003)			(0.003)
BL GDP per capita, PPP (constant 2011 international $)		0.000173**	0.000364***		0.000259***	0.000312**		0.000144*	0.000397***		0.000174**	0.000394***
		(0.000)	(0.000)		(0.000)	(0.000)		(0.000)	(0.000)		(0.000)	(0.000)
BL HIV prevalence (% of population ages 15-49)		0.641***	0.726***		0.751***	0.795***		-0.0918	-0.0293		0.452***	0.532***
		(0.115)	(0.120)		(0.132)	(0.140)		(0.534)	(0.545)		(0.127)	(0.131)
BL acute Hep B3 prevalence (% of population, under 5)		-7,563***	-7,376***		-9,085***	-8,809***		-6,252***	-5,927***		-7,882***	-7,669***
		(646.900)	(660.200)		(1114.000)	(1139.000)		(761.200)	(780.200)		(654.300)	(666.900)

For peer review only - http://bmjopen.bmj.com/site/about/guidelines.xhtml

S4. Full model results for the indicator "Percentage of children age 12-23 months who received hepatitis B vaccines, 3 doses) "

	All PEPFAR			COP			Other			H/M/L		
	Model 1	Model 2	Model 3	Model 1	Model 2	Model 3	Model 1	Model 2	Model 3	Model 1	Model 2	Model 3
BL life expectancy at birth		0.477***	0.544***		0.334*	0.385**		0.197	0.297*		0.467***	0.544***
		(0.101)	(0.107)		(0.134)	(0.146)		(0.110)	(0.121)		(0.101)	(0.106)
BL Urban population (%)		-0.0423*	-0.0208		-0.118***	-0.111***		-0.0301	0.00257		-0.0492*	-0.0244
		(0.021)	(0.023)		(0.025)	(0.027)		(0.022)	(0.024)		(0.022)	(0.023)
BL School enrollment, secondary (% gross)		0.0703**	0.0694**		0.0799***	0.0793**		0.0827**	0.0801**		0.0780***	0.0776**
		(0.024)	(0.024)		(0.030)	(0.030)		(0.025)	(0.025)		(0.024)	(0.024)
BL Fertility rate (births per woman)		-0.0822	-0.266		-0.718	-0.843		-1.843***	-2.202***		-0.188	-0.404
		(0.448)	(0.454)		(0.602)	(0.618)		(0.482)	(0.491)		(0.447)	(0.452)
Recepient of US HIV aid before 2004 (=1 if yes)		-0.946	-1.066		6.570***	6.564***		-1.317	-1.702		-1.999*	-2.237*
		(0.847)	(0.858)		(1.426)	(1.435)		(0.990)	(1.010)		(0.906)	(0.918)
Constant	78.67***	46.00***	41.62***	78.67***	62.65***	59.37***	78.67***	69.04***	62.93***	78.67***	47.18***	42.19***
	(0.918)	(7.847)	(8.160)	(0.891)	(10.890)	(11.500)	(0.891)	(8.618)	(9.193)	(0.919)	(7.822)	(8.130)
Observations	2,799	2,592	2,579	1,789	1,611	1,598	2,270	2,078	2,065	2,799	2,592	2,579
R-squared	0.094	0.29	0.292	0.159	0.356	0.357	0.072	0.277	0.283	0.094	0.298	0.301

Standard errors in parentheses
*** p<0.001, ** p<0.01, * p<0.05

ew only

S5. Full model results for the indicator "Percent of children ages 12-23 months who received the measles vaccination"

	All PEPFAR			COP			Other			H/M/L		
	**Model 1**	**Model 2**	**Model 3**	**Model 1**	**Model 2**	**Model 3**	**Model 1**	**Model 2**	**Model 3**	**Model 1**	**Model 2**	**Model 3**
Time variable (=1 post-2004)	4.348***	3.622***	3.968***	4.348***	3.635***	3.964***	4.348***	3.626***	3.954***	4.348***	3.619***	3.967***
	(0.796)	(0.595)	(0.589)	(0.723)	(0.547)	(0.537)	(0.789)	(0.559)	(0.554)	(0.794)	(0.594)	(0.588)
Intervention (=1 if PEPFAR)	-14.16***	-7.518***	-6.963***	-16.87***	-12.21***	-11.20***	-12.64***	-5.763***	-5.398***			
	(0.750)	(0.675)	(0.671)	(0.893)	(1.188)	(1.176)	(0.823)	(0.692)	(0.686)			
Intervention (intensity of PEPFAR funding per cap)												
Low										-14.91***	-8.191***	-7.481***
										(1.000)	(0.802)	(0.797)
Medium										-11.49***	-6.720***	-6.327***
										(1.021)	(0.829)	(0.820)
High										-15.92***	-5.788***	-5.011***
										(0.998)	(1.042)	(1.032)
Interaction term (PEPFAR impact estimate)	6.644***	7.281***	6.946***	7.192***	7.795***	7.462***	6.260***	6.862***	6.560***			
	(1.030)	(0.757)	(0.748)	(1.235)	(0.913)	(0.894)	(1.128)	(0.779)	(0.771)			
Interaction term (PEPFAR impact estimate)												
Low vs control										8.439***	8.931***	8.571***
										(1.378)	(0.996)	(0.983)
Medium vs control										4.287**	5.645***	5.339***
										(1.393)	(0.996)	(0.984)
High vs control										7.034***	7.283***	6.947***
										(1.376)	(1.019)	(1.006)
Country income level (=1 if middle income)		-4.850***	-5.948***		-6.402***	-7.716***		-3.362***	-4.429***		-4.852***	-5.986***
		(0.677)	(0.684)		(0.843)	(0.845)		(0.761)	(0.787)		(0.679)	(0.688)
BL Population		-4.13e-09**	-4.14e-09**		-9.06e-09***	-9.31e-09***		-1.53E-09	-1.69E-09		-3.94e-09**	-4.09e-09**
		(0.000)	(0.000)		(0.000)	(0.000)		(0.000)	(0.000)		(0.000)	(0.000)
Other donor spending (baseline)			0.0585***			0.0499***			0.0402***			0.0590***
			(0.008)			(0.008)			(0.008)			(0.008)
Domestic spending (baseline)			-0.00638***			-0.00652***			-0.00475**			-0.00673***
			(0.002)			(0.002)			(0.002)			(0.002)
BL GDP per capita, PPP (constant 2011 international $)		0.0000235	0.000253***		7.07e-05	0.000292***		0.000028	0.000185***		0.0000263	0.000266***
		(0.000)	(0.000)		(0.000)	(0.000)		(0.000)	(0.000)		(0.000)	(0.000)
BL HIV prevalence (% of population ages 15-49)		1.112***	1.208***		1.053***	1.162***		-0.507	-0.231		1.041***	1.130***
		(0.066)	(0.066)		(0.073)	(0.073)		(0.263)	(0.268)		(0.071)	(0.071)

S5. Full model results for the indicator "Percent of children ages 12-23 months who received the measles vaccination"

	All PEPFAR			COP			Other			H/M/L		
	Model 1	Model 2	Model 3	Model 1	Model 2	Model 3	Model 1	Model 2	Model 3	Model 1	Model 2	Model 3
BL measles prevalence (% of population, under 5)		-1,518***	-1,705***		-1,868***	-2,121***		-373.2**	-534.9***		-1,473***	-1,662***
		(124.800)	(126.800)		(153.100)	(156.100)		(143.000)	(144.900)		(125.700)	(127.400)
BL life expectancy at birth		0.740***	0.826***		0.591***	0.714***		0.522***	0.624***		0.740***	0.828***
		(0.057)	(0.057)		(0.074)	(0.076)		(0.063)	(0.065)		(0.057)	(0.057)
BL Urban population (%)		-0.0324*	-0.0128		-0.0803***	-0.0555***		0.0197	0.0322*		-0.0303*	-0.0103
		(0.013)	(0.013)		(0.014)	(0.015)		(0.013)	(0.014)		(0.013)	(0.013)
BL School enrollment, secondary (% gross)		0.0953***	0.0834***		0.0696***	0.0567***		0.137***	0.128***		0.0997***	0.0884***
		(0.014)	(0.014)		(0.017)	(0.017)		(0.015)	(0.015)		(0.014)	(0.014)
BL Fertility rate (births per woman)		-2.664***	-2.887***		-2.847***	-3.192***		-3.264***	-3.468***		-2.681***	-2.918***
		(0.263)	(0.261)		(0.340)	(0.338)		(0.282)	(0.281)		(0.263)	(0.261)
Recepient of US HIV aid before 2004 (=1 if yes)		2.165***	2.089***		2.918***	3.191***		3.182***	2.937***		1.737***	1.621**
		(0.475)	(0.474)		(0.797)	(0.782)		(0.542)	(0.549)		(0.517)	(0.516)
Constant	85.59***	41.38***	36.74***	85.59***	58.55***	51.92***	85.59***	49.58***	44.10***	85.59***	40.89***	36.16***
	(0.579)	(4.538)	(4.521)	(0.526)	(6.121)	(6.128)	(0.574)	(4.985)	(5.053)	(0.578)	(4.536)	(4.517)
Observations	4,275	3,939	3,926	2,621	2,341	2,328	3,379	3,100	3,087	4,275	3,939	3,926
R-squared	0.148	0.567	0.579	0.193	0.556	0.575	0.123	0.593	0.604	0.152	0.569	0.581

Standard errors in parentheses
*** p<0.001, ** p<0.01, * p<0.05

only

For peer review only - http://bmjopen.bmj.com/site/about/guidelines.xhtml

S6. Full model results for the indicator "Percentage of births by women of child-bearing age who are immunized against tetanus"

	All PEPFAR			COP			Other			H/M/L		
	Model 1	Model 2	Model 3	Model 1	Model 2	Model 3	Model 1	Model 2	Model 3	Model 1	Model 2	Model 3
Time variable (=1 post-2004)	14.23***	14.80***	14.93***	14.23***	15.04***	15.09***	14.23***	14.69***	14.83***	14.23***	14.77***	14.89***
	(1.155)	(1.144)	(1.124)	(1.138)	(1.093)	(1.090)	(1.175)	(1.143)	(1.119)	(1.155)	(1.142)	(1.122)
Intervention (=1 if PEPFAR)	-6.327***	0.481	0.63	-5.814***	-3.740*	-3.080	-6.706***	2.299	1.822			
	(0.984)	(1.149)	(1.139)	(1.130)	(1.762)	(1.778)	(1.094)	(1.254)	(1.254)			
Intervention (intensity of PEPFAR funding per cap)												
Low										-7.175***	-0.542	-0.435
										(1.236)	(1.294)	(1.284)
Medium										-4.992***	2.230	2.450
										(1.289)	(1.423)	(1.401)
High										-6.555***	2.640	2.756
										(1.154)	(1.527)	(1.510)
Interaction term (PEPFAR impact estimate)	6.069***	5.523***	5.240***	4.775**	3.779*	3.736*	6.995***	6.772***	6.393***			
	(1.350)	(1.323)	(1.300)	(1.557)	(1.477)	(1.472)	(1.499)	(1.432)	(1.403)			
Interaction term (PEPFAR impact estimate)												
Low vs control										6.059***	5.522***	5.398***
										(1.707)	(1.634)	(1.606)
Medium vs control										5.845***	5.619***	4.921**
										(1.757)	(1.681)	(1.653)
High vs control										6.154***	5.427***	5.338***
										(1.585)	(1.562)	(1.534)
Country income level (=1 if middle income)		0.0721	-0.0482		2.948*	2.648*		0.123	0.0849		0.381	0.212
		(1.104)	(1.092)		(1.346)	(1.346)		(1.465)	(1.470)		(1.107)	(1.094)
BL Population		3.7E-09	7.33e-09**		3.63E-09	4.18E-09		-2.47e-08*	3.24E-09		7.06e-09**	1.05e-08***
		(0.000)	(0.000)		(0.000)	(0.000)		(0.000)	(0.000)		(0.000)	(0.000)
Other donor spending (baseline)			0.481***			0.184**			0.478***			0.478***
			(0.050)			(0.063)			(0.056)			(0.050)
Domestic spending (baseline)			0.00673*			-0.00432			0.00927**			0.00722*
			(0.003)			(0.005)			(0.004)			(0.003)
BL GDP per capita, PPP (constant 2011 international $)		0.000440***	0.000399***		0.000527***	0.000653***		0.000531***	0.000427***		0.000431***	0.000380***
		(0.000)	(0.000)		(0.000)	(0.000)		(0.000)	(0.000)		(0.000)	(0.000)
BL HIV prevalence (% of population ages 15-49)		0.243*	0.0139		0.416***	0.351**		-1.832***	-1.602***		0.209	-0.0289
		(0.101)	(0.103)		(0.126)	(0.134)		(0.472)	(0.471)		(0.107)	(0.108)
BL life expectancy at birth		0.241**	0.0449		0.388**	0.325*		-0.340**	-0.481***		0.263**	0.0651
		(0.092)	(0.092)		(0.140)	(0.143)		(0.131)	(0.129)		(0.092)	(0.093)
BL Urban population (%)		-0.154***	-0.162***		-0.295***	-0.267***		-0.130***	-0.163***		-0.143***	-0.152***

S6. Full model results for the indicator "Percentage of births by women of child-bearing age who are immunized against tetanus"

	All PEPFAR			COP			Other			H/M/L		
	Model 1	Model 2	Model 3	Model 1	Model 2	Model 3	Model 1	Model 2	Model 3	Model 1	Model 2	Model 3
		(0.023)	(0.023)		(0.029)	(0.030)		(0.028)	(0.029)		(0.023)	(0.023)
BL School enrollment, secondary (% gross)		-0.0051	0.00306		-0.0628*	-0.043		-0.00633	0.0214		0.00285	0.0104
		(0.025)	(0.025)		(0.032)	(0.033)		(0.030)	(0.030)		(0.025)	(0.025)
BL Fertility rate (births per woman)		-2.610***	-3.190***		-2.666***	-2.920***		-5.430***	-5.454***		-2.366***	-2.955***
		(0.442)	(0.439)		(0.623)	(0.628)		(0.574)	(0.566)		(0.450)	(0.446)
Recepient of US HIV aid before 2004 (=1 if yes)		-0.023	0.828		5.996***	6.109***		-1.747*	-0.649		-1.309	-0.444
		(0.670)	(0.665)		(1.096)	(1.094)		(0.849)	(0.849)		(0.785)	(0.777)
Constant	68.91***	63.13***	73.74***	68.91***	60.04***	61.94***	68.91***	111.9***	115.9***	68.91***	59.65***	70.48***
	(0.843)	(7.312)	(7.325)	(0.831)	(11.290)	(11.400)	(0.858)	(10.350)	(10.150)	(0.843)	(7.390)	(7.392)
Observations	2,830	2,657	2,657	1,624	1,480	1,480	1,964	1,848	1,848	2,830	2,657	2,657
R-squared	0.265	0.362	0.385	0.235	0.362	0.367	0.258	0.387	0.413	0.267	0.365	0.387

Standard errors in parentheses
*** p<0.001, ** p<0.01, * p<0.05

For peer review only - http://bmjopen.bmj.com/site/about/guidelines.xhtml

S7. Full model results for the indicator "Number of women who die from pregnancy-related causes while pregnant or within 42 days of pregnancy termination per 100,000 live births"

	All PEPFAR			COP			Other			H/M/L		
	Model 1	Model 2	Model 3	Model 1	Model 2	Model 3	Model 1	Model 2	Model 3	Model 1	Model 2	Model 3
Time variable (=1 post-2004)	-23.62	-21.85	-22.22	-23.62	-21.85*	-22.22**	-23.62	-21.85	-22.22	-23.62	-21.85	-22.22
	(20.960)	(12.270)	(12.120)	(14.010)	(8.498)	(8.474)	(21.290)	(12.060)	(11.730)	(20.610)	(12.230)	(12.090)
Intervention (=1 if PEPFAR)	344.3***	124.4***	119.3***	434.5***	181.7***	173.1***	296.8***	109.3***	96.95***			
	(23.540)	(15.010)	(14.780)	(20.700)	(17.420)	(17.280)	(26.210)	(15.990)	(15.500)			
Intervention (intensity of PEPFAR funding per cap)												
Low										287.2***	113.3***	106.6***
										(30.770)	(18.620)	(18.290)
Medium										287.8***	108.4***	104.5***
										(30.770)	(18.680)	(18.350)
High										457.8***	154.4***	137.5***
										(30.770)	(21.540)	(21.180)
Interaction term (PEPFAR impact estimate)	-96.46***	-101.1***	-100.7***	-120.9***	-131.3***	-130.9***	-83.62**	-85.77***	-85.40***			
	(26.700)	(15.370)	(15.130)	(23.470)	(13.940)	(13.810)	(29.720)	(16.440)	(15.930)			
Interaction term (PEPFAR impact estimate)												
Low vs control										-64.96	-69.31***	-68.94***
										(34.890)	(20.060)	(19.700)
Medium vs control										-98.65**	-100.4***	-100.0***
										(34.890)	(19.850)	(19.500)
High vs control										-125.8***	-136.0***	-135.7***
										(34.890)	(20.520)	(20.150)
Country income level (=1 if middle income)		-20.14	-9.21		-68.14***	-54.34***		-2.465	8.032		-23.72*	-12.76
		(11.330)	(11.210)		(10.730)	(10.760)		(13.480)	(13.210)		(11.370)	(11.260)
BL Population		5.71E-09	1.61E-08		-1.91E-08	-5.3E-09		-3.53E-10	2.39E-08		-1.25E-08	-3.19E-09
		(0.000)	(0.000)		(0.000)	(0.000)		(0.000)	(0.000)		(0.000)	(0.000)
Other donor spending (baseline)			-1.030***			-0.590***			-0.976***			-1.021***
			(0.190)			(0.141)			(0.187)			(0.190)
Domestic spending (baseline)			0.235***			0.126***			0.287***			0.235***
			(0.025)			(0.021)			(0.026)			(0.025)
BL GDP per capita, PPP (constant 2011 international $)		0.000322	-0.00652***		0.00119*	-0.00247**		-0.000316	-0.00829***		0.000449	-0.00642***
		(0.001)	(0.001)		(0.001)	(0.001)		(0.001)	(0.001)		(0.001)	(0.001)
BL HIV prevalence (% of population ages 15-49)		-16.40***	-18.21***		-7.369***	-8.354***		2.702	3.454		-16.59***	-17.90***
		(1.098)	(1.090)		(0.954)	(0.954)		(4.721)	(4.559)		(1.194)	(1.177)
BL life expectancy at birth		-25.89***	-27.23***		-15.43***	-16.37***		-25.01***	-26.98***		-25.81***	-27.13***

S7. Full model results for the indicator "Number of women who die from pregnancy-related causes while pregnant or within 42 days of pregnancy termination per 100,000 live births"

	All PEPFAR			COP			Other			H/M/L		
	Model 1	Model 2	Model 3	Model 1	Model 2	Model 3	Model 1	Model 2	Model 3	Model 1	Model 2	Model 3
		(0.937)	(0.926)		(0.984)	(0.980)		(1.128)	(1.097)		(0.935)	(0.925)
BL Urban population (%)		0.753***	-0.042		0.352	-0.195		0.604*	-0.403		0.662**	-0.139
		(0.217)	(0.224)		(0.190)	(0.201)		(0.238)	(0.242)		(0.220)	(0.227)
BL School enrollment, secondary (% gross)		-1.390***	-1.340***		-0.873***	-0.846***		-2.031***	-1.851***		-1.365***	-1.341***
		(0.233)	(0.231)		(0.214)	(0.216)		(0.258)	(0.250)		(0.234)	(0.231)
BL Fertility rate (births per woman)		18.32***	24.85***		20.88***	26.97***		14.90**	25.77***		17.74***	24.37***
		(4.327)	(4.288)		(4.333)	(4.357)		(4.799)	(4.708)		(4.319)	(4.283)
Recepient of US HIV aid before 2004 (=1 if yes)		-71.39***	-66.19***		-26.60**	-27.82**		-66.07***	-49.84***		-68.99***	-60.91***
		(7.788)	(7.686)		(10.070)	(9.951)		(9.225)	(9.067)		(8.444)	(8.355)
Constant	101.8***	1,979***	2,057***	101.8***	1,236***	1,287***	101.8***	1,971***	2,070***	101.8***	1,980***	2,059***
	(18.490)	(73.940)	(72.810)	(12.350)	(79.100)	(78.390)	(18.780)	(87.120)	(84.290)	(18.170)	(73.750)	(72.640)
Observations	2,628	2,430	2,412	1,566	1,404	1,386	2,070	1,908	1,890	2,628	2,430	2,412
R-squared	0.197	0.762	0.773	0.45	0.811	0.817	0.155	0.763	0.782	0.225	0.763	0.774

Standard errors in parentheses
*** p<0.001, ** p<0.01, * p<0.05

For peer review only - http://bmjopen.bmj.com/site/about/guidelines.xhtml

S8. Full model results for the indicator "Probability of a child dying between birth and 5 years of age, per 1,000 live births"

	All PEPFAR			COP			Other			H/M/L		
	**Model 1**	**Model 2**	**Model 3**	**Model 1**	**Model 2**	**Model 3**	**Model 1**	**Model 2**	**Model 3**	**Model 1**	**Model 2**	**Model 3**
Time variable (=1 post-2004)	-14.45***	-14.50***	-14.62***	-14.45***	-14.50***	-14.62***	-14.45***	-14.50***	-14.62***	-14.45***	-14.50***	-14.62***
	(2.114)	(1.151)	(1.124)	(1.659)	(1.038)	(1.014)	(2.052)	(1.071)	(1.031)	(2.057)	(1.137)	(1.110)
Intervention (=1 if PEPFAR)	62.90***	17.56***	14.73***	82.70***	22.60***	17.64***	52.49***	13.75***	11.21***			
	(1.976)	(1.295)	(1.268)	(2.067)	(2.246)	(2.203)	(2.113)	(1.309)	(1.258)			
Intervention (intensity of PEPFAR funding per cap)												
Low										52.62***	15.88***	12.57***
										(2.590)	(1.528)	(1.492)
Medium										49.38***	12.94***	10.67***
										(2.590)	(1.558)	(1.515)
High										86.70***	25.08***	21.50***
										(2.590)	(1.958)	(1.907)
Interaction term (PEPFAR impact estimate)	-26.90***	-27.50***	-27.38***	-34.29***	-35.78***	-35.67***	-23.02***	-23.28***	-23.17***			
	(2.747)	(1.464)	(1.424)	(2.873)	(1.745)	(1.694)	(2.939)	(1.488)	(1.426)			
Interaction term (PEPFAR impact estimate)												
Low vs control										-20.97***	-21.29***	-21.18***
										(3.602)	(1.912)	(1.855)
Medium vs control										-21.98***	-21.92***	-21.81***
										(3.602)	(1.892)	(1.835)
High vs control										-37.76***	-40.36***	-40.24***
										(3.602)	(1.957)	(1.898)
Country income level (=1 if middle income)		-6.574***	-1.82		-1.938	2.627		-11.74***	-4.866***		-6.938***	-2.107
		(1.311)	(1.299)		(1.617)	(1.605)		(1.451)	(1.448)		(1.302)	(1.291)
BL Population		9.20e-09***	9.74e-09***		1.10e-08**	1.27e-08***		9.97e-09**	1.05e-08***		7.30e-09**	8.32e-09**
		(0.000)	(0.000)		(0.000)	(0.000)		(0.000)	(0.000)		(0.000)	(0.000)
Other donor spending (baseline)			-0.266***			-0.210***			-0.261***			-0.264***
			(0.016)			(0.015)			(0.015)			(0.016)
Domestic spending (baseline)			0.0148***			0.00285			0.0203***			0.0146***
			(0.003)			(0.003)			(0.003)			(0.003)
BL GDP per capita, PPP (constant 2011 international $)		0.000210***	-0.000482***		0.000382***	0.0000306		0.000181*	-0.000612***		0.000221**	-0.000469***
		(0.000)	(0.000)		(0.000)	(0.000)		(0.000)	(0.000)		(0.000)	(0.000)
BL HIV prevalence (% of population ages 15-49)		-2.052***	-2.428***		-1.565***	-1.969***		0.908	-0.694		-2.101***	-2.438***
		(0.126)	(0.124)		(0.139)	(0.138)		(0.504)	(0.494)		(0.136)	(0.133)
BL life expectancy at birth		-3.554***	-3.943***		-3.096***	-3.659***		-3.304***	-3.952***		-3.550***	-3.936***
		(0.106)	(0.105)		(0.139)	(0.141)		(0.117)	(0.117)		(0.105)	(0.104)
BL Urban population (%)		0.0483*	-0.00453		0.0803**	0.0526		-0.0265	-0.0839***		0.0398	-0.0104

S8. Full model results for the indicator "Probability of a child dying between birth and 5 years of age, per 1,000 live births"

	All PEPFAR			COP			Other			H/M/L		
	Model 1	Model 2	Model 3	Model 1	Model 2	Model 3	Model 1	Model 2	Model 3	Model 1	Model 2	Model 3
		(0.025)	(0.025)		(0.027)	(0.028)		(0.025)	(0.025)		(0.025)	(0.025)
BL School enrollment, secondary (% gross)		-0.262***	-0.206***		-0.343***	-0.274***		-0.247***	-0.194***		-0.258***	-0.205***
		(0.027)	(0.027)		(0.032)	(0.032)		(0.028)	(0.027)		(0.027)	(0.026)
BL Fertility rate (births per woman)		6.537***	7.443***		5.513***	6.159***		5.789***	7.046***		6.468***	7.392***
		(0.505)	(0.492)		(0.650)	(0.640)		(0.529)	(0.513)		(0.499)	(0.487)
Recepient of US HIV aid before 2004 (=1 if yes)		-1.393	-1.934*		5.404***	3.893**		-0.787	-0.604		-1.281	-1.726
		(0.913)	(0.890)		(1.532)	(1.486)		(1.024)	(0.995)		(0.980)	(0.958)
Constant	37.56***	294.4***	317.9***	37.56***	262.5***	298.1***	37.56***	285.9***	323.9***	37.56***	294.7***	317.9***
	(1.520)	(8.395)	(8.231)	(1.193)	(11.350)	(11.280)	(1.476)	(9.148)	(8.970)	(1.479)	(8.294)	(8.130)
Observations	4,408	4,031	4,002	2,697	2,378	2,349	3,509	3,190	3,161	4,408	4,031	4,002
R-squared	0.299	0.827	0.839	0.487	0.832	0.843	0.244	0.828	0.844	0.337	0.832	0.843

Standard errors in parentheses
*** p<0.001, ** p<0.01, * p<0.05

review only

For peer review only - http://bmjopen.bmj.com/site/about/guidelines.xhtml

S9. Full model results for the indicator "Prevalence of anemia among women of reproductive age (% of women ages 15-49)"

	All PEPFAR			COP			Other			H/M/L		
	**Model 1**	**Model 2**	**Model 3**	**Model 1**	**Model 2**	**Model 3**	**Model 1**	**Model 2**	**Model 3**	**Model 1**	**Model 2**	**Model 3**
Time variable (=1 post-2004)	-5.415***	-5.515***	-5.587***	-5.415***	-5.515***	-5.587***	-5.415***	-5.515***	-5.587***	-5.415***	-5.515***	-5.587***
	(0.567)	(0.443)	(0.437)	(0.489)	(0.431)	(0.425)	(0.581)	(0.411)	(0.406)	(0.566)	(0.442)	(0.436)
Intervention (=1 if PEPFAR)	8.469***	-0.208	-0.967*	10.39***	-1.126	-2.582**	7.461***	-2.316***	-2.707***			
	(0.505)	(0.481)	(0.475)	(0.575)	(0.907)	(0.898)	(0.568)	(0.483)	(0.477)			
Intervention (intensity of PEPFAR funding per cap)												
Low										7.287***	-0.75	-1.609**
										(0.673)	(0.568)	(0.561)
Medium										7.960***	-0.266	-0.904
										(0.673)	(0.581)	(0.571)
High										10.16***	-1.755*	-2.220**
										(0.673)	(0.734)	(0.723)
Interaction term (PEPFAR impact estimate)	-1.256	-1.095	-1.023	-1.908*	-1.653*	-1.581*	-0.914	-0.812	-0.740			
	(0.727)	(0.559)	(0.550)	(0.829)	(0.716)	(0.702)	(0.818)	(0.565)	(0.556)			
Interaction term (PEPFAR impact estimate)												
Low vs control										-0.482	-0.324	-0.252
										(0.970)	(0.734)	(0.720)
Medium vs control										-0.897	-0.796	-0.724
										(0.970)	(0.726)	(0.712)
High vs control										-2.389*	-2.256**	-2.184**
										(0.970)	(0.751)	(0.736)
Country income level (=1 if middle income)		-7.053***	-5.738***		-1.539*	-0.478		-9.552***	-7.546***		-7.064***	-5.706***
		(0.495)	(0.496)		(0.659)	(0.661)		(0.546)	(0.559)		(0.496)	(0.498)
BL Population		4.17e-09***	3.62e-09***		1.22e-08***	1.21e-08***		5.49E-10	-2.66E-10		3.82e-09***	3.51e-09***
		(0.000)	(0.000)		(0.000)	(0.000)		(0.000)	(0.000)		(0.000)	(0.000)
Other donor spending (baseline)			-0.0717***			-0.0633***			-0.0685***			-0.0725***
			(0.006)			(0.006)			(0.006)			(0.006)
Domestic spending (baseline)			-0.00834***			-0.00623***			-0.00420***			-0.00792***
			(0.001)			(0.001)			(0.001)			(0.001)
BL GDP per capita, PPP (constant 2011 international $)		0.000255***	0.000395***		0.000303***	0.000380***		0.000277***	0.000315***		0.000251***	0.000379***
		(0.000)	(0.000)		(0.000)	(0.000)		(0.000)	(0.000)		(0.000)	(0.000)
BL HIV prevalence (% of population ages 15-49)		-0.656***	-0.674***		-0.494***	-0.571***		1.309***	0.782***		-0.566***	-0.599***
		(0.048)	(0.048)		(0.057)	(0.057)		(0.190)	(0.191)		(0.052)	(0.052)
BL life expectancy at birth		-0.537***	-0.584***		-0.337***	-0.474***		-0.324***	-0.452***		-0.533***	-0.583***
		(0.040)	(0.040)		(0.058)	(0.058)		(0.044)	(0.045)		(0.040)	(0.040)
BL Urban population (%)		0.0196*	0.0352***		-0.0651***	-0.0543***		-0.0347***	-0.0240*		0.0176	0.0332***

For peer review only - http://bmjopen.bmj.com/site/about/guidelines.xhtml

S9. Full model results for the indicator "Prevalence of anemia among women of reproductive age (% of women ages 15-49)"

	All PEPFAR			COP			Other			H/M/L		
	**Model 1**	**Model 2**	**Model 3**	**Model 1**	**Model 2**	**Model 3**	**Model 1**	**Model 2**	**Model 3**	**Model 1**	**Model 2**	**Model 3**
		(0.010)	(0.010)		(0.012)	(0.012)		(0.010)	(0.010)		(0.010)	(0.010)
BL School enrollment, secondary (% gross)		-0.0741***	-0.0529***		-0.155***	-0.128***		-0.0815***	-0.0689***		-0.0783***	-0.0567***
		(0.010)	(0.010)		(0.013)	(0.013)		(0.011)	(0.011)		(0.010)	(0.010)
BL Fertility rate (births per woman)		1.162***	1.243***		0.322	0.329		1.462***	1.543***		1.181***	1.273***
		(0.191)	(0.188)		(0.267)	(0.264)		(0.199)	(0.198)		(0.191)	(0.188)
Recepient of US HIV aid before 2004 (=1 if yes)		-0.212	-0.777*		3.045***	2.547***		0.939*	0.361		0.321	-0.335
		(0.345)	(0.340)		(0.625)	(0.612)		(0.385)	(0.384)		(0.374)	(0.369)
Constant	32.99***	76.35***	79.22***	32.99***	69.62***	78.72***	32.99***	65.62***	73.80***	32.99***	76.46***	79.45***
	(0.394)	(3.188)	(3.155)	(0.339)	(4.681)	(4.678)	(0.403)	(3.467)	(3.479)	(0.393)	(3.180)	(3.148)
Observations	3,996	3,699	3,672	2,403	2,160	2,133	3,159	2,916	2,889	3,996	3,699	3,672
R-squared	0.162	0.572	0.592	0.242	0.505	0.53	0.137	0.644	0.661	0.166	0.574	0.594

Standard errors in parentheses
*** p<0.001, ** p<0.01, * p<0.05

review only

CHEERS 2022 Checklist

Analysis of Maternal and Child Health Spillover Effects in PEPFAR Countries: An Economic Evaluation

	Item	Guidance for Reporting	Reported in section
**TITLE**			
Title	1	Identify the study as an economic evaluation and specify the interventions being compared.	Title page 1
**ABSTRACT**			
Abstract	2	Provide a structured summary that highlights context, key methods, results and alternative analyses.	Abstract - Pages 2 and 3
**INTRODUCTION**			
Background and objectives	3	Give the context for the study, the study question and its practical relevance for decision making in policy or practice.	Introduction Page 4
**METHODS**			
Health economic analysis plan	4	Indicate whether a health economic analysis plan was developed and where available.	this is an econometric evaluation Page 5-6
Study population	5	Describe characteristics of the study population (such as age range, demographics, socioeconomic, or clinical characteristics).	Methods - Page 5 and Table 1
Setting and location	6	Provide relevant contextual information that may influence findings.	Methods - Page 5
Comparators	7	Describe the interventions or strategies being compared and why chosen.	Methods - Pages 5-6
Perspective	8	State the perspective(s) adopted by the study and why chosen.	Pages 5-6
Time horizon	9	State the time horizon for the study and why appropriate.	Page 5
Discount rate	10	Report the discount rate(s) and reason chosen.	NA
Selection of outcomes	11	Describe what outcomes were used as the measure(s) of benefit(s) and harm(s).	Methods - Page 6
Measurement of outcomes	12	Describe how outcomes used to capture benefit(s) and harm(s) were measured.	Methods - Page 6
Valuation of outcomes	13	Describe the population and methods used to measure and value outcomes.	Methods Page 5
Measurement and valuation of resources and costs	14	Describe how costs were valued.	NA
Currency, price date, and conversion	15	Report the dates of the estimated resource quantities and unit costs, plus the currency and year of conversion.	NA
Rationale and description of model	16	If modelling is used, describe in detail and why used. Report if the model is publicly available and where it can be accessed.	Methods – pages 5-6
Analytics and assumptions	17	Describe any methods for analysing or statistically transforming data, any extrapolation methods, and approaches for validating any model used.	Methods – pages 5-6
Characterizing heterogeneity	18	Describe any methods used for estimating how the results of the study vary for sub-groups.	Methods page 6
Characterizing distributional effects	19	Describe how impacts are distributed across different individuals or adjustments made to reflect priority populations.	NA
Characterizing uncertainty	20	Describe methods to characterize any sources of uncertainty in the analysis.	NA
Approach to engagement with patients and others affected by the study	21	Describe any approaches to engage patients or service recipients, the general public, communities, or stakeholders (e.g., clinicians or payers) in the design of the study.	NA
**RESULTS**			
Study parameters	22	Report all analytic inputs (e.g., values, ranges, references) including uncertainty or distributional assumptions.	Tables 2 and 3 and Supplement tables S3-S9
Summary of main results	23	Report the mean values for the main categories of costs and outcomes of interest and summarise them in the most appropriate overall measure.	Tables 2 and 3 and Supplement tables S3-S9
Effect of uncertainty	24	Describe how uncertainty about analytic judgments, inputs, or projections affect findings. Report the effect of choice of discount rate and time horizon, if applicable.	NA
Effect of engagement with patients and others affected by the study	25	Report on any difference patient/service recipient, general public, community, or stakeholder involvement made to the approach or findings of the study	Results page 7
**DISCUSSION**			
Study findings, limitations, generalizability, and current knowledge	26	Report key findings, limitations, ethical or equity considerations not captured, and how these could impact patients, policy, or practice.	Discussion pages 7-9
**OTHER RELEVANT INFORMATION**			
Source of funding	27	Describe how the study was funded and any role of the funder in the identification, design, conduct, and reporting of the analysis	Source of funding page

For peer review only - http://bmjopen.bmj.com/site/about/guidelines.xhtml

			10
Conflicts of interest	28	Report authors conflicts of interest according to journal or International Committee of Medical Journal Editors requirements.	Conflicts page 10

For peer review only

BMJ Open

Analysis of Maternal and Child Health Spillover Effects in PEPFAR Countries

Journal:	*BMJ Open*
Manuscript ID	bmjopen-2022-070221.R1
Article Type:	Original research
Date Submitted by the Author:	10-Nov-2023
Complete List of Authors:	Gaumer, Gary; Brandeis University Heller School for Social Policy and Management, Institute for Global Health and Development<br>Crown, William H; Brandeis University Heller School for Social Policy and Management, Institute for Global Health and Development<br>Kates, Jennifer; The Kaiser Family Foundation, Global Heallh and HIV Policy<br>Luan, Yiqun ; Brandeis University Heller School for Social Policy and Management, Institute for Global Health and Development<br>Hariharan, Dhwani; Brandeis University Heller School for Social Policy and Management, Institute for Global Health and Development<br>Jordan, Monica; Brandeis University Heller School for Social Policy and Management, Institute for Global Health and Development<br>Hurley, Clare; Brandeis University Heller School for Social Policy and Management, Institute for Global Health and Development<br>Nandakumar, Allyala; Brandeis University Heller School for Social Policy and Management, Institute for Global Health and Development
**Primary Subject Heading**:	Health economics
Secondary Subject Heading:	Health policy, Public health, HIV/AIDS
Keywords:	Health policy < HEALTH SERVICES ADMINISTRATION & MANAGEMENT, Health economics < HEALTH SERVICES ADMINISTRATION & MANAGEMENT, HIV & AIDS < INFECTIOUS DISEASES, QUALITATIVE RESEARCH, Health Services Accessibility, Public health < INFECTIOUS DISEASES

SCHOLARONE™
Manuscripts

I, the Submitting Author has the right to grant and does grant on behalf of all authors of the Work (as defined in the below author licence), an exclusive licence and/or a non-exclusive licence for contributions from authors who are: i) UK Crown employees; ii) where BMJ has agreed a CC-BY licence shall apply, and/or iii) in accordance with the terms applicable for US Federal Government officers or employees acting as part of their official duties; on a worldwide, perpetual, irrevocable, royalty-free basis to BMJ Publishing Group Ltd ("BMJ") its licensees and where the relevant Journal is co-owned by BMJ to the co-owners of the Journal, to publish the Work in this journal and any other BMJ products and to exploit all rights, as set out in our licence.

The Submitting Author accepts and understands that any supply made under these terms is made by BMJ to the Submitting Author unless you are acting as an employee on behalf of your employer or a postgraduate student of an affiliated institution which is paying any applicable article publishing charge ("APC") for Open Access articles. Where the Submitting Author wishes to make the Work available on an Open Access basis (and intends to pay the relevant APC), the terms of reuse of such Open Access shall be governed by a Creative Commons licence – details of these licences and which Creative Commons licence will apply to this Work are set out in our licence referred to above.

Other than as permitted in any relevant BMJ Author's Self Archiving Policies, I confirm this Work has not been accepted for publication elsewhere, is not being considered for publication elsewhere and does not duplicate material already published. I confirm all authors consent to publication of this Work and authorise the granting of this licence.

For peer review only

Analysis of Maternal and Child Health Spillover Effects in PEPFAR Countries

Gary Gaumer[1*], William Crown[1], Jennifer Kates[2],

Yiqun Luan[1], Dhwani Hariharan,[1]Monica Jordan[1], Clare Hurley[1], AK Nandakumar[1]

9 November, 2023

[1]Institute for Global Health and Development, The Heller School for Social Policy and Management, Brandeis University, Waltham, MA, USA

[2]KFF, Global Health & HIV Policy, Washington, DC, USA

*Corresponding Author: Gary Gaumer, PhD, Institute for Global Health and Development, Heller School for Social Policy and Management, Brandeis University, 415 South Street/MS035, Waltham, MA 02453, USA. E-mail: garygaumer@gmail.com

Word count for Introduction to References:

ABSTRACT

Objectives: This study examined whether the US President's Emergency Plan for AIDS Relief (PEPFAR) funding had effects beyond HIV, specifically on several measures of maternal and child health in low- and middle-income countries (LIMCs). The results of previous research on the question of PEPFAR health spillovers have been inconsistent. This study, using a large, multi-country panel data set of 157 low- and middle-income countries (LMICs) including 90 recipient countries, adds to the literature.

For peer review only

Design: Seven indicators including child and maternal mortality, several child vaccination rates, and anemia among childbearing-age women are important population health indicators. Panel data and difference-in-differences estimators (DID) were used to estimate the impact of the PEPFAR program from inception in 2004 to 2018 using a comparison group of 67 LMICs. Several different models of baseline (2004) covariates were used to help balance the comparison and treatment groups. Staggered DID was used to estimate impacts since all countries did not start receiving aid at PEPFAR's inception.

Setting: All 157 LMICs from 1990-2018.

Participants: 90 LMICs receiving PEPFAR aid and cohorts of those countries, including those required to submit annual country operational plans (COP), other recipient countries (non-COP), and three groupings of countries based on cumulative amount of per capita aid received (high, medium, low).

Interventions: PEPFAR aid to combat the HIV epidemic.

Primary outcome measures: Maternal mortality and child mortality rates, vaccination rates to protect children for DPT, measles, HepB3, and tetanus, and prevalence of anemia in women of childbearing age.

Results: Across PEPFAR recipient countries, large, favorable PEPFAR health effects were found for rates of childhood immunization, child mortality, and maternal mortality. These beneficial health effects were large and significant in all segments of PEPFAR recipient countries studied. We also found significant and favorable program effects on the prevalence of anemia in women of childbearing age in PEPFAR recipient countries receiving the most intensive financial support from the PEPFAR program. Other recipient countries did not demonstrate significant effects on anemia.

Conclusions: This study demonstrated that important health indicators, beyond HIV, have been consistently and favorably influenced by PEPFAR presence. Child and maternal mortality have been substantially reduced, and childhood immunization rates increased. We also found no evidence of "crowding out" or negative spillovers in these resource-poor countries. These findings add to the body of evidence that PEPFAR has had favorable health effects beyond HIV. The implications of these findings are that foreign aid for health in one area may have favorable health effects other areas in recipient countries. More research is needed on the influence of the mechanisms at work that create these spillover health effects of PEPFAR

Article Summary:

The literature is conflicted about whether PEPFAR may have had health effects beyond HIV. Using a panel data set for 157 low- and middle-income countries from 1990-2018, we examined

For peer review only

the relationship between PEPFAR funding and several measures of maternal and child health using difference in difference (DID) methods. Results show that across cohorts of PEPFAR recipient countries, favorable PEPFAR health spillover effects are consistently found for childhood immunization rates, child and maternal mortality rates, and prevalence of anemia in women of childbearing age. While earlier studies of spillovers offer conflicting findings, the results of this study show consistent evidence of positive health effects, beyond HIV, across the full history of PEPFAR in 90 recipient countries.

Strengths and Limitations:

- This study used long baseline and treatment periods (1990-2018) and both a traditional and staggered difference-in-difference research designs with differing model specifications that included specifications with just baseline covariates to better balance comparison and treatment groups, since randomization was not feasible.
- The impacts of PEPFAR were consistently favorable across six country cohorts we examined separately, though impacts were largest for countries where PEPFAR provided the most aid per capita, and where intense annual planning was required (COP).
- Despite the strength of the study design, there are still potential limitations; though we controlled for numerous baseline country differences, confirmed parallel baselines for treatment and comparison groups, and created estimates using two separate estimation approaches (traditional DID and staggered DID methods), we may have failed to control for other unobserved variables, which may have biased our estimates of the magnitude of PEPFAR impact on various outcomes.
- On the other hand, our comparison group included 18 countries that received very small and infrequent PEPFAR aid over the 2004-18 period (less than a $1M in aid over the period, or less than $.05 per capita). This may make our impact estimates of PEPFAR impact somewhat conservative.

For peer review only

Keywords: HIV, PEPFAR, low- and middle-income countries, spillover, impacts, panel dataset, maternal and child health

INTRODUCTION AND OBJECTIVES

The US President's Emergency Plan for AIDS Relief (PEPFAR) was authorized in 2003 to address the impact of HIV globally, and has become the largest commitment by any nation to address a single disease (now totaling more than $100B).[1] PEPFAR was signed into law by President G.W. Bush in 2003 to assist poor countries in stemming the HIV epidemic, and since then, over 100 countries have received assistance. Unlike many other aid programs, where funds

are transferred to country governments and they execute programs, the implementation of PEPFAR has always required joint planning and monitoring and partnership with countries.

PEPFAR assists countries by supporting free (to patients) programs for (1) testing for HIV, (2) treatment for persons living with HIV, (3) providing care and support for families affected by and living with HIV (4) training new health care workers, and (5) strengthening domestic health systems in recipient countries, among other services. Program officials report that PEPFAR has saved 25 million lives, supported 20 million persons with life-saving antiretroviral treatment (ARV), enabled 5.5 million babies to be born HIV-free through the reduction of HIV incidence and prevalence among women and men of child-bearing age.[2] Officials say the program has trained 340,000 health workers, provided critical care and support for 7 million vulnerable children and orphans and their caregivers, and provided services to more than 2.9M girls and young women.[3] Researchers have long confirmed the life-saving effects of PEPFAR activities.[4-9]

During the study period, 2004 to 2018, PEPFAR disbursed approximately $70 billion in bilateral aid to more than 100 low- and middle-income countries (LMICs).[10] While PEPFAR is credited with saving millions of lives and changing the trajectory of the HIV epidemic, there have been concerns and conflicting evidence about other health spillover effects (impacts on outcomes for health conditions other than HIV) of this very large, long lasting, and multifaceted program.

On the one hand, there have been concerns about negative spillover effects on other health sectors, given the program's significant size.[11-13] Some have argued that PEPFAR financing, including for the creation of new jobs, could, theoretically, "crowd out" the supply of staff and other resources to non-HIV health programs in poor countries.[14-22] If so, these negative spillover effects could potentially lead to worse, non-HIV related outcomes.[18-20] One such study,[18] using OECD Credit Reporting System (CRS) data,[23] found financial displacement effects within countries, showing that more HIV/AIDS funding was associated with a reduction in malaria program funding. Another study found HIV funding was associated with reduced provision of childhood immunizations in sub Saharan Africa (SSA).[15]

On the other hand, others have posited that PEPFAR could indirectly result in positive health spillover effects in other areas of health, particularly maternal and child health.[17] One confirming study of favorable impacts on maternal care in 257 facilities in eight African countries, found evidence that pregnant women who were not living with HIV were more likely to deliver in hospitals in areas that had more HIV-funded patient services or more HIV infrastructure support.[16] Another study examined 46 countries in SSA from 1995-2010, and found three of four vaccination indicators were positively associated with PEPFAR presence.[24] That study found, however, that PEPFAR was not significantly associated with improvements in infant mortality and under-five child mortality.[15] Another study of under-5 mortality also

For peer review only

failed to show significant reductions associated with PEPFAR.[25] One of these studies shows favorable impacts on child mortality by the President's Malaria Initiative in 19 countries in SSA from 2006 to 2014, but the PEPFAR covariate was found to be insignificant in influencing child mortality.[26] However, in a recent Kenya study of PMTCT impacts, the authors found favorable infant mortality declines associated with PEPFAR.[27]

Finally, two early African studies on spillover Health indicators show inconclusive results. In a 2000-2005 study of all African countries, 13 health indicators (vaccination rates, life expectancy at birth, sanitation, among others) showed no differences in trends between PEPFAR focus countries, and others.[28] A second early (2003-2008) DHS study of adults in nine PEPFAR focus countries (compared to 18 other countries in Africa) showed that all-cause mortality reductions attributed to PEPFAR were larger than the number of attributed HIV deaths. This difference, which would suggest spillovers in lives saved due to PEPFAR, were not statistically significant and deemed inconclusive by authors.[6]

These inconsistent findings of spillover effects on health warrant further analysis. Spillover significance, and direction are critical to health aid policymaking in the future. Following much of the extensive literature about spillovers of PEPFAR, we used several maternal child health (MCH) indicators to examine three research questions, using difference-in-differences (DID) analysis: (1) Do patterns of favorable MCH spillovers exist in a large group of 90 recipient countries over the PEPFAR operating period of 2004-2018? (2) Is the pattern of spillover effects across subgroups of countries consistent with differing intensities of PEPFAR planning and funding across recipient countries? (3) Are there any indications of negative spillover effects of PEPFAR?

Background on Possible Spillover Mechanisms

While PEPFAR was not intended to directly fund specific non-HIV maternal and child health services, its potential impact in this area seems likely, as reflected in some prior studies.[17 24 29 30]

This impact is possible given its significant investments, totaling more than $1 billion per year, in the health workforce, laboratory services, and various aspect of health systems strengthening.[11 31 32] These investments, in turn, may have had favorable health impacts through health care worker training (340,000 health workers trained), improved management, infrastructure, equipment, and supply chains, and may have even improved the operational culture and service quality in health facilities.[8] Further, PEPFAR supported creation of new medical education systems, a departure from its core areas of direct intervention in service delivery, and this probably "yielded large positive spillover benefits beyond HIV."[4] PEPFAR has also increasingly emphasized serving women at point of entry for prenatal care, as well as where they seek immunizations for their children, , allowing possible spillovers in this area.[11 12 33]

For peer review only

In addition, PEPFAR has supported the provision of HIV treatment, prevention, and testing services by supporting clinics and testing sites in previously medically underserved areas, and by so doing, exposed many households to professional medical care services for the first time. Indeed, emerging evidence from activity-based costing and management studies across multiple countries indicates that PEPFAR-funded clinicians, who are fully funded to do HIV work, are able to spend 30-40% of their time providing broader primary health care services at the facility level.[34 35] It seems likely that many or most of these non-HIV services would not have been provided to households in the absence of PEPFAR.

Finally, PEPFAR spending has also boosted the economy and jobs in recipient countries, as found by prior research.[36 37]This economic spillover may have created better health care access for households and contributed to other factors that could improve general health.

METHODS

Study design

Using a 29-year panel dataset (1990-2018) of 157 LMICs, we assessed the spillover impacts of PEPFAR on maternal and child health indicators. Ninety of these countries were PEPFAR recipients. The analysis makes estimates of PEPFAR impacts for six recipient cohorts of countries, each representing different intensities of PEPFAR funding and program oversight. These cohorts are (1) all 90 recipient countries taken together, (2) the 31 countries where countries participate in an annual country operational planning (COP) process with PEPFAR/OGAC staff, countries which generally have received more aid and, in many cases, have greater HIV burden, (3) other 59 recipient countries that do not prepare COPs, (4) the 30 countries with the highest cumulative PEPFAR aid (per capita) over the 2004-18 period; (5) the 30 countries with the lowest cumulative aid, and (6) the 30 countries with medium levels of cumulative aid. As we might expect with all aid programs, the early recipients were generally those with the highest HIV prevalence and the use of these cohorts, each studied separately, avoids the issue of endogeneity that might occur in pooling countries.

We assessed PEPFAR's impact by comparing country cohort outcomes to a comparison group of 67 LMICs that either did not receive any PEPFAR support or received minimal PEPFAR support (<$1M in total or <$0.05 per capita cumulatively between 2004 and 2018). **Supplement S1** provides lists of countries for each cohort and the comparison group.

Outcome variables

Seven indicators of maternal and child health were selected from the World Bank's World Development Indicators (WDI)[38] list. These indicators include four child immunization rate indicators (DPT, hepatitis B, and measles among children under age five; newborns protected against tetanus), the maternal mortality rate, child mortality rate, and the prevalence of anemia

For peer review only

among women of reproductive age (which is a risk factor for poor maternal and child birth outcomes). These indicators were selected because the child health indicators have been used in the literature to study PEPFAR spillover impacts and the maternal indicators are commonly used as indicators of maternal health. These indicators are defined in **Table 1**.

Covariates

We accounted for country-level time-invariant covariates to balance the characteristics between PEPFAR and comparison countries. Our emphasis on baseline covariates rather than time varying covariates was a deliberate decision that was made to avoid endogeneity problems. Causal inference regarding PEPFAR's impact on spillovers is derived from a model specification designed to implement the Rubin potential outcomes framework.[39] We intentionally used countries' baseline covariates in 2004 to avoid endogeneity issues. The covariates include gross domestic product (GDP) per capita, total population, life expectancy at birth, fertility rate, percent urban population of total population, gross enrollment rate for secondary education, HIV prevalence among the population ages 15-49, non-PEPFAR donor spending on health per capita, domestic health spending per capita, a dichotomous variable indicating whether a country received HIV funding from the U.S. prior to 2004, and a dichotomous variable indicating country income classifications. We also controlled for the prevalence of diphtheria, pertussis, tetanus (DPT), hepatitis B, and measles among the under-5 population when examining DPT, hepatitis B, and measles immunization rates as outcomes in our regressions. We obtained data on included covariates from five publicly accessible databases: the World Bank's World Development Indicators (WDI),[38] U.S. government's foreignassistance.gov database,[40] OECD Creditor Reporting System,[23] the United Nations Population Division,[41] and the Institute of Health Metrics and Evaluation GBD Result's Tool.[42] The rationale for including each covariate is specified in **Supplement S2**.

Statistical analysis

We employed both the traditional difference-in-difference method (traditional DID) and a staggered difference-in-difference panel event study approach (staggered DID) to estimate PEPFAR's impact on our selected MCH indicators. The traditional DID approach assesses the PEPFAR impact in recipient countries after PEPFAR began operating in 2004 and thereafter. The staggered DID approach refines the DID estimates to account for the country-specific year in which PEPFAR began to fund country operations.

For both traditional and staggered DID, in addition to using equations (1) and (3) below with adjusted covariates, we also estimated PEPFAR's impacts without adjusting for baseline covariates. Robust standard errors were calculated for all estimations. The full estimation results are shown in **Supplements S3-S6**.

For peer review only - http://bmjopen.bmj.com/site/about/guidelines.xhtml

Traditional DID. The traditional DID method has been widely used in the program evaluation literature to estimate treatment effects as a non-parametric alternative to parametric sample selection models.[43] The method can be used to estimate attribution to a treatment intervention, such as PEPFAR funding, using pre- and post-intervention data for both treatment and non-treatment countries (e.g., the comparison group). The traditional DID method creates an impact estimate by computing the pre-post change in the treatment group and subtracting from it the pre-post change in the comparison group. Econometrically, we used equation (1) to estimate PEPFAR's impact with the traditional DID method:

$$MCH_{it} = \beta_0 + \beta_1 PEPFAR_i + \beta_2 POST_t + \beta_3 PEPFAR_i * POST_t + \beta_4 X_{i,2004} + \varepsilon_{it} \quad (1)$$

where MCH_{it} is the outcome indicator for country i in year t; $PEPFAR_i$ is a dichotomous variable that equals one if country i is a PEPFAR recipient country and zero otherwise; $POST_t$ is a dichotomous variable that equals one for the year ≥ 2004 and zero otherwise; $X_{i,2004}$ is a set of country-level covariates in the baseline year 2004; ε_{it} is an error term. The key parameter of interest is β_3, representing the difference in pre-post changes between PEPFAR countries and comparison countries.

The assumption of parallel trends in outcomes before the intervention is essential for the internal validity of the traditional DID method. We assessed this assumption by examining the descriptive levels and trends of each outcome between PEPFAR and comparison countries at the aggregate level and empirically regressing the following equation:

$$MCH_{it} = \beta_0 + \beta_1 PEPFAR_i + \beta_2 YEAR_t + \beta_3 PEPFAR_i * YEAR_t + \varepsilon_{it} \quad (2)$$

where $YEAR_t$ entered as a set of dichotomous variables with 2004 as the reference year. The main parameter of interest is β_3, representing the moderating effect of country's PEPFAR status on the difference in outcomes for a given year relative to 2004. A series of statistically insignificant β_3s before 2004 suggested the satisfaction of the parallel trend assumption between PEPFAR and comparison countries. The tests confirmed parallel baselines for every dependent variable for every country cohort (see **Supplement S7**).

Staggered DID. We used staggered DID to account for when countries joined the PEPFAR program. The staggered DID approach generalizes the traditional DID method by considering country-specific participation year and allows for dynamic leads and lags to the participation to be estimated.[44] Equation (3) was used for staggered DID estimates:

$$MCH_{it} = \beta_0 + \beta_1 PEPFAR_i + \beta_2 D_{it} + \beta_3 X_{i,2004} + \gamma_t + \varepsilon_{it} \quad (3)$$

For peer review only

where D_{it} is a dichotomous variable that equals one for country i in each of its participation years and zero otherwise; β_2 is of our primary interest, measuring the impact of the presence of PEPFAR's support on recipient country MCH indicators; γ_t is a vector of year fixed effects dichotomous variables.

Specification testing of the staggered DID models was accomplished by estimating the dynamic impact of PEPFAR on each MCH outcome (see **Supplement S8** for the results). This investigation serves two purposes: first, to determine whether PEPFAR and comparison countries had similar pre-intervention outcome trends before recipient countries joined the program; and second, to track the evolution of PEPFAR's impact in terms of magnitude and statistical significance over time. We achieve this by substituting D_{it} in equation (3) with a set of country-specific dichotomous variables, D_{it}^{T}, indicating years relative to each country's PEFPAR initial participation year. Equation (4) was used:

$$MCH_{it} = \beta_0 + \beta_1 PEPFAR_i + \sum_{T=-10}^{15} \beta_T D_{it}^{T} + \delta X_{i,2004} + \gamma_t + \varepsilon_{it} \quad (4)$$

where D_{it}^{T} equals zero, except as following: D_{it}^{T} ($-10 \leq T \leq -1$) equals one for country i in its Tth year before participating the PEPFAR program, while D_{it}^{T} ($0 \leq T \leq 15$) equals one for country i in its Tth year after participating the PEPFAR program. A set of statistically insignificant D_{it}^{T} ($-10 \leq T \leq -1$) without a discernible trend would confirm a similar pre-intervention trend between PEPFAR and comparison countries, demonstrating the internal validity of the staggered DID design. Further, we used the year prior to initial participation as the baseline year and therefore excluded the dummy variable of D_{it}^{-1} from the equation. We followed previous studies[45] in using a 25-year window, which set D_{it}^{-10} equals one for all years that are 10 or more years before PEPFAR participation, while D_{it}^{15} equals one for all years that are 15 or more years after PEPFAR participation. Thus, PEPFAR's dynamic impacts estimated for these two endpoints may have greater variance and, consequently, less precision. Our results showed that in some situations the baseline assumptions of staggered DID were not met. In the case of immunization outcomes, this assumption was met for HepB vaccination, but only met for DPT, measles, and tetanus vaccination in the COP and high spending cohorts. While child mortality and anemia outcome models met the assumption, maternal mortality generally did not meet the assumption.

Lastly, we conducted two types of robustness tests. First, we did a placebo test to examine the robustness of PEPFAR impacts estimated from the staggered DID design. To conduct the test, we randomly assigned each recipient country a pseudo-policy-start year which is between 1990 and the country's actual PEPFAR participation year and used these randomly assigned years to run equation (3). We repeated the process 500 times. We examined the kernel density distribution of the 500 estimated β_2s against a normal distribution and plotted p-values of these 500 β_2s. A kernel density distribution with a mean close to zero and p-values being larger than

For peer review only - http://bmjopen.bmj.com/site/about/guidelines.xhtml

0.05 is indicative of good robustness of our staggered DID estimates with country actual participation years. **Supplement S9** shows that for MCH outcomes the mean of the estimated β_2 s with pseudo -policy-start years is close to zero, with the majority of the associated p-values being greater than 0.05. This suggests that the estimated PEPFAR impacts calculated using the actual PEPFAR participation years are not obtained by chance, confirming the robustness of the staggered DID estimates. Second, we logarithmically transformed the six outcomes and non-dichotomous covariates and regressed both the adjusted and unadjusted equations (1) and (3). We compared coefficients in terms of sign and significance with our non-logged estimates which are reported in **Tables 2 and 3**. The patterns of sign and significance of PEPFAR's impact in the logged models closely mirror the unlogged results reported in Table 2 and 3. (**Supplement S10**) We used Stata 18.0 (StataCorp LLC, College Station, TX) for all analyses.

RESULTS

1. Impact of PEPFAR on the four immunization rates

Figure 1 presents the levels and trends in the four immunization rates from 1990 to 2018 across all PEPFAR countries, COP countries, and comparison countries. PEPFAR recipient countries, particularly the COP countries, initially had lower (poorer) values for all four immunization rates compared to the comparison countries, but they demonstrated increasing immunization rates over time. In all cases, however, the improvement appears to be faster in PEPFAR countries than in comparison countries. The post PEPFAR uptake for DPT and measles is more pronounced than hepatitis B, and the situation is even less pronounced for tetanus than hepatitis B. In all cases, the pre-period trend pattern (before 2004) is generally the same in PEPFAR countries and comparisons.

Pre-2004 parallel trends are observed for all immunization rates and country cohorts in the traditional DID setting, with most 95% confidence intervals of β_3 in equation (2) before 2004 being insignificantly different from zero. However, for staggered DID the satisfaction of the essential pre-trend assumption was generally met, but with exceptions. Specifically, all the six cohorts fulfilled the assumption for the HepB rate, while only the COP and high spending cohorts satisfied the assumption for DPT, measles, and tetanus (see **Supplements S7-S8** for detail).

Table 2 reports the key estimation results from traditional DID (β_3 in equation 1) and staggered DID (β_2 in equation 3) for the six country cohorts. For each cohort of countries (6 columns) we report 4 models for each of the 4 immunization rate outcomes. The models include no covariates (Model 1) and covariates (Model 2) versions of the traditional and staggered DID methods.

For peer review only

Specifically, both econometric methods (traditional DID and staggered DID) suggest that PEPFAR's support has been associated with improvements in DPT, measles, and tetanus immunization rates for all the six country cohorts relative to what would have been expected in the absence of the program. The coefficients are interpreted as the changes in the dependent variable attributed to PEPFAR presence through 2018. Generally, the estimated coefficients are similar in magnitude, positive sign, and statistical significance across the adjusted and unadjusted specifications.

The single exception is that one of the models, for tetanus the coefficients for (Model 2 (adjusted) using the staggered DID specification), shows a positive coefficient for all cohorts, but the coefficient is insignificant for the cohort receiving the least amount of PEPFAR support.

For the hepatitis B immunization rate, PEPFAR's support results are somewhat inconsistent, with only 14 of 24 PEPFAR coefficients being positive and significant. But, the adjusted models generally show (10 out of 12 models) strong favorable PEPFAR impacts across cohorts, with larger coefficients for the staggered DID compared to the traditional DID. This pattern of somewhat inconclusive findings is suggested in the trend data in **Figure 1**. All the coefficients for the adjusted staggered model, and 4 of the 6 coefficients for adjusted model for traditional DID are significant (the exceptions are the cohorts of the non-COP countries and the cohort of medium levels of PEPFAR funding).

Other consistent patterns are evident in **Table 2**. Excepting the hepatitis B immunization rate, model 1 (no baseline covariates) has coefficients that are similar in size when compared to model 2 (with covariates). And results show consistency in size and significance of coefficients across cohorts, except for funding intensity: with medium-intensity cohorts, PEPFAR generally shows smaller impacts.

2. Impacts of PEPFAR on Maternal and Child Mortality Rates and Anemia in Women

Figure 2 describes the unadjusted trends in these outcomes. The maternal mortality rate for comparison countries remains relatively flat without improvement, whereas rates in PEPFAR countries improve and catch up to the comparison group (**Figure 2A**). The under-5 child mortality rate (**Figure 2B**) shows a very clear pre-2004 pattern of higher child mortality in PEPFAR recipient countries and then faster declines after 2004. In all PEPFAR and COP recipient countries, there were higher pre-2004 levels of under-5 mortality and even faster declines after 2004. Trends show poorer pre-2004 levels of anemia among women aged 15-49 in all PEPFAR countries (**Figure 2C**), and particularly in the COP country segments, with only slight evidence of convergence between PEPFAR and comparison countries.

For peer review only - http://bmjopen.bmj.com/site/about/guidelines.xhtml

All three outcomes in all six cohorts satisfied the parallel trend assumption in the traditional DID setting. However, in staggered DID, the pre-trend assumption was not met in certain cases. Specifically, the assumption was met for child mortality rates in COP and high-intensity countries (but not for the other cohorts). The assumption was met in all cohorts for anemia. Maternal mortality rates did not satisfy the assumption in all country cohorts, indicating the limited internal validity for the maternal mortality results in the staggered DID models. (See **Supplement S7-S8** for detail).

Table 3 shows that maternal mortality ratios and child mortality rates are substantially and consistently lower with the presence of PEPFAR. With the adjusted specifications (Model 2), the pattern generally shows larger reductions in maternal mortality ratios and child mortality rates for COP and high-intensity segments. Notably, these two segments also have much higher baseline maternal mortality ratios and child mortality rates compared to other recipient segments. But, Table 3 shows that PEPFAR has inconsistent effects on reducing the prevalence of anemia in women of reproductive age across different country cohorts. A statistically significant spillover benefit is only consistently observed in high-intensity country cohorts across all econometric methods and modeling specifications.

Here too, these modeling results in Table 3, excepting the prevalence of anemia among women, show consistency in PEPFAR influences across models (1 and 2) and across the traditional DID and staggered DID approaches.

DISCUSSION

Our assessment of PEPFAR's impact on several non-HIV/AIDS health measures in the areas of maternal and child health finds favorable consistent evidence of spillover effects for child immunization rates, maternal mortality, and child mortality. Though immunizations are not provided directly by PEPFAR, utilization rates for several childhood immunizations demonstrate positive spillover health benefits of PEPFAR presence in recipient countries. We estimate that four childhood immunizations were higher by 2018 than would have been expected in the absence of PEPFAR. The two earlier research studies were conflicting in their findings about immunization spillovers. Our study confirms one of these earlier and less comprehensive studies showing positive spillovers[24] and conflicts with the other prior study[15] showing negative effects using sub Saharan Africa data from 2003-2010.

We find large and significant impacts of PEPFAR on child mortality across all country segments and models. This confirms one earlier study done in Kenya[27] and differs from three earlier studies that all showed insignificant relationships between PEPFAR and child mortality.[24-26] Our findings are particularly strong for child and maternal mortality. Some of these reductions

For peer review only - http://bmjopen.bmj.com/site/about/guidelines.xhtml

are likely due to reductions in HIV-related mortality, but they appear to extend beyond this. For example, HIV accounted for 5.1% of the estimated 4 million child deaths in sub-Saharan Africa in 2003, compared to 1.6% of the estimated 2.8 million child deaths in 2018, a drop that would not fully drive the overall decline in child mortality we estimate.[30]

To our knowledge, PEPFAR effects on maternal mortality rates have not been studied before, though studies have confirmed mechanisms[17] for such effects, and confirmed that PEPFAR has improved obstetric care patterns.[16] Our estimates suggest PEPFAR has reduced maternal mortality. While the staggered DID estimates did not satisfy an important pre-period assumption of the method, the effects of the traditional DID satisfied the parallel baseline assumption, and show large and favorable impacts of PEPFAR. Since HIV is not considered to be a direct cause of maternal mortality, the spillover benefits of PEPFAR appear to be, as for child mortality, very substantial (HIV was estimated to have accounted for 6.4% of maternal deaths in sub-Saharan Africa between 2003 and 2009.[30]

We find evidence of favorable impacts on the prevalence of anemia in women of childbearing age in COP and highly-funded countries, but the favorable pattern is not seen in other country cohorts. No negative spillover effects were found for any of the seven outcomes we study here.

While our evidence of positive spillover effects on maternal and child health is notable and important, the mechanisms for such effects are not possible to isolate in our research. As noted earlier, one possible means is through PEPFAR's efforts to address HIV in pregnant women and children, since it brings women (and their children) into contact with formal health care services and providers.[46] A second, related mechanism may be the widespread scale-up of PEPFAR infrastructure that has created health-delivery system capacities to serve many isolated and previously underserved communities. This scale up often has been more rapid than the demand for HIV services, enabling and encouraging provision of other needed medical services in these locations.[34] More research is needed to document the source programmatic mechanisms of these and possibly other spillover benefits.

CONCLUSIONS

In addition to averting HIV deaths and limiting the spread of HIV, we find that PEPFAR funding has had other health benefits beyond HIV for recipient LMIC populations. While some early and focused studies reported instances of PEPFAR "crowding out" the supply of staff and other resources and evidence of negative spillover effects, this study, which includes a more comprehensive, multi-country data set, with observations obtained over a longer time period, did not identify evidence of any such effects.

This study demonstrates evidence of large, positive spillover effects in maternal mortality and child mortality and also shows consistent evidence of positive spillovers in childhood

For peer review only

immunization rates. Anemia in women shows favorable impacts but only in the COP and High Spend cohorts. These effects may stem from many aspects of the PEPFAR program, though this study did not examine potential mechanisms for such effects, which warrant further research.

Acknowledgments: The authors thank Yara Halasa-Rappel, Brandeis University, Waltham, MA, and Adam Wexler, KFF, Washington DC, for data retrieval and dataset preparation.

Contributors: GG, WC, JK and AKN led the study conception and design; GG, WC, JK, YL, DH, and MJ conducted the analysis and interpretation of results; GG, WC, JK, YL, DH, MJ, and CLH contributed to manuscript preparation; AKN was involved in funding acquisition and supervision. All authors reviewed the results and approved the final version of the manuscript. GG is the guarantor of this work.

Funding sources: This paper was produced with funding from Palladium International, LLC under subcontract number 217730-Brandeis-01; and Prime Contract number 2021-002516 from the Global Fund to Fight AIDS, Tuberculosis, and Malaria. It was also produced in part with funding from the Bill and Melinda Gates Foundation under grant INV-046299. Its contents are solely the responsibility of Brandeis University and do not necessarily represent the official views of the Bill and Melinda Gates Foundation, Palladium or The Global Fund.

Conflict of Interest: None declared.

Patient and public involvement: Patients or the public were not involved in the design, conduct, reporting, or dissemination plans of this research study.

Patient consent: Not applicable.

Ethics approval: Ethics approval was not required for this study.

Provenance and peer review: Not commissioned; externally peer reviewed.

Data availability: Our data came from five publicly available datasets: World Bank's World Development Indicators; U.S. government's foreignassistance.gov database; OECD Creditor Reporting System database; United Nations Population Division; and the Institute of Health Metrics and Evaluation GBD Result's Tool

Supplemental Material: This content has been supplied by the authors. It has not been vetted by BMJ Publishing Group Limited (BMJ) and may not have been peer-reviewed. Any opinions or recommendations discussed are solely those of the authors and are not endorsed by BMJ. BMJ disclaims all liability and responsibility arising from any reliance placed on the content. Where the content includes any translated material, BMJ does not warrant the accuracy and reliability of

For peer review only

the translations (including but not limited to local regulations, clinical guidelines, terminology, drug names and drug dosages), and is not responsible for any error and/or omissions arising from translation and adaptation or otherwise.

Open access: This is an open access article distributed in accordance with the Creative Commons Attribution 4.0 Unreported (CC BY 4.0) license, which permits others to copy, redistribute, remix, transform and build upon this work for any purpose, provided the original work is properly cited, a link to the license is given, and indication of whether changes were made. See: https://creativecommons.org/licenses/by/4.0/.

ORCID IDs:

Gary Gaumer: https://orcid.org/0000-0003-3304-1258

William Crown: https://orcid.org/0000-0002-8822-2737

Yiqun Luan: https://orcid.org/ 0000-0001-7275-1848

Dhwani Hariharan: https://orcid.org/0000-0001-7329-7376

Monica Jordan: https://orcid.org/0000-0003-4080-0267

Allyala Nandakumar: https://orcid.org/0000-0002-0976-9313

Author email addresses:

Gary Gaumer: garygaumer@gmail.com

William Crown: wcrown@brandeis.edu

Jennifer Kates: jkates@kff.org

Yiqun Luan: yluan@brandeis.edu

Dhwani Hariharan: dhwanihariharan@brandeis.edu

Monica Jordan: mjordan@brandeis.edu

Clare Hurley: churley@brandeis.edu

Allyala Nandakumar: aknkumar@brandeis.edu

For peer review only

REFERENCES

1. PEPFAR. The United States President's Emergency Plan for AIDS Relief 2021 Annual Report to Congress, 2021. Available: https://www.state.gov/wp-content/uploads/2021/02/PEPFAR2021AnnualReporttoCongress.pdf.
2. PEPFAR. PEPFAR's Latest Global Results, 2022. Available: https://www.state.gov/wp-content/uploads/2022/07/PEPFAR-Latest-Global-Results_JULY-2022_FINAL.pdf.
3. US Department of State. Results and Impact - PEPFAR, 2022. Available: https://www.state.gov/results-and-impact-pepfar/
4. Bendavid E. Past and future performance: PEPFAR in the landscape of foreign aid for health. *Curr HIV/AIDS Rep* 2016;13(5):256–62. doi:10.1007/s11904-016-0326-8
5. Bendavid E, Bhattacharya J. The President's Emergency Plan for AIDS Relief in Africa: An evaluation of outcomes. *Ann Intern Med* 2009;150(10):688–95. doi: 10.7326/0003-4819-150-10-200905190-00117.
6. Bendavid E, Holmes CB, Bhattacharya J, et al. HIV development assistance and adult mortality in Africa. *JAMA* 2012;307(19):2060-67. doi:10.1001/jama.2012.2001
7. Heaton LM, Bouey PD, Fu J, et al. Estimating the impact of the US President's Emergency Plan for AIDS Relief on HIV treatment and prevention programmes in Africa. *Sex Transm Infect* 2015;91(8):615-20. doi: 10.1136/sextrans-2014-051991
8. Institute of Medicine. Evaluation of PEPFAR. Washington, DC: The National Academies Press, Washington, DC: The National Academies Press; 2013. Available: https://www.nap.edu/catalog/18256/evaluation-of-pepfar.
9. Kim Y. The effectiveness of PEPFAR's funding for women and children with HIV/AIDS. *Int J Health Plann Manage* 2019;34(1):e896-e916. doi: 10.1002/hpm.2706
10. Kaiser Family Foundation. The U.S. President's Emergency Program for Aids Relief, Key Facts, 2023. Available: https://www.kff.org/global-health-policy/fact-sheet/the-u-s-presidents-emergency-plan-for-aids-relief-pepfar/#:~:text=PEPFAR%20is%20the%20largest%20commitment,government%20is%20the%20largest%20donor.
11. Kates J, Gaumer G, Crown W, et al. Assessing PEPFAR's Impact: Analysis of Maternal and Child Health Spillover Effects in PEPFAR Countries, 2022. Available: https://www.kff.org/global-health-policy/issue-brief/assessing-pepfars-impact-analysis-of-maternal-and-child-health-spillover-effects-in-pepfar-countries/.
12. PEPFAR Solutions Platform. CFM: Improving mother-baby pair retention in integrated maternal and child health and HIV services in Eswatini, 2018. Available: https://www.pepfarsolutions.org/solutions/2018/12/19/cfm-improving-mother-baby-pair-retention-in-interated-maternal-and-child-health-and-hiv-services-in-eswatini?rq=eswatini
13. US State Department. PEPFAR 2022 Country and Regional Operational Plan (COP/ROP) Guidance for all PEPFAR-Supported Countries. Available:

For peer review only

https://www.state.gov/wpcontent/uploads/2022/01/COP22-Guidance-Final_508-Compliant.pdf.

14. Garrett L. The challenge of global health. *Foreign Aff* 2007;Jan/Feb:14-38. doi: https://www.jstor.org/stable/20032209
15. Grépin KA. HIV donor funding has both boosted and curbed the delivery of different non-HIV health services in sub-Saharan Africa. *Health Aff (Millwood)* 2012;31(7):1406-14. doi: 10.1377/hlthaff.2012.0279
16. Kruk ME, Jakubowski A, Rabkin M, et al. PEPFAR programs linked to more deliveries in health facilities by African women who are not infected with HIV *Health Aff (Millwood)* 2012;31(7):1478-88. doi: 10.1377/hlthaff.2012.0197
17. Leeper SC, Reddi A. United States global health policy: HIV/AIDS, maternal and child health, and The President's Emergency Plan for AIDS Relief (PEPFAR). *AIDS* 2010;24(14):2145-9. doi: 10.1097/QAD.0b013e32833cbb41
18. Lordan G, Tang KK, Carmignani F. Has HIV/AIDS displaced other health funding priorities? Evidence from a new dataset of development aid for health. *Soc Sci Med* 2011;73(3):351-5. doi: 10.1016/j.socscimed.2011.05.045
19. McCoy D, Chopra M, Loewenson R, et al. Expanding access to anti-retroviral therapy in sub-Saharan Africa, avoiding pitfalls and dangers, capitalizing on the opportunities. *Am J Pub Health* 2005;95(1):18–22. doi: 10.2105/AJPH.2004.040121
20. Shiffman J. Has donor prioritization of HIV/AIDS displaced aid for other health issues? *Health Policy Plan* 2008;23(2):95–100. doi: https://doi.org/10.1093/heapol/czm045
21. Walensky RP, Kuritzkes DR. The impact of the President's Emergency Plan for AIDS Relief (PEPFAR) beyond HIV and why it remains essential. *Clin Infect Dis* 2010;50(2):272–75. doi: https://doi.org/10.1086/649214
22. Walsh A, Ndubani P, Simbaya J, et al. Task sharing in Zambia: HIV service scaleup compounds the human resource crisis. *BMC Health Serv Res* 2010;10(1):272. doi: https://doi.org/10.1186/1472-6963-10-272

[dataset] 23. Organization for Economic Cooperation and Development (OECD). Creditor Reporting System (CRS) Accessed: 22 June 2022. Available: https://stats.oecd.org/Index.aspx?DataSetCode=crs1.

24. Cohen RL, Li Y, Giese R, et al. An evaluation of the President's Emergency Plan for AIDS Relief effect on health systems strengthening in sub-Saharan Africa. *J Acquir Immune Defic Syndr* 2013;62(4):471-9. doi: 10.1097/QAI.0b013e3182816a86
25. Saya U, Wagner Z. The Impact of PEPFAR Funding on Child Morbidity and Mortality: Evidence from Sub-Saharan Africa (presentation) RAND Corporation, 2021
26. Jakubowski A, Stearns SC, Kruk ME, et al. The US President's Malaria Initiative and under-5 child mortality in sub-Saharan Africa: A difference-in-differences analysis. *PLoS Med* 2017;14(6): e1002319. doi: https://doi.org/10.1371/journal.pmed.1002319
27. Barnhart DA, Tsikhutsu I, Kirui D, et al. Association of the US President's Emergency Plan for AIDS Relief's funding with prevention of mother-to-child transmission of HIV in

For peer review only

Kenya. *JAMA Netw Open* 2019;2(9):e1911318. doi: 10.1001/jamanetworkopen.2019.11318

28. Duber HC, Coates TJ, Szekeras G, et al. Is there an association between PEPFAR funding and improvement in national health indicators in Africa? A retrospective study. *J Int AIDS Soc* 2010;13:21. doi: 10.1186/1758-2652-13-21
29. Kruk ME, Rabkin M, Grépin KA, et al. 'Big Push' to reduce maternal mortality In Uganda and Zambia enhanced health systems but lacked a sustainability plan. *Health Aff (Millwood)* 2014;66(6):1058-66. doi: 10.1377/hlthaff.2013.0637
30. Salihu HM. Global reduction in HIV-related maternal mortality: ART as a key strategy. *Int J MCH AIDS* 2015;4(2):8–10.
31. PEPFAR. Leveraging American Rescue Plan Act Funding to Support HIV and COVID-19 Responses 2022. Available: https://www.state.gov/wp-content/uploads/2022/01/PEPFAR-ARPA-Funding-Fact-Sheet_2022.pdf.
32. Quam L, Achrekar A, Clay R. Saving mothers, giving life: A systems approach to reducing maternal and perinatal deaths in Uganda and Zambia. *Glob Health Sci Pract* 2019;7(Supplement 1):S1-S5. doi: 10.9745/GHSP-D-19-00037
33. PEPFAR. County Opperational Plan Guide - Final Draft for USG Distribution Only, 2008. Available: https://www.kff.org/wp-content/uploads/2021/12/PEPFAR-FY2009-COP-Guidance-Final.pdf.
34. Lee B, Pan H, Ruhago G, et al. Applying Activity-Based Costing and Management to HIV Services in Tanzania: Improving Resource Allocation and Efficiency. Table 6. Washington, DC: Palladium, Health Policy Plus, 2022.
35. USAID, Ministry of Health Republic of Uganda, Uganda AIDS Commission. Uganda Health Systems Strengthening (UHSS) Activity. Applying Activity-Based Costing and Management (ABC/M) to HIV Services in Uganda: Improving Resource Allocation and Efficiency, 2022. Available: http://www.healthpolicyplus.com/ns/pubs/18589-19023_UgandaABCMReport.pdf.
36. Crown W, Hariharan D, Kates J, et al. Analysis of economic and educational spillover effects in PEPFAR countries. *PLoS One* Submitted
37. Wagner Z, Barofsky J, Sood N. PEPFAR funding associated with an increase in employment among males in ten sub-Saharan African countries. *Health Aff (Millwood)* 2015;34(6):946-53. doi: 10.1377/hlthaff.2014.1006

[dataset] 38. World Bank. World Development Indicators, 2021. Accessed 22 June 2022. Available: https://datatopics.worldbank.org/world-development-indicators/.

39. Imbens GW. Statistical significance, p-values, and the reporting of uncertainty. *J Econ Perspect* 2021;35(3):157–74. doi: https://doi.org/10.1257/jep.35.3.157.

[dataset] 40. USAID, US Department of State. Foreign Assistance.gov. Accessed 22 June 2022. Available: https://www.foreignassistance.gov/.

For peer review only

[dataset] 41. United Nations. Department of Economic and Social Affairs. Population Division: World Population Prospects. Accessed 22 June 2022. Available: https://population.un.org/wpp/.

[dataset] 42. Institute for Health Metrics and Evaluation (IHME). GBD Results Tool, 2022. Accessed 22 June 2022. Available: http://ghdx.healthdata.org/gbd-results-tool.

43. Wooldridge J. Econometric Analysis of Cross Section and Panel Data. Cambridge, Massachusetts: MIT Press 2002.

44. Clarke D, Tapia-Schythe K. Implementing the panel event study. *Stata J* 2021;21(4):853-84. doi: IZA DP No. 13524

45. Beck T, Levine R, Levkov A. Big bad banks? The winners and losers from bank deregulation in the United States. *J Finance* 2010;65(5):1637-67. doi: https://www.jstor.org/stable/40864982

46. PEPFAR. Country and Regional Operational Plan (COP/ROP) Guidance for all PEPFAR Countries PEPFAR,; 2021. Available: https://www.state.gov/wp-content/uploads/2022/01/COP22-Guidance-Final_508-Compliant.pdf

For peer review only

TABLES

Table 1. Indicators analyzed in this study

Variable	Definition
DPT immunizations	Percent of children ages 12-23 months who received DPT vaccinations (3 doses)
HepB3 immunizations	Percent of children ages 12-23 months who received hepatitis B vaccines (3 doses)
Measles immunization	Percent of children ages 12-23 months who received the measles vaccination
Newborns protected against tetanus	Percentage of births by women of child-bearing age who are immunized against tetanus
Maternal mortality ratio	Number of women who die from pregnancy-related causes while pregnant or within 42 days of pregnancy termination per 100,000 live births
Child mortality rate	Probability of a child dying between birth and 5 years of age, per 1,000 live births
Prevalence of anemia among women of reproductive age	Prevalence of anemia among women of reproductive age (% of women ages 15-49)

Notes: DPT = diphtheria, whooping cough and tetanus; HepB3 = immunization against acute hepatitis B

For peer review only - http://bmjopen.bmj.com/site/about/guidelines.xhtml

Table 2. PEPFAR impacts on four immunization rates: traditional and staggered DID estimates

Outcomes and estimation methods	All PEPFAR countries	COP countries	Non-COP countries	High intensity PEPFAR countries	Medium intensity PEPFAR countries	Low intensity PEPFAR countries
**DPT immunization rate**						
Means	78.0	74.2	79.9	74.8	80.1	79.0
Model 1. Unadjusted model (traditional)	8.421***	8.607***	8.221***	9.728***	5.467***	9.855***
Model 1. Unadjusted model (staggered)	8.206***	6.538***	10.80***	8.373***	4.149**	14.49***
Model 2. Adjusted model (traditional)	8.810***	8.693***	8.889***	9.380***	6.635***	10.07***
Model 2. Adjusted model (staggered)	7.328***	7.459***	8.334***	8.326***	6.836***	8.979***
**Hepatitis B immunization rate**						
Means	79.1	74.4	81.7	80.7	78.3	78.5
Model 1. Unadjusted model (traditional)	1.374	8.027**	-2.097	2.661	2.163	-0.648
Model 1. Unadjusted model (staggered)	5.436**	16.33***	3.000	10.78*	3.096	6.397
Model 2. Adjusted model (traditional)	6.667**	13.64***	3.638	8.922**	5.446	7.410*
Model 2. Adjusted model (staggered)	10.52***	21.33***	7.050***	17.15***	6.967**	9.912***
**Measles immunization rate**						
Means	77.5	74.5	79.1	72.9	79.9	79.7
Model 1. Unadjusted model (traditional)	6.644***	7.192***	6.260***	7.034***	4.287**	8.439***
Model 1. Unadjusted model (staggered)	6.816***	5.091***	9.077***	5.692***	3.280*	13.28***
Model 2. Adjusted model (traditional)	6.946***	7.462***	6.560***	6.973***	5.061***	8.529***
Model 2. Adjusted model (staggered)	5.515***	5.546***	6.115***	5.158***	5.357***	7.083***
**Prevalence of newborns protected against tetanus**						
Means	75.7	75.6	75.8	75.1	78.3	74.2
Model 1. Unadjusted model (traditional)	6.069***	4.775**	6.995***	6.154***	5.845**	6.059***
Model 1. Unadjusted model (staggered)	5.218***	5.444***	5.942***	7.005***	4.966**	5.401***
Model 2. Adjusted model (traditional)	5.240***	3.736*	6.393***	5.271***	5.510**	5.229***
Model 2. Adjusted model (staggered)	3.619***	4.053**	4.103***	5.475***	5.391**	1.022

Notes: COP= country operational plans; DID=difference-in-differences; DPT= diphtheria, pertussis, tetanus; PEPFAR= President's Emergency Plan for AIDS Relief
***p < 0.001 **p < 0.01 * p< 0.05.

For peer review only

For peer review only

Table 3. PEPFAR impacts on maternal mortality ratio, child (under-five) mortality rate, and the prevalence of anemia among women of reproductive age: traditional and staggered DID estimates

Outcomes and methods	All PEPFAR countries	COP countries	Non-COP countries	High intensity PEPFAR countries	Medium intensity PEPFAR countries	Low intensity PEPFAR countries
**Maternal mortality ratio**						
Means	409.8	497.5	363.7	519.6	345.5	364.4
Model 1. Unadjusted model (traditional)	-96.46***	-120.90***	-83.62*	-125.77***	-98.65*	-64.96
Model 1. Unadjusted model (staggered)	-109.33***	-82.58*	-171.61***	-145.11***	19.84	-310.18***
Model 2. Adjusted model (traditional)	-100.74***	-130.90***	-85.40***	-135.68***	-100.04***	-68.94**
Model 2. Adjusted model (staggered)	-60.11***	-80.42***	-58.73***	-118.64***	-53.09*	-95.90***
**Child (under-five) mortality rate**						
Means	78.9	97.9	68.9	99.5	65.4	71.8
Model 1. Unadjusted model (traditional)	-26.90***	-34.29***	-23.02***	-37.76***	-21.98***	-20.97***
Model 1. Unadjusted model (staggered)	-22.41***	-30.81***	-26.47***	-36.15***	-11.73**	-37.12***
Model 2. Adjusted model (traditional)	-27.38***	-35.67***	-23.17***	-40.24***	-21.81***	-21.18***
Model 2. Adjusted model (staggered)	-20.65***	-32.80***	-18.44***	-37.75***	-19.11***	-19.56***
**Prevalence of anemia among women of reproductive age**						
Means	37.0	39.0	36.0	38.6	36.3	36.2
Model 1. Unadjusted model (traditional)	-1.26	-1.91*	-0.91	-2.39**	-0.90	-0.48
Model 1. Unadjusted model (staggered)	-1.25*	-1.13	-1.99*	-1.67*	1.29	-4.20***
Model 2. Adjusted model (traditional)	-1.02	-1.58*	-0.74	-2.18**	-0.72	-0.25
Model 2. Adjusted model (staggered)	-0.42	-1.09	0.00	-1.71*	-0.55	0.02

Notes: COP= country operational plans; DID=difference-in-differences; PEPFAR= President's Emergency Plan for AIDS Relief
***p < 0.001 **p < 0.01 * p< 0.05.

For peer review only

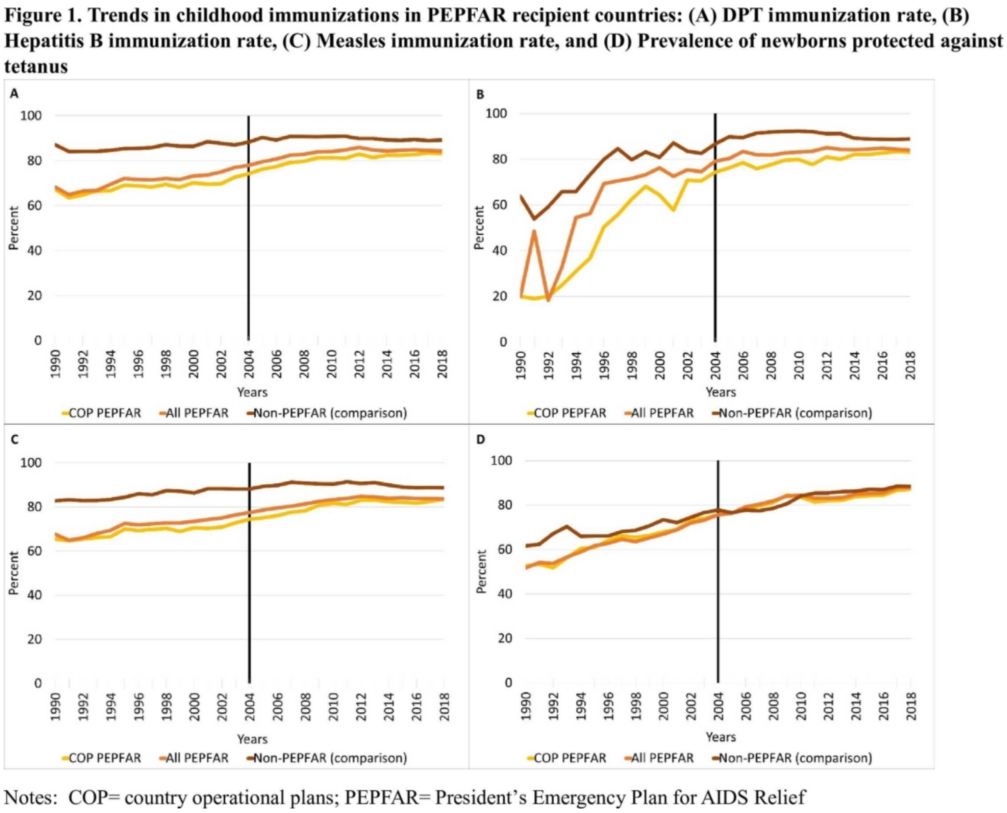

Figure 1. Trends in childhood immunizations in PEPFAR recipient countries: (A) DPT immunization rate, (B) Hepatitis B immunization rate, (C) Measles immunization rate, and (D) Prevalence of newborns protected against tetanus

190x152mm (300 x 300 DPI)

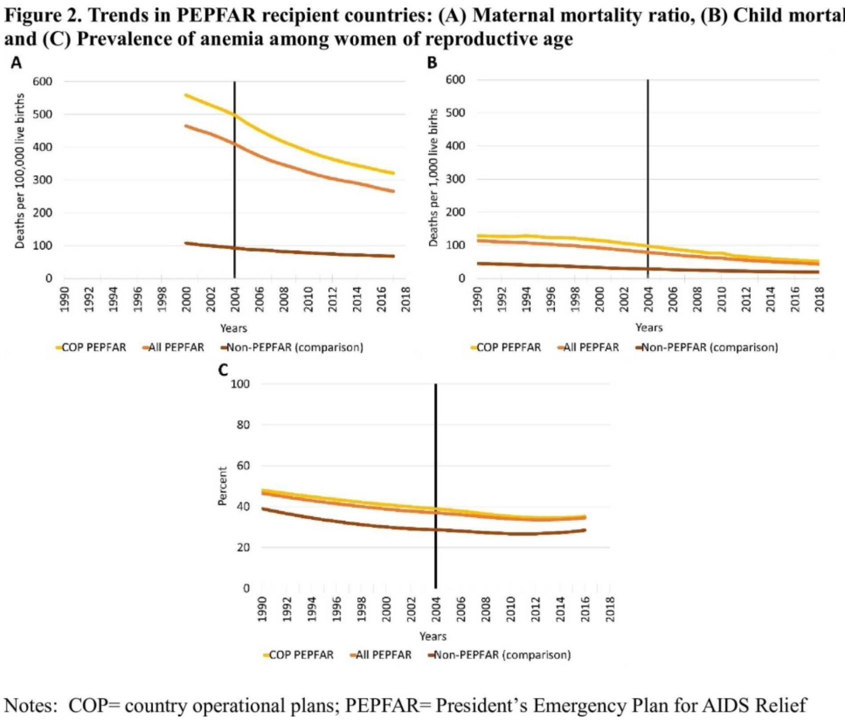

Figure 2. Trends in PEPFAR recipient countries: (A) Maternal mortality ratio, (B) Child mortality rate (under-5), and (C) Prevalence of anemia among women of reproductive age

190x134mm (300 x 300 DPI)

Analysis of Maternal and Child Health Spillover Effects in PEPFAR Countries Supplemental Materials

Gary Gaumer[1], William Crown[1], Jennifer Kates[2], Yiqun Luan[1], Dhwani Hariharan[1], Monica Jordan[1], Clare Hurley[1], AK Nandakumar[1]

[1]Institute for Global Health and Development, The Heller School for Social Policy and Management, Brandeis University, Waltham, MA, USA

[2]Kaiser Family Foundation, Global Health & HIV Policy, Washington, DC, USA

Supplement Number	Title
S1	Country list
S2	Data source and covariate inclusion rationale
S3	Full model of unlogged regression of seven outcomes from traditional DID
S4	Full model of logged regression of seven outcomes from traditional DID
S5	Full model of unlogged regression of seven outcomes from staggered DID
S6	Full model of logged regression of seven outcomes from staggered DID
S7	Parallel pre-trend assumption test for traditional DID
S8	Parallel pre-trend assumption test for staggered DID
S9	Placebo test for staggered DID
S10	Estimation results from logarithmically transformed equations 1 and 3

only

S1. Country list

All PEPFAR Funded LMICs	COPs	Non-COP PEPFAR countries	High intensity PEFAR funding per capita group	Middle intensity PEPFAR funding per capita group	Low intensity PEPFAR funding per capita group	Control group
Afghanistan	Angola	Afghanistan	Angola	Albania	Afghanistan	Algeria
Albania	Botswana	Albania	Botswana	Barbados	Armenia	American Samoa
Angola	Burundi	Armenia	Burundi	Belize	Bangladesh	Antigua and Barbuda
Armenia	Cambodia	Bangladesh	Cambodia	Benin	Bolivia	Argentina
Bangladesh	Cameroon	Barbados	Cameroon	Congo, Dem. Rep.	Brazil	Azerbaijan
Barbados	Congo, Dem. Rep.	Belize	Cote d'Ivoire	El Salvador	Burkina Faso	Belarus
Belize	Cote d'Ivoire	Benin	Djibouti	Eritrea	Central African Republic	Bhutan
Benin	Dominican Republic	Bolivia	Dominican Republic	Estonia	Chad	Bosnia and Herzegovina
Bolivia	Eswatini	Brazil	Eswatini	Gabon	China	Bulgaria
Botswana	Ethiopia	Burkina Faso	Ethiopia	Gambia, The	Comoros	Cabo Verde
Brazil	Ghana	Central African Republic	Ghana	Georgia	Congo, Rep.	Chile
Burkina Faso	Guyana	Chad	Guyana	Guatemala	Guinea-Bissau	Colombia
Burundi	Haiti	China	Haiti	Guinea	India	Costa Rica
Cambodia	India	Comoros	Honduras	Kyrgyz Republic	Indonesia	Croatia
Cameroon	Indonesia	Congo, Rep.	Jamaica	Lao PDR	Jordan	Cuba
Central African Republic	Kenya	Djibouti	Kenya	Madagascar	Kazakhstan	Czech Republic
Chad	Lesotho	El Salvador	Lesotho	Mali	Mauritania	Dominica
China	Malawi	Eritrea	Liberia	Moldova	Mexico	Ecuador
Comoros	Mozambique	Estonia	Malawi	Myanmar	Niger	Egypt, Arab Rep.
Congo, Dem. Rep.	Myanmar	Gabon	Mozambique	Nepal	North Macedonia	Equatorial Guinea
Congo, Rep.	Namibia	Gambia, The	Namibia	Nicaragua	Pakistan	Fiji
Cote d'Ivoire	Nigeria	Georgia	Nigeria	Papua New Guinea	Peru	Grenada
Djibouti	Papua New Guinea	Guatemala	Rwanda	Samoa	Philippines	Hungary
Dominican Republic	Rwanda	Guinea	Senegal	Sao Tome and Principe	Romania	Iran, Islamic Rep.
El Salvador	South Africa	Guinea-Bissau	South Africa	Sierra Leone	Russian Federation	Iraq
Eritrea	Tanzania	Honduras	Tanzania	Tajikistan	Seychelles	Kiribati
Estonia	Uganda	Jamaica	Uganda	Thailand	Suriname	Korea, Dem. People's Rep.
Eswatini	Ukraine	Jordan	Vietnam	Timor-Leste	Togo	Kosovo
Ethiopia	Vietnam	Kazakhstan	Zambia	Trinidad and Tobago	Turkmenistan	Latvia
Gabon	Zambia	Kyrgyz Republic	Zimbabwe	Ukraine	Uzbekistan	Lebanon
Gambia, The	Zimbabwe	Lao PDR				Libya
Georgia		Liberia				Lithuania
Ghana		Madagascar				Malaysia
Guatemala		Mali				Maldives
Guinea		Mauritania				Marshall Islands
Guinea-Bissau		Mexico				Mauritius
Guyana		Moldova				Mayotte
Haiti		Nepal				Micronesia, Fed. Sts.
Honduras		Nicaragua				Mongolia
India		Niger				Montenegro
Indonesia		North Macedonia				Morocco
Jamaica		Pakistan				Nauru
Jordan		Peru				Northern Mariana Islands
Kazakhstan		Philippines				Oman
Kenya		Romania				Palau
Kyrgyz Republic		Russian Federation				Panama
Lao PDR		Samoa				Paraguay
Lesotho		Sao Tome and Principe				Poland
Liberia		Senegal				Serbia
Madagascar		Seychelles				Serbia and Montenegro
Malawi		Sierra Leone				Slovak Republic
Mali		Suriname				Solomon Islands
Mauritania		Tajikistan				Somalia

Mexico		Thailand				Sri Lanka
Moldova		Timor-Leste				St. Kitts and Nevis
Mozambique		Togo				St. Lucia
Myanmar		Trinidad and Tobago				St. Vincent and the Grenadines
Namibia		Turkmenistan				Syrian Arab Republic
Nepal		Uzbekistan				Tonga
Nicaragua						Tunisia
Niger						Turkey
Nigeria						Tuvalu
North Macedonia						Uruguay
Pakistan						Vanuatu
Papua New Guinea						Venezuela, RB
Peru						West Bank and Gaza
Philippines						Yemen, Rep.
Romania						
Russian Federation						
Rwanda						
Samoa						
Sao Tome and Principe						
Senegal						
Seychelles						
Sierra Leone						
South Africa						
Suriname						
Tajikistan						
Tanzania						
Thailand						
Timor-Leste						
Togo						
Trinidad and Tobago						
Turkmenistan						
Uganda						
Ukraine						
Uzbekistan						
Vietnam						
Zambia						
Zimbabwe						

Notes: COP= country operational plans; LMICs = low- and middle-income countries; PEPFAR= President’s Emergency Plan for AIDS Relief

only

S2. Data source and covariate inclusion rationale

Covariates	Data source	Rationale for inclusion
GDP per capita (constant USD)	WDI, https://datatopics.worldbank.org/world-development-indicators/	Income levels in country will contribute to accessibility of health facilities; fraction of population that are able to afford copayments for domestic health services
Total population	United Nations, Department of Economic and Social Affairs, Population Division (2019). World Population Prospects 2019, Online Edition. Rev, https://population.un.org/wpp/	The scale of the country may influence the size/scope/and financing capabilities of country governments
Life expectancy at birth (years)	WDI, https://datatopics.worldbank.org/world-development-indicators/	A general indicator of the health level of the population
Fertility rate, total (births per woman)	WDI, https://datatopics.worldbank.org/world-development-indicators/	Fertility rate will be an indicator of the demand for MCH services and an important dimension of how fast the population growing
Percent urban population (of total population)	WDI, https://datatopics.worldbank.org/world-development-indicators/	Disease spread differs, access to HIV and other health care services is also different by residential area
School enrollment, secondary (% gross)	WDI, https://datatopics.worldbank.org/world-development-indicators/	Level of education of the population matters in risk avoidance, and care seeking
HIV prevalence (% of population ages 15-49)	WDI, https://datatopics.worldbank.org/world-development-indicators/ (from UNAIDS); The Global Burden of Disease Collaborative Network, Global Burden of Disease Study 2019 (GBD 2019) Results. Seattle, United States: Institute for Health Metrics and Evaluation (IHME), 2020, http://ghdx.healthdata.org/gbd-results-tool.	The level of intensity of the epidemic in the population
non-PEPFAR donor spending on health per capita	OECD Creditor Reporting System database, https://stats.oecd.org/Index.aspx?DataSetCode=crs1	Other donors including the Global Fund, Gates, bilateral donors provide funding for HIV and other health purposes. This covariate would capture the intensity of these potentially confounding country programs
domestic health spending per capita	WDI, https://datatopics.worldbank.org/world-development-indicators/	Private spending on Health and Domestic Government spending on Health would indicate the intensity of domestic health activity in the country
Recipient of U.S. HIV funding prior to 2004 (dummy variable)	USAID, https://foreignassistance.gov/	Many countries began to receive US Aid for HIV programming prior to PEPFAR's passage in late 2003. This is a dummy variable (1=received early HIV aid) might indicate that a country has a 'running start" on setting up PEPFAR programming

For peer review only

WB country income classification	World Bank, https://datahelpdesk.worldbank.org/knowledgebase/articles/906519-world-bank-country-and-lending-groups	Aims to control for shared unobservable characteristics among recipients of the same income classification
Diphtheria prevalence in under 5 population (DPT immunization models only)	IHME, http://ghdx.healthdata.org/gbd-results-tool	The baseline incidence levels of one of these diseases, for example, would may indicate the level of demand for the vaccine by households or caregivers
Whooping cough prevalence in under 5 population (DPT immunization models only)	IHME, http://ghdx.healthdata.org/gbd-results-tool	
Tetanus prevalence in under 5 population (DPT immunization models only)	IHME, http://ghdx.healthdata.org/gbd-results-tool	
Hepatitis B prevalence in under 5 population (HepB3 immunization models only)	IHME, http://ghdx.healthdata.org/gbd-results-tool	
Measles prevalence (measles immunization models only)	IHME, http://ghdx.healthdata.org/gbd-results-tool	

Notes: COP= country operational plans; DPT=diphtheria, pertussis, tetanus; GDP=gross domestic product; HIV=human immunodeficiency virus; IHME=Institute for Health Metrics and Evaluation; LMICs = low- and middle-income countries; MCH=maternal child health; OECD=Organization for Economic Cooperation and Development; PEPFAR= President's Emergency Plan for AIDS Relief; WB=World Bank; WDI=World Development Indicators

For peer review only

S3. Full model of unlogged regression of seven outcomes from traditional DID

For peer review only

Table S3-1. Full model results for the indicator "Percent of children ages 12-23 months who received DPT vaccinations (3 doses)"

Variables	All PEPFAR countries		COP countries		Non-COP PEPFAR countries		High-intensity PEPFAR countries		Medium-intensity PEPFAR countries		Low-intensity PEPFAR countries	
	Model 1	Model 2	Model 1	Model 2	Model 1	Model 2	Model 1	Model 2	Model 1	Model 2	Model 1	Model 2
Time variable (=1 post-2004)	3.783***<br>(0.687)	3.308***<br>(0.422)	3.783***<br>(0.687)	3.301***<br>(0.416)	3.783***<br>(0.687)	3.293***<br>(0.409)	3.783***<br>(0.687)	3.287***<br>(0.413)	3.783***<br>(0.687)	3.293***<br>(0.408)	3.783***<br>(0.687)	3.260***<br>(0.405)
Intervention (=1 if PEPFAR)	-14.91***<br>(0.823)	-5.662***<br>(0.610)	-17.80***<br>(1.146)	-11.37***<br>(1.208)	-13.28***<br>(0.964)	-4.259***<br>(0.696)	-16.88***<br>(1.173)	-6.547***<br>(1.248)	-11.54***<br>(1.154)	-3.369***<br>(0.908)	-16.08***<br>(1.318)	-5.135***<br>(0.908)
Interaction term (PEPFAR impact estimate)	8.421***<br>(1.030)	8.810***<br>(0.647)	8.607***<br>(1.430)	8.693***<br>(0.920)	8.221***<br>(1.183)	8.889***<br>(0.725)	9.728***<br>(1.446)	9.380***<br>(0.856)	5.467***<br>(1.420)	6.635***<br>(1.009)	9.855***<br>(1.598)	10.07***<br>(0.903)
Country income level (=1 if middle income)		-5.021***<br>(0.660)		-6.596***<br>(0.857)		-2.787***<br>(0.792)		-5.729***<br>(0.888)		-1.991*<br>(0.897)		-2.623**<br>(0.995)
BL Population		-1.91e-09*<br>(0.000)		-7.54e-09***<br>(0.000)		-2.31e-09**<br>(0.000)		-1.21e-08<br>(0.000)		-5.88e-08***<br>(0.000)		-3.38e-09***<br>(0.000)
BL other donor health spending per capita		0.00376<br>(0.008)		-0.0194*<br>(0.008)		-0.00417<br>(0.008)		-0.0151<br>(0.008)		-0.0169*<br>(0.008)		-0.0166<br>(0.009)
BL domestic health spending per capita		-0.00460***<br>(0.001)		-0.00274<br>(0.001)		-0.00610***<br>(0.001)		-0.00586***<br>(0.001)		-0.00759***<br>(0.001)		-0.00415***<br>(0.001)
BL GDP per capita		0.000123*<br>(0.000)		-0.00000876<br>(0.000)		0.000142**<br>(0.000)		0.0000377<br>(0.000)		0.000178***<br>(0.000)		0.0000729<br>(0.000)
BL HIV prevalence (% of population ages 15-49)		0.827***<br>(0.071)		0.685***<br>(0.088)		-0.799*<br>(0.312)		0.481***<br>(0.077)		-1.664**<br>(0.555)		-1.519***<br>(0.412)
BL diphtheria prevalence (% of population, under 5)		-3912.0***<br>(535.878)		-5160.0***<br>(547.070)		-4392.1***<br>(924.111)		-3999.0***<br>(643.387)		-1502.8<br>(1678.940)		-5415.7***<br>(961.081)
BL tetanus prevalence (% of population, under 5)		-307.1**<br>(111.856)		414.2**<br>(136.699)		-482.3***<br>(132.748)		-65.90<br>(159.354)		-46.31<br>(152.256)		-867.9***<br>(138.327)
BL pertussis (whooping cough) prevalence (% of population, under 5)		-2571.3***<br>(113.999)		-2560.4***<br>(144.504)		-2067.7***<br>(125.198)		-2787.8***<br>(136.462)		-2246.2***<br>(150.496)		-1905.3***<br>(121.393)
BL life expectancy at birth		0.628***<br>(0.067)		0.322***<br>(0.084)		0.440***<br>(0.072)		0.305***<br>(0.071)		0.428***<br>(0.083)		0.218**<br>(0.083)
BL Urban population (%)		-0.0285*<br>(0.012)		-0.0393**<br>(0.013)		-0.00421<br>(0.012)		-0.0364**<br>(0.012)		0.0374**<br>(0.013)		-0.0421***<br>(0.012)
BL School enrollment, secondary (% gross)		0.0778***<br>(0.014)		0.0745***<br>(0.017)		0.0740***<br>(0.015)		0.0447**<br>(0.016)		0.0628***<br>(0.016)		0.0349*<br>(0.016)
		-0.667*		-1.475***		-1.563***		-2.493***		-1.054**		-3.114***

For peer review only

BL Fertility rate (births per woman)		(0.285)		(0.385)		(0.291)		(0.355)		(0.368)		(0.308)
Recipient of US HIV aid before 2004 (=1 if yes)		-0.577<br>(0.488)		0.226<br>(0.741)		-1.001<br>(0.599)		-0.799<br>(0.891)		-2.251**<br>(0.818)		-1.366<br>(0.746)
Constant	86.03***<br>(0.516)	55.99***<br>(5.247)	86.03***<br>(0.516)	82.86***<br>(6.490)	86.03***<br>(0.516)	67.69***<br>(5.670)	86.03***<br>(0.516)	90.30***<br>(5.548)	86.03***<br>(0.516)	67.01***<br>(6.235)	86.03***<br>(0.516)	92.32***<br>(6.207)
Adjusted R-squared	0.144	0.656	0.181	0.669	0.121	0.661	0.163	0.696	0.096	0.564	0.136	0.717
Observations	4282	3926	2628	2328	3386	3087	2592	2263	2565	2322	2589	2319

Notes: Standard errors in parentheses *** p<0.001, ** p<0.01, * p<0.05 BL=baseline; COP=country operational plans; PEPFAR= President's Emergency Plan for AIDS Relief

For peer review only

Table S3-2. Full model results for the indicator "Percentage of children age 12-23 months who received hepatitis B vaccines, 3 doses) "

Variables	All PEPFAR countries		COP countries		Non-COP PEPFAR countries		High-intensity PEPFAR countries		Medium-intensity PEPFAR countries		Low-intensity PEPFAR countries	
	Model 1	Model 2	Model 1	Model 2	Model 1	Model 2	Model 1	Model 2	Model 1	Model 2	Model 1	Model 2
Time variable (=1 post-2004)	11.54*** (1.288)	11.60*** (1.316)	11.54*** (1.289)	11.55*** (1.321)	11.54*** (1.289)	11.60*** (1.299)	11.54*** (1.289)	11.37*** (1.305)	11.54*** (1.289)	11.55*** (1.286)	11.54*** (1.289)	11.29*** (1.279)
Intervention (=1 if PEPFAR)	-8.247*** (2.114)	-5.161* (2.171)	-18.39*** (2.933)	-20.57*** (3.351)	-2.948 (2.449)	-1.352 (2.452)	-9.384** (3.124)	-4.230 (3.406)	-9.150** (3.135)	-6.953* (3.278)	-6.256 (3.243)	-2.329 (3.163)
Interaction term (PEPFAR impact estimate)	1.374 (2.203)	6.667** (2.145)	8.027** (3.079)	13.64*** (2.967)	-2.097 (2.542)	3.638 (2.490)	2.661 (3.228)	8.922** (3.105)	2.163 (3.253)	5.446 (3.210)	-0.648 (3.396)	7.410* (3.176)
Country income level (=1 if middle income)		-4.026*** (1.189)		-6.567*** (1.752)		-4.236** (1.400)		-5.682*** (1.677)		-5.353** (1.692)		-4.018* (2.040)
BL Population		-1.21e-08** (0.000)		-3.39e-08*** (0.000)		-2.53e-09 (0.000)		-0.000000104*** (0.000)		-8.85e-08*** (0.000)		-1.60e-08*** (0.000)
BL other donor health spending per capita		0.0161 (0.013)		0.0100 (0.014)		0.0162 (0.013)		0.00816 (0.014)		0.00729 (0.014)		0.0413** (0.014)
BL domestic health spending per capita		-0.00691* (0.003)		-0.00178 (0.003)		-0.00983*** (0.003)		-0.00499 (0.003)		-0.00964*** (0.003)		-0.00944** (0.003)
BL GDP per capita		0.000364*** (0.000)		0.000312* (0.000)		0.000397*** (0.000)		0.000329** (0.000)		0.000379*** (0.000)		0.000490*** (0.000)
BL HIV prevalence (% of population ages 15-49)		0.726*** (0.116)		0.795*** (0.147)		-0.0293 (0.666)		0.193 (0.128)		-0.223 (1.234)		-0.994 (0.916)
BL hepatitis B prevalence (% of population, under 5)		-73.76*** (6.893)		-88.09*** (11.420)		-59.27*** (9.726)		-85.64*** (9.377)		-79.29*** (11.973)		-1.979 (14.980)
BL life expectancy at birth		0.544*** (0.111)		0.385* (0.164)		0.297* (0.120)		0.244 (0.145)		0.452** (0.160)		0.626*** (0.152)
BL Urban population (%)		-0.0208 (0.021)		-0.111*** (0.024)		0.00257 (0.023)		-0.0877*** (0.024)		0.0198 (0.027)		-0.0551* (0.025)
BL School enrollment, secondary (% gross)		0.0694** (0.025)		0.0793* (0.035)		0.0801** (0.026)		0.0632 (0.033)		0.111*** (0.031)		0.130*** (0.030)
BL Fertility rate (births per woman)		-0.266 (0.482)		-0.843 (0.641)		-2.202*** (0.505)		-2.715*** (0.641)		-0.834 (0.623)		-4.052*** (0.629)
Recipient of US HIV aid before 2004 (=1 if yes)		-1.066 (0.846)		6.564*** (1.451)		-1.702 (1.054)		2.547 (1.457)		1.188 (1.524)		-3.207 (1.733)
Constant	78.67***	41.62***	78.67***	59.37***	78.67***	62.93***	78.67***	76.13***	78.67***	47.78***	78.67***	40.84***

For peer review only

	(1.219)	(8.855)	(1.220)	(12.966)	(1.219)	(9.572)	(1.220)	(11.518)	(1.220)	(12.488)	(1.220)	(11.480)
Adjusted R-squared	0.093	0.288	0.158	0.351	0.071	0.278	0.104	0.343	0.104	0.264	0.085	0.325
Observations	2799	2579	1789	1598	2270	2065	1744	1541	1809	1633	1766	1573

Notes: Standard errors in parentheses *** p<0.001, ** p<0.01, * p<0.05 BL=baseline; COP=country operational plans; PEPFAR= President's Emergency Plan for AIDS Relief

For peer review only

Table S3-3. Full model results for the indicator "Percent of children ages 12-23 months who received the measles vaccination"

Variables	All PEPFAR countries		COP countries		Non-COP PEPFAR countries		High-intensity PEPFAR countries		Medium-intensity PEPFAR countries		Low-intensity PEPFAR countries	
	Model 1	Model 2	Model 1	Model 2	Model 1	Model 2	Model 1	Model 2	Model 1	Model 2	Model 1	Model 2
Time variable (=1 post-2004)	4.348*** (0.679)	3.968*** (0.461)	4.348*** (0.679)	3.964*** (0.468)	4.348*** (0.679)	3.954*** (0.450)	4.348*** (0.679)	3.958*** (0.474)	4.348*** (0.679)	3.962*** (0.453)	4.348*** (0.679)	3.928*** (0.450)
Intervention (=1 if PEPFAR)	-14.16*** (0.794)	-6.963*** (0.672)	-16.87*** (1.061)	-11.20*** (1.267)	-12.64*** (0.928)	-5.398*** (0.740)	-15.92*** (1.057)	-10.19*** (1.301)	-11.49*** (1.120)	-5.582*** (1.022)	-14.91*** (1.265)	-5.543*** (0.874)
Interaction term (PEPFAR impact estimate)	6.644*** (0.995)	6.946*** (0.694)	7.192*** (1.321)	7.462*** (0.986)	6.260*** (1.153)	6.560*** (0.768)	7.034*** (1.330)	6.973*** (0.954)	4.287** (1.399)	5.061*** (1.053)	8.439*** (1.540)	8.529*** (0.905)
Country income level (=1 if middle income)		-5.948*** (0.657)		-7.716*** (0.862)		-4.429*** (0.827)		-7.699*** (0.916)		-6.306*** (1.022)		-4.098*** (0.982)
BL Population		-4.14e-09*** (0.000)		-9.31e-09*** (0.000)		-1.69e-09 (0.000)		-7.29e-08*** (0.000)		-1.66e-08 (0.000)		-6.02e-09*** (0.000)
BL other donor health spending per capita		0.0585*** (0.009)		0.0499*** (0.010)		0.0402*** (0.009)		0.0358*** (0.010)		0.0364*** (0.009)		0.0469*** (0.009)
BL domestic health spending per capita		-0.00638*** (0.001)		-0.00652*** (0.001)		-0.00475*** (0.001)		-0.00494*** (0.001)		-0.00383** (0.001)		-0.00556*** (0.001)
BL GDP per capita		0.000253*** (0.000)		0.000292*** (0.000)		0.000185*** (0.000)		0.000224*** (0.000)		0.000163** (0.000)		0.000280*** (0.000)
BL HIV prevalence (% of population ages 15-49)		1.208*** (0.067)		1.162*** (0.076)		-0.231 (0.310)		1.047*** (0.077)		0.137 (0.554)		0.0681 (0.385)
BL measles prevalence (% of population, under 5)		-17.05*** (1.365)		-21.21*** (1.669)		-5.349*** (1.579)		-20.82*** (1.694)		-0.382 (1.815)		-10.86*** (1.805)
BL life expectancy at birth		0.826*** (0.063)		0.714*** (0.075)		0.624*** (0.072)		0.694*** (0.074)		0.706*** (0.087)		0.650*** (0.081)
BL Urban population (%)		-0.0128 (0.013)		-0.0555*** (0.013)		0.0322* (0.013)		-0.0425*** (0.013)		0.0319* (0.013)		-0.0279* (0.013)
BL School enrollment, secondary (% gross)		0.0834*** (0.014)		0.0567*** (0.017)		0.128*** (0.014)		0.0774*** (0.016)		0.120*** (0.017)		0.126*** (0.015)
BL Fertility rate (births per woman)		-2.887*** (0.281)		-3.192*** (0.348)		-3.468*** (0.299)		-2.524*** (0.351)		-3.561*** (0.385)		-3.976*** (0.289)
Recipient of US HIV aid before 2004 (=1 if yes)		2.089*** (0.528)		3.191*** (0.839)		2.937*** (0.598)		4.077*** (0.907)		2.064* (0.902)		2.265** (0.770)
Constant	85.59*** (0.529)	36.74*** (5.004)	85.59*** (0.529)	51.92*** (5.769)	85.59*** (0.529)	44.10*** (5.710)	85.59*** (0.529)	50.15*** (5.719)	85.59*** (0.529)	40.33*** (6.769)	85.59*** (0.529)	46.69*** (6.147)
Adjusted R-squared	0.147	0.578	0.192	0.572	0.123	0.602	0.175	0.571	0.106	0.504	0.135	0.675

For peer review only

Observations	4275	3926	2621	2328	3379	3087	2585	2263	2558	2322	2582	2319

Notes: Standard errors in parentheses *** p<0.001, ** p<0.01, * p<0.05 BL=baseline; COP=country operational plans; PEPFAR= President's Emergency Plan for AIDS Relief

For peer review only

Table S3-4. Full model results for the indicator "Percentage of births by women of child-bearing age who are immunized against tetanus"

Variables	All PEPFAR countries		COP countries		Non-COP PEPFAR countries		High-intensity PEPFAR countries		Medium-intensity PEPFAR countries		Low-intensity PEPFAR countries	
	Model 1	Model 2	Model 1	Model 2	Model 1	Model 2	Model 1	Model 2	Model 1	Model 2	Model 1	Model 2
Time variable (=1 post-2004)	14.23***	14.93***	14.23***	15.09***	14.23***	14.83***	14.23***	14.92***	14.23***	14.51***	14.23***	15.06***
	(1.201)	(1.144)	(1.202)	(1.128)	(1.201)	(1.120)	(1.202)	(1.139)	(1.202)	(1.134)	(1.202)	(1.098)
Intervention (=1 if PEPFAR)	-6.327***	0.630	-5.814***	-3.080	-6.706***	1.822	-6.555***	-3.278	-4.992**	0.458	-7.175***	3.863**
	(1.211)	(1.316)	(1.405)	(1.840)	(1.341)	(1.435)	(1.399)	(2.029)	(1.621)	(1.777)	(1.576)	(1.448)
Interaction term (PEPFAR impact estimate)	6.069***	5.240***	4.775**	3.736*	6.995***	6.393***	6.154***	5.271***	5.845**	5.510**	6.059***	5.229***
	(1.399)	(1.330)	(1.603)	(1.526)	(1.543)	(1.431)	(1.600)	(1.548)	(1.838)	(1.763)	(1.807)	(1.490)
Country income level (=1 if middle income)		-0.0482		2.648*		0.0849		1.399		-0.717		-0.317
		(0.981)		(1.229)		(1.450)		(1.367)		(1.938)		(1.683)
BL Population		7.33e-09***		4.18e-09*		3.24e-09		-9.99e-08***		-3.03e-08		6.74e-10
		(0.000)		(0.000)		(0.000)		(0.000)		(0.000)		(0.000)
BL other donor health spending per capita		0.481***		0.184**		0.478***		0.138*		0.408***		0.332***
		(0.046)		(0.056)		(0.051)		(0.061)		(0.064)		(0.049)
BL domestic health spending per capita		0.00673*		-0.00432		0.00927**		0.00477		0.0163***		0.0108***
		(0.003)		(0.005)		(0.003)		(0.005)		(0.005)		(0.003)
BL GDP per capita		0.000399***		0.000653***		0.000427***		0.000430***		0.0000233		0.000584***
		(0.000)		(0.000)		(0.000)		(0.000)		(0.000)		(0.000)
BL HIV prevalence (% of population ages 15-49)		0.0139		0.351**		-1.602**		0.116		0.0926		-1.498*
		(0.104)		(0.131)		(0.515)		(0.135)		(0.855)		(0.604)
BL life expectancy at birth		0.0449		0.325*		-0.481***		0.236		-0.543***		-0.434*
		(0.099)		(0.146)		(0.137)		(0.138)		(0.163)		(0.183)
BL Urban population (%)		-0.162***		-0.267***		-0.163***		-0.258***		-0.116***		-0.315***
		(0.023)		(0.030)		(0.028)		(0.028)		(0.032)		(0.031)
BL School enrollment, secondary (% gross)		0.00306		-0.0430		0.0214		-0.0244		0.198***		-0.0307
		(0.024)		(0.035)		(0.029)		(0.039)		(0.038)		(0.033)
BL Fertility rate (births per woman)		-3.190***		-2.920***		-5.454***		-2.315**		-3.763***		-8.121***
		(0.475)		(0.649)		(0.619)		(0.824)		(0.678)		(0.619)
Recipient of US HIV aid before 2004 (=1 if yes)		0.828		6.109***		-0.649		6.284***		1.447		-1.469
		(0.663)		(1.090)		(0.832)		(1.092)		(1.288)		(1.127)
Constant	68.91***	73.74***	68.91***	61.94***	68.91***	115.9***	68.91***	67.17***	68.91***	103.5***	68.91***	131.1***
	(1.027)	(7.814)	(1.027)	(10.981)	(1.027)	(10.941)	(1.027)	(11.383)	(1.027)	(12.419)	(1.027)	(13.269)
Adjusted R-squared	0.264	0.381	0.233	0.361	0.257	0.409	0.252	0.378	0.220	0.331	0.226	0.504
Observations	2830	2657	1624	1480	1964	1848	1614	1441	1336	1249	1396	1309

Notes: Standard errors in parentheses *** p<0.001, ** p<0.01, * p<0.05 BL=baseline; COP=country operational plans; PEPFAR= President's Emergency Plan for AIDS Relief

For peer review only

Table S3-5. Full model results for the indicator "Number of women who die from pregnancy-related causes while pregnant or within 42 days of pregnancy termination per 100,000 live births"

Variables	All PEPFAR countries		COP countries		Non-COP PEPFAR countries		High-intensity PEPFAR countries		Medium-intensity PEPFAR countries		Low-intensity PEPFAR countries	
	Model 1	Model 2	Model 1	Model 2	Model 1	Model 2	Model 1	Model 2	Model 1	Model 2	Model 1	Model 2
Time variable (=1 post-2004)	-23.62*	-22.22***	-23.62*	-22.22***	-23.62*	-22.22**	-23.62*	-22.22***	-23.62*	-22.22**	-23.62*	-22.22***
	(12.025)	(6.590)	(12.031)	(4.599)	(12.027)	(6.926)	(12.031)	(4.952)	(12.031)	(6.818)	(12.031)	(5.261)
Intervention (=1 if PEPFAR)	344.3***	119.3***	434.5***	173.1***	296.8***	96.95***	457.8***	312.0***	287.8***	96.56***	287.2***	127.4***
	(24.509)	(13.902)	(28.938)	(22.518)	(31.804)	(16.785)	(29.865)	(21.591)	(44.605)	(25.704)	(40.462)	(20.937)
Interaction term (PEPFAR impact estimate)	-96.46***	-100.7***	-120.9***	-130.9***	-83.62*	-85.40***	-125.8***	-135.7***	-98.65*	-100.0***	-64.96	-68.94**
	(26.427)	(14.549)	(31.291)	(18.636)	(34.231)	(18.448)	(32.313)	(17.343)	(47.410)	(27.738)	(44.089)	(22.129)
Country income level (=1 if middle income)		-9.210		-54.34***		8.032		-52.43***		11.30		-15.88
		(12.949)		(12.326)		(14.711)		(12.773)		(17.366)		(14.605)
BL Population		1.61e-08		-5.30e-09		2.39e-08*		0.00000154***		-6.83e-08		-2.78e-08**
		(0.000)		(0.000)		(0.000)		(0.000)		(0.000)		(0.000)
BL other donor health spending per capita		-1.030***		-0.590***		-0.976***		-0.329***		-0.850***		-0.646***
		(0.131)		(0.086)		(0.132)		(0.061)		(0.124)		(0.105)
BL domestic health spending per capita		0.235***		0.126***		0.287***		0.0875***		0.269***		0.195***
		(0.019)		(0.017)		(0.022)		(0.015)		(0.027)		(0.021)
BL GDP per capita		-0.00652***		-0.00247***		-0.00829***		-0.00151***		-0.00682***		-0.00604***
		(0.001)		(0.000)		(0.001)		(0.000)		(0.001)		(0.001)
BL HIV prevalence (% of population ages 15-49)		-18.21***		-8.354***		3.454		-10.38***		-9.382		25.44***
		(1.719)		(1.404)		(6.607)		(1.112)		(8.656)		(7.458)
BL life expectancy at birth		-27.23***		-16.37***		-26.98***		-15.91***		-28.87***		-14.35***
		(1.694)		(1.316)		(2.223)		(1.039)		(2.979)		(1.447)
BL Urban population (%)		-0.0420		-0.195		-0.403*		-0.468**		-0.157		-0.454**
		(0.183)		(0.155)		(0.191)		(0.153)		(0.208)		(0.162)
BL School enrollment, secondary (% gross)		-1.340***		-0.846***		-1.851***		-1.176***		-1.333***		-2.084***
		(0.203)		(0.192)		(0.203)		(0.180)		(0.204)		(0.218)
BL Fertility rate (births per woman)		24.85***		26.97***		25.77***		12.82**		22.32***		37.64***
		(4.525)		(3.681)		(4.823)		(4.175)		(4.459)		(4.616)
Recipient of US HIV aid before 2004 (=1 if yes)		-66.19***		-27.82*		-49.84***		-155.8***		-35.08*		-46.21***
		(8.720)		(11.967)		(9.233)		(11.494)		(16.111)		(9.503)
Constant	101.8***	2057.0***	101.8***	1287.4***	101.8***	2069.6***	101.8***	1322.0***	101.8***	2149.3***	101.8***	1195.5***
	(11.036)	(120.547)	(11.042)	(88.946)	(11.038)	(156.571)	(11.042)	(75.173)	(11.042)	(207.386)	(11.042)	(98.220)
Adjusted R-squared	0.196	0.772	0.449	0.815	0.154	0.780	0.469	0.861	0.161	0.726	0.181	0.789
Observations	2628	2412	1566	1386	2070	1890	1548	1350	1548	1404	1548	1386

Notes: Standard errors in parentheses *** p<0.001, ** p<0.01, * p<0.05 BL=baseline; COP=country operational plans; PEPFAR= President's Emergency Plan for AIDS Relief

For peer review only

Table S3-6. Full model results for the indicator "Probability of a child dying between birth and 5 years of age, per 1,000 live births"

Variables	All PEPFAR countries		COP countries		Non-COP PEPFAR countries		High-intensity PEPFAR countries		Medium-intensity PEPFAR countries		Low-intensity PEPFAR countries	
	Model 1	Model 2	Model 1	Model 2	Model 1	Model 2	Model 1	Model 2	Model 1	Model 2	Model 1	Model 2
Time variable (=1 post-2004)	-14.45***	-14.62***	-14.45***	-14.62***	-14.45***	-14.62***	-14.45***	-14.62***	-14.45***	-14.62***	-14.45***	-14.62***
	(1.374)	(0.795)	(1.374)	(0.767)	(1.374)	(0.794)	(1.374)	(0.772)	(1.374)	(0.767)	(1.374)	(0.775)
Intervention (=1 if PEPFAR)	62.90***	14.73***	82.70***	17.64***	52.49***	11.21***	86.70***	33.69***	49.38***	7.373***	52.62***	12.81***
	(2.123)	(1.291)	(2.868)	(2.437)	(2.549)	(1.364)	(3.043)	(3.286)	(3.253)	(1.576)	(3.323)	(1.893)
Interaction term (PEPFAR impact estimate)	-26.90***	-27.38***	-34.29***	-35.67***	-23.02***	-23.17***	-37.76***	-40.24***	-21.98***	-21.81***	-20.97***	-21.18***
	(2.517)	(1.285)	(3.362)	(1.984)	(3.021)	(1.418)	(3.522)	(2.209)	(3.856)	(1.661)	(3.981)	(1.797)
Country income level (=1 if middle income)		-1.820		2.627		-4.866**		3.619		-5.253**		-5.711*
		(1.324)		(2.046)		(1.526)		(2.094)		(1.638)		(2.229)
BL Population		9.74e-09***		1.27e-08***		1.05e-08***		6.95e-08***		6.61e-08***		6.26e-09***
		(0.000)		(0.000)		(0.000)		(0.000)		(0.000)		(0.000)
BL other donor health spending per capita		-0.266***		-0.210***		-0.261***		-0.203***		-0.239***		-0.207***
		(0.019)		(0.018)		(0.020)		(0.019)		(0.020)		(0.020)
BL domestic health spending per capita		0.0148***		0.00285		0.0203***		-0.00188		0.0190***		0.0119***
		(0.002)		(0.003)		(0.002)		(0.003)		(0.003)		(0.003)
BL GDP per capita		- 0.000482***		0.0000306		- 0.000612***		0.000102		- 0.000481***		- 0.000364***
		(0.000)		(0.000)		(0.000)		(0.000)		(0.000)		(0.000)
BL HIV prevalence (% of population ages 15-49)		-2.428***		-1.969***		-0.694		-2.338***		1.199		1.000
		(0.148)		(0.166)		(0.612)		(0.183)		(0.861)		(0.808)
BL life expectancy at birth		-3.943***		-3.659***		-3.952***		-3.748***		-4.121***		-3.139***
		(0.118)		(0.153)		(0.146)		(0.146)		(0.177)		(0.164)
BL Urban population (%)		-0.00453		0.0526*		-0.0839***		0.0754**		-0.0708**		-0.0734***
		(0.023)		(0.023)		(0.022)		(0.026)		(0.022)		(0.022)
BL School enrollment, secondary (% gross)		-0.206***		-0.274***		-0.194***		-0.259***		-0.125***		-0.239***
		(0.024)		(0.031)		(0.025)		(0.033)		(0.027)		(0.027)
BL Fertility rate (births per woman)		7.443***		6.159***		7.046***		5.158***		6.509***		7.496***
		(0.525)		(0.630)		(0.569)		(0.704)		(0.545)		(0.683)
Recipient of US HIV aid before 2004 (=1 if yes)		-1.934		3.893*		-0.604		-6.205**		1.181		0.592
		(1.010)		(1.634)		(1.060)		(2.401)		(1.355)		(1.491)
Constant	37.56***	317.9***	37.56***	298.1***	37.56***	323.9***	37.56***	304.9***	37.56***	329.0***	37.56***	269.2***
	(1.129)	(8.954)	(1.129)	(12.020)	(1.129)	(10.724)	(1.129)	(11.757)	(1.129)	(12.990)	(1.129)	(11.589)
Adjusted R-squared	0.298	0.838	0.486	0.842	0.243	0.843	0.494	0.837	0.240	0.840	0.254	0.839
Observations	4408	4002	2697	2349	3509	3161	2668	2291	2668	2378	2668	2349

Notes: Standard errors in parentheses *** p<0.001, ** p<0.01, * p<0.05 BL=baseline; COP=country operational plans; PEPFAR= President's Emergency Plan for AIDS Relief

For peer review only

Table S3-7. Full model results for the indicator "Prevalence of anemia among women of reproductive age (% of women ages 15-49)"

Variables	All PEPFAR countries		COP countries		Non-COP PEPFAR countries		High-intensity PEPFAR countries		Medium-intensity PEPFAR countries		Low-intensity PEPFAR countries	
	Model 1	Model 2	Model 1	Model 2	Model 1	Model 2	Model 1	Model 2	Model 1	Model 2	Model 1	Model 2
Time variable (=1 post-2004)	-5.415*** (0.478)	-5.587*** (0.413)	-5.415*** (0.479)	-5.587*** (0.390)	-5.415*** (0.478)	-5.587*** (0.395)	-5.415*** (0.479)	-5.587*** (0.395)	-5.415*** (0.479)	-5.587*** (0.380)	-5.415*** (0.479)	-5.587*** (0.390)
Intervention (=1 if PEPFAR)	8.469*** (0.501)	-0.967* (0.480)	10.39*** (0.588)	-2.582** (0.889)	7.461*** (0.591)	-2.707*** (0.488)	10.16*** (0.610)	-1.116 (1.017)	7.960*** (0.773)	-2.932*** (0.578)	7.287*** (0.710)	-2.899*** (0.692)
Interaction term (PEPFAR impact estimate)	-1.256 (0.688)	-1.023 (0.536)	-1.908* (0.839)	-1.581* (0.739)	-0.914 (0.813)	-0.740 (0.551)	-2.389** (0.875)	-2.184** (0.800)	-0.897 (1.063)	-0.724 (0.657)	-0.482 (0.989)	-0.252 (0.632)
Country income level (=1 if middle income)		-5.738*** (0.562)		-0.478 (0.757)		-7.546*** (0.705)		-1.090 (0.819)		-4.577*** (0.766)		-9.547*** (1.025)
BL Population		3.62e-09** (0.000)		1.21e-08*** (0.000)		-2.66e-10 (0.000)		-1.16e-08 (0.000)		3.43e-08*** (0.000)		2.27e-09* (0.000)
BL other donor health spending per capita		-0.0717*** (0.003)		-0.0633*** (0.004)		-0.0685*** (0.004)		-0.0628*** (0.004)		-0.0797*** (0.004)		-0.0605*** (0.005)
BL domestic health spending per capita		-0.00834*** (0.001)		-0.00623*** (0.001)		-0.00420*** (0.001)		-0.00714*** (0.001)		0.000529 (0.001)		-0.00470*** (0.001)
BL GDP per capita		0.000395*** (0.000)		0.000380*** (0.000)		0.000315*** (0.000)		0.000416*** (0.000)		0.000224*** (0.000)		0.000315*** (0.000)
BL HIV prevalence (% of population ages 15-49)		-0.674*** (0.043)		-0.571*** (0.052)		0.782*** (0.197)		-0.690*** (0.063)		0.766 (0.393)		0.628** (0.220)
BL life expectancy at birth		-0.584*** (0.036)		-0.474*** (0.049)		-0.452*** (0.041)		-0.541*** (0.053)		-0.597*** (0.048)		-0.247*** (0.054)
BL Urban population (%)		0.0352** (0.012)		-0.0543*** (0.013)		-0.0240* (0.011)		-0.0362** (0.014)		-0.0739*** (0.012)		-0.0698*** (0.013)
BL School enrollment, secondary (% gross)		-0.0529*** (0.011)		-0.128*** (0.014)		-0.0689*** (0.011)		-0.130*** (0.015)		-0.114*** (0.012)		-0.0621*** (0.013)
BL Fertility rate (births per woman)		1.243*** (0.195)		0.329 (0.308)		1.543*** (0.210)		-0.0342 (0.367)		1.816*** (0.229)		1.459*** (0.257)
Recipient of US HIV aid before 2004 (=1 if yes)		-0.777* (0.335)		2.547*** (0.534)		0.361 (0.387)		2.561*** (0.638)		-1.045 (0.534)		1.752** (0.584)
Constant	32.99*** (0.359)	79.22*** (3.029)	32.99*** (0.359)	78.72*** (4.425)	32.99*** (0.359)	73.80*** (3.318)	32.99*** (0.359)	84.49*** (4.858)	32.99*** (0.359)	85.47*** (3.923)	32.99*** (0.359)	63.42*** (4.450)
Adjusted R-squared	0.162	0.590	0.241	0.526	0.137	0.659	0.226	0.505	0.146	0.654	0.144	0.636
Observations	3996	3672	2403	2133	3159	2889	2376	2079	2376	2160	2376	2133

Notes: Standard errors in parentheses ** p<0.001, ** p<0.01, * p<0.05 BL=baseline; COP=country operational plans; PEPFAR= President's Emergency Plan for AIDS Relief

For peer review only

S4. Full model of logged regression of seven outcomes from traditional DID

For peer review only

Table S4-1. Full model results for the indicator "Percent of children ages 12-23 months who received DPT vaccinations (3 doses)"

Variables	All PEPFAR countries		COP countries		Non-COP PEPFAR countries		High-intensity PEPFAR countries		Medium-intensity PEPFAR countries		Low-intensity PEPFAR countries	
	Model 1	Model 2	Model 1	Model 2	Model 1	Model 2	Model 1	Model 2	Model 1	Model 2	Model 1	Model 2
Time variable (=1 post-2004)	0.0457***	0.0388***	0.0457***	0.0382***	0.0457***	0.0387***	0.0457***	0.0382***	0.0457***	0.0385***	0.0457***	0.0383***
	(0.011)	(0.010)	(0.011)	(0.010)	(0.011)	(0.008)	(0.011)	(0.009)	(0.011)	(0.008)	(0.011)	(0.008)
Intervention (=1 if PEPFAR)	-0.237***	-0.120***	-0.277***	-0.119***	-0.214***	-0.106***	-0.260***	-0.0709**	-0.173***	-0.0529***	-0.273***	-0.153***
	(0.014)	(0.012)	(0.021)	(0.024)	(0.017)	(0.013)	(0.021)	(0.025)	(0.020)	(0.015)	(0.025)	(0.018)
Interaction term (PEPFAR impact estimate)	0.157***	0.171***	0.160***	0.186***	0.154***	0.165***	0.172***	0.159***	0.102***	0.143***	0.193***	0.214***
	(0.018)	(0.014)	(0.026)	(0.020)	(0.020)	(0.015)	(0.025)	(0.020)	(0.023)	(0.018)	(0.028)	(0.020)
Country income level (=1 if middle income)		-0.0674***		-0.0864***		-0.0727***		-0.0877***		-0.0884***		-0.100***
		(0.015)		(0.020)		(0.014)		(0.022)		(0.016)		(0.018)
Ln(BL Population)		-0.00746***		-0.00232		-0.0165***		0.000465		-0.00577*		-0.0157***
		(0.002)		(0.003)		(0.002)		(0.003)		(0.002)		(0.002)
Ln(BL other donor health spending per capita)		0.0172***		0.00806		0.00300		0.00551		0.00967**		0.00221
		(0.004)		(0.004)		(0.003)		(0.004)		(0.004)		(0.003)
Ln(BL domestic health spending per capita)		-0.0194		0.00207		-0.0537***		-0.0283*		-0.000728		-0.0624***
		(0.012)		(0.014)		(0.013)		(0.013)		(0.013)		(0.015)
Ln(BL GDP per capita)		-0.0143		-0.0582**		-0.0243		-0.0500*		-0.0306		-0.0226
		(0.016)		(0.022)		(0.017)		(0.020)		(0.017)		(0.021)
Ln(BL HIV prevalence (% of population ages 15-49))		-0.000723		-0.00307		-0.00518***		-0.00490		-0.00458**		-0.00301
		(0.001)		(0.002)		(0.001)		(0.003)		(0.001)		(0.002)
Ln(BL diphtheria prevalence (% of population, under 5))		-0.0302***		-0.0331***		-0.0104***		-0.0298***		-0.00679**		-0.00376
		(0.002)		(0.003)		(0.002)		(0.003)		(0.002)		(0.002)
Ln(BL tetanus prevalence (% of population, under 5))		0.0153***		0.0110***		0.0132***		0.00464		0.0138***		0.0105***
		(0.002)		(0.002)		(0.002)		(0.002)		(0.003)		(0.002)
Ln(BL pertussis (whooping cough) prevalence (% of population, under 5))		-0.143***		-0.132***		-0.0992***		-0.137***		-0.106***		-0.0779***
		(0.007)		(0.010)		(0.006)		(0.010)		(0.007)		(0.006)
Ln(BL life expectancy at birth)		-0.00507		-0.200***		0.770***		-0.110*		0.764***		0.958***
		(0.045)		(0.050)		(0.088)		(0.050)		(0.097)		(0.110)
Ln(BL Urban population (%))		0.00682		-0.00816		0.130***		0.00458		0.0841***		0.109***
		(0.012)		(0.015)		(0.015)		(0.015)		(0.012)		(0.017)
Ln(BL School enrollment, secondary (%		0.128***		0.0650**		0.140***		0.0787***		0.114***		0.159***
		(0.017)		(0.023)		(0.019)		(0.022)		(0.023)		(0.021)

For peer review only

gross))												
Ln(BL Fertility rate (births per woman))		-0.173*** (0.016)		-0.255*** (0.022)		-0.112*** (0.015)		-0.229*** (0.022)		-0.0900*** (0.017)		-0.151*** (0.017)
Recipient of US HIV aid before 2004 (=1 if yes)		0.0298** (0.011)		-0.0474** (0.014)		0.0464*** (0.010)		-0.0452* (0.018)		-0.0208 (0.013)		0.0339** (0.012)
Constant	4.433*** (0.008)	3.430*** (0.201)	4.433*** (0.008)	4.865*** (0.232)	4.433*** (0.008)	0.429 (0.336)	4.433*** (0.008)	4.357*** (0.247)	4.433*** (0.008)	0.344 (0.421)	4.433*** (0.008)	-0.102 (0.416)
Adjusted R-squared	0.120	0.511	0.142	0.502	0.106	0.569	0.130	0.492	0.074	0.492	0.123	0.618
Observations	4282	3540	2628	2024	3386	2728	2592	1986	2565	1992	2589	1986

Notes: Standard errors in parentheses *** p<0.001, ** p<0.01, * p<0.05 BL=baseline; COP=country operational plans; PEPFAR= President's Emergency Plan for AIDS Relief

For peer review only

Table S4-2. Full model results for the indicator "Percentage of children age 12-23 months who received hepatitis B vaccines, 3 doses) "

Variables	All PEPFAR countries		COP countries		Non-COP PEPFAR countries		High-intensity PEPFAR countries		Medium-intensity PEPFAR countries		Low-intensity PEPFAR countries	
	Model 1	Model 2	Model 1	Model 2	Model 1	Model 2	Model 1	Model 2	Model 1	Model 2	Model 1	Model 2
Time variable (=1 post-2004)	0.240*** (0.034)	0.212*** (0.032)	0.240*** (0.034)	0.201*** (0.032)	0.240*** (0.034)	0.212*** (0.032)	0.240*** (0.034)	0.200*** (0.032)	0.240*** (0.034)	0.212*** (0.032)	0.240*** (0.034)	0.199*** (0.031)
Intervention (=1 if PEPFAR)	-0.184** (0.060)	-0.190** (0.060)	-0.343*** (0.090)	-0.348*** (0.087)	-0.101 (0.070)	-0.158* (0.072)	-0.149 (0.094)	-0.0337 (0.084)	-0.253** (0.095)	-0.230* (0.093)	-0.123 (0.082)	-0.115 (0.088)
Interaction term (PEPFAR impact estimate)	0.0894 (0.061)	0.185** (0.060)	0.194* (0.091)	0.262** (0.086)	0.0350 (0.071)	0.172* (0.072)	0.0669 (0.095)	0.148 (0.090)	0.162 (0.096)	0.220* (0.092)	0.0121 (0.084)	0.194* (0.088)
Country income level (=1 if middle income)		0.0346 (0.029)		0.0849* (0.041)		-0.0451 (0.031)		0.0127 (0.036)		-0.0288 (0.038)		0.0358 (0.052)
Ln(BL Population)		-0.0174* (0.008)		-0.0265** (0.010)		-0.0103 (0.008)		-0.00208 (0.009)		-0.0105 (0.009)		-0.0194* (0.009)
Ln(BL other donor health spending per capita)		0.0168 (0.009)		-0.00133 (0.011)		0.0171 (0.010)		0.0137 (0.012)		0.0109 (0.010)		0.0109 (0.011)
Ln(BL domestic health spending per capita)		-0.0654** (0.023)		-0.0549 (0.030)		-0.102*** (0.029)		-0.0955** (0.029)		-0.140*** (0.032)		-0.0888* (0.036)
Ln(BL GDP per capita)		0.0164 (0.033)		-0.0127 (0.054)		0.0474 (0.035)		0.0470 (0.051)		0.0532 (0.041)		0.0240 (0.046)
Ln(BL HIV prevalence (% of population ages 15-49))		0.0130*** (0.004)		0.0252*** (0.006)		0.00269 (0.003)		0.00918 (0.005)		0.00156 (0.004)		0.0111* (0.005)
Ln(BL hepatitis B prevalence (% of population, under 5))		-0.0742*** (0.009)		-0.0683*** (0.013)		-0.0561*** (0.010)		-0.0618*** (0.012)		-0.0641*** (0.013)		-0.0455*** (0.013)
Ln(BL life expectancy at birth)		0.109 (0.111)		0.0343 (0.158)		0.601*** (0.168)		0.247 (0.134)		0.410 (0.217)		0.947*** (0.209)
Ln(BL Urban population (%))		0.0303 (0.025)		-0.0338 (0.028)		0.0879* (0.036)		-0.0553* (0.028)		0.125*** (0.035)		0.0304 (0.041)
Ln(BL School enrollment, secondary (% gross))		0.0153 (0.029)		0.0250 (0.041)		0.0291 (0.033)		0.0337 (0.038)		0.109* (0.047)		0.0543 (0.041)
Ln(BL Fertility rate (births per woman))		-0.136*** (0.036)		-0.184*** (0.052)		-0.118** (0.038)		-0.231*** (0.054)		-0.121* (0.052)		-0.144*** (0.041)
Recipient of US HIV aid before 2004 (=1 if yes)		0.00105 (0.022)		0.0784* (0.038)		-0.0232 (0.026)		-0.0458 (0.036)		0.0247 (0.037)		-0.110* (0.048)
Constant	4.247*** (0.034)	3.956*** (0.542)	4.247*** (0.034)	4.901*** (0.783)	4.247*** (0.034)	1.496* (0.729)	4.247*** (0.034)	3.459*** (0.736)	4.247*** (0.034)	1.948 (1.011)	4.247*** (0.034)	0.452 (0.855)

For peer review only

Adjusted R-squared	0.081	0.184	0.106	0.217	0.067	0.179	0.076	0.211	0.090	0.194	0.067	0.215
Observations	2799	2304	1789	1379	2270	1809	1744	1341	1809	1399	1766	1332

Notes: Standard errors in parentheses *** p<0.001, ** p<0.01, * p<0.05 BL=baseline; COP=country operational plans; PEPFAR= President's Emergency Plan for AIDS Relief

For peer review only

Table S4-3. Full model results for the indicator "Percent of children ages 12-23 months who received the measles vaccination"

Variables	All PEPFAR countries		COP countries		Non-COP PEPFAR countries		High-intensity PEPFAR countries		Medium-intensity PEPFAR countries		Low-intensity PEPFAR countries	
	Model 1	Model 2	Model 1	Model 2	Model 1	Model 2	Model 1	Model 2	Model 1	Model 2	Model 1	Model 2
Time variable (=1 post-2004)	0.0577***	0.0546***	0.0577***	0.0545***	0.0577***	0.0551***	0.0577***	0.0546***	0.0577***	0.0550***	0.0577***	0.0552***
	(0.010)	(0.008)	(0.010)	(0.009)	(0.010)	(0.008)	(0.010)	(0.008)	(0.010)	(0.008)	(0.010)	(0.008)
Intervention (=1 if PEPFAR)	-0.213***	-0.136***	-0.248***	-0.178***	-0.193***	-0.105***	-0.228***	-0.115***	-0.167***	-0.0911***	-0.241***	-0.116***
	(0.013)	(0.013)	(0.019)	(0.024)	(0.015)	(0.013)	(0.018)	(0.023)	(0.019)	(0.017)	(0.022)	(0.016)
Interaction term (PEPFAR impact estimate)	0.118***	0.124***	0.129***	0.143***	0.110***	0.115***	0.118***	0.105***	0.0769***	0.100***	0.155***	0.168***
	(0.016)	(0.013)	(0.022)	(0.020)	(0.018)	(0.014)	(0.022)	(0.019)	(0.022)	(0.018)	(0.026)	(0.018)
Country income level (=1 if middle income)		-0.0269*		-0.0130		-0.0541***		-0.00223		-0.0879***		-0.0434**
		(0.014)		(0.017)		(0.014)		(0.018)		(0.017)		(0.017)
Ln(BL Population)		-0.00658**		-0.0130***		-0.00953***		-0.00973**		-0.00599*		-0.0134***
		(0.002)		(0.003)		(0.002)		(0.003)		(0.003)		(0.002)
Ln(BL other donor health spending per capita)		0.0161***		0.00291		0.00384		0.00268		0.00734*		0.00190
		(0.003)		(0.004)		(0.003)		(0.004)		(0.003)		(0.003)
Ln(BL domestic health spending per capita)		0.0176		0.0314*		-0.0165		0.00623		0.0140		-0.0243
		(0.012)		(0.014)		(0.013)		(0.014)		(0.014)		(0.014)
Ln(BL GDP per capita)		-0.0122		-0.0256		-0.0198		-0.0247		-0.00894		-0.00176
		(0.013)		(0.019)		(0.014)		(0.016)		(0.014)		(0.016)
Ln(BL HIV prevalence (% of population ages 15-49))		0.0113***		0.0146***		0.00308**		0.0126***		0.00435***		0.00502***
		(0.001)		(0.002)		(0.001)		(0.002)		(0.001)		(0.001)
Ln(BL measles prevalence (% of population, under 5))		-0.00127		-0.0000882		0.00105		-0.00144		0.00140		0.00437***
		(0.001)		(0.001)		(0.001)		(0.001)		(0.001)		(0.001)
Ln(BL life expectancy at birth)		0.222***		0.0349		0.825***		0.117*		0.756***		0.963***
		(0.047)		(0.056)		(0.079)		(0.050)		(0.092)		(0.091)
Ln(BL Urban population (%))		-0.0360**		-0.0702***		0.0732***		-0.0522***		0.0364**		0.0420**
		(0.012)		(0.013)		(0.013)		(0.011)		(0.013)		(0.014)
Ln(BL School enrollment, secondary (% gross))		0.117***		0.0400*		0.136***		0.0641***		0.124***		0.132***
		(0.014)		(0.020)		(0.015)		(0.018)		(0.017)		(0.017)
Ln(BL Fertility rate (births per woman))		-0.222***		-0.287***		-0.115***		-0.246***		-0.107***		-0.138***
		(0.017)		(0.023)		(0.016)		(0.023)		(0.021)		(0.017)
Recipient of US HIV aid before 2004 (=1 if yes)		0.0595***		0.0299*		0.0604***		0.0219		0.0258		0.0444***
		(0.011)		(0.015)		(0.010)		(0.015)		(0.015)		(0.011)
Constant	4.427***	3.498***	4.427***	4.964***	4.427***	0.639*	4.427***	4.469***	4.427***	0.830*	4.427***	0.172
	(0.008)	(0.201)	(0.008)	(0.244)	(0.008)	(0.313)	(0.008)	(0.223)	(0.008)	(0.413)	(0.008)	(0.356)

For peer review only - http://bmjopen.bmj.com/site/about/guidelines.xhtml

For peer review only

Adjusted R-squared	0.129	0.441	0.160	0.394	0.111	0.548	0.145	0.374	0.090	0.435	0.129	0.615
Observations	4275	3540	2621	2024	3379	2728	2585	1986	2558	1992	2582	1986

Notes: Standard errors in parentheses *** p<0.001, ** p<0.01, * p<0.05 BL=baseline; COP=country operational plans; PEPFAR= President's Emergency Plan for AIDS Relief

For peer review only

Table S4-4. Full model results for the indicator "Percentage of births by women of child-bearing age who are immunized against tetanus"

Variables	All PEPFAR countries		COP countries		Non-COP PEPFAR countries		High-intensity PEPFAR countries		Medium-intensity PEPFAR countries		Low-intensity PEPFAR countries	
	Model 1	Model 2	Model 1	Model 2	Model 1	Model 2	Model 1	Model 2	Model 1	Model 2	Model 1	Model 2
Time variable (=1 post-2004)	0.233***	0.243***	0.233***	0.247***	0.233***	0.243***	0.233***	0.245***	0.233***	0.237***	0.233***	0.254***
	(0.022)	(0.023)	(0.022)	(0.022)	(0.022)	(0.023)	(0.022)	(0.023)	(0.022)	(0.023)	(0.022)	(0.022)
Intervention (=1 if PEPFAR)	-0.109***	-0.0370	-0.0992***	-0.112**	-0.115***	-0.0228	-0.109***	-0.0966*	-0.0835**	-0.0452	-0.130***	0.0334
	(0.025)	(0.027)	(0.029)	(0.038)	(0.027)	(0.029)	(0.029)	(0.042)	(0.032)	(0.034)	(0.032)	(0.031)
Interaction term (PEPFAR impact estimate)	0.109***	0.0978***	0.0918**	0.0747*	0.122***	0.111***	0.109***	0.0954**	0.0979**	0.0958**	0.119***	0.0973**
	(0.026)	(0.026)	(0.031)	(0.031)	(0.029)	(0.029)	(0.031)	(0.032)	(0.034)	(0.034)	(0.035)	(0.031)
Country income level (=1 if middle income)		-0.00739		0.00782		-0.0485		0.0394		-0.00567		-0.0985**
		(0.021)		(0.026)		(0.030)		(0.027)		(0.036)		(0.033)
Ln(BL Population)		-0.0240***		-0.0268***		-0.0245***		-0.0302***		-0.0358***		-0.0181**
		(0.005)		(0.006)		(0.006)		(0.007)		(0.007)		(0.006)
Ln(BL other donor health spending per capita)		0.00245		-0.00663		0.00546		-0.00149		-0.00557		0.0162*
		(0.006)		(0.008)		(0.007)		(0.008)		(0.008)		(0.007)
Ln(BL domestic health spending per capita)		-0.0446**		-0.0858***		-0.0293		-0.0217		-0.0175		-0.0821***
		(0.016)		(0.022)		(0.021)		(0.029)		(0.025)		(0.024)
Ln(BL GDP per capita)		0.0601***		0.134***		0.0598***		0.0645*		0.00203		0.196***
		(0.017)		(0.025)		(0.018)		(0.029)		(0.022)		(0.023)
Ln(BL HIV prevalence (% of population ages 15-49))		-0.00119		-0.00516		0.000978		0.00249		-0.000466		-0.000968
		(0.002)		(0.003)		(0.003)		(0.004)		(0.003)		(0.003)
Ln(BL life expectancy at birth)		-0.0369		-0.154		-0.00327		0.0562		-0.379**		0.134
		(0.073)		(0.111)		(0.117)		(0.095)		(0.130)		(0.154)
Ln(BL Urban population (%))		-0.0261		-0.115***		-0.00585		-0.107***		0.0509*		-0.162***
		(0.017)		(0.022)		(0.025)		(0.020)		(0.025)		(0.026)
Ln(BL School enrollment, secondary (% gross))		-0.0258		-0.0871***		-0.00317		-0.0797**		-0.00822		-0.0253
		(0.018)		(0.026)		(0.026)		(0.027)		(0.042)		(0.032)
Ln(BL Fertility rate (births per woman))		-0.264***		-0.313***		-0.291***		-0.204***		-0.342***		-0.398***
		(0.032)		(0.048)		(0.036)		(0.053)		(0.044)		(0.042)
Recipient of US HIV aid before 2004 (=1 if yes)		0.0615***		0.182***		0.0185		0.132***		0.0427		0.0566**
		(0.016)		(0.028)		(0.017)		(0.028)		(0.024)		(0.022)
Constant	4.174***	4.900***	4.174***	5.621***	4.174***	4.585***	4.174***	4.865***	4.174***	6.625***	4.174***	3.812***
	(0.021)	(0.361)	(0.021)	(0.545)	(0.021)	(0.488)	(0.021)	(0.502)	(0.021)	(0.574)	(0.021)	(0.604)
Adjusted R-squared	0.217	0.289	0.187	0.267	0.216	0.317	0.201	0.265	0.184	0.265	0.191	0.389
Observations	2830	2657	1624	1480	1964	1848	1614	1441	1336	1249	1396	1309

Notes: Standard errors in parentheses *** p<0.001, ** p<0.01, * p<0.05 BL=baseline; COP=country operational plans; PEPFAR= President's Emergency Plan for AIDS Relief

For peer review only

For peer review only - http://bmjopen.bmj.com/site/about/guidelines.xhtml

Table S4-5. Full model results for the indicator "Number of women who die from pregnancy-related causes while pregnant or within 42 days of pregnancy termination per 100,000 live births"

Variables	All PEPFAR countries		COP countries		Non-COP PEPFAR countries		High-intensity PEPFAR countries		Medium-intensity PEPFAR countries		Low-intensity PEPFAR countries	
	Model 1	Model 2	Model 1	Model 2	Model 1	Model 2	Model 1	Model 2	Model 1	Model 2	Model 1	Model 2
Time variable (=1 post-2004)	-0.289***<br>(0.083)	-0.272***<br>(0.049)	-0.289***<br>(0.083)	-0.272***<br>(0.050)	-0.289***<br>(0.083)	-0.272***<br>(0.047)	-0.289***<br>(0.083)	-0.272***<br>(0.049)	-0.289***<br>(0.083)	-0.272***<br>(0.046)	-0.289***<br>(0.083)	-0.272***<br>(0.046)
Intervention (=1 if PEPFAR)	1.497***<br>(0.098)	0.179**<br>(0.055)	2.037***<br>(0.104)	0.480***<br>(0.083)	1.213***<br>(0.114)	0.0921<br>(0.057)	2.080***<br>(0.104)	0.332***<br>(0.077)	1.195***<br>(0.141)	0.00660<br>(0.062)	1.216***<br>(0.142)	0.146<br>(0.075)
Interaction term (PEPFAR impact estimate)	-0.0337<br>(0.112)	-0.0358<br>(0.058)	-0.0171<br>(0.118)	-0.0504<br>(0.064)	-0.0424<br>(0.130)	-0.0282<br>(0.062)	-0.00368<br>(0.118)	-0.0275<br>(0.062)	-0.0530<br>(0.160)	-0.0612<br>(0.068)	-0.0444<br>(0.163)	-0.0177<br>(0.078)
Country income level (=1 if middle income)		-0.188***<br>(0.040)		-0.174**<br>(0.053)		-0.0875<br>(0.046)		-0.295***<br>(0.048)		-0.0132<br>(0.050)		-0.141*<br>(0.064)
Ln(BL Population)		-0.00714<br>(0.010)		-0.0187<br>(0.012)		-0.0438***<br>(0.010)		-0.0513***<br>(0.012)		-0.0937***<br>(0.010)		-0.0401**<br>(0.013)
Ln(BL other donor health spending per capita)		-0.0201<br>(0.014)		-0.0162<br>(0.017)		-0.0392**<br>(0.015)		-0.0275<br>(0.018)		-0.0891***<br>(0.014)		-0.0351*<br>(0.018)
Ln(BL domestic health spending per capita)		-0.202***<br>(0.029)		-0.148***<br>(0.041)		-0.244***<br>(0.035)		-0.0176<br>(0.046)		-0.307***<br>(0.035)		-0.318***<br>(0.046)
Ln(BL GDP per capita)		0.101***<br>(0.030)		0.0177<br>(0.046)		0.0579<br>(0.032)		-0.0985*<br>(0.045)		0.0429<br>(0.036)		0.0300<br>(0.039)
Ln(BL HIV prevalence (% of population ages 15-49))		0.0384***<br>(0.005)		0.0153*<br>(0.006)		0.0467***<br>(0.004)		0.0331***<br>(0.006)		0.0366***<br>(0.005)		0.0337***<br>(0.006)
Ln(BL life expectancy at birth)		-2.348***<br>(0.105)		-1.986***<br>(0.156)		-2.147***<br>(0.199)		-1.969***<br>(0.126)		-2.663***<br>(0.216)		-0.828**<br>(0.263)
Ln(BL Urban population (%))		0.0605*<br>(0.029)		0.0170<br>(0.035)		0.127**<br>(0.041)		0.0255<br>(0.035)		0.133**<br>(0.042)		0.129*<br>(0.052)
Ln(BL School enrollment, secondary (% gross))		-0.238***<br>(0.044)		-0.141**<br>(0.052)		-0.509***<br>(0.054)		-0.141**<br>(0.051)		-0.981***<br>(0.066)		-0.462***<br>(0.057)
Ln(BL Fertility rate (births per woman))		1.030***<br>(0.055)		0.856***<br>(0.094)		0.948***<br>(0.059)		1.007***<br>(0.093)		0.494***<br>(0.068)		1.100***<br>(0.080)
Recipient of US HIV aid before 2004 (=1 if yes)		-0.0728*<br>(0.032)		-0.00921<br>(0.044)		-0.0185<br>(0.038)		-0.0615<br>(0.043)		0.0313<br>(0.041)		0.123<br>(0.064)
Constant	4.004***<br>(0.073)	14.78***<br>(0.528)	4.004***<br>(0.073)	13.68***<br>(0.845)	4.004***<br>(0.073)	16.03***<br>(0.817)	4.004***<br>(0.073)	14.37***<br>(0.725)	4.004***<br>(0.073)	21.82***<br>(0.969)	4.004***<br>(0.073)	10.69***<br>(1.021)
Adjusted R-squared	0.263	0.822	0.478	0.821	0.187	0.816	0.492	0.836	0.183	0.809	0.179	0.785
Observations	2628	2178	1566	1206	2070	1674	1548	1188	1548	1206	1548	1188

For peer review only - http://bmjopen.bmj.com/site/about/guidelines.xhtml

Notes: Standard errors in parentheses *** p<0.001, ** p<0.01, * p<0.05 BL=baseline; COP=country operational plans; PEPFAR= President's Emergency Plan for AIDS Relief

For peer review only

Table S4-6. Full model results for the indicator "Probability of a child dying between birth and 5 years of age, per 1,000 live births"

Variables	All PEPFAR countries		COP countries		Non-COP PEPFAR countries		High-intensity PEPFAR countries		Medium-intensity PEPFAR countries		Low-intensity PEPFAR countries	
	Model 1	Model 2	Model 1	Model 2	Model 1	Model 2	Model 1	Model 2	Model 1	Model 2	Model 1	Model 2
Time variable (=1 post-2004)	-0.526***	-0.479***	-0.526***	-0.479***	-0.526***	-0.479***	-0.526***	-0.479***	-0.526***	-0.479***	-0.526***	-0.479***
	(0.035)	(0.025)	(0.035)	(0.025)	(0.035)	(0.024)	(0.035)	(0.024)	(0.035)	(0.023)	(0.035)	(0.023)
Intervention (=1 if PEPFAR)	1.013***	0.162***	1.309***	0.188***	0.857***	0.0812**	1.337***	0.0916*	0.821***	0.0452	0.881***	-0.00393
	(0.033)	(0.025)	(0.037)	(0.045)	(0.037)	(0.025)	(0.037)	(0.045)	(0.047)	(0.027)	(0.045)	(0.031)
Interaction term (PEPFAR impact estimate)	-0.0390	-0.0860**	0.00754	-0.0516	-0.0634	-0.104***	-0.00887	-0.0759*	-0.0656	-0.0963**	-0.0425	-0.0854**
	(0.047)	(0.027)	(0.051)	(0.032)	(0.054)	(0.028)	(0.052)	(0.032)	(0.068)	(0.030)	(0.066)	(0.032)
Country income level (=1 if middle income)		-0.206***		-0.0972**		-0.240***		-0.0970**		-0.139***		-0.326***
		(0.021)		(0.033)		(0.026)		(0.033)		(0.031)		(0.037)
Ln(BL Population)		0.0165***		0.0165**		0.0161**		0.000547		-0.00462		0.0136*
		(0.004)		(0.006)		(0.005)		(0.007)		(0.006)		(0.006)
Ln(BL other donor health spending per capita)		-0.0176**		0.000581		-0.0215**		-0.0148		-0.0362***		-0.0123
		(0.007)		(0.008)		(0.007)		(0.008)		(0.007)		(0.008)
Ln(BL domestic health spending per capita)		-0.116***		-0.139***		-0.0719***		-0.0884**		-0.117***		-0.0820***
		(0.015)		(0.026)		(0.018)		(0.029)		(0.023)		(0.023)
Ln(BL GDP per capita)		-0.0146		0.0262		-0.0479*		-0.0577		-0.0125		-0.0515
		(0.020)		(0.037)		(0.021)		(0.036)		(0.024)		(0.029)
Ln(BL HIV prevalence (% of population ages 15-49))		-0.0205***		-0.0314***		-0.0174***		-0.0228***		-0.0229***		-0.0187***
		(0.003)		(0.004)		(0.003)		(0.004)		(0.003)		(0.004)
Ln(BL life expectancy at birth)		-2.372***		-2.435***		-3.076***		-2.526***		-3.689***		-2.983***
		(0.072)		(0.112)		(0.117)		(0.102)		(0.140)		(0.187)
Ln(BL Urban population (%))		0.199***		0.204***		0.150***		0.239***		0.149***		0.192***
		(0.016)		(0.022)		(0.022)		(0.023)		(0.022)		(0.028)
Ln(BL School enrollment, secondary (% gross))		-0.139***		-0.212***		-0.128***		-0.187***		-0.309***		-0.142***
		(0.020)		(0.032)		(0.023)		(0.032)		(0.034)		(0.029)
Ln(BL Fertility rate (births per woman))		0.483***		0.367***		0.423***		0.450***		0.198***		0.451***
		(0.027)		(0.050)		(0.029)		(0.050)		(0.035)		(0.042)
Recipient of US HIV aid before 2004 (=1 if yes)		0.0436**		0.100***		0.126***		0.135***		0.167***		0.273***
		(0.014)		(0.025)		(0.016)		(0.029)		(0.021)		(0.031)
Constant	3.347***	13.62***	3.347***	13.91***	3.347***	16.90***	3.347***	14.70***	3.347***	20.64***	3.347***	16.56***
	(0.024)	(0.344)	(0.024)	(0.561)	(0.024)	(0.477)	(0.024)	(0.534)	(0.024)	(0.585)	(0.024)	(0.744)
Adjusted R-squared	0.343	0.847	0.487	0.814	0.281	0.841	0.492	0.817	0.256	0.816	0.283	0.808
Observations	4408	3596	2697	2030	3509	2784	2668	2001	2668	2030	2668	2001

Notes: Standard errors in parentheses *** p<0.001, ** p<0.01, * p<0.05 BL=baseline; COP=country operational plans; PEPFAR= President's Emergency Plan for AIDS Relief

For peer review only

Table S4-7. Full model results for the indicator "Prevalence of anemia among women of reproductive age (% of women ages 15-49)"

Variables	All PEPFAR countries		COP countries		Non-COP PEPFAR countries		High-intensity PEPFAR countries		Medium-intensity PEPFAR countries		Low-intensity PEPFAR countries	
	Model 1	Model 2	Model 1	Model 2	Model 1	Model 2	Model 1	Model 2	Model 1	Model 2	Model 1	Model 2
Time variable (=1 post-2004)	-0.176***<br>(0.015)	-0.206***<br>(0.017)	-0.176***<br>(0.015)	-0.206***<br>(0.017)	-0.176***<br>(0.015)	-0.206***<br>(0.016)	-0.176***<br>(0.015)	-0.206***<br>(0.017)	-0.176***<br>(0.015)	-0.206***<br>(0.015)	-0.176***<br>(0.015)	-0.206***<br>(0.016)
Intervention (=1 if PEPFAR)	0.227***<br>(0.014)	0.0302<br>(0.017)	0.294***<br>(0.016)	0.106***<br>(0.029)	0.192***<br>(0.016)	-0.0199<br>(0.017)	0.286***<br>(0.016)	0.0870**<br>(0.031)	0.201***<br>(0.021)	-0.0353<br>(0.019)	0.193***<br>(0.020)	-0.0345<br>(0.022)
Interaction term (PEPFAR impact estimate)	-0.0116<br>(0.021)	0.0140<br>(0.020)	-0.0224<br>(0.024)	0.00718<br>(0.024)	-0.00592<br>(0.024)	0.0176<br>(0.020)	-0.0409<br>(0.025)	-0.0106<br>(0.025)	-0.000701<br>(0.030)	0.0219<br>(0.023)	0.00682<br>(0.029)	0.0305<br>(0.022)
Country income level (=1 if middle income)		-0.258***<br>(0.020)		-0.202***<br>(0.026)		-0.234***<br>(0.025)		-0.241***<br>(0.027)		-0.124***<br>(0.028)		-0.265***<br>(0.033)
BL Population		-0.000913<br>(0.003)		-0.00223<br>(0.004)		0.00358<br>(0.003)		-0.00821<br>(0.004)		-0.0144***<br>(0.004)		0.0100**<br>(0.004)
BL other donor health spending per capita		-0.0318***<br>(0.004)		-0.0244***<br>(0.005)		-0.0336***<br>(0.004)		-0.0216***<br>(0.005)		-0.0662***<br>(0.005)		-0.0287***<br>(0.005)
BL domestic health spending per capita		-0.0960***<br>(0.011)		-0.0535***<br>(0.016)		-0.0606***<br>(0.014)		-0.0166<br>(0.017)		-0.0231<br>(0.017)		-0.0766***<br>(0.015)
BL GDP per capita		0.143***<br>(0.012)		0.181***<br>(0.016)		0.129***<br>(0.012)		0.171***<br>(0.016)		0.0557***<br>(0.015)		0.156***<br>(0.011)
BL HIV prevalence (% of population ages 15-49)		-0.0198***<br>(0.002)		-0.0357***<br>(0.003)		-0.0142***<br>(0.002)		-0.0335***<br>(0.003)		-0.0229***<br>(0.002)		-0.0170***<br>(0.002)
BL life expectancy at birth		-0.584***<br>(0.048)		-0.237***<br>(0.068)		-1.234***<br>(0.077)		-0.283***<br>(0.063)		-1.371***<br>(0.104)		-0.958***<br>(0.088)
BL Urban population (%)		0.0726***<br>(0.014)		-0.0212<br>(0.019)		-0.0464**<br>(0.017)		-0.0150<br>(0.020)		-0.0873***<br>(0.020)		-0.111***<br>(0.021)
BL School enrollment, secondary (% gross)		-0.0676***<br>(0.014)		-0.148***<br>(0.024)		-0.0668***<br>(0.017)		-0.173***<br>(0.024)		-0.302***<br>(0.025)		-0.0469*<br>(0.021)
BL Fertility rate (births per woman)		0.243***<br>(0.020)		0.222***<br>(0.033)		0.168***<br>(0.021)		0.230***<br>(0.034)		0.0626*<br>(0.026)		0.206***<br>(0.028)
Recipient of US HIV aid before 2004 (=1 if yes)		-0.0162<br>(0.012)		0.137***<br>(0.018)		-0.00280<br>(0.014)		0.120***<br>(0.021)		0.0369*<br>(0.019)		0.0342<br>(0.022)
Constant	3.449***<br>(0.011)	5.184***<br>(0.233)	3.449***<br>(0.011)	3.748***<br>(0.345)	3.449***<br>(0.011)	8.333***<br>(0.327)	3.449***<br>(0.011)	4.024***<br>(0.340)	3.449***<br>(0.011)	10.77***<br>(0.445)	3.449***<br>(0.011)	7.049***<br>(0.359)
Adjusted R-squared	0.159	0.524	0.238	0.449	0.129	0.590	0.221	0.442	0.134	0.580	0.135	0.560
Observations	3996	3321	2403	1863	3159	2565	2376	1836	2376	1863	2376	1836

Notes: Standard errors in parentheses *** p<0.001, ** p<0.01, * p<0.05 BL=baseline; COP=country operational plans; PEPFAR= President's Emergency Plan for AIDS Relief

For peer review only

S5. Full model of unlogged regression of seven outcomes from staggered DID

For peer review only

Table S5-1. Full model results for the indicator "Percent of children ages 12-23 months who received DPT vaccinations (3 doses)"

Variables	All PEPFAR countries		COP countries		Non-COP PEPFAR countries		High-intensity PEPFAR countries		Medium-intensity PEPFAR countries		Low-intensity PEPFAR countries	
	Model 1	Model 2	Model 1	Model 2	Model 1	Model 2	Model 1	Model 2	Model 1	Model 2	Model 1	Model 2
Intervention (=1 if PEPFAR)	-14.85***<br>(0.802)	-4.304***<br>(0.559)	-17.09***<br>(1.199)	-10.68***<br>(1.206)	-14.45***<br>(0.922)	-3.359***<br>(0.638)	-16.66***<br>(1.240)	-5.981***<br>(1.266)	-10.83***<br>(1.103)	-3.004***<br>(0.858)	-18.07***<br>(1.253)	-4.129***<br>(0.852)
Treatment (=1 for the years PEPFAR starts and after)	8.206***<br>(0.992)	7.328***<br>(0.593)	6.538***<br>(1.461)	7.459***<br>(0.911)	10.80***<br>(1.104)	8.334***<br>(0.681)	8.373***<br>(1.498)	8.326***<br>(0.899)	4.149**<br>(1.364)	6.836***<br>(0.964)	14.49***<br>(1.473)	8.979***<br>(0.875)
Country income level (=1 if middle income)		-4.911***<br>(0.649)		-6.629***<br>(0.850)		-2.615***<br>(0.765)		-5.702***<br>(0.883)		-1.832*<br>(0.871)		-2.634**<br>(0.980)
BL Population		-1.75e-09<br>(0.000)		-7.40e-09***<br>(0.000)		-2.10e-09*<br>(0.000)		-1.05e-08<br>(0.000)		-5.58e-08***<br>(0.000)		-3.31e-09***<br>(0.000)
BL other donor health spending per capita		0.00318<br>(0.008)		-0.0196*<br>(0.008)		-0.00465<br>(0.008)		-0.0154<br>(0.008)		-0.0168*<br>(0.008)		-0.0180*<br>(0.009)
BL domestic health spending per capita		-0.00474***<br>(0.001)		-0.00302*<br>(0.001)		-0.00608***<br>(0.001)		-0.00608***<br>(0.001)		-0.00777***<br>(0.001)		-0.00396**<br>(0.001)
BL GDP per capita		0.000120<br>(0.000)		-0.00000602<br>(0.000)		0.000131*<br>(0.000)		0.0000389<br>(0.000)		0.000180***<br>(0.000)		0.0000538<br>(0.000)
BL HIV prevalence (% of population ages 15-49)		0.823***<br>(0.070)		0.680***<br>(0.088)		-0.675*<br>(0.316)		0.478***<br>(0.077)		-1.646**<br>(0.567)		-1.378***<br>(0.415)
BL diphtheria prevalence (% of population, under 5)		-3820.2***<br>(537.869)		-5143.0***<br>(573.583)		-4092.6***<br>(895.923)		-4071.4***<br>(666.350)		-1930.0<br>(1677.185)		-4972.3***<br>(944.550)
BL tetanus prevalence (% of population, under 5)		-341.2**<br>(109.133)		389.8**<br>(135.183)		-526.3***<br>(129.114)		-72.51<br>(158.000)		-44.21<br>(147.707)		-921.9***<br>(136.270)
BL pertussis (whooping cough) prevalence (% of population, under 5)		-2568.6***<br>(112.485)		-2581.9***<br>(144.914)		-2080.6***<br>(122.128)		-2806.0***<br>(136.893)		-2212.6***<br>(149.232)		-1976.4***<br>(120.957)
BL life expectancy at birth		0.639***<br>(0.066)		0.324***<br>(0.085)		0.455***<br>(0.070)		0.306***<br>(0.071)		0.448***<br>(0.082)		0.216**<br>(0.081)
BL Urban population (%)		-0.0291*<br>(0.012)		-0.0380**<br>(0.013)		-0.00745<br>(0.012)		-0.0349**<br>(0.012)		0.0345**<br>(0.013)		-0.0406***<br>(0.012)
BL School enrollment, secondary (% gross)		0.0804***<br>(0.014)		0.0725***<br>(0.017)		0.0768***<br>(0.015)		0.0435**<br>(0.016)		0.0685***<br>(0.016)		0.0316*<br>(0.016)
BL Fertility rate (births per woman)		-0.552<br>(0.282)		-1.485***<br>(0.388)		-1.434***<br>(0.287)		-2.477***<br>(0.357)		-0.920*<br>(0.362)		-3.016***<br>(0.307)

For peer review only

For peer review only - http://bmjopen.bmj.com/site/about/guidelines.xhtml

Recipient of US HIV aid before 2004 (=1 if yes)		-1.621***<br>(0.487)		-0.356<br>(0.737)		-2.125***<br>(0.593)		-1.480<br>(0.871)		-2.957***<br>(0.808)		-2.217**<br>(0.726)
Constant	84.78***<br>(1.761)	53.74***<br>(5.391)	86.15***<br>(1.800)	83.46***<br>(6.642)	85.39***<br>(1.971)	65.45***<br>(5.862)	86.68***<br>(1.800)	90.42***<br>(5.691)	84.99***<br>(2.062)	64.31***<br>(6.481)	86.34***<br>(2.002)	93.42***<br>(6.346)
Adjusted R-squared	0.152	0.663	0.173	0.668	0.140	0.670	0.156	0.695	0.094	0.572	0.160	0.719
Observations	4282	3926	2628	2328	3386	3087	2592	2263	2565	2322	2589	2319

Notes: Standard errors in parentheses *** p<0.001, ** p<0.01, * p<0.05 BL=baseline; COP=country operational plans; PEPFAR= President's Emergency Plan for AIDS Relief

For peer review only

Table S5-2. Full model results for the indicator "Percentage of children age 12-23 months who received hepatitis B vaccines, 3 doses) "

Variables	All PEPFAR countries		COP countries		Non-COP PEPFAR countries		High-intensity PEPFAR countries		Medium-intensity PEPFAR countries		Low-intensity PEPFAR countries	
	**Model 1**	**Model 2**	**Model 1**	**Model 2**	**Model 1**	**Model 2**	**Model 1**	**Model 2**	**Model 1**	**Model 2**	**Model 1**	**Model 2**
Intervention (=1 if PEPFAR)	-12.21***	-7.517***	-26.51***	-26.10***	-7.571***	-3.643	-17.68***	-12.76**	-10.08***	-7.392**	-12.64***	-4.294
	(1.898)	(1.755)	(3.197)	(3.375)	(2.115)	(1.943)	(4.480)	(4.488)	(2.490)	(2.572)	(3.120)	(2.746)
Treatment (=1 for the years PEPFAR starts and after)	5.436**	10.52***	16.33***	21.33***	3.000	7.050***	10.78*	17.15***	3.096	6.967**	6.397	9.912***
	(1.963)	(1.827)	(3.311)	(3.220)	(2.205)	(2.076)	(4.545)	(4.303)	(2.624)	(2.600)	(3.277)	(2.839)
Country income level (=1 if middle income)		-3.539**		-6.157***		-3.752**		-5.545***		-4.939**		-3.550
		(1.140)		(1.676)		(1.337)		(1.633)		(1.605)		(1.916)
BL Population		-1.17e-08**		-3.30e-08***		-2.41e-09		-0.000000104***		-8.32e-08***		-1.54e-08***
		(0.000)		(0.000)		(0.000)		(0.000)		(0.000)		(0.000)
BL other donor health spending per capita		0.0312**		0.0230		0.0277*		0.0194		0.0191		0.0509***
		(0.011)		(0.012)		(0.012)		(0.012)		(0.012)		(0.013)
BL domestic health spending per capita		-0.00869**		-0.00398		-0.0113***		-0.00652*		-0.0112***		-0.0104***
		(0.003)		(0.003)		(0.003)		(0.003)		(0.003)		(0.003)
BL GDP per capita		0.000426***		0.000390**		0.000446***		0.000389**		0.000441***		0.000530***
		(0.000)		(0.000)		(0.000)		(0.000)		(0.000)		(0.000)
BL HIV prevalence (% of population ages 15-49)		0.713***		0.751***		-0.0858		0.235		-0.233		-1.404
		(0.109)		(0.140)		(0.658)		(0.124)		(1.231)		(0.890)
BL hepatitis B prevalence (% of population, under 5)		-75.75***		-83.95***		-62.05***		-84.02***		-77.59***		-3.689
		(6.747)		(10.895)		(9.734)		(9.175)		(11.448)		(15.177)
BL life expectancy at birth		0.566***		0.370*		0.307**		0.227		0.453**		0.573***
		(0.107)		(0.161)		(0.115)		(0.143)		(0.151)		(0.143)
BL Urban population (%)		-0.0318		-0.116***		-0.00460		-0.0875***		0.0106		-0.0554*
		(0.021)		(0.023)		(0.023)		(0.022)		(0.026)		(0.024)
BL School enrollment, secondary (% gross)		0.0864***		0.0847*		0.0998***		0.0779*		0.129***		0.144***
		(0.024)		(0.035)		(0.026)		(0.033)		(0.030)		(0.030)
BL Fertility rate (births per woman)		0.0261		-1.001		-1.844***		-2.578***		-0.554		-3.787***
		(0.477)		(0.640)		(0.501)		(0.655)		(0.624)		(0.634)
Recipient of US HIV aid before 2004 (=1 if yes)		-2.917***		3.818**		-2.991**		1.791		-0.307		-4.525*
		(0.851)		(1.361)		(1.100)		(1.462)		(1.535)		(1.775)
Constant	58.54***	19.80	60.92***	45.55**	63.80***	46.74**	63.80***	63.54***	58.18***	27.72	63.80***	29.63
	(12.180)	(13.776)	(11.556)	(16.530)	(13.426)	(15.388)	(13.454)	(16.797)	(12.354)	(15.964)	(13.452)	(16.476)
Adjusted R-squared	0.138	0.341	0.218	0.410	0.114	0.324	0.150	0.390	0.159	0.317	0.136	0.377
Observations	2799	2579	1789	1598	2270	2065	1744	1541	1809	1633	1766	1573

For peer review only

For peer review only - http://bmjopen.bmj.com/site/about/guidelines.xhtml

Notes: Standard errors in parentheses *** p<0.001, ** p<0.01, * p<0.05 BL=baseline; COP=country operational plans; PEPFAR= President's Emergency Plan for AIDS Relief

For peer review only

Table S5-3. Full model results for the indicator "Percent of children ages 12-23 months who received the measles vaccination"

Variables	All PEPFAR countries		COP countries		Non-COP PEPFAR countries		High-intensity PEPFAR countries		Medium-intensity PEPFAR countries		Low-intensity PEPFAR countries	
	Model 1	Model 2	Model 1	Model 2	Model 1	Model 2	Model 1	Model 2	Model 1	Model 2	Model 1	Model 2
Intervention (=1 if PEPFAR)	-14.30***<br>(0.773)	-5.796***<br>(0.636)	-16.04***<br>(1.118)	-10.16***<br>(1.255)	-13.97***<br>(0.885)	-4.759***<br>(0.708)	-15.53***<br>(1.119)	-9.263***<br>(1.283)	-10.96***<br>(1.079)	-5.385***<br>(1.000)	-17.04***<br>(1.194)	-4.471***<br>(0.841)
Treatment (=1 for the years PEPFAR starts and after)	6.816***<br>(0.952)	5.515***<br>(0.677)	5.091***<br>(1.344)	5.546***<br>(0.997)	9.077***<br>(1.089)	6.115***<br>(0.753)	5.692***<br>(1.355)	5.158***<br>(0.970)	3.280*<br>(1.365)	5.357***<br>(1.036)	13.28***<br>(1.417)	7.083***<br>(0.885)
Country income level (=1 if middle income)		-5.795***<br>(0.635)		-7.742***<br>(0.835)		-4.200***<br>(0.799)		-7.702***<br>(0.893)		-6.163***<br>(0.989)		-3.973***<br>(0.974)
BL Population		-4.03e-09***<br>(0.000)		-9.32e-09***<br>(0.000)		-1.48e-09<br>(0.000)		-7.25e-08***<br>(0.000)		-1.38e-08<br>(0.000)		-6.01e-09***<br>(0.000)
BL other donor health spending per capita		0.0581***<br>(0.009)		0.0501***<br>(0.010)		0.0399***<br>(0.009)		0.0360***<br>(0.009)		0.0373***<br>(0.009)		0.0456***<br>(0.009)
BL domestic health spending per capita		-0.00649***<br>(0.001)		-0.00679***<br>(0.001)		-0.00476***<br>(0.001)		-0.00518***<br>(0.001)		-0.00418**<br>(0.001)		-0.00523***<br>(0.001)
BL GDP per capita		0.000250***<br>(0.000)		0.000295***<br>(0.000)		0.000178***<br>(0.000)		0.000226***<br>(0.000)		0.000167**<br>(0.000)		0.000264***<br>(0.000)
BL HIV prevalence (% of population ages 15-49)		1.212***<br>(0.067)		1.168***<br>(0.075)		-0.117<br>(0.318)		1.056***<br>(0.076)		0.194<br>(0.565)		0.179<br>(0.395)
BL measles prevalence (% of population, under 5)		-17.01***<br>(1.341)		-21.20***<br>(1.657)		-5.621***<br>(1.554)		-20.76***<br>(1.681)		-0.661<br>(1.794)		-11.01***<br>(1.774)
BL life expectancy at birth		0.839***<br>(0.062)		0.724***<br>(0.075)		0.638***<br>(0.071)		0.705***<br>(0.072)		0.732***<br>(0.086)		0.646***<br>(0.079)
BL Urban population (%)		-0.0128<br>(0.013)		-0.0545***<br>(0.012)		0.0294*<br>(0.013)		-0.0415***<br>(0.012)		0.0301*<br>(0.013)		-0.0286*<br>(0.012)
BL School enrollment, secondary (% gross)		0.0855***<br>(0.013)		0.0562***<br>(0.017)		0.131***<br>(0.014)		0.0772***<br>(0.016)		0.122***<br>(0.017)		0.126***<br>(0.015)
BL Fertility rate (births per woman)		-2.767***<br>(0.275)		-3.176***<br>(0.349)		-3.321***<br>(0.292)		-2.496***<br>(0.352)		-3.439***<br>(0.375)		-3.855***<br>(0.288)
Recipient of US HIV aid before 2004 (=1 if yes)		1.307*<br>(0.524)		2.753**<br>(0.842)		2.090***<br>(0.587)		3.695***<br>(0.907)		1.434<br>(0.880)		1.588*<br>(0.765)
Constant	82.28***<br>(1.616)	32.25***<br>(5.134)	82.36***<br>(1.775)	48.39***<br>(5.926)	82.88***<br>(1.811)	39.84***<br>(5.903)	83.19***<br>(1.797)	46.44***<br>(5.851)	81.93***<br>(1.890)	35.17***<br>(6.957)	82.77***<br>(1.952)	44.05***<br>(6.303)
Adjusted R-squared	0.160	0.588	0.190	0.574	0.143	0.615	0.174	0.574	0.112	0.519	0.162	0.680
Observations	4275	3926	2621	2328	3379	3087	2585	2263	2558	2322	2582	2319

Notes: Standard errors in parentheses *** p<0.001, ** p<0.01, * p<0.05 BL=baseline; COP=country operational plans; PEPFAR= President's Emergency Plan for AIDS Relief

For peer review only

For peer review only - http://bmjopen.bmj.com/site/about/guidelines.xhtml

Table S5-4. Full model results for the indicator "Percentage of births by women of child-bearing age who are immunized against tetanus"

Variables	All PEPFAR countries		COP countries		Non-COP PEPFAR countries		High-intensity PEPFAR countries		Medium-intensity PEPFAR countries		Low-intensity PEPFAR countries	
	**Model 1**	**Model 2**	**Model 1**	**Model 2**	**Model 1**	**Model 2**	**Model 1**	**Model 2**	**Model 1**	**Model 2**	**Model 1**	**Model 2**
Intervention (=1 if PEPFAR)	-5.962***<br>(1.037)	1.698<br>(1.093)	-6.498***<br>(1.422)	-3.279<br>(1.790)	-6.051***<br>(1.151)	3.261**<br>(1.233)	-7.470***<br>(1.394)	-3.572<br>(1.887)	-4.661**<br>(1.574)	0.572<br>(1.674)	-6.488***<br>(1.349)	6.202***<br>(1.266)
Treatment (=1 for the years PEPFAR starts and after)	5.218***<br>(1.049)	3.619***<br>(1.014)	5.444***<br>(1.518)	4.053**<br>(1.447)	5.942***<br>(1.228)	4.103***<br>(1.170)	7.005***<br>(1.483)	5.475***<br>(1.446)	4.966**<br>(1.720)	5.391**<br>(1.638)	5.401***<br>(1.474)	1.022<br>(1.242)
Country income level (=1 if middle income)		0.0616<br>(0.888)		2.662*<br>(1.126)		0.277<br>(1.337)		1.422<br>(1.267)		-0.621<br>(1.786)		-0.364<br>(1.583)
BL Population		7.32e-09***<br>(0.000)		4.12e-09*<br>(0.000)		3.11e-09<br>(0.000)		-9.92e-08***<br>(0.000)		-2.91e-08<br>(0.000)		5.24e-10<br>(0.000)
BL other donor health spending per capita		0.476***<br>(0.045)		0.180**<br>(0.055)		0.472***<br>(0.050)		0.134*<br>(0.059)		0.400***<br>(0.062)		0.334***<br>(0.048)
BL domestic health spending per capita		0.00687*<br>(0.003)		-0.00487<br>(0.005)		0.00968**<br>(0.003)		0.00411<br>(0.005)		0.0161***<br>(0.005)		0.0108***<br>(0.003)
BL GDP per capita		0.000394***<br>(0.000)		0.000666***<br>(0.000)		0.000415***<br>(0.000)		0.000443***<br>(0.000)		0.0000186<br>(0.000)		0.000589***<br>(0.000)
BL HIV prevalence (% of population ages 15-49)		-0.000776<br>(0.099)		0.354**<br>(0.121)		-1.559**<br>(0.513)		0.115<br>(0.121)		0.206<br>(0.841)		-1.416*<br>(0.574)
BL life expectancy at birth		0.0388<br>(0.096)		0.335*<br>(0.135)		-0.489***<br>(0.133)		0.237<br>(0.125)		-0.539***<br>(0.160)		-0.416*<br>(0.165)
BL Urban population (%)		-0.162***<br>(0.022)		-0.270***<br>(0.029)		-0.166***<br>(0.027)		-0.260***<br>(0.027)		-0.120***<br>(0.032)		-0.320***<br>(0.031)
BL School enrollment, secondary (% gross)		0.000962<br>(0.022)		-0.0445<br>(0.032)		0.0192<br>(0.027)		-0.0273<br>(0.036)		0.198***<br>(0.036)		-0.0312<br>(0.031)
BL Fertility rate (births per woman)		-3.193***<br>(0.460)		-2.913***<br>(0.613)		-5.457***<br>(0.595)		-2.330**<br>(0.769)		-3.805***<br>(0.651)		-8.096***<br>(0.590)
Recipient of US HIV aid before 2004 (=1 if yes)		0.303<br>(0.656)		5.835***<br>(1.088)		-1.239<br>(0.804)		5.876***<br>(1.041)		0.975<br>(1.275)		-1.459<br>(1.087)
Constant	58.82***<br>(2.478)	63.39***<br>(8.043)	60.35***<br>(3.064)	52.43***<br>(10.716)	58.95***<br>(3.007)	105.5***<br>(11.324)	59.55***<br>(3.087)	56.95***<br>(11.082)	60.39***<br>(3.513)	94.79***<br>(13.137)	60.16***<br>(3.493)	119.8***<br>(12.960)
Adjusted R-squared	0.325	0.444	0.290	0.422	0.307	0.464	0.318	0.452	0.261	0.380	0.263	0.546
Observations	2830	2657	1624	1480	1964	1848	1614	1441	1336	1249	1396	1309

Notes: Standard errors in parentheses *** p<0.001, ** p<0.01, * p<0.05 BL=baseline; COP=country operational plans; PEPFAR= President's Emergency Plan for AIDS Relief

For peer review only

Table S5-5. Full model results for the indicator "Number of women who die from pregnancy-related causes while pregnant or within 42 days of pregnancy termination per 100,000 live births"

Variables	All PEPFAR countries		COP countries		Non-COP PEPFAR countries		High-intensity PEPFAR countries		Medium-intensity PEPFAR countries		Low-intensity PEPFAR countries	
	Model 1	Model 2	Model 1	Model 2	Model 1	Model 2	Model 1	Model 2	Model 1	Model 2	Model 1	Model 2
Intervention (=1 if PEPFAR)	355.4*** (26.828)	80.00*** (12.827)	412.6*** (38.080)	133.1*** (26.575)	359.5*** (30.661)	69.38*** (13.947)	486.8*** (38.028)	298.9*** (24.624)	195.9*** (41.087)	53.63* (22.027)	462.5*** (38.059)	141.0*** (19.905)
Treatment (=1 for the years PEPFAR starts and after)	-109.3*** (28.820)	-60.11*** (14.914)	-82.58* (39.845)	-80.42*** (24.385)	-171.6*** (33.208)	-58.73*** (16.511)	-145.1*** (39.857)	-118.6*** (23.209)	19.84 (44.065)	-53.09* (26.557)	-310.2*** (40.523)	-95.90*** (21.931)
Country income level (=1 if middle income)		-11.16 (12.778)		-53.87*** (12.214)		5.238 (14.585)		-52.15*** (12.726)		9.295 (17.031)		-18.82 (14.331)
BL Population		1.56e-08 (0.000)		-4.26e-09 (0.000)		2.25e-08* (0.000)		0.00000154*** (0.000)		-8.65e-08 (0.000)		-2.62e-08** (0.000)
BL other donor health spending per capita		-1.019*** (0.131)		-0.585*** (0.084)		-0.960*** (0.131)		-0.322*** (0.058)		-0.841*** (0.123)		-0.626*** (0.103)
BL domestic health spending per capita		0.234*** (0.019)		0.128*** (0.017)		0.284*** (0.022)		0.0901*** (0.015)		0.271*** (0.028)		0.183*** (0.021)
BL GDP per capita		-0.00644*** (0.001)		-0.00247*** (0.000)		-0.00812*** (0.001)		-0.00150*** (0.000)		-0.00682*** (0.001)		-0.00560*** (0.001)
BL HIV prevalence (% of population ages 15-49)		-18.06*** (1.697)		-8.348*** (1.413)		2.198 (6.563)		-10.47*** (1.106)		-9.512 (8.684)		23.58** (7.206)
BL life expectancy at birth		-27.28*** (1.685)		-16.44*** (1.328)		-27.02*** (2.227)		-16.07*** (1.048)		-29.07*** (3.032)		-14.02*** (1.414)
BL Urban population (%)		-0.0358 (0.183)		-0.202 (0.159)		-0.355 (0.193)		-0.489** (0.155)		-0.127 (0.211)		-0.439** (0.161)
BL School enrollment, secondary (% gross)		-1.400*** (0.200)		-0.832*** (0.192)		-1.919*** (0.201)		-1.160*** (0.176)		-1.400*** (0.205)		-2.094*** (0.216)
BL Fertility rate (births per woman)		23.31*** (4.534)		27.28*** (3.611)		24.11*** (4.863)		12.70** (4.108)		20.92*** (4.679)		36.01*** (4.603)
Recipient of US HIV aid before 2004 (=1 if yes)		-52.71*** (8.684)		-17.72 (11.645)		-37.63*** (9.542)		-141.1*** (10.915)		-25.98 (15.371)		-31.98** (9.902)
Constant	109.0*** (30.611)	2103.9*** (123.188)	121.7*** (24.827)	1314.3*** (90.961)	81.44* (33.768)	2105.6*** (161.891)	103.1*** (24.677)	1343.5*** (76.467)	146.4*** (34.214)	2198.5*** (217.129)	48.75 (32.077)	1181.9*** (98.242)
Adjusted R-squared	0.203	0.776	0.445	0.814	0.171	0.782	0.471	0.862	0.151	0.723	0.249	0.795
Observations	2628	2412	1566	1386	2070	1890	1548	1350	1548	1404	1548	1386

Notes: Standard errors in parentheses *** p<0.001, ** p<0.01, * p<0.05 BL=baseline; COP=country operational plans; PEPFAR= President's Emergency Plan for AIDS Relief

For peer review only

Table S5-6. Full model results for the indicator "Probability of a child dying between birth and 5 years of age, per 1,000 live births"

Variables	All PEPFAR countries		COP countries		Non-COP PEPFAR countries		High-intensity PEPFAR countries		Medium-intensity PEPFAR countries		Low-intensity PEPFAR countries	
	Model 1	Model 2	Model 1	Model 2	Model 1	Model 2	Model 1	Model 2	Model 1	Model 2	Model 1	Model 2
Intervention (=1 if PEPFAR)	60.72***<br>(2.098)	9.613***<br>(1.098)	82.72***<br>(3.063)	15.97***<br>(2.302)	53.72***<br>(2.443)	7.436***<br>(1.148)	88.03***<br>(3.236)	32.41***<br>(3.127)	43.97***<br>(3.147)	4.532***<br>(1.371)	59.82***<br>(3.158)	11.02***<br>(1.654)
Treatment (=1 for the years PEPFAR starts and after)	-22.41***<br>(2.529)	-20.65***<br>(1.098)	-30.81***<br>(3.485)	-32.80***<br>(1.957)	-26.47***<br>(2.909)	-18.44***<br>(1.171)	-36.15***<br>(3.633)	-37.75***<br>(2.159)	-11.73**<br>(3.798)	-19.11***<br>(1.532)	-37.12***<br>(3.634)	-19.56***<br>(1.488)
Country income level (=1 if middle income)		-2.241<br>(1.242)		2.733<br>(1.938)		-5.415***<br>(1.429)		3.657<br>(1.992)		-5.732***<br>(1.531)		-6.059**<br>(2.110)
BL Population		9.64e-09***<br>(0.000)		1.30e-08***<br>(0.000)		1.02e-08***<br>(0.000)		6.95e-08***<br>(0.000)		6.18e-08***<br>(0.000)		6.47e-09***<br>(0.000)
BL other donor health spending per capita		-0.263***<br>(0.019)		-0.210***<br>(0.019)		-0.258***<br>(0.020)		-0.202***<br>(0.019)		-0.239***<br>(0.020)		-0.203***<br>(0.019)
BL domestic health spending per capita		0.0147***<br>(0.002)		0.00330<br>(0.002)		0.0196***<br>(0.002)		-0.00144<br>(0.003)		0.0194***<br>(0.002)		0.0105***<br>(0.002)
BL GDP per capita		-<br>0.000464***<br>(0.000)		0.0000298<br>(0.000)		-<br>0.000578***<br>(0.000)		0.000104<br>(0.000)		-<br>0.000480***<br>(0.000)		-<br>0.000310***<br>(0.000)
BL HIV prevalence (% of population ages 15-49)		-2.397***<br>(0.144)		-1.964***<br>(0.161)		-0.936<br>(0.586)		-2.352***<br>(0.178)		1.191<br>(0.819)		0.737<br>(0.775)
BL life expectancy at birth		-3.953***<br>(0.112)		-3.675***<br>(0.148)		-3.958***<br>(0.141)		-3.775***<br>(0.142)		-4.159***<br>(0.172)		-3.107***<br>(0.160)
BL Urban population (%)		-0.00291<br>(0.023)		0.0506*<br>(0.022)		-0.0748***<br>(0.022)		0.0715**<br>(0.025)		-0.0648**<br>(0.021)		-0.0700**<br>(0.021)
BL School enrollment, secondary (% gross)		-0.219***<br>(0.023)		-0.270***<br>(0.030)		-0.207***<br>(0.023)		-0.255***<br>(0.032)		-0.139***<br>(0.025)		-0.241***<br>(0.026)
BL Fertility rate (births per woman)		7.117***<br>(0.495)		6.249***<br>(0.591)		6.726***<br>(0.536)		5.146***<br>(0.670)		6.211***<br>(0.506)		7.277***<br>(0.649)
Recipient of US HIV aid before 2004 (=1 if yes)		0.939<br>(0.955)		6.445***<br>(1.525)		1.772<br>(0.974)		-3.306<br>(2.242)		3.206**<br>(1.242)		2.400<br>(1.351)
Constant	50.14***<br>(4.776)	334.4***<br>(8.895)	45.67***<br>(4.779)	306.7***<br>(12.055)	49.05***<br>(5.219)	340.5***<br>(10.837)	45.22***<br>(4.967)	314.4***<br>(11.918)	50.38***<br>(5.249)	345.9***<br>(12.926)	45.60***<br>(5.259)	279.5***<br>(11.904)
Adjusted R-squared	0.313	0.855	0.494	0.856	0.264	0.859	0.504	0.850	0.242	0.856	0.293	0.854
Observations	4408	4002	2697	2349	3509	3161	2668	2291	2668	2378	2668	2349

Notes: Standard errors in parentheses *** p<0.001, ** p<0.01, * p<0.05 BL=baseline; COP=country operational plans; PEPFAR= President's Emergency Plan for AIDS Relief

For peer review only

Table S5-7. Full model results for the indicator "Prevalence of anemia among women of reproductive age (% of women ages 15-49)"

Variables	All PEPFAR countries		COP countries		Non-COP PEPFAR countries		High-intensity PEPFAR countries		Medium-intensity PEPFAR countries		Low-intensity PEPFAR countries	
	Model 1	Model 2	Model 1	Model 2	Model 1	Model 2	Model 1	Model 2	Model 1	Model 2	Model 1	Model 2
Intervention (=1 if PEPFAR)	8.473*** (0.467)	-1.293** (0.409)	10.09*** (0.592)	-2.824*** (0.828)	7.934*** (0.541)	-3.064*** (0.415)	9.923*** (0.615)	-1.341 (0.959)	6.922*** (0.716)	-3.062*** (0.494)	8.935*** (0.661)	-3.030*** (0.630)
Treatment (=1 for the years PEPFAR starts and after)	-1.246* (0.630)	-0.422 (0.457)	-1.131 (0.798)	-1.091 (0.707)	-1.990* (0.772)	0.00201 (0.483)	-1.672* (0.836)	-1.715* (0.770)	1.286 (1.031)	-0.547 (0.609)	-4.197*** (0.930)	0.0226 (0.577)
Country income level (=1 if middle income)		-5.748*** (0.537)		-0.474 (0.724)		-7.546*** (0.675)		-1.088 (0.791)		-4.592*** (0.726)		-9.547*** (0.993)
BL Population		3.62e-09*** (0.000)		1.21e-08*** (0.000)		-2.66e-10 (0.000)		-1.16e-08 (0.000)		3.42e-08*** (0.000)		2.27e-09* (0.000)
BL other donor health spending per capita		-0.0717*** (0.003)		-0.0633*** (0.004)		-0.0685*** (0.003)		-0.0628*** (0.004)		-0.0797*** (0.004)		-0.0605*** (0.004)
BL domestic health spending per capita		-0.00835*** (0.001)		-0.00621*** (0.001)		-0.00420*** (0.001)		-0.00712*** (0.001)		0.000541 (0.001)		-0.00470*** (0.001)
BL GDP per capita		0.000396*** (0.000)		0.000380*** (0.000)		0.000315*** (0.000)		0.000416*** (0.000)		0.000224*** (0.000)		0.000315*** (0.000)
BL HIV prevalence (% of population ages 15-49)		-0.673*** (0.042)		-0.571*** (0.050)		0.783*** (0.193)		-0.691*** (0.061)		0.765* (0.386)		0.628** (0.215)
BL life expectancy at birth		-0.584*** (0.034)		-0.475*** (0.047)		-0.452*** (0.038)		-0.543*** (0.051)		-0.598*** (0.046)		-0.248*** (0.052)
BL Urban population (%)		0.0352** (0.011)		-0.0544*** (0.012)		-0.0240* (0.011)		-0.0364** (0.013)		-0.0737*** (0.012)		-0.0698*** (0.013)
BL School enrollment, secondary (% gross)		-0.0532*** (0.010)		-0.128*** (0.013)		-0.0689*** (0.010)		-0.129*** (0.014)		-0.114*** (0.012)		-0.0621*** (0.013)
BL Fertility rate (births per woman)		1.236*** (0.189)		0.333 (0.301)		1.544*** (0.203)		-0.0349 (0.361)		1.806*** (0.221)		1.459*** (0.249)
Recipient of US HIV aid before 2004 (=1 if yes)		-0.714* (0.331)		2.638*** (0.504)		0.360 (0.374)		2.702*** (0.605)		-0.982 (0.511)		1.749** (0.548)
Constant	38.50*** (0.910)	84.97*** (2.990)	38.71*** (1.047)	84.42*** (4.341)	38.47*** (1.049)	79.70*** (3.259)	38.71*** (1.054)	90.28*** (4.815)	39.21*** (1.184)	91.53*** (3.877)	38.19*** (1.151)	69.22*** (4.362)
Adjusted R-squared	0.191	0.620	0.274	0.561	0.166	0.689	0.259	0.540	0.175	0.687	0.184	0.669
Observations	3996	3672	2403	2133	3159	2889	2376	2079	2376	2160	2376	2133

Notes: Standard errors in parentheses *** p<0.001, ** p<0.01, * p<0.05 BL=baseline; COP=country operational plans; PEPFAR= President's Emergency Plan for AIDS Relief

For peer review only

S6. Full model of logged regression of seven outcomes from staggered DID

For peer review only

Table S6-1. Full model results for the indicator "Percent of children ages 12-23 months who received DPT vaccinations (3 doses)"

Variables	All PEPFAR countries		COP countries		Non-COP PEPFAR countries		High-intensity PEPFAR countries		Medium-intensity PEPFAR countries		Low-intensity PEPFAR countries	
	Model 1	Model 2	Model 1	Model 2	Model 1	Model 2	Model 1	Model 2	Model 1	Model 2	Model 1	Model 2
Intervention (=1 if PEPFAR)	-0.233***<br>(0.014)	-0.0906***<br>(0.011)	-0.264***<br>(0.022)	-0.102***<br>(0.024)	-0.230***<br>(0.016)	-0.0833***<br>(0.012)	-0.255***<br>(0.022)	-0.0574*<br>(0.026)	-0.161***<br>(0.019)	-0.0417**<br>(0.014)	-0.301***<br>(0.023)	-0.125***<br>(0.016)
Treatment (=1 for the years PEPFAR starts and after)	0.147***<br>(0.017)	0.135***<br>(0.013)	0.122***<br>(0.026)	0.155***<br>(0.021)	0.190***<br>(0.019)	0.144***<br>(0.013)	0.145***<br>(0.026)	0.134***<br>(0.021)	0.0806***<br>(0.022)	0.141***<br>(0.018)	0.261***<br>(0.026)	0.179***<br>(0.019)
Country income level (=1 if middle income)		-0.0644***<br>(0.015)		-0.0866***<br>(0.020)		-0.0661***<br>(0.014)		-0.0873***<br>(0.022)		-0.0852***<br>(0.016)		-0.0979***<br>(0.018)
Ln(BL Population)		-0.00818***<br>(0.002)		-0.00212<br>(0.003)		-0.0170***<br>(0.002)		0.000520<br>(0.003)		-0.00564*<br>(0.002)		-0.0164***<br>(0.002)
Ln(BL other donor health spending per capita)		0.0165***<br>(0.004)		0.00808<br>(0.005)		0.00247<br>(0.003)		0.00549<br>(0.004)		0.00999**<br>(0.004)		0.00129<br>(0.004)
Ln(BL domestic health spending per capita)		-0.0181<br>(0.012)		0.00146<br>(0.014)		-0.0513***<br>(0.013)		-0.0280*<br>(0.014)		0.00146<br>(0.013)		-0.0578***<br>(0.015)
Ln(BL GDP per capita)		-0.0167<br>(0.016)		-0.0587**<br>(0.022)		-0.0266<br>(0.017)		-0.0516**<br>(0.020)		-0.0318<br>(0.017)		-0.0284<br>(0.022)
Ln(BL HIV prevalence (% of population ages 15-49))		-0.000417<br>(0.001)		-0.00310<br>(0.002)		-0.00461***<br>(0.001)		-0.00488<br>(0.003)		-0.00409**<br>(0.002)		-0.00257<br>(0.002)
Ln(BL diphtheria prevalence (% of population, under 5))		-0.0294***<br>(0.002)		-0.0331***<br>(0.003)		-0.00966***<br>(0.002)		-0.0298***<br>(0.003)		-0.00606**<br>(0.002)		-0.00371<br>(0.002)
Ln(BL tetanus prevalence (% of population, under 5))		0.0149***<br>(0.002)		0.0112***<br>(0.002)		0.0126***<br>(0.002)		0.00505*<br>(0.002)		0.0138***<br>(0.003)		0.0104***<br>(0.002)
Ln(BL pertussis (whooping cough) prevalence (% of population, under 5))		-0.144***<br>(0.008)		-0.134***<br>(0.010)		-0.100***<br>(0.006)		-0.139***<br>(0.010)		-0.105***<br>(0.007)		-0.0825***<br>(0.006)
Ln(BL life expectancy at birth)		0.0155<br>(0.045)		-0.196***<br>(0.049)		0.773***<br>(0.087)		-0.107*<br>(0.050)		0.798***<br>(0.097)		0.922***<br>(0.109)
Ln(BL Urban population (%))		0.00706<br>(0.012)		-0.00672<br>(0.015)		0.126***<br>(0.015)		0.00650<br>(0.014)		0.0804***<br>(0.012)		0.111***<br>(0.017)
Ln(BL School enrollment, secondary (% gross))		0.128***<br>(0.017)		0.0640**<br>(0.023)		0.140***<br>(0.019)		0.0783***<br>(0.022)		0.122***<br>(0.023)		0.155***<br>(0.021)
		-0.166***		-0.256***		-0.104***		-0.229***		-0.0793***		-0.148***

For peer review only

Ln(BL Fertility rate (births per woman))		(0.016)		(0.023)		(0.015)		(0.022)		(0.017)		(0.017)
Recipient of US HIV aid before 2004 (=1 if yes)		0.0113		-0.0608***		0.0271**		-0.0567**		-0.0353**		0.0175
		(0.011)		(0.015)		(0.010)		(0.018)		(0.013)		(0.012)
Constant	4.407***	3.338***	4.438***	4.861***	4.411***	0.403	4.445***	4.353***	4.403***	0.157	4.435***	0.0783
	(0.033)	(0.203)	(0.032)	(0.233)	(0.038)	(0.337)	(0.032)	(0.249)	(0.040)	(0.427)	(0.038)	(0.417)
Adjusted R-squared	0.127	0.515	0.132	0.496	0.122	0.574	0.122	0.486	0.070	0.497	0.145	0.614
Observations	4282	3540	2628	2024	3386	2728	2592	1986	2565	1992	2589	1986

Notes: Standard errors in parentheses *** p<0.001, ** p<0.01, * p<0.05 BL=baseline; COP=country operational plans; PEPFAR= President's Emergency Plan for AIDS Relief

For peer review only

Table S6-2. Full model results for the indicator "Percentage of children age 12-23 months who received hepatitis B vaccines, 3 doses) "

Variables	All PEPFAR countries		COP countries		Non-COP PEPFAR countries		High-intensity PEPFAR countries		Medium-intensity PEPFAR countries		Low-intensity PEPFAR countries	
	Model 1	Model 2	Model 1	Model 2	Model 1	Model 2	Model 1	Model 2	Model 1	Model 2	Model 1	Model 2
Intervention (=1 if PEPFAR)	-0.271***	-0.232***	-0.549***	-0.478***	-0.185**	-0.175**	-0.397**	-0.239	-0.236***	-0.197**	-0.271***	-0.163*
	(0.051)	(0.050)	(0.107)	(0.095)	(0.056)	(0.057)	(0.148)	(0.134)	(0.070)	(0.068)	(0.077)	(0.078)
Treatment (=1 for the years PEPFAR starts and after)	0.173***	0.252***	0.404***	0.446***	0.119*	0.200***	0.313*	0.353*	0.134	0.191**	0.172*	0.255**
	(0.051)	(0.052)	(0.108)	(0.105)	(0.056)	(0.059)	(0.148)	(0.147)	(0.072)	(0.069)	(0.079)	(0.080)
Country income level (=1 if middle income)		0.0404		0.0882*		-0.0382		0.0202		-0.0187		0.0383
		(0.027)		(0.039)		(0.030)		(0.036)		(0.037)		(0.050)
Ln(BL Population)		-0.0186**		-0.0275**		-0.0124		-0.00579		-0.0130		-0.0212*
		(0.007)		(0.009)		(0.007)		(0.009)		(0.008)		(0.009)
Ln(BL other donor health spending per capita)		0.0205*		0.000936		0.0194*		0.0131		0.0120		0.0123
		(0.009)		(0.010)		(0.009)		(0.011)		(0.009)		(0.010)
Ln(BL domestic health spending per capita)		-0.0709**		-0.0679*		-0.102***		-0.0991***		-0.142***		-0.0882*
		(0.022)		(0.028)		(0.029)		(0.028)		(0.031)		(0.035)
Ln(BL GDP per capita)		0.0297		0.00630		0.0547		0.0533		0.0596		0.0291
		(0.031)		(0.052)		(0.033)		(0.048)		(0.038)		(0.044)
Ln(BL HIV prevalence (% of population ages 15-49))		0.0123***		0.0244***		0.00260		0.00930		0.00279		0.0103*
		(0.003)		(0.006)		(0.003)		(0.005)		(0.003)		(0.005)
Ln(BL hepatitis B prevalence (% of population, under 5))		-0.0682***		-0.0597***		-0.0529***		-0.0580***		-0.0590***		-0.0420**
		(0.008)		(0.013)		(0.010)		(0.012)		(0.012)		(0.013)
Ln(BL life expectancy at birth)		0.161		0.0731		0.586***		0.188		0.381		0.917***
		(0.104)		(0.141)		(0.157)		(0.112)		(0.204)		(0.195)
Ln(BL Urban population (%))		0.0144		-0.0394		0.0746*		-0.0548*		0.111**		0.0263
		(0.024)		(0.027)		(0.035)		(0.027)		(0.034)		(0.041)
Ln(BL School enrollment, secondary (% gross))		0.0375		0.0433		0.0554		0.0500		0.142**		0.0801*
		(0.028)		(0.040)		(0.032)		(0.037)		(0.045)		(0.040)
Ln(BL Fertility rate (births per woman))		-0.112**		-0.168**		-0.0944*		-0.216***		-0.0911		-0.120**
		(0.035)		(0.051)		(0.037)		(0.053)		(0.050)		(0.043)
Recipient of US HIV aid before 2004 (=1 if yes)		-0.0354		0.0234		-0.0478		-0.0548		-0.000819		-0.127**
		(0.023)		(0.037)		(0.027)		(0.036)		(0.035)		(0.047)
Constant	3.814***	3.186***	3.860***	4.216***	3.923***	1.139	3.923***	3.353***	3.808***	1.553	3.923***	0.128
	(0.327)	(0.613)	(0.318)	(0.799)	(0.373)	(0.787)	(0.374)	(0.756)	(0.330)	(0.998)	(0.374)	(0.890)
Adjusted R-squared	0.149	0.236	0.192	0.277	0.135	0.214	0.154	0.245	0.167	0.246	0.140	0.251
Observations	2799	2304	1789	1379	2270	1809	1744	1341	1809	1399	1766	1332

Notes: Standard errors in parentheses *** p<0.001, ** p<0.01, * p<0.05 BL=baseline; COP=country operational plans; PEPFAR= President's Emergency Plan for AIDS Relief

For peer review only

Table S6-3. Full model results for the indicator "Percent of children ages 12-23 months who received the measles vaccination"

Variables	All PEPFAR countries		COP countries		Non-COP PEPFAR countries		High-intensity PEPFAR countries		Medium-intensity PEPFAR countries		Low-intensity PEPFAR countries	
	Model 1	Model 2	Model 1	Model 2	Model 1	Model 2	Model 1	Model 2	Model 1	Model 2	Model 1	Model 2
Intervention (=1 if PEPFAR)	-0.214***	-0.117***	-0.237***	-0.161***	-0.212***	-0.0908***	-0.223***	-0.101***	-0.159***	-0.0873***	-0.270***	-0.0914***
	(0.013)	(0.012)	(0.020)	(0.024)	(0.015)	(0.012)	(0.019)	(0.023)	(0.018)	(0.017)	(0.021)	(0.014)
Treatment (=1 for the years PEPFAR starts and after)	0.119***	0.104***	0.0970***	0.111***	0.151***	0.104***	0.0972***	0.0778***	0.0629**	0.109***	0.224***	0.135***
	(0.015)	(0.013)	(0.023)	(0.020)	(0.017)	(0.013)	(0.022)	(0.020)	(0.021)	(0.019)	(0.023)	(0.017)
Country income level (=1 if middle income)		-0.0239		-0.0130		-0.0490***		-0.00184		-0.0850***		-0.0407*
		(0.013)		(0.017)		(0.013)		(0.018)		(0.017)		(0.017)
Ln(BL Population)		-0.00723**		-0.0131***		-0.00989***		-0.00986***		-0.00599*		-0.0141***
		(0.002)		(0.003)		(0.002)		(0.003)		(0.003)		(0.002)
Ln(BL other donor health spending per capita)		0.0156***		0.00277		0.00354		0.00256		0.00750*		0.00116
		(0.003)		(0.004)		(0.003)		(0.004)		(0.003)		(0.003)
Ln(BL domestic health spending per capita)		0.0188		0.0311*		-0.0145		0.00652		0.0153		-0.0205
		(0.012)		(0.014)		(0.013)		(0.014)		(0.014)		(0.014)
Ln(BL GDP per capita)		-0.0144		-0.0260		-0.0217		-0.0254		-0.00971		-0.00611
		(0.013)		(0.019)		(0.014)		(0.017)		(0.014)		(0.017)
Ln(BL HIV prevalence (% of population ages 15-49))		0.0115***		0.0147***		0.00345**		0.0127***		0.00464***		0.00548***
		(0.001)		(0.002)		(0.001)		(0.002)		(0.001)		(0.001)
Ln(BL measles prevalence (% of population, under 5))		-0.00107		0.0000214		0.00118		-0.00134		0.00156		0.00442***
		(0.001)		(0.001)		(0.001)		(0.001)		(0.001)		(0.001)
Ln(BL life expectancy at birth)		0.239***		0.0412		0.828***		0.122*		0.783***		0.940***
		(0.047)		(0.056)		(0.078)		(0.050)		(0.092)		(0.091)
Ln(BL Urban population (%))		-0.0360**		-0.0698***		0.0702***		-0.0518***		0.0340**		0.0418**
		(0.012)		(0.013)		(0.013)		(0.011)		(0.013)		(0.014)
Ln(BL School enrollment, secondary (% gross))		0.117***		0.0396*		0.136***		0.0638***		0.129***		0.131***
		(0.014)		(0.020)		(0.015)		(0.018)		(0.017)		(0.017)
Ln(BL Fertility rate (births per woman))		-0.217***		-0.287***		-0.109***		-0.246***		-0.0981***		-0.136***
		(0.016)		(0.024)		(0.016)		(0.023)		(0.020)		(0.017)
Recipient of US HIV aid before 2004 (=1 if yes)		0.0455***		0.0209		0.0463***		0.0161		0.0145		0.0317**
		(0.010)		(0.015)		(0.010)		(0.015)		(0.014)		(0.011)
Constant	4.384***	3.409***	4.390***	4.919***	4.392***	0.601	4.400***	4.421***	4.382***	0.661	4.394***	0.267
	(0.028)	(0.201)	(0.028)	(0.243)	(0.031)	(0.314)	(0.029)	(0.221)	(0.030)	(0.412)	(0.034)	(0.362)
Adjusted R-squared	0.140	0.452	0.156	0.392	0.129	0.558	0.143	0.375	0.093	0.450	0.153	0.615
Observations	4275	3540	2621	2024	3379	2728	2585	1986	2558	1992	2582	1986

Notes: Standard errors in parentheses *** p<0.001, ** p<0.01, * p<0.05 BL=baseline; COP=country operational plans; PEPFAR= President's Emergency Plan for AIDS Relief

For peer review only

Table S6-4. Full model results for the indicator "Percentage of births by women of child-bearing age who are immunized against tetanus"

Variables	All PEPFAR countries		COP countries		Non-COP PEPFAR countries		High-intensity PEPFAR countries		Medium-intensity PEPFAR countries		Low-intensity PEPFAR countries	
	Model 1	Model 2	Model 1	Model 2	Model 1	Model 2	Model 1	Model 2	Model 1	Model 2	Model 1	Model 2
Intervention (=1 if PEPFAR)	-0.102***<br>(0.021)	-0.0190<br>(0.022)	-0.114***<br>(0.030)	-0.117**<br>(0.037)	-0.104***<br>(0.023)	0.000771<br>(0.024)	-0.126***<br>(0.029)	-0.103*<br>(0.040)	-0.0804**<br>(0.031)	-0.0426<br>(0.031)	-0.115***<br>(0.027)	0.0756**<br>(0.027)
Treatment (=1 for the years PEPFAR starts and after)	0.0947***<br>(0.019)	0.0697***<br>(0.019)	0.107***<br>(0.030)	0.0813**<br>(0.030)	0.103***<br>(0.022)	0.0729***<br>(0.022)	0.125***<br>(0.029)	0.0993**<br>(0.031)	0.0874**<br>(0.032)	0.0947**<br>(0.031)	0.104***<br>(0.027)	0.0190<br>(0.024)
Country income level (=1 if middle income)		-0.00576<br>(0.019)		0.00662<br>(0.024)		-0.0453<br>(0.027)		0.0386<br>(0.025)		-0.00149<br>(0.033)		-0.0993**<br>(0.031)
Ln(BL Population)		-0.0234***<br>(0.005)		-0.0267***<br>(0.006)		-0.0235***<br>(0.006)		-0.0297***<br>(0.007)		-0.0351***<br>(0.007)		-0.0174**<br>(0.006)
Ln(BL other donor health spending per capita)		0.00299<br>(0.006)		-0.00627<br>(0.007)		0.00661<br>(0.007)		-0.000983<br>(0.008)		-0.00484<br>(0.008)		0.0176*<br>(0.007)
Ln(BL domestic health spending per capita)		-0.0432**<br>(0.015)		-0.0882***<br>(0.022)		-0.0264<br>(0.020)		-0.0229<br>(0.027)		-0.0154<br>(0.024)		-0.0821***<br>(0.022)
Ln(BL GDP per capita)		0.0579***<br>(0.017)		0.137***<br>(0.024)		0.0581***<br>(0.017)		0.0651*<br>(0.028)		-0.000499<br>(0.021)		0.199***<br>(0.022)
Ln(BL HIV prevalence (% of population ages 15-49))		-0.000730<br>(0.002)		-0.00498<br>(0.003)		0.00161<br>(0.003)		0.00265<br>(0.004)		0.000212<br>(0.003)		-0.000435<br>(0.003)
Ln(BL life expectancy at birth)		-0.0252<br>(0.070)		-0.144<br>(0.106)		-0.0140<br>(0.116)		0.0601<br>(0.090)		-0.379**<br>(0.129)		0.132<br>(0.150)
Ln(BL Urban population (%))		-0.0248<br>(0.016)		-0.116***<br>(0.021)		-0.00817<br>(0.024)		-0.107***<br>(0.019)		0.0486*<br>(0.025)		-0.166***<br>(0.026)
Ln(BL School enrollment, secondary (% gross))		-0.0284<br>(0.017)		-0.0884***<br>(0.024)		-0.00440<br>(0.025)		-0.0815**<br>(0.026)		-0.00713<br>(0.040)		-0.0258<br>(0.030)
Ln(BL Fertility rate (births per woman))		-0.264***<br>(0.031)		-0.313***<br>(0.044)		-0.293***<br>(0.036)		-0.203***<br>(0.049)		-0.343***<br>(0.042)		-0.400***<br>(0.041)
Recipient of US HIV aid before 2004 (=1 if yes)		0.0499**<br>(0.016)		0.177***<br>(0.027)		0.00608<br>(0.017)		0.124***<br>(0.026)		0.0331<br>(0.024)		0.0544*<br>(0.022)
Constant	3.946***<br>(0.062)	4.614***<br>(0.358)	3.985***<br>(0.082)	5.371***<br>(0.530)	3.937***<br>(0.077)	4.368***<br>(0.506)	3.965***<br>(0.083)	4.615***<br>(0.475)	3.979***<br>(0.092)	6.420***<br>(0.591)	3.955***<br>(0.093)	3.558***<br>(0.622)
Adjusted R-squared	0.284	0.359	0.250	0.334	0.270	0.376	0.273	0.345	0.226	0.313	0.232	0.434
Observations	2830	2657	1624	1480	1964	1848	1614	1441	1336	1249	1396	1309

Notes: Standard errors in parentheses *** p<0.001, ** p<0.01, * p<0.05 BL=baseline; COP=country operational plans; PEPFAR= President's Emergency Plan for AIDS Relief

For peer review only

Table S6-5. Full model results for the indicator "Number of women who die from pregnancy-related causes while pregnant or within 42 days of pregnancy termination per 100,000 live births"

Variables	All PEPFAR countries		COP countries		Non-COP PEPFAR countries		High-intensity PEPFAR countries		Medium-intensity PEPFAR countries		Low-intensity PEPFAR countries	
	Model 1	Model 2	Model 1	Model 2	Model 1	Model 2	Model 1	Model 2	Model 1	Model 2	Model 1	Model 2
Intervention (=1 if PEPFAR)	1.503***<br>(0.089)	0.113**<br>(0.040)	1.984***<br>(0.126)	0.424***<br>(0.079)	1.439***<br>(0.099)	0.0248<br>(0.042)	2.163***<br>(0.121)	0.309***<br>(0.071)	0.862***<br>(0.123)	-0.0240<br>(0.048)	1.822***<br>(0.118)	0.115<br>(0.062)
Treatment (=1 for the years PEPFAR starts and after)	-0.0406<br>(0.094)	0.0594<br>(0.040)	0.0448<br>(0.130)	0.0214<br>(0.061)	-0.347**<br>(0.110)	0.0696<br>(0.046)	-0.0987<br>(0.126)	0.00106<br>(0.057)	0.383**<br>(0.139)	-0.0257<br>(0.057)	-0.881***<br>(0.134)	0.0251<br>(0.064)
Country income level (=1 if middle income)		-0.186***<br>(0.039)		-0.174***<br>(0.051)		-0.0830<br>(0.045)		-0.295***<br>(0.046)		-0.0140<br>(0.048)		-0.140*<br>(0.062)
Ln(BL Population)		-0.00770<br>(0.010)		-0.0186<br>(0.012)		-0.0440***<br>(0.010)		-0.0513***<br>(0.012)		-0.0938***<br>(0.010)		-0.0403**<br>(0.013)
Ln(BL other donor health spending per capita)		-0.0204<br>(0.014)		-0.0162<br>(0.017)		-0.0393**<br>(0.015)		-0.0275<br>(0.018)		-0.0893***<br>(0.014)		-0.0351*<br>(0.018)
Ln(BL domestic health spending per capita)		-0.202***<br>(0.028)		-0.149***<br>(0.041)		-0.242***<br>(0.034)		-0.0176<br>(0.045)		-0.307***<br>(0.034)		-0.316***<br>(0.046)
Ln(BL GDP per capita)		0.0988***<br>(0.029)		0.0176<br>(0.046)		0.0560<br>(0.031)		-0.0985*<br>(0.044)		0.0430<br>(0.035)		0.0284<br>(0.039)
Ln(BL HIV prevalence (% of population ages 15-49))		0.0385***<br>(0.004)		0.0153*<br>(0.006)		0.0470***<br>(0.004)		0.0331***<br>(0.006)		0.0366***<br>(0.005)		0.0338***<br>(0.005)
Ln(BL life expectancy at birth)		-2.337***<br>(0.101)		-1.985***<br>(0.151)		-2.156***<br>(0.192)		-1.969***<br>(0.121)		-2.667***<br>(0.208)		-0.839**<br>(0.257)
Ln(BL Urban population (%))		0.0611*<br>(0.028)		0.0171<br>(0.034)		0.124**<br>(0.040)		0.0255<br>(0.033)		0.134**<br>(0.041)		0.129*<br>(0.051)
Ln(BL School enrollment, secondary (% gross))		-0.237***<br>(0.043)		-0.141**<br>(0.050)		-0.507***<br>(0.053)		-0.141**<br>(0.049)		-0.983***<br>(0.066)		-0.463***<br>(0.056)
Ln(BL Fertility rate (births per woman))		1.035***<br>(0.054)		0.855***<br>(0.093)		0.952***<br>(0.058)		1.007***<br>(0.093)		0.491***<br>(0.068)		1.100***<br>(0.079)
Recipient of US HIV aid before 2004 (=1 if yes)		-0.0854**<br>(0.033)		-0.0120<br>(0.044)		-0.0328<br>(0.038)		-0.0616<br>(0.043)		0.0356<br>(0.041)		0.120<br>(0.063)
Constant	4.049***<br>(0.110)	14.84***<br>(0.514)	4.073***<br>(0.123)	13.74***<br>(0.833)	3.940***<br>(0.121)	16.16***<br>(0.793)	4.024***<br>(0.122)	14.42***<br>(0.712)	4.176***<br>(0.131)	21.91***<br>(0.951)	3.846***<br>(0.133)	10.80***<br>(1.010)
Adjusted R-squared	0.265	0.829	0.478	0.827	0.192	0.823	0.492	0.842	0.185	0.815	0.201	0.790
Observations	2628	2178	1566	1206	2070	1674	1548	1188	1548	1206	1548	1188

Notes: Standard errors in parentheses *** p<0.001, ** p<0.01, * p<0.05 BL=baseline; COP=country operational plans; PEPFAR=President's Emergency Plan for AIDS Relief

For peer review only

For peer review only - http://bmjopen.bmj.com/site/about/guidelines.xhtml

f

Table S6-6. Full model results for the indicator "Probability of a child dying between birth and 5 years of age, per 1,000 live births"

Variables	All PEPFAR countries		COP countries		Non-COP PEPFAR countries		High-intensity PEPFAR countries		Medium-intensity PEPFAR countries		Low-intensity PEPFAR countries	
	Model 1	Model 2	Model 1	Model 2	Model 1	Model 2	Model 1	Model 2	Model 1	Model 2	Model 1	Model 2
Intervention (=1 if PEPFAR)	1.001***<br>(0.032)	0.141***<br>(0.020)	1.294***<br>(0.038)	0.182***<br>(0.041)	0.892***<br>(0.036)	0.0607**<br>(0.020)	1.337***<br>(0.038)	0.0846*<br>(0.040)	0.734***<br>(0.046)	0.0357<br>(0.022)	1.008***<br>(0.044)	-0.0136<br>(0.026)
Treatment (=1 for the years PEPFAR starts and after)	-0.0147<br>(0.043)	-0.0521**<br>(0.019)	0.0326<br>(0.049)	-0.0394<br>(0.027)	-0.135**<br>(0.050)	-0.0758***<br>(0.021)	-0.00670<br>(0.049)	-0.0620*<br>(0.028)	0.103<br>(0.065)	-0.0907***<br>(0.024)	-0.306***<br>(0.061)	-0.0750**<br>(0.025)
Country income level (=1 if middle income)		-0.207***<br>(0.018)		-0.0972**<br>(0.031)		-0.243***<br>(0.023)		-0.0972**<br>(0.030)		-0.141***<br>(0.027)		-0.328***<br>(0.034)
Ln(BL Population)		0.0167***<br>(0.004)		0.0165**<br>(0.006)		0.0163**<br>(0.005)		0.000543<br>(0.006)		-0.00483<br>(0.006)		0.0140*<br>(0.006)
Ln(BL other donor health spending per capita)		-0.0174**<br>(0.006)		0.000586<br>(0.008)		-0.0213**<br>(0.007)		-0.0148<br>(0.008)		-0.0365***<br>(0.007)		-0.0119<br>(0.008)
Ln(BL domestic health spending per capita)		-0.117***<br>(0.013)		-0.139***<br>(0.025)		-0.0729***<br>(0.017)		-0.0886**<br>(0.027)		-0.117***<br>(0.021)		-0.0843***<br>(0.022)
Ln(BL GDP per capita)		-0.0138<br>(0.019)		0.0263<br>(0.036)		-0.0468*<br>(0.020)		-0.0571<br>(0.035)		-0.0124<br>(0.022)		-0.0490<br>(0.028)
Ln(BL HIV prevalence (% of population ages 15-49))		-0.0205***<br>(0.002)		-0.0314***<br>(0.004)		-0.0176***<br>(0.003)		-0.0229***<br>(0.004)		-0.0231***<br>(0.003)		-0.0190***<br>(0.003)
Ln(BL life expectancy at birth)		-2.378***<br>(0.068)		-2.436***<br>(0.106)		-3.072***<br>(0.109)		-2.527***<br>(0.096)		-3.698***<br>(0.131)		-2.968***<br>(0.181)
Ln(BL Urban population (%))		0.198***<br>(0.015)		0.204***<br>(0.020)		0.152***<br>(0.021)		0.238***<br>(0.021)		0.152***<br>(0.020)		0.193***<br>(0.026)
Ln(BL School enrollment, secondary (% gross))		-0.139***<br>(0.018)		-0.212***<br>(0.030)		-0.129***<br>(0.022)		-0.187***<br>(0.029)		-0.314***<br>(0.032)		-0.141***<br>(0.027)
Ln(BL Fertility rate (births per woman))		0.480***<br>(0.025)		0.367***<br>(0.047)		0.420***<br>(0.027)		0.450***<br>(0.048)		0.192***<br>(0.032)		0.451***<br>(0.039)
Recipient of US HIV aid before 2004 (=1 if yes)		0.0506***<br>(0.013)		0.103***<br>(0.023)		0.136***<br>(0.014)		0.139***<br>(0.026)		0.177***<br>(0.018)		0.280***<br>(0.027)
Constant	3.534***<br>(0.060)	13.83***<br>(0.329)	3.522***<br>(0.069)	14.08***<br>(0.539)	3.541***<br>(0.068)	17.10***<br>(0.449)	3.518***<br>(0.070)	14.87***<br>(0.515)	3.591***<br>(0.077)	20.93***<br>(0.546)	3.518***<br>(0.074)	16.70***<br>(0.723)
Adjusted R-squared	0.364	0.874	0.504	0.837	0.303	0.868	0.509	0.839	0.276	0.844	0.309	0.834
Observations	4408	3596	2697	2030	3509	2784	2668	2001	2668	2030	2668	2001

Notes: Standard errors in parentheses *** p<0.001, ** p<0.01, * p<0.05 BL=baseline; COP=country operational plans; PEPFAR= President's Emergency Plan for AIDS Relief

For peer review only

Table S6-7. Full model results for the indicator "Prevalence of anemia among women of reproductive age (% of women ages 15-49)"

Variables	All PEPFAR countries		COP countries		Non-COP PEPFAR countries		High-intensity PEPFAR countries		Medium-intensity PEPFAR countries		Low-intensity PEPFAR countries	
	**Model 1**	**Model 2**	**Model 1**	**Model 2**	**Model 1**	**Model 2**	**Model 1**	**Model 2**	**Model 1**	**Model 2**	**Model 1**	**Model 2**
Intervention (=1 if PEPFAR)	0.231***	0.0319*	0.283***	0.102***	0.211***	-0.0194	0.278***	0.0839**	0.177***	-0.0276	0.243***	-0.0258
	(0.013)	(0.014)	(0.016)	(0.028)	(0.015)	(0.014)	(0.016)	(0.030)	(0.019)	(0.016)	(0.018)	(0.020)
Treatment (=1 for the years PEPFAR starts and after)	-0.0189	0.0130	-0.000761	0.0138	-0.0484*	0.0200	-0.0211	-0.00408	0.0506	0.00721	-0.103***	0.0145
	(0.018)	(0.015)	(0.022)	(0.022)	(0.022)	(0.016)	(0.023)	(0.023)	(0.029)	(0.021)	(0.027)	(0.019)
Country income level (=1 if middle income)		-0.258***		-0.202***		-0.233***		-0.242***		-0.124***		-0.264***
		(0.019)		(0.026)		(0.024)		(0.026)		(0.027)		(0.032)
BL Population		-0.000988		-0.00222		0.00352		-0.00821*		-0.0144***		0.00994**
		(0.003)		(0.004)		(0.003)		(0.004)		(0.004)		(0.004)
BL other donor health spending per capita		-0.0318***		-0.0244***		-0.0337***		-0.0216***		-0.0662***		-0.0288***
		(0.004)		(0.005)		(0.004)		(0.005)		(0.005)		(0.005)
BL domestic health spending per capita		-0.0960***		-0.0535***		-0.0603***		-0.0166		-0.0231		-0.0761***
		(0.011)		(0.015)		(0.014)		(0.016)		(0.016)		(0.015)
BL GDP per capita		0.143***		0.181***		0.129***		0.171***		0.0557***		0.156***
		(0.012)		(0.015)		(0.011)		(0.015)		(0.015)		(0.011)
BL HIV prevalence (% of population ages 15-49)		-0.0198***		-0.0357***		-0.0141***		-0.0335***		-0.0229***		-0.0169***
		(0.002)		(0.002)		(0.002)		(0.003)		(0.002)		(0.002)
BL life expectancy at birth		-0.583***		-0.237***		-1.235***		-0.283***		-1.370***		-0.961***
		(0.047)		(0.065)		(0.075)		(0.061)		(0.102)		(0.084)
BL Urban population (%)		0.0726***		-0.0211		-0.0470**		-0.0151		-0.0875***		-0.111***
		(0.013)		(0.018)		(0.017)		(0.020)		(0.020)		(0.020)
BL School enrollment, secondary (% gross)		-0.0675***		-0.148***		-0.0664***		-0.173***		-0.302***		-0.0471*
		(0.014)		(0.024)		(0.016)		(0.023)		(0.024)		(0.021)
BL Fertility rate (births per woman)		0.244***		0.222***		0.169***		0.230***		0.0631*		0.206***
		(0.019)		(0.033)		(0.021)		(0.033)		(0.025)		(0.028)
Recipient of US HIV aid before 2004 (=1 if yes)		-0.0180		0.136***		-0.00555		0.121***		0.0361*		0.0327
		(0.012)		(0.018)		(0.013)		(0.021)		(0.018)		(0.021)
Constant	3.602***	5.334***	3.612***	3.911***	3.605***	8.508***	3.613***	4.190***	3.629***	10.94***	3.601***	7.234***
	(0.022)	(0.226)	(0.026)	(0.336)	(0.026)	(0.319)	(0.026)	(0.331)	(0.029)	(0.436)	(0.029)	(0.349)
Adjusted R-squared	0.188	0.554	0.270	0.482	0.160	0.621	0.253	0.475	0.166	0.613	0.172	0.592
Observations	3996	3321	2403	1863	3159	2565	2376	1836	2376	1863	2376	1836

Notes: Standard errors in parentheses *** p<0.001, ** p<0.01, * p<0.05 BL=baseline; COP=country operational plans; PEPFAR= President’s Emergency Plan for AIDS Relief

For peer review only

S7. Parallel pre-trend assumption test for traditional DID

S7-1. DPT immunization rate

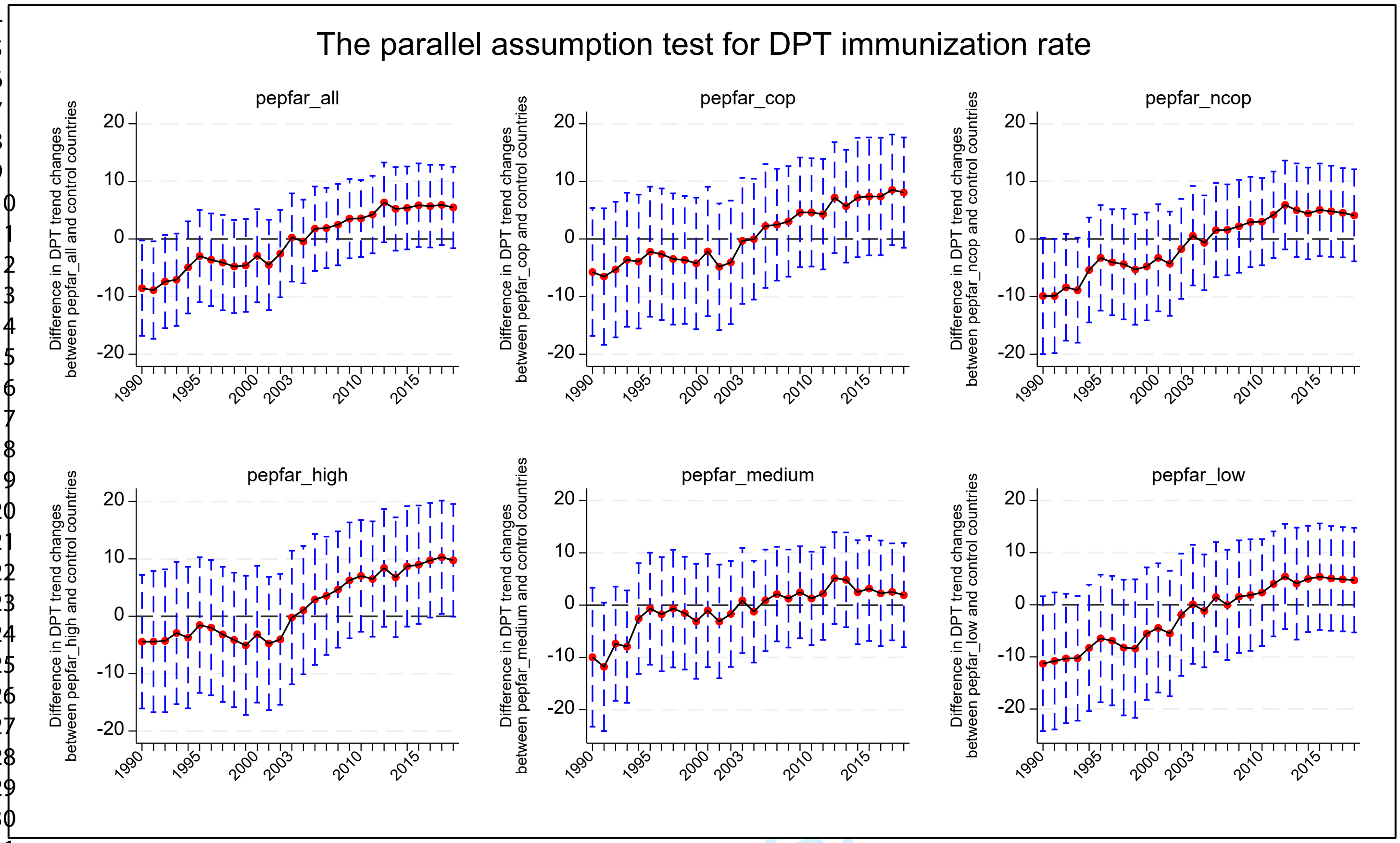

Notes: pepfar_all = all PEPFAR country cohort; pepfar_cop = COP PEPFAR country cohort; pepfar_ncop = non-COP PEPFAR country cohort; pepfar_high = high-intensity PEPFAR country cohort; pepfar_medium = medium-intensity PEPFAR country cohort; pepfar_low = low-intensity PEPFAR country cohort. COP= country operational plans; DPT=diphtheria, pertussis, tetanus; PEPFAR= President's Emergency Plan for AIDS Relief

S7-2. Hepatitis B immunization rate

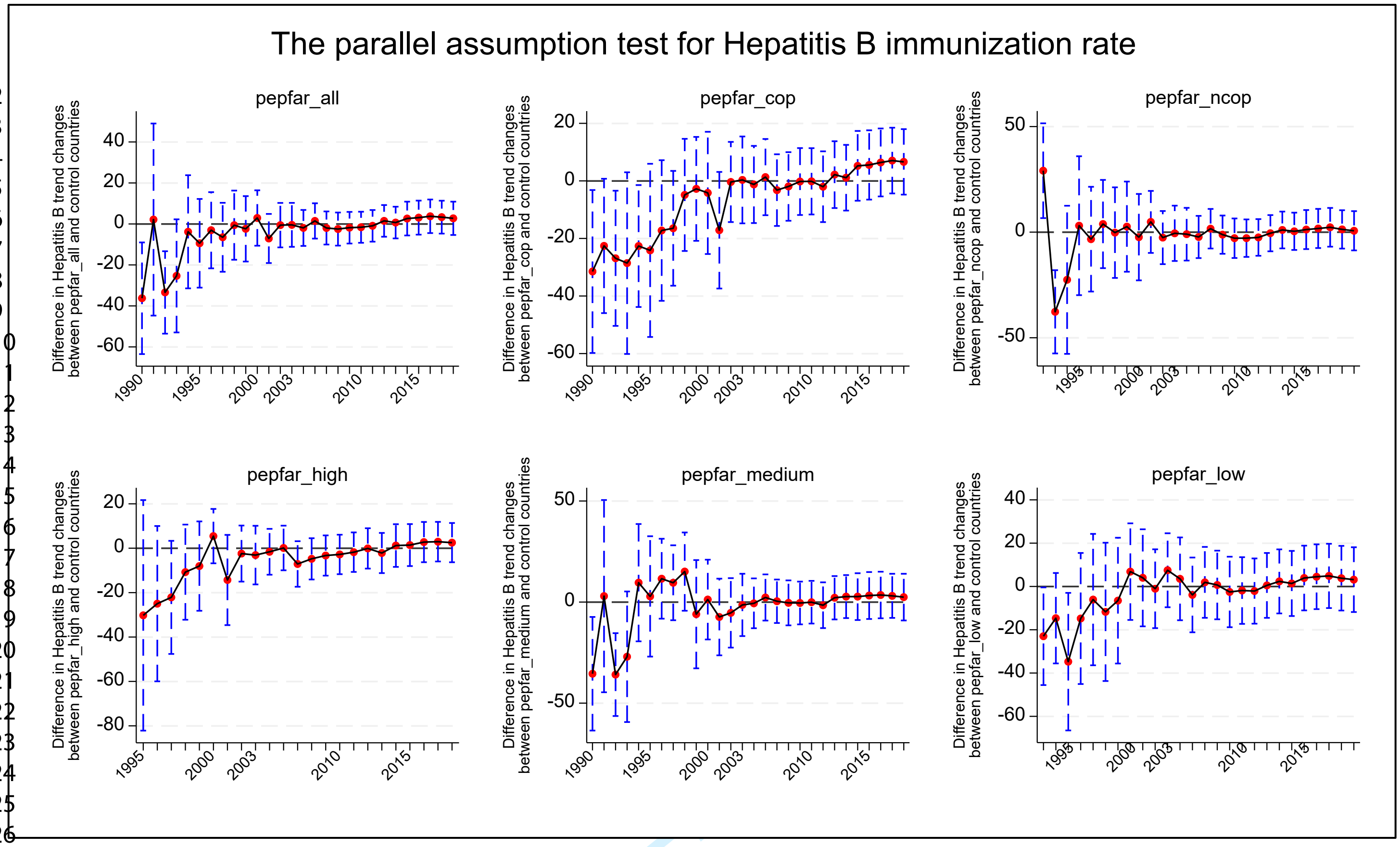

Notes: pepfar_all = all PEPFAR country cohort; pepfar_cop = COP PEPFAR country cohort; pepfar_ncop = non-COP PEPFAR country cohort; pepfar_high = high-intensity PEPFAR country cohort; pepfar_medium = medium-intensity PEPFAR country cohort; pepfar_low = low-intensity PEPFAR country cohort. COP= country operational plans; PEPFAR= President's Emergency Plan for AIDS Relief

S7-3. Measles immunization rate

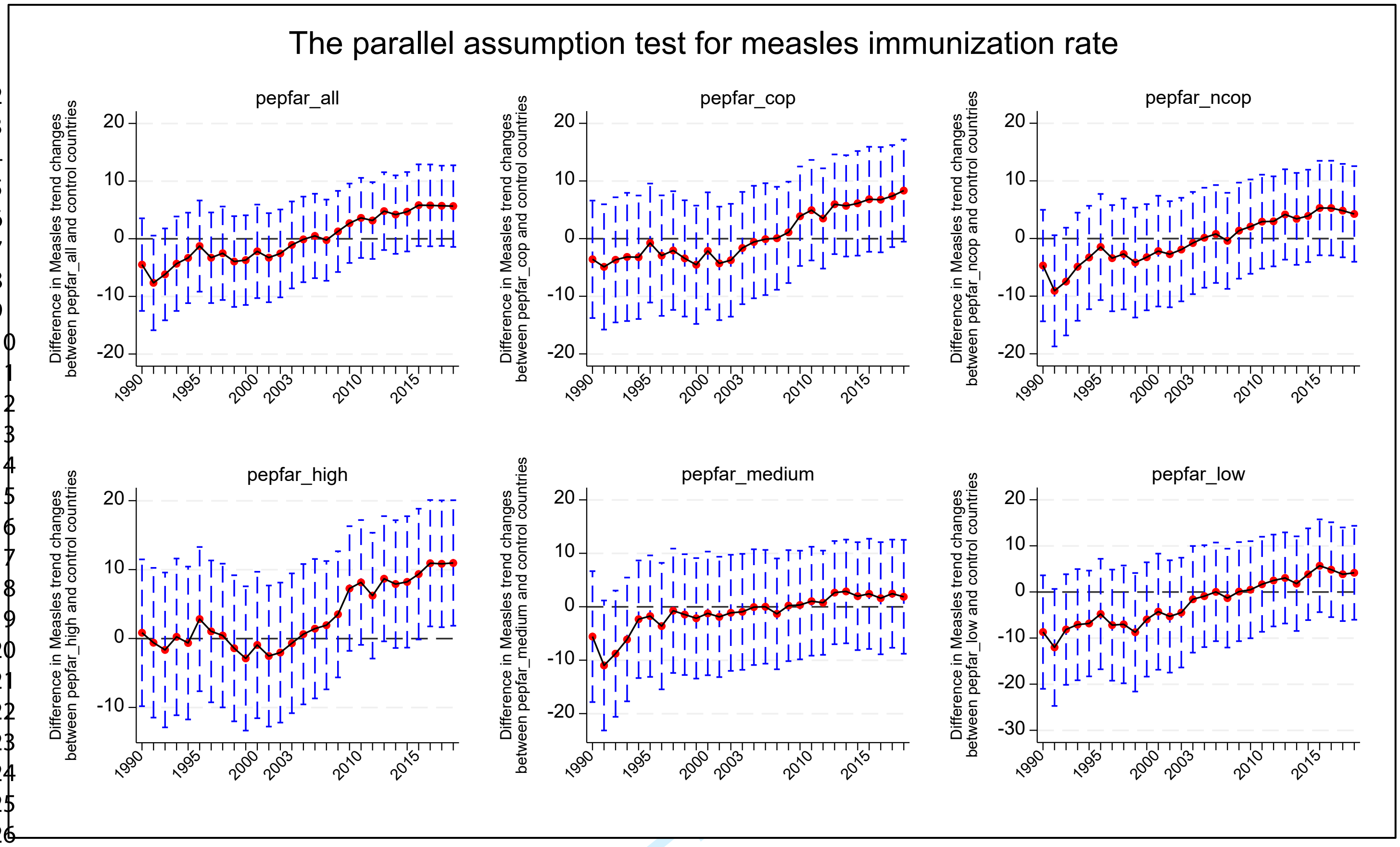

Notes: pepfar_all = all PEPFAR country cohort; pepfar_cop = COP PEPFAR country cohort; pepfar_ncop = non-COP PEPFAR country cohort; pepfar_high = high-intensity PEPFAR country cohort; pepfar_medium = medium-intensity PEPFAR country cohort; pepfar_low = low-intensity PEPFAR country cohort. COP= country operational plans; PEPFAR= President's Emergency Plan for AIDS Relief

S7-4. Prevalence of newborns protected against tetanus

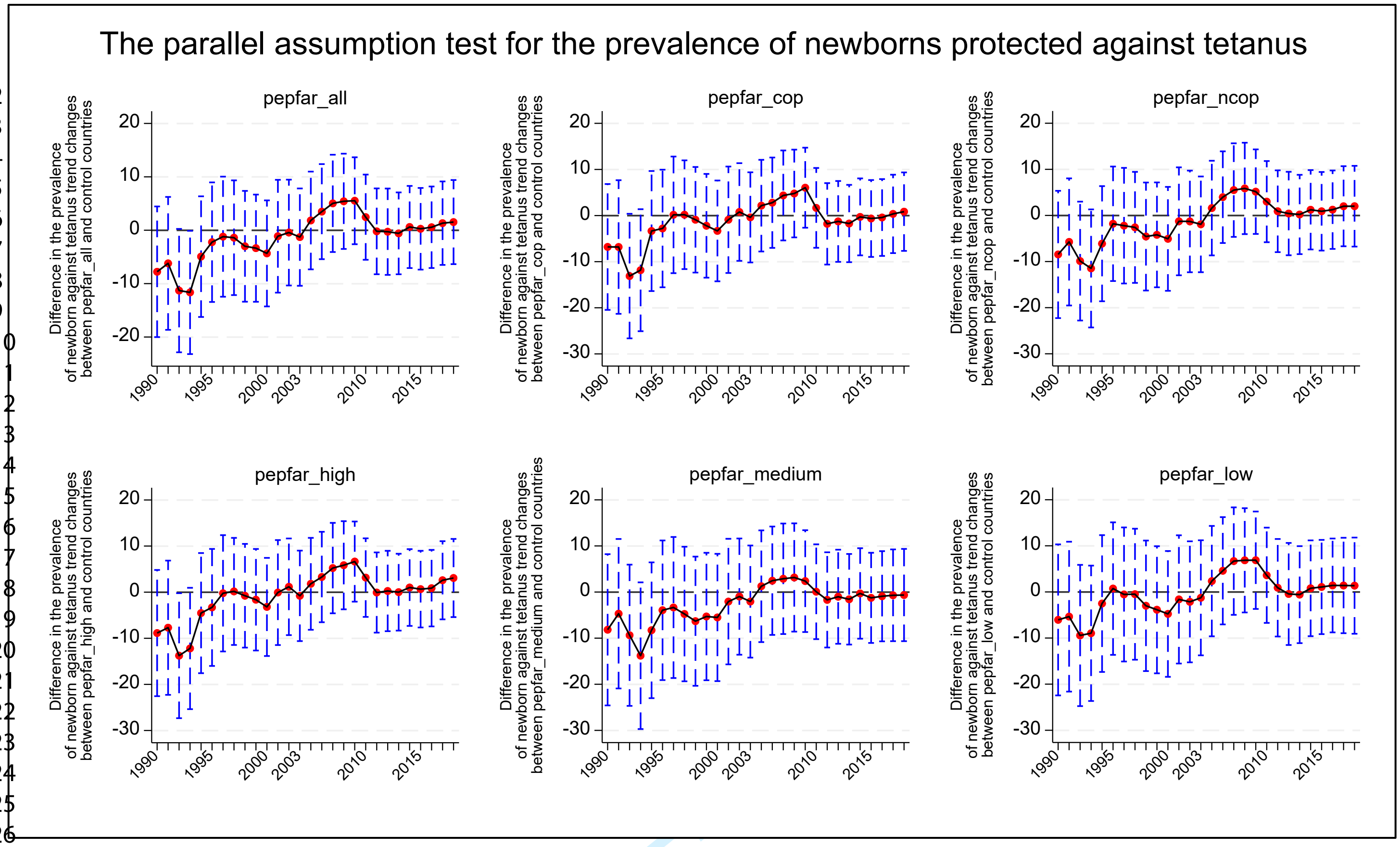

Notes: pepfar_all = all PEPFAR country cohort; pepfar_cop = COP PEPFAR country cohort; pepfar_ncop = non-COP PEPFAR country cohort; pepfar_high = high-intensity PEPFAR country cohort; pepfar_medium = medium-intensity PEPFAR country cohort; pepfar_low = low-intensity PEPFAR country cohort. COP= country operational plans; PEPFAR= President's Emergency Plan for AIDS Relief

For peer review only

S7-5. Maternal mortality ratio

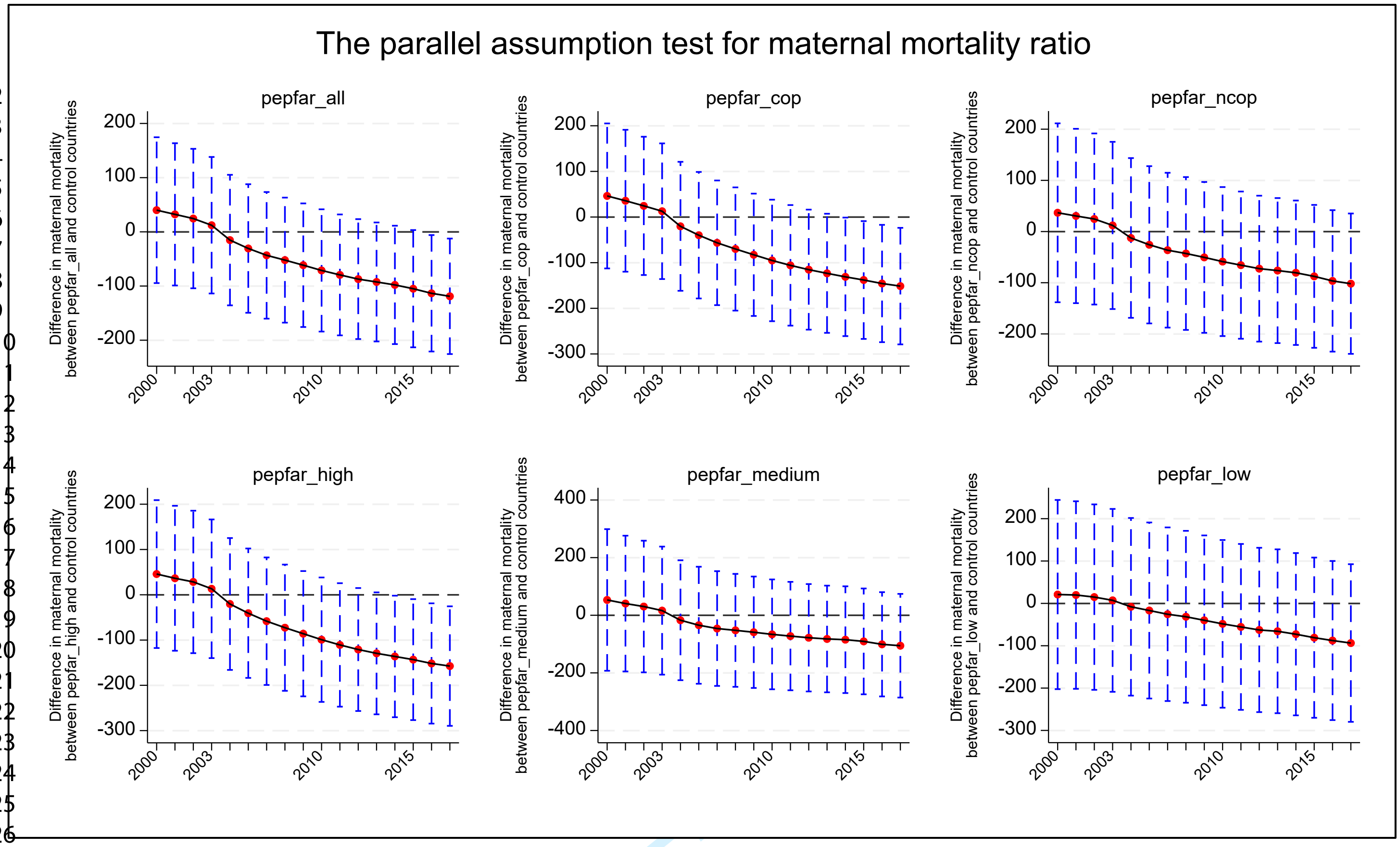

Notes: pepfar_all = all PEPFAR country cohort; pepfar_cop = COP PEPFAR country cohort; pepfar_ncop = non-COP PEPFAR country cohort; pepfar_high = high-intensity PEPFAR country cohort; pepfar_medium = medium-intensity PEPFAR country cohort; pepfar_low = low-intensity PEPFAR country cohort. COP= country operational plans; PEPFAR= President's Emergency Plan for AIDS Relief

For peer review only

S7-6. Child under-five mortality rate

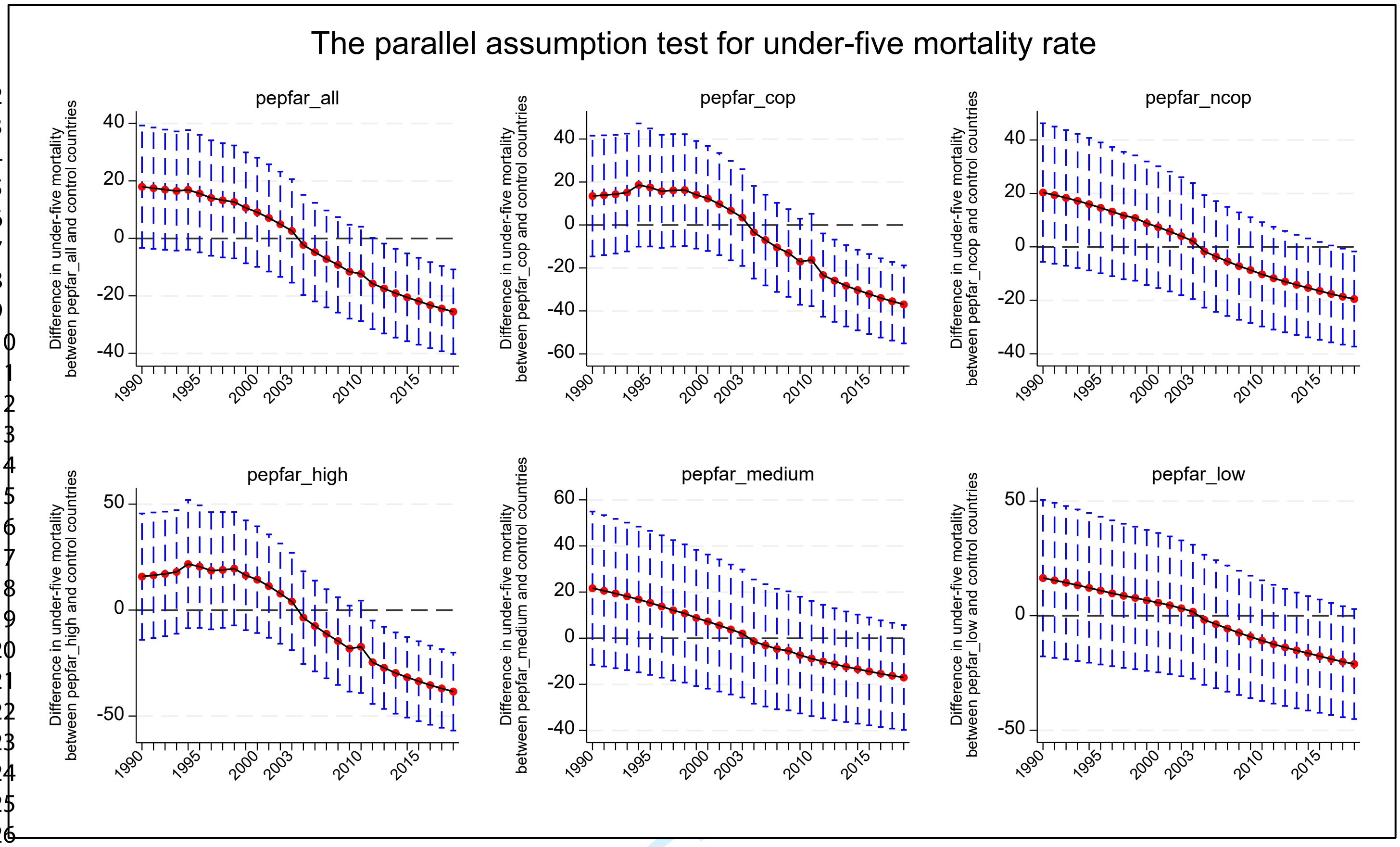

Notes: pepfar_all = all PEPFAR country cohort; pepfar_cop = COP PEPFAR country cohort; pepfar_ncop = non-COP PEPFAR country cohort; pepfar_high = high-intensity PEPFAR country cohort; pepfar_medium = medium-intensity PEPFAR country cohort; pepfar_low = low-intensity PEPFAR country cohort. COP= country operational plans; PEPFAR= President's Emergency Plan for AIDS Relief

S7-7. Prevalence of anemia among women of reproductive age

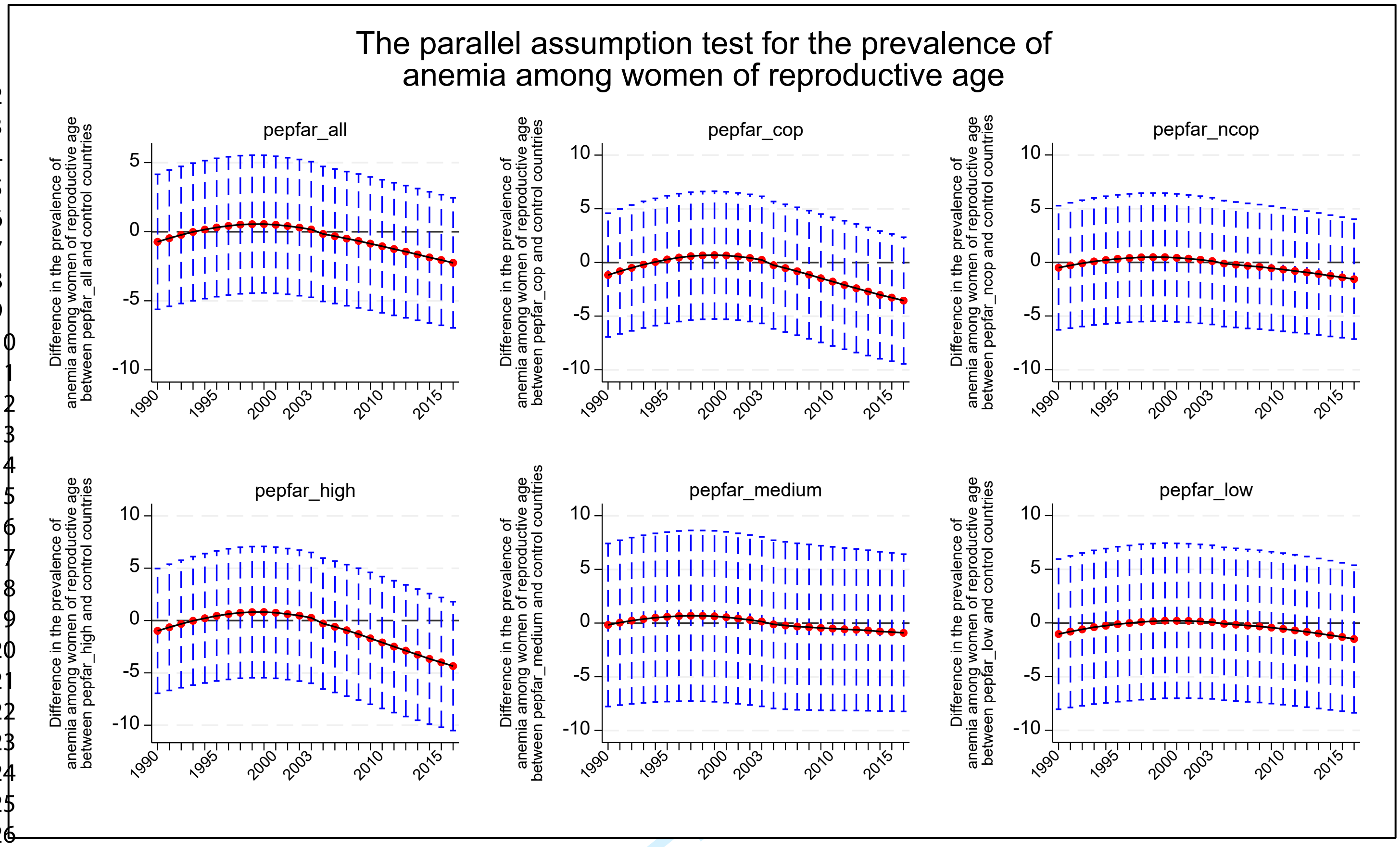

Notes: pepfar_all = all PEPFAR country cohort; pepfar_cop = COP PEPFAR country cohort; pepfar_ncop = non-COP PEPFAR country cohort; pepfar_high = high-intensity PEPFAR country cohort; pepfar_medium = medium-intensity PEPFAR country cohort; pepfar_low = low-intensity PEPFAR country cohort. COP= country operational plans; PEPFAR= President's Emergency Plan for AIDS Relief

S8. Parallel pre-trend assumption test for staggered DID

Figures in supplement S4 illustrate the dynamic impacts of PEPFAR as estimated by D_{it}^{T} from equation (4).

There are two key findings for the four immunization rates (see S4-1 to S4-4). First, improvements in hepatitis B immunization rate did not precede the PEPFAR program for all six country cohorts, as shown as D_{it}^{T} that are insignificantly different from zero (eg. without trends for all the years before PEPFAR started). A similar finding is also true for the COP and high-intensity country cohorts in DPT, measles, and tetanus immunization rates, but not for the other country cohorts of these three immunization rates. This verifies the internal validity of the staggered DID design for the six cohorts in hepatitis B immunization rate and for the COP and high-intensity country cohorts in DPT, measles, and tetanus immunization rates.

Second, for hepatitis B immunization rate, PEPFAR materialized its impact quickly in the COP country cohort, represented by the immediate jump of the 95% confidence interval that is right after the program started. Furthermore, PEPFAR gradually increased its magnitude of impact in the all PEPFAR country, COP country, and high-intensity country cohorts but leveled off or even diminished in the other three country cohorts. A similar impact pattern was observed for DPT and measles immunization rates, but not for the tetanus immunization rate.

Several patterns were observed for maternal and child mortality rate, as well as the prevalence of amenia among women of reproductive age. Although all the country cohorts have statistically insignificant D_{it}^{T}s prior to PEPFAR in terms of maternal mortality rate, D_{it}^{T}s exhibited some degree of declining trend before recipient countries joined the PEPFAR program (see S4-5). The existence of this preceding trend suggested limited internal validity for the staggered DID method in estimating the PEPFAR spillover effects on maternal mortality ratios. In terms of child mortality rate, a pattern of flat D_{it}^{T}s that are prior to PEPFAR started is evident for the COP and high-intensity country segments but not for other segments, demonstrating the good internal validity for the COP and high-intensity country cohorts but not for the other cohorts. Additionally, the impact of PEPFAR on child mortality rate becomes evident rapidly in COP and high-intensity countries, marked as an immediate decline for the 95% confidence intervals right after the cohorts started the PEPFAR program. No evident trend is observed before or after the start of the PEPFAR program for the prevalence of anemia among women of reproductive age, consistent with the inconclusive coefficients estimated by staggered DID.

For peer review only

S8-1. DPT immunization rate

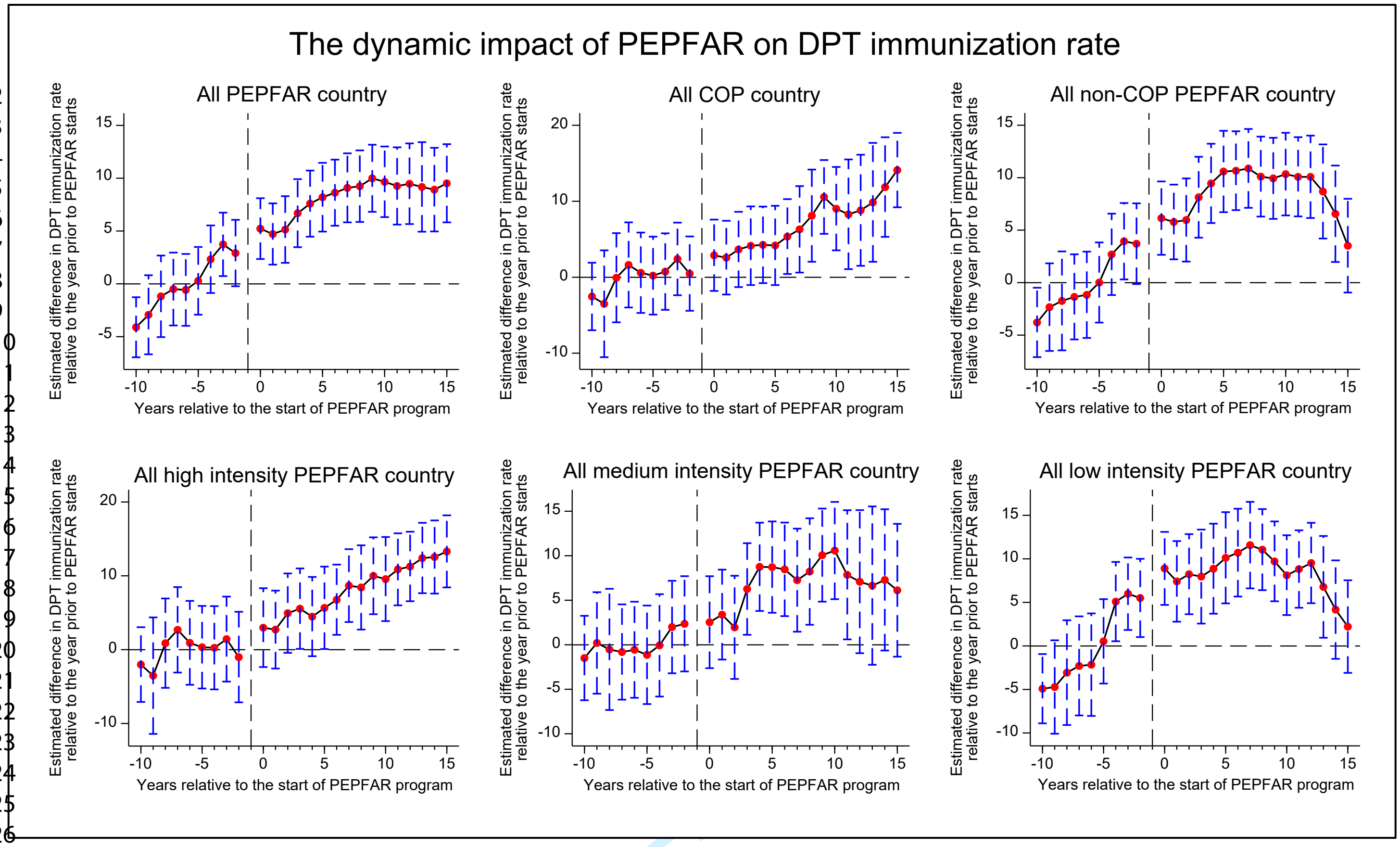

Notes: pepfar_all = all PEPFAR country cohort; pepfar_cop = COP PEPFAR country cohort; pepfar_ncop = non-COP PEPFAR country cohort; pepfar_high = high-intensity PEPFAR country cohort; pepfar_medium = medium-intensity PEPFAR country cohort; pepfar_low = low-intensity PEPFAR country cohort. COP= country operational plans; DPT=diphtheria, pertussis, tetanus; PEPFAR= President's Emergency Plan for AIDS Relief

S8-2. Hepatitis B immunization rate

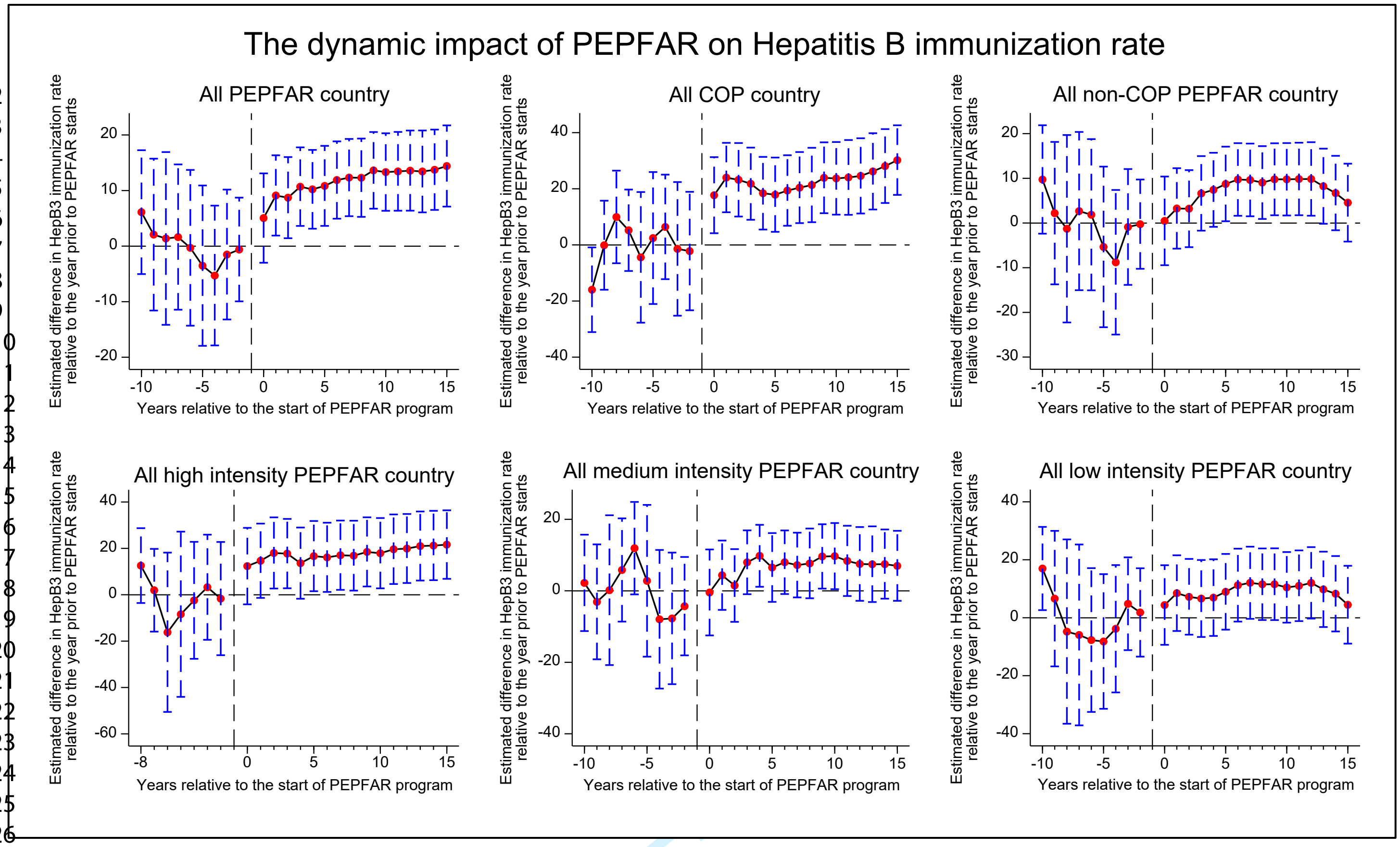

Notes: pepfar_all = all PEPFAR country cohort; pepfar_cop = COP PEPFAR country cohort; pepfar_ncop = non-COP PEPFAR country cohort; pepfar_high = high-intensity PEPFAR country cohort; pepfar_medium = medium-intensity PEPFAR country cohort; pepfar_low = low-intensity PEPFAR country cohort. COP= country operational plans; PEPFAR= President's Emergency Plan for AIDS Relief

S8-3. Measles immunization rate

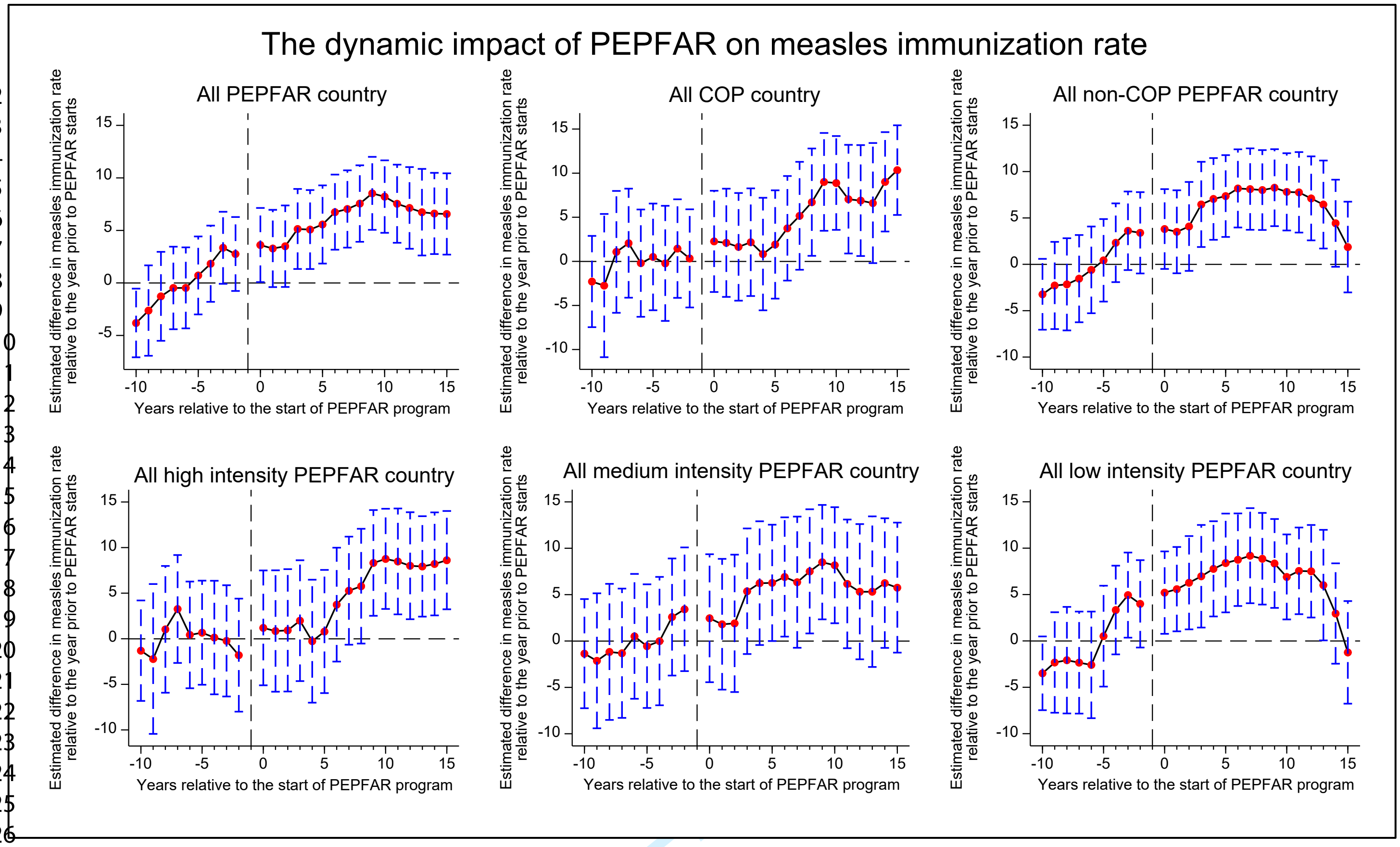

Notes: pepfar_all = all PEPFAR country cohort; pepfar_cop = COP PEPFAR country cohort; pepfar_ncop = non-COP PEPFAR country cohort; pepfar_high = high-intensity PEPFAR country cohort; pepfar_medium = medium-intensity PEPFAR country cohort; pepfar_low = low-intensity PEPFAR country cohort. COP= country operational plans; PEPFAR= President's Emergency Plan for AIDS Relief

For peer review only

S8-4. Prevalence of newborns against tetanus

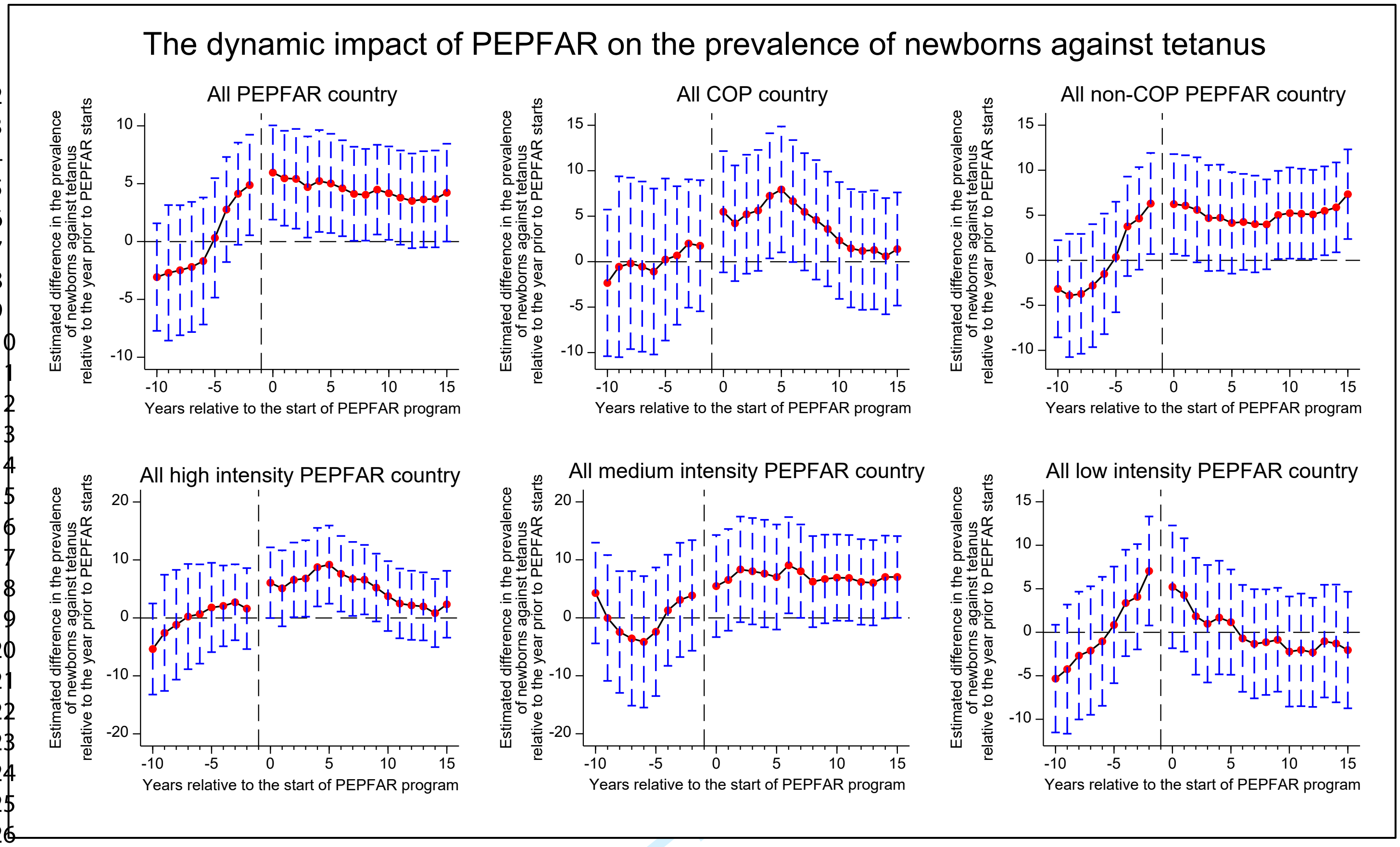

Notes: pepfar_all = all PEPFAR country cohort; pepfar_cop = COP PEPFAR country cohort; pepfar_ncop = non-COP PEPFAR country cohort; pepfar_high = high-intensity PEPFAR country cohort; pepfar_medium = medium-intensity PEPFAR country cohort; pepfar_low = low-intensity PEPFAR country cohort. COP= country operational plans; PEPFAR= President's Emergency Plan for AIDS Relief

S8-5. Maternal mortality ratio

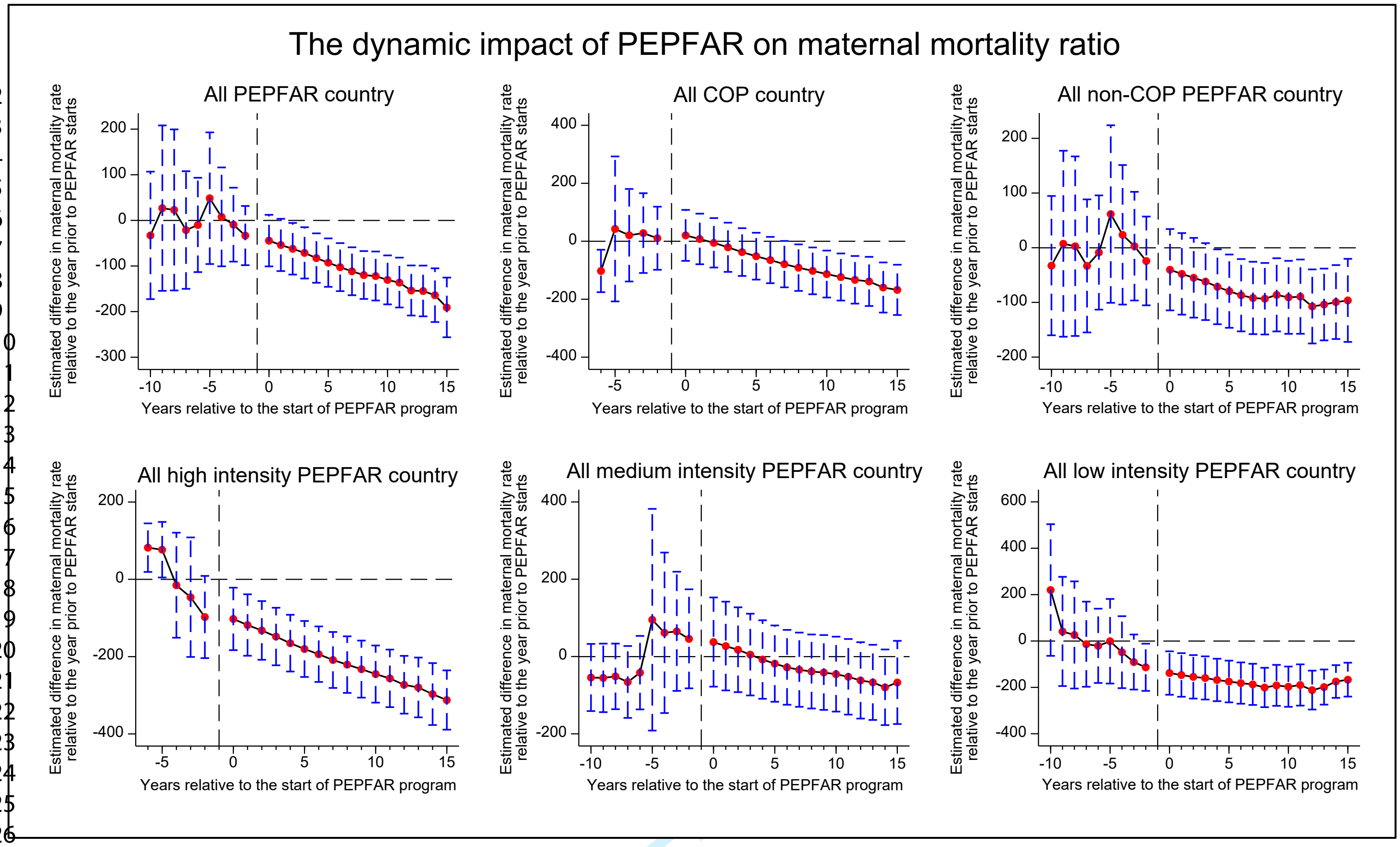

Notes: pepfar_all = all PEPFAR country cohort; pepfar_cop = COP PEPFAR country cohort; pepfar_ncop = non-COP PEPFAR country cohort; pepfar_high = high-intensity PEPFAR country cohort; pepfar_medium = medium-intensity PEPFAR country cohort; pepfar_low = low-intensity PEPFAR country cohort. COP= country operational plans; PEPFAR= President's Emergency Plan for AIDS Relief

S8-6. Child (under-five) mortality rate

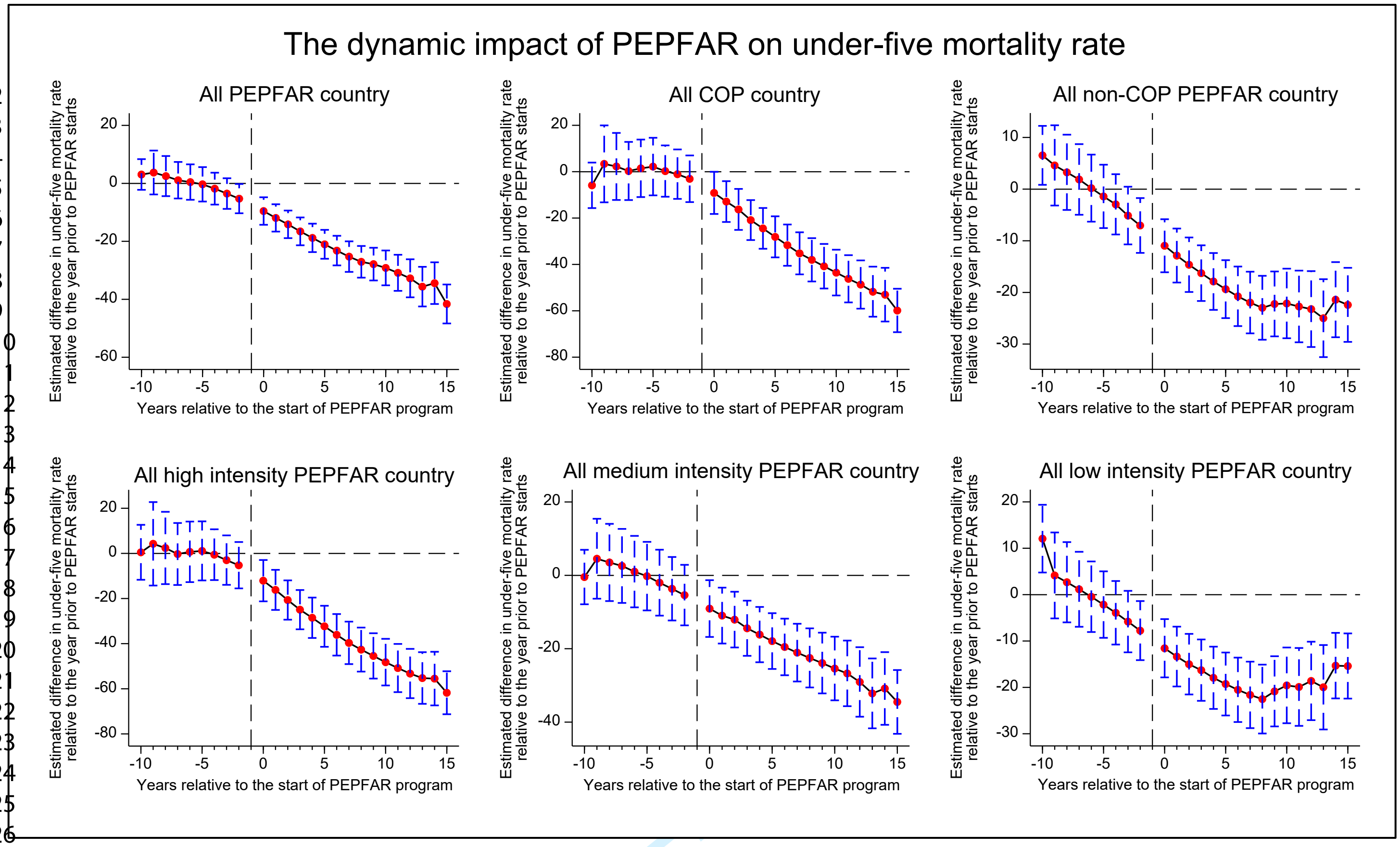

Notes: pepfar_all = all PEPFAR country cohort; pepfar_cop = COP PEPFAR country cohort; pepfar_ncop = non-COP PEPFAR country cohort; pepfar_high = high-intensity PEPFAR country cohort; pepfar_medium = medium-intensity PEPFAR country cohort; pepfar_low = low-intensity PEPFAR country cohort. COP= country operational plans; PEPFAR= President's Emergency Plan for AIDS Relief

S8-7. Prevalence of anemia among women of reproductive age

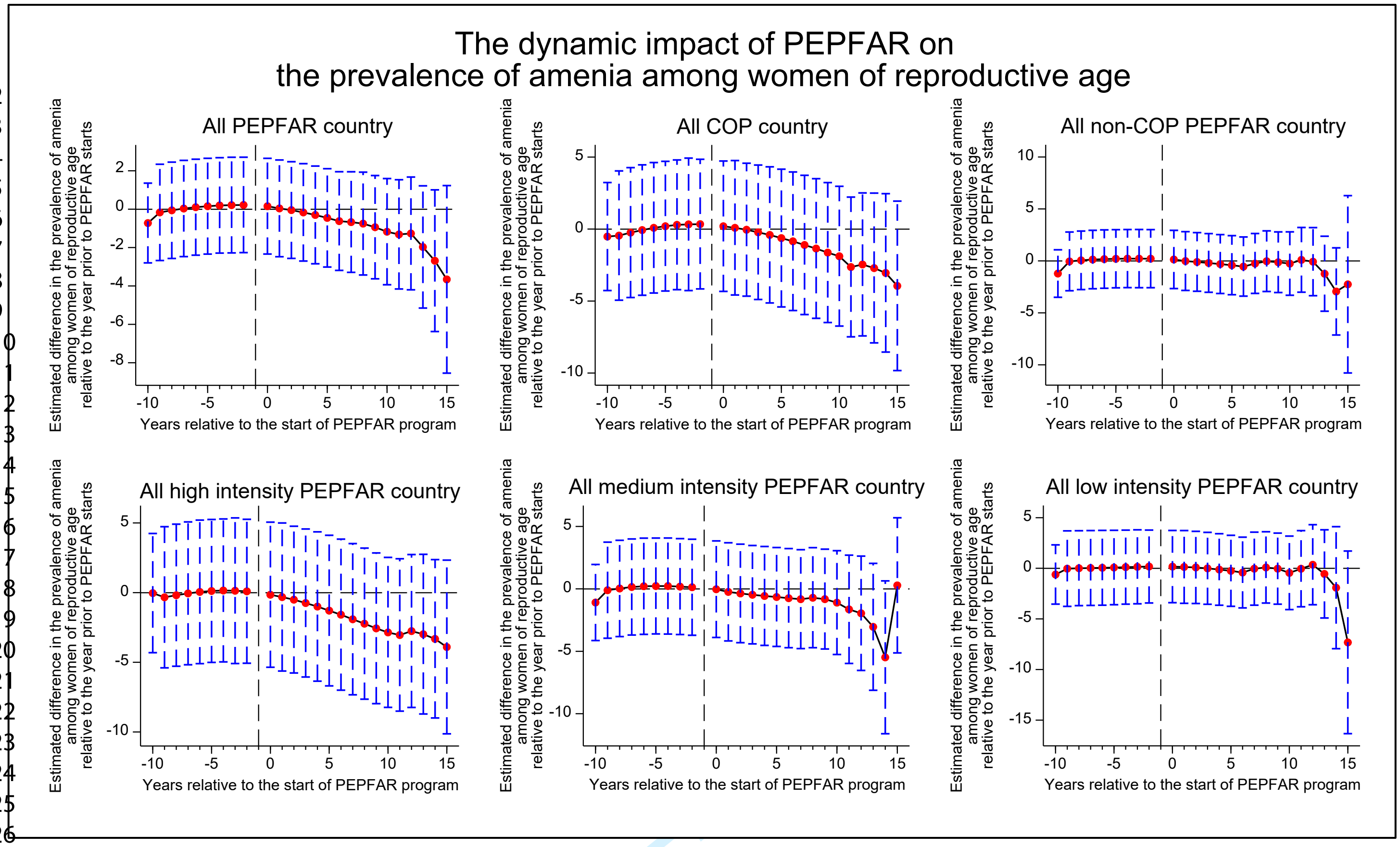

Notes: pepfar_all = all PEPFAR country cohort; pepfar_cop = COP PEPFAR country cohort; pepfar_ncop = non-COP PEPFAR country cohort; pepfar_high = high-intensity PEPFAR country cohort; pepfar_medium = medium-intensity PEPFAR country cohort; pepfar_low = low-intensity PEPFAR country cohort. COP= country operational plans; PEPFAR= President's Emergency Plan for AIDS Relief

S9. Placebo test for staggered DID

S9-1. DPT immunization rate

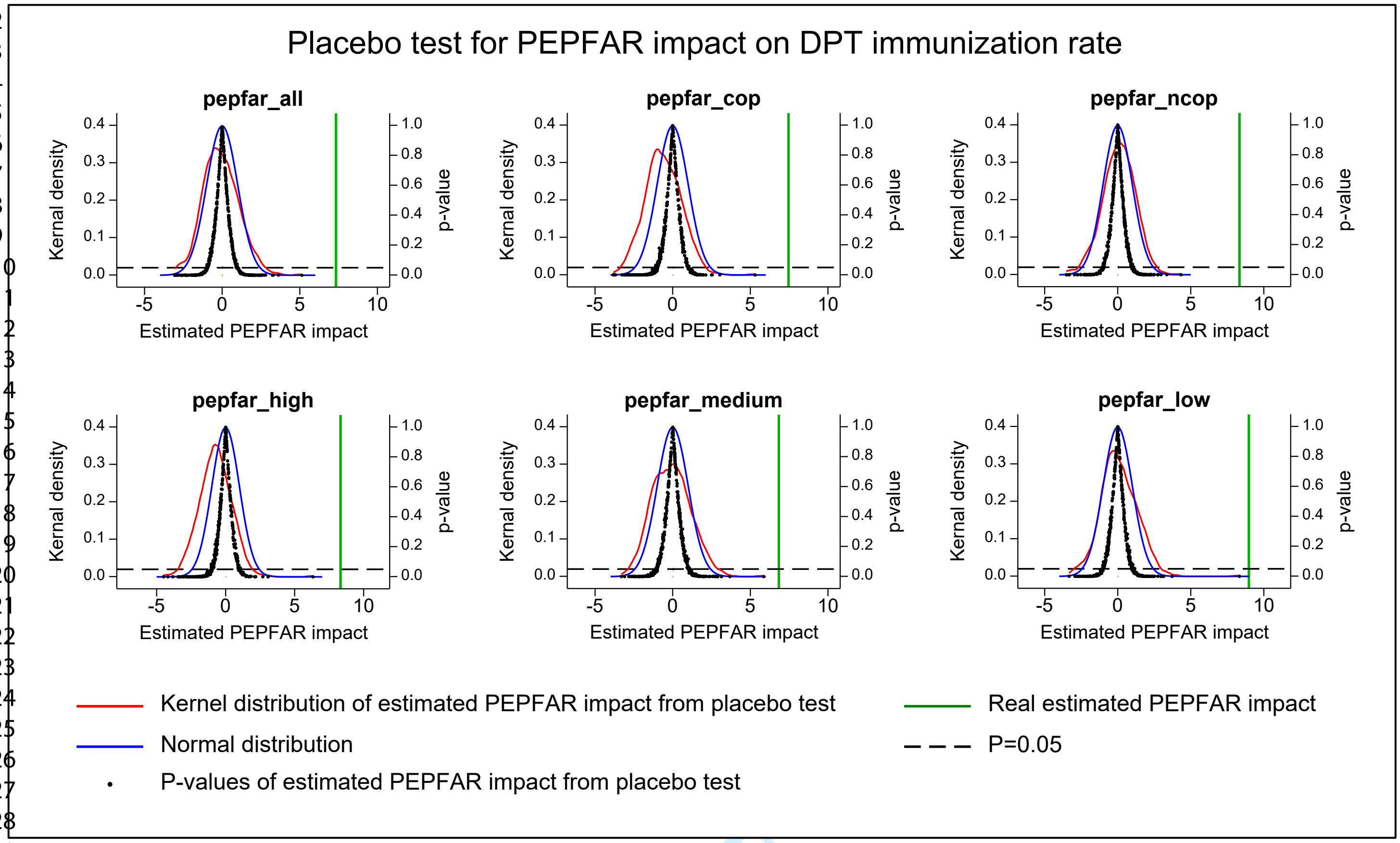

Notes: pepfar_all = all PEPFAR country cohort; pepfar_cop = COP PEPFAR country cohort; pepfar_ncop = non-COP PEPFAR country cohort; pepfar_high = high-intensity PEPFAR country cohort; pepfar_medium = medium-intensity PEPFAR country cohort; pepfar_low = low-intensity PEPFAR country cohort. COP= country operational plans; DID=difference-in-differences; PEPFAR= President's Emergency Plan for AIDS Relief

S9-2. Hepatitis B immunization rate

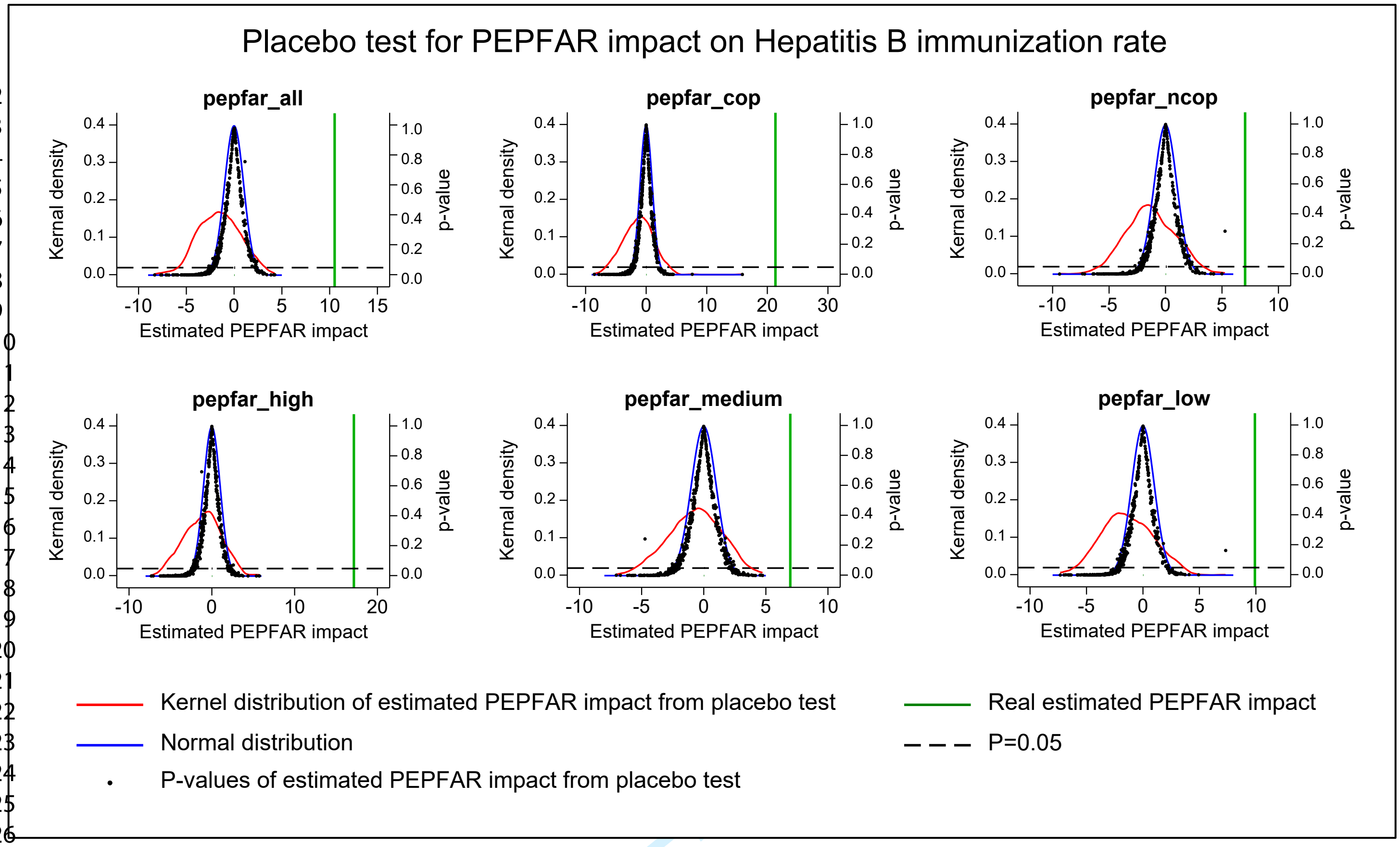

Notes: pepfar_all = all PEPFAR country cohort; pepfar_cop = COP PEPFAR country cohort; pepfar_ncop = non-COP PEPFAR country cohort; pepfar_high = high-intensity PEPFAR country cohort; pepfar_medium = medium-intensity PEPFAR country cohort; pepfar_low = low-intensity PEPFAR country cohort. COP= country operational plans; PEPFAR= President's Emergency Plan for AIDS Relief

For peer review only

S9-3. Measles immunization rate

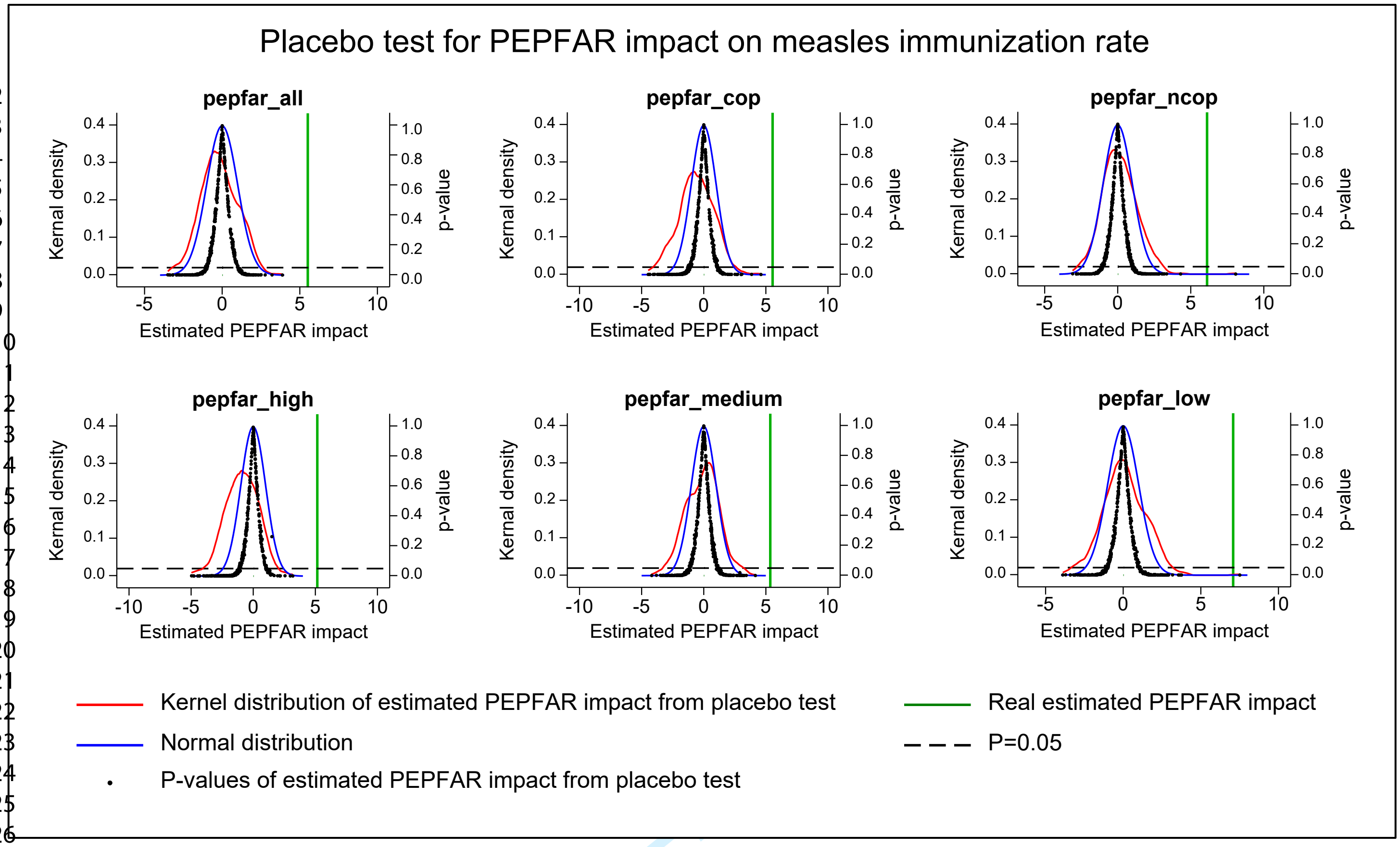

Notes: pepfar_all = all PEPFAR country cohort; pepfar_cop = COP PEPFAR country cohort; pepfar_ncop = non-COP PEPFAR country cohort; pepfar_high = high-intensity PEPFAR country cohort; pepfar_medium = medium-intensity PEPFAR country cohort; pepfar_low = low-intensity PEPFAR country cohort. COP= country operational plans; PEPFAR= President's Emergency Plan for AIDS Relief

S9-4. Prevalence of newborns against tetanus

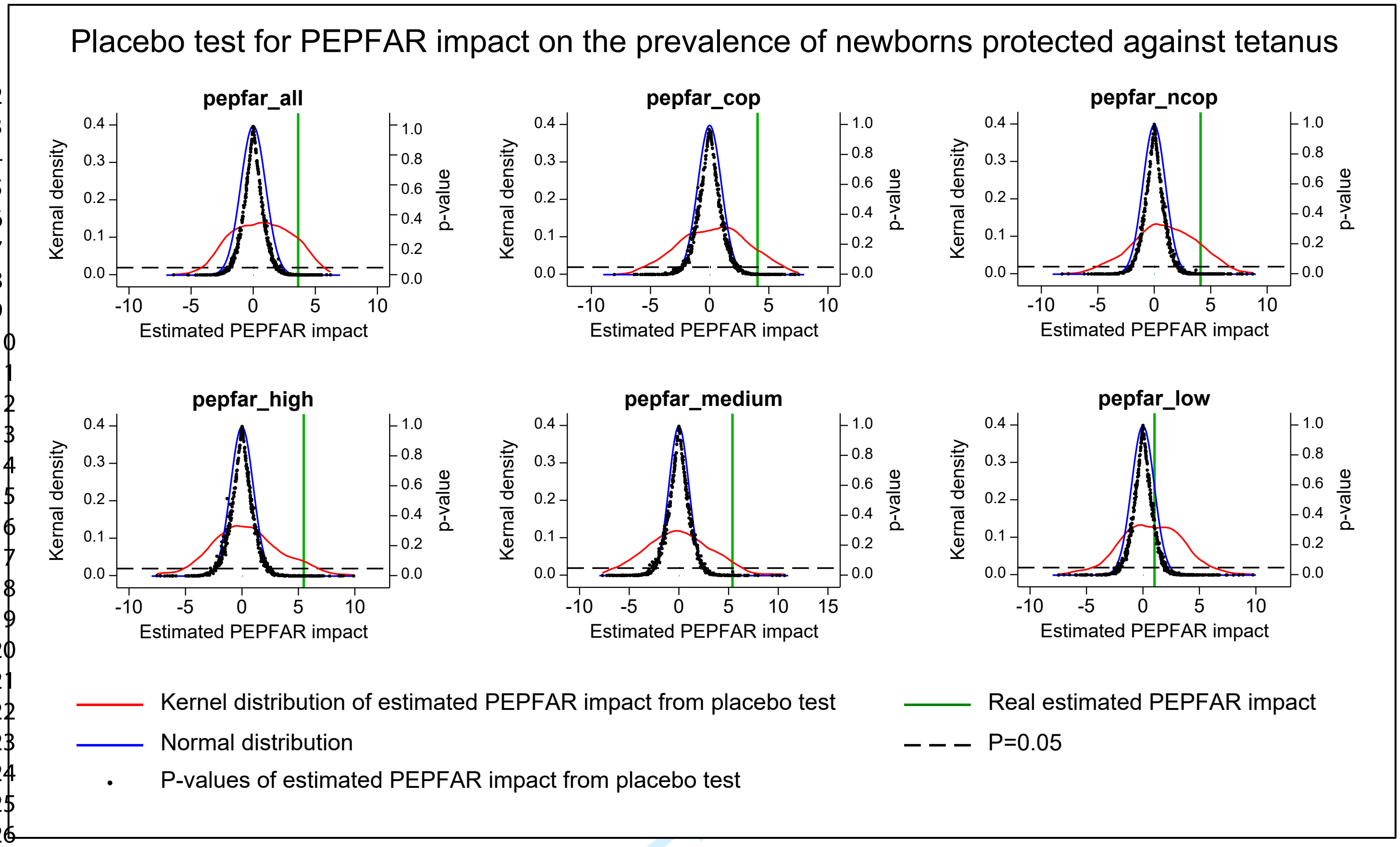

Notes: pepfar_all = all PEPFAR country cohort; pepfar_cop = COP PEPFAR country cohort; pepfar_ncop = non-COP PEPFAR country cohort; pepfar_high = high-intensity PEPFAR country cohort; pepfar_medium = medium-intensity PEPFAR country cohort; pepfar_low = low-intensity PEPFAR country cohort. COP= country operational plans; PEPFAR= President's Emergency Plan for AIDS Relief

For peer review only

S9-5. Maternal mortality ratio

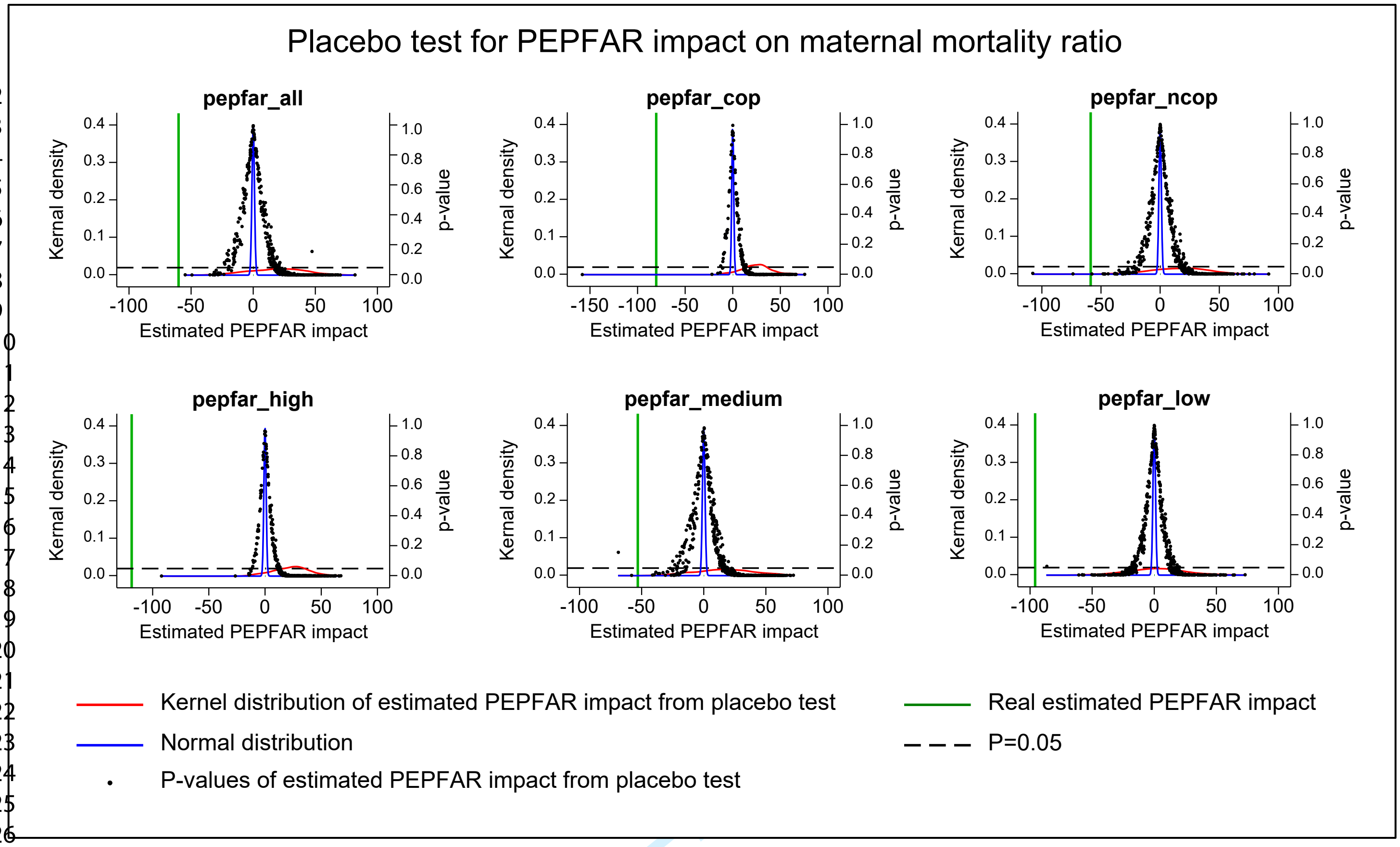

Notes: pepfar_all = all PEPFAR country cohort; pepfar_cop = COP PEPFAR country cohort; pepfar_ncop = non-COP PEPFAR country cohort; pepfar_high = high-intensity PEPFAR country cohort; pepfar_medium = medium-intensity PEPFAR country cohort; pepfar_low = low-intensity PEPFAR country cohort. COP= country operational plans; PEPFAR= President's Emergency Plan for AIDS Relief

S9-6. Under-five mortality rate

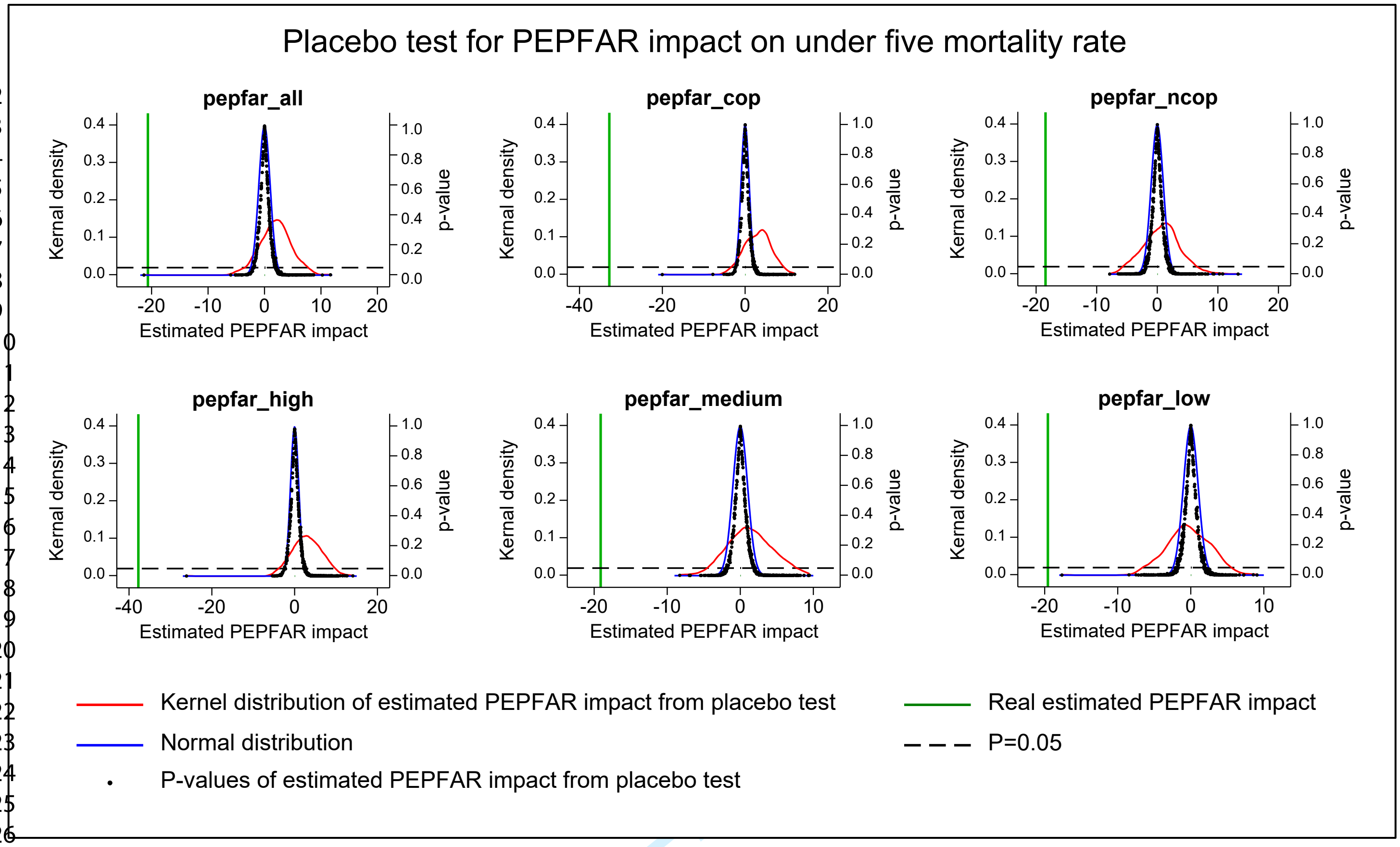

Notes: pepfar_all = all PEPFAR country cohort; pepfar_cop = COP PEPFAR country cohort; pepfar_ncop = non-COP PEPFAR country cohort; pepfar_high = high-intensity PEPFAR country cohort; pepfar_medium = medium-intensity PEPFAR country cohort; pepfar_low = low-intensity PEPFAR country cohort. COP= country operational plans; PEPFAR= President's Emergency Plan for AIDS Relief

For peer review only

S9-7. Prevalence of anemia among women of reproductive age

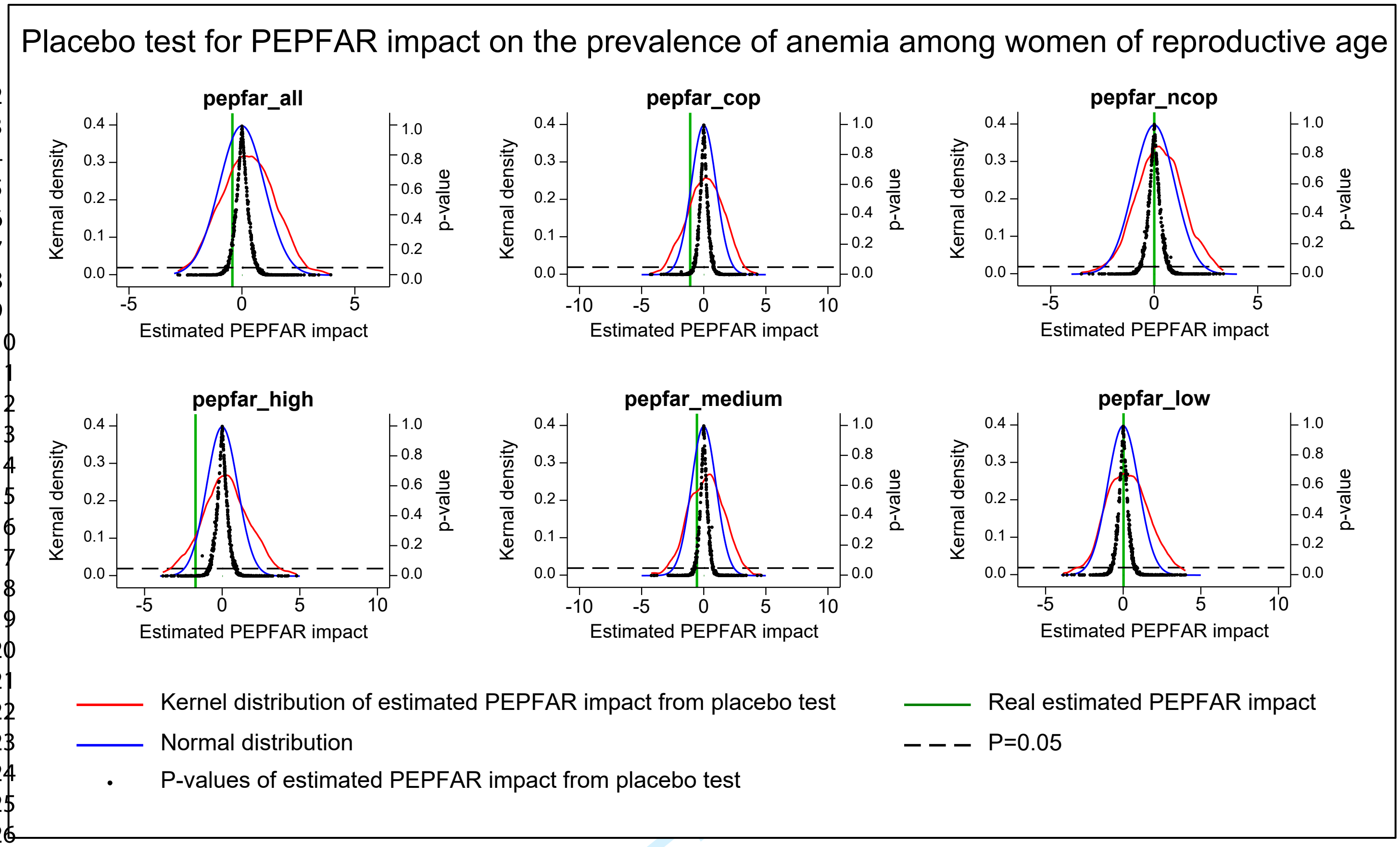

Notes: pepfar_all = all PEPFAR country cohort; pepfar_cop = COP PEPFAR country cohort; pepfar_ncop = non-COP PEPFAR country cohort; pepfar_high = high-intensity PEPFAR country cohort; pepfar_medium = medium-intensity PEPFAR country cohort; pepfar_low = low-intensity PEPFAR country cohort. COP= country operational plans; PEPFAR= President's Emergency Plan for AIDS Relief

S10. Estimation results from logarithmically transformed equations 1 and 3

S10-1. PEPFAR impacts on four immunization rates: traditional and staggered DID estimates

Outcomes and estimation methods	All PEPFAR countries	COP countries	Non-COP countries	High intensity PEPFAR countries	Medium intensity PEPFAR countries	Low intensity PEPFAR countries
**DPT immunization rate**						
Model 1. Unadjusted model (traditional)	0.157***	0.160***	0.154***	0.172***	0.102***	0.193***
Model 1. Unadjusted model (staggered)	0.147***	0.122***	0.190***	0.145***	0.0806***	0.261***
Model 2. Adjusted model (traditional)	0.171***	0.186***	0.165***	0.159***	0.143***	0.214***
Model 2. Adjusted model (staggered)	0.135***	0.155***	0.144***	0.134***	0.141***	0.179***
**Hepatitis B immunization rate**						
Model 1. Unadjusted model (traditional)	0.0894	0.194*	0.0350	0.0669	0.162	0.0121
Model 1. Unadjusted model (staggered)	0.173***	0.404***	0.119*	0.313*	0.134	0.172*
Model 2. Adjusted model (traditional)	0.185**	0.262**	0.172*	0.148	0.220*	0.194*
Model 2. Adjusted model (staggered)	0.252***	0.446***	0.200***	0.353*	0.191**	0.255**
**Measles immunization rate**						
Model 1. Unadjusted model (traditional)	0.118***	0.129***	0.110***	0.118***	0.0769***	0.155***
Model 1. Unadjusted model (staggered)	0.119***	0.0970***	0.151***	0.0972***	0.0629**	0.224***
Model 2. Adjusted model (traditional)	0.124***	0.143***	0.115***	0.105***	0.100***	0.168***
Model 2. Adjusted model (staggered)	0.104***	0.111***	0.104***	0.0778***	0.109***	0.135***
**Prevalence of newborns protected against tetanus**						
Model 1. Unadjusted model (traditional)	0.109***	0.0918**	0.122***	0.109***	0.0979**	0.119***
Model 1. Unadjusted model (staggered)	0.0947***	0.107***	0.103***	0.125***	0.0874**	0.104***
Model 2. Adjusted model (traditional)	0.0978***	0.0747*	0.111***	0.0954**	0.0958**	0.0973**
Model 2. Adjusted model (staggered)	0.0697***	0.0813**	0.0729***	0.0993**	0.0947**	0.0190

***p < 0.001 **p < 0.01 * p< 0.05. Notes: COP=country operational plans; DPT=diphtheria, pertussis, tetanus; PEPFAR= President's Emergency Plan for AIDS Relief

S10-2. PEPFAR impacts on maternal mortality ratio, child (under-five) mortality rate, and the prevalence of anemia among women of reproductive age: traditional and staggered DID estimates

Outcomes and methods	All PEPFAR countries	COP countries	Non-COP countries	High intensity PEPFAR countries	Medium intensity PEPFAR countries	Low intensity PEPFAR countries
**Maternal mortality ratio**						
Model 1. Unadjusted model (traditional)	-0.0337	-0.0171	-0.0424	-0.00368	-0.0530	-0.0444
Model 1. Unadjusted model (staggered)	-0.0406	0.0448	-0.347**	-0.0987	0.383**	-0.881***
Model 2. Adjusted model (traditional)	-0.0358	-0.0504	-0.0282	-0.0275	-0.0612	-0.0177
Model 2. Adjusted model (staggered)	0.0594	0.0214	0.0696	0.00106	-0.0257	0.0251
**Child (under-five) mortality rate**						
Model 1. Unadjusted model (traditional)	-0.0390	0.00754	-0.0634	-0.00887	-0.0656	-0.0425
Model 1. Unadjusted model (staggered)	-0.0147	0.0326	-0.135**	-0.00670	0.103	-0.306***
Model 2. Adjusted model (traditional)	-0.0860**	-0.0516	-0.104***	-0.0759*	-0.0963**	-0.0854**
Model 2. Adjusted model (staggered)	-0.0521**	-0.0394	-0.0758***	-0.0620*	-0.0907***	-0.0750**
**Prevalence of anemia among women of reproductive age**						
Model 1. Unadjusted model (traditional)	-0.0116	-0.0224	-0.00592	-0.0409	-0.000701	0.00682
Model 1. Unadjusted model (staggered)	-0.0189	-0.000761	-0.0484*	-0.0211	0.0506	-0.103***
Model 2. Adjusted model (traditional)	0.0140	0.00718	0.0176	-0.0106	0.0219	0.0305
Model 2. Adjusted model (staggered)	0.0130	0.0138	0.0200	-0.00408	0.00721	0.0145

***p < 0.001 **p < 0.01 * p< 0.05. Notes: COP=country operational plans; PEPFAR= President's Emergency Plan for AIDS Relief

For peer review only

** p<0.001, ** p<0.01, * p<0.05   BL=baseline; COP=country operational plans; PEPFAR= President's Emergency Plan for AIDS Relief

For peer review only

For peer review only
